# DNA methylation analysis to differentiate reference, breed, and parent-of-origin effects in the bovine pangenome era

**DOI:** 10.1093/gigascience/giae061

**Published:** 2024-10-17

**Authors:** Callum MacPhillamy, Tong Chen, Stefan Hiendleder, John L Williams, Hamid Alinejad-Rokny, Wai Yee Low

**Affiliations:** The Davies Research Centre, School of Animal and Veterinary Sciences, The University of Adelaide, Roseworthy SA 5371, Australia; The Davies Research Centre, School of Animal and Veterinary Sciences, The University of Adelaide, Roseworthy SA 5371, Australia; The Davies Research Centre, School of Animal and Veterinary Sciences, The University of Adelaide, Roseworthy SA 5371, Australia; Robinson Research Institute,, The University of Adelaide, North Adelaide SA 5006, Australia; The Davies Research Centre, School of Animal and Veterinary Sciences, The University of Adelaide, Roseworthy SA 5371, Australia; Department of Animal Science, Food and Nutrition, Università Cattolica del Sacro Cuore, 29122 Piacenza, Italy; BioMedical Machine Learning Lab, The Graduate School of Biomedical Engineering, Univeristy of New South Wales, Sydney, NSW 2052, Australia; The Davies Research Centre, School of Animal and Veterinary Sciences, The University of Adelaide, Roseworthy SA 5371, Australia

**Keywords:** bisulfite sequencing, methylation, CpG, structural variants, *Dgat1*, differentially methylated region, bovine pangenome

## Abstract

**Background:**

Most DNA methylation studies have used a single reference genome with little attention paid to the bias introduced due to the reference chosen. Reference genome artifacts and genetic variation, including single nucleotide polymorphisms (SNPs) and structural variants (SVs), can lead to differences in methylation sites (CpGs) between individuals of the same species. We analyzed whole-genome bisulfite sequencing data from the fetal liver of Angus (*Bos taurus taurus*), Brahman (*Bos taurus indicus*), and reciprocally crossed samples. Using reference genomes for each breed from the Bovine Pangenome Consortium, we investigated the influence of reference genome choice on the breed and parent-of-origin effects in methylome analyses.

**Results:**

Our findings revealed that ∼75% of CpG sites were shared between Angus and Brahman, ∼5% were breed specific, and ∼20% were unresolved. We demonstrated up to ∼2% quantification bias in global methylation when an incorrect reference genome was used. Furthermore, we found that SNPs impacted CpGs 13 times more than other autosomal sites (*P* < $5 \times {10}^{ - 324}$) and SVs contained 1.18 times (*P* < $5 \times {10}^{ - 324}$) more CpGs than non-SVs. We found a poor overlap between differentially methylated regions (DMRs) and differentially expressed genes (DEGs) and suggest that DMRs may be impacting enhancers that target these DEGs. DMRs overlapped with imprinted genes, of which 1, *DGAT1*, which is important for fat metabolism and weight gain, was found in the breed-specific and sire-of-origin comparisons.

**Conclusions:**

This work demonstrates the need to consider reference genome effects to explore genetic and epigenetic differences accurately and identify DMRs involved in controlling certain genes.

## Background

DNA methylation is a key epigenetic modification that plays a vital role in regulating gene expression, repression of transposable elements, and parental chromosome-specific regulation through genomic imprinting and X-chromosome inactivation [1, 2]. In mammals, DNA methylation primarily occurs at C-phosphate-G dinucleotides (CpGs) [3, 4]. DNA methylation influences gene expression either by recruiting proteins involved in gene repression or by blocking transcription factor binding sites (TFBSs) within promoter regions [5]. Hypomethylation of a promoter has been associated with the increased expression of the corresponding gene [6]. However, recent work has shown that promoter hypermethylation can also lead to gene expression [7]. The relationship between DNA methylation and gene expression is complicated by the role of enhancer methylation in regulating gene expression [8, 9]. In the presence of high DNA methylation, enhancers have been observed to be associated with high levels of the histone modification H3K27ac [10], which is often associated with active gene transcription [11–14].

Most DNA methylation studies have used a single reference genome with little or no knowledge of the impact of reference genome choice on the interpretation of methylome differences. The choice of reference genome has been shown to have an impact on DNA methylation analyses, with up to a 9% bias reported when the incorrect reference is used [15]. Using a single reference genome has been shown to bias read mapping in favor of reads with high similarity to the reference [16–20]. This bias occurs because reads containing nonreference alleles or regions that are divergent from the reference align poorly, align to the wrong genomic region, or fail to align. This reference bias has been shown to affect analyses of cattle breeds [21, 22], humans [17, 23], and sheep [18].

The majority of mammalian methylation occurs in the CpG context. Consequently, a single nucleotide polymorphism (SNP) can remove a methylation site, thus introducing a reference bias if the individuals being studied do not possess the same SNPs as the individual used to generate the reference. In addition to SNPs, structural variations (SVs) among individuals may remove or introduce CpG sites. The disparity between CpG sites can confound analyses by identifying a methylated CpG in one individual when another individual has no CpG at that position. As a result, SNPs and SVs can both introduce bias, as reads may be unambiguously assigned in duplicated regions not found in the reference, and mismatches in reads can result in the loss of some reads. Moreover, if individuals have insertion SVs that carry CpG sites, reads that originate from the insertion/deletion (IGF) regions can only be mapped if the complete sequence data for the population is available. We consider SNPs and SVs that alter CpGs as genetic changes with potential effects on epigenetic regulation. We use the term “genetics of epigenetics” to describe this phenomenon.

As more genomes for a given species become available, the research community is gradually shifting toward using pangenomes to account for genetic variation within a population more accurately. A pangenome is a collection of the genomes of multiple individuals, representing all genetic variation within that population and is thus a more accurate way to represent genetic diversity than a single reference genome [24]. Current pangenome projects include human [24, 25], cattle [26], and maize [27]. As genetic differences within a population can result in CpG differences, these pangenomes provide a valuable resource to study DNA methylation changes between diverse groups of individuals of the same species.

The 2 main lineages of modern cattle breeds are generally accepted to have been derived from 2 separate domestication events of the wild auroch (*Bos primigenius*) [28]. The first domestication event occurred in the Fertile Crescent around 10,000 years ago and gave rise to *Bos taurus taurus* from the wild auroch, *B. p. primigenius* [29–31]. A second domestication event occurred in the Indus Valley, ∼1,500 years later, from *Bos primigenius nomadicus*, which separated from *B. p. primigenius* around 250,000 to 330,000 years ago [32] and gave rise to *Bos taurus indicus*. The subspecies are referred to here as taurine and indicine cattle, respectively [28], where the Angus breed represents taurine cattle, and Brahman is representative of indicine cattle.

Angus and Brahman have contrasting phenotypes; for example, Angus have been bred for meat production traits [33], whereas Brahman have superior heat and disease tolerance traits [34, 35]. DNA methylation differences may partly be responsible for the phenotypic differences between these 2 breeds.

As expected from their domestication history, Angus and Brahman cattle represent genetically highly diverged subspecies [36, 37]. However, as they produce fertile offspring when mated [38], they are an appropriate model to investigate the impact of using a single reference genome on methylome analysis of 2 genetically diverse populations. We have previously produced high-quality haplotype-resolved reference genomes for Angus and Brahman [39], which are genomes included in the Bovine Pangenome Consortium project [26] and are used in the present study.

Breed-specific differences in CpGs may occur due to a SNP, such as those caused by spontaneous deamination [40, 41], or may result from SVs. A single SNP affecting a CpG site has been shown to drastically alter the methylation state of the *IGF2* gene in pigs, leading to changes in muscle development [42]. SVs have been associated with decreased methylation in cancers [43] and with changes in the methylation of the kappa opioid receptor (*KOR*) promoter associated with KOR dysfunction and schizophrenia [44].

Parent-of-origin effects (POEs) occur when only 1 allele is expressed, and the phenotype in the offspring may depend on which parent contributed the expressed allele [45]. Reciprocal crossing is necessary to elucidate how each parent contributes to a particular phenotype. POEs have been observed in hybrids of mice [46], cattle [47], and pigs [48], and there is increasing evidence that fetal development is influenced by POEs [49–53]. Given the similarity in gestation period between cattle and human and the single fetus with a similar development trajectory, cattle are an attractive model species to study human reproductive and developmental biology [54–57].

To investigate the potential impact of reference genome choice on methylome analyses and to improve our understanding of the genetic and epigenetic factors driving the phenotypic differences between cattle subspecies, we used whole-genome bisulfite sequencing (WGBS) data from 24 fetal liver samples of purebred Brahman and Angus cattle and their reciprocal crosses to perform a comprehensive assessment of the impact of reference genome choice on differential methylation and gene expression. This study serves as an example of how to investigate epigenetic differences between breeds, strains, and populations within species and informs about reference genome effects on the interpretation of methylome analyses.

## Results

### Mapping of WGBS data and calling CpG

**Figure 1:**
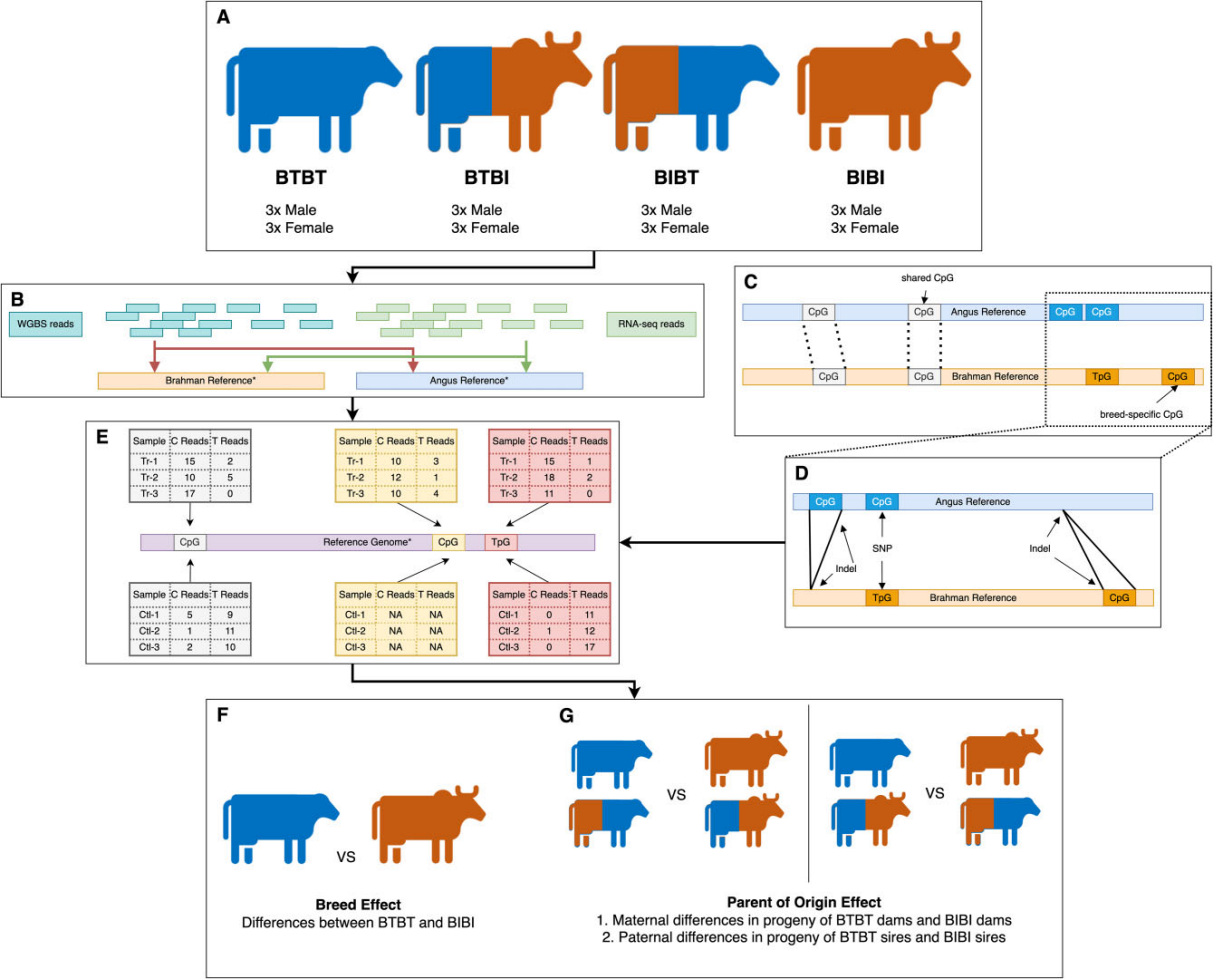
Overview of methods. (A) Representation of the 4 genetic groups used in this study. The blue cow represents pure Angus individuals (BTBT). The blue then orange cow represents individuals with an Angus sire and Brahman dam (BTBI). The orange then blue cow represents individuals with a Brahman sire and Angus dam (BIBT). The orange cow represents pure Brahman individuals (BIBI). (B) Process of mapping WGBS reads (light green-blue) and RNA sequencing reads (green) to both the Brahman and Angus reference genomes. (C) Simple representation of shared and breed-specific CpG sites between Brahman and Angus reference genomes. (D) Breed-specific CpGs arise from a single nucleotide polymorphism between Brahman and Angus, such as spontaneous deamination of the C to a T. Structural variants, such as indels between the 2 genomes, can introduce or remove CpGs in one genome relative to the other. (E) Simple representation of how differential methylation can be influenced by breed-specific CpGs. The gray boxes demonstrate how a differentially methylated cytosine is identified when both breeds share that site. Essentially, one compares the number of Cs and Ts in group 1 against the number of Cs and Ts in group 2. If 1 group reports significantly more Cs than the other, it is considered differentially methylated. The yellow boxes represent a breed-specific CpG where only samples from 1 group have that CpG, so differential methylation cannot be determined. The red boxes represent a situation where the CpG is present in 1 subspecies, but spontaneous deamination has mutated the CpG site into a TpG site in the other subspecies. In this case, differential methylation can be calculated. However, it will be erroneous as only 1 group has a true CpG at that site. (F) Graphical representation of how breed differences were determined. We compared methylation and gene expression between BTBT and BIBI samples. (G) Graphical representation of how we determined parent-of-origin effects (POEs). Maternal POEs were determined by comparing BTBT and BIBT against BIBI and BTBI. Paternal POEs were determined by comparing BTBT and BTBI against BIBI and BIBT.

Each of the 24 samples representing the 4 genetic groups (Fig. 1A; Supplementary Table S1) was sequenced for WGBS analysis to at least 30× coverage and then mapped separately to the Brahman and Angus genomes (Fig. 1B). An average mapping rate of ∼95% was achieved when reads were mapped from each sample to the Brahman reference genome and ∼93% when mapping to the Angus reference genome (Table 1; Supplementary Table S1). All samples had at least 10× coverage for 93% of the Brahman sequence. Using the Angus reference, all samples had 10× coverage for at least 90% of the sequence (Table 1; Supplementary Table S1).

**Table 1:** Mapping statistics of Angus and Brahman reference genomes

	Angus	Brahman
Mapped reads^a^	1,455,481,398 (99%)	1,457,794,807 (99%)
Duplication rate (%)^a^	10	14
CpGs with ≥10× coverage in all samples	22,116,287 (86%)	21,962,589 (85%)
CpG coverage^a^	30	30

a Mean of all samples.

We performed all analyses twice for each reference genome, first using all CpGs with ≥10× in each reference genome and again where we retained only CpG sites with ≥10× that we could confidently assign as being shared between both breeds. Between 85% and 88% of autosomal CpG sites had coverage ≥10× when considering all CpG sites on both the Brahman and Angus reference and shared CpG sites (Table 1; Supplementary Table S2). Median coverage of CpG sites across all samples ranged from 25× to 34× regardless of reference and CpG sites considered (i.e., shared or all) (Table 1; Supplementary Table S3).

### Clustering of genetic groups

Comparing the methylation patterns between the genetic groups, we found that samples within a genetic group were more similar to each other than with samples from other groups. For example, samples from the BTBT group had higher correlations with other BTBT samples than BIBI samples. BTBT had the highest within-group Pearson correlations (*r* between 0.81 and 0.88) (Supplementary Fig. S1). The samples that were least correlated with one another were those belonging to BTBT and BIBI, with correlations between 0.75 and 0.78. Samples from the reciprocal cross-groups (BIBT; BTBI) had similar correlations with other samples within their own group (*r* between 0.81 and 0.83), as well as with samples from the alternative reciprocal cross (*r* between 0.80 and 0.83). Overall, correlations were high within each genetic group (*r* ≥ 0.8) (Supplementary Fig. S1).

**Figure 2:**
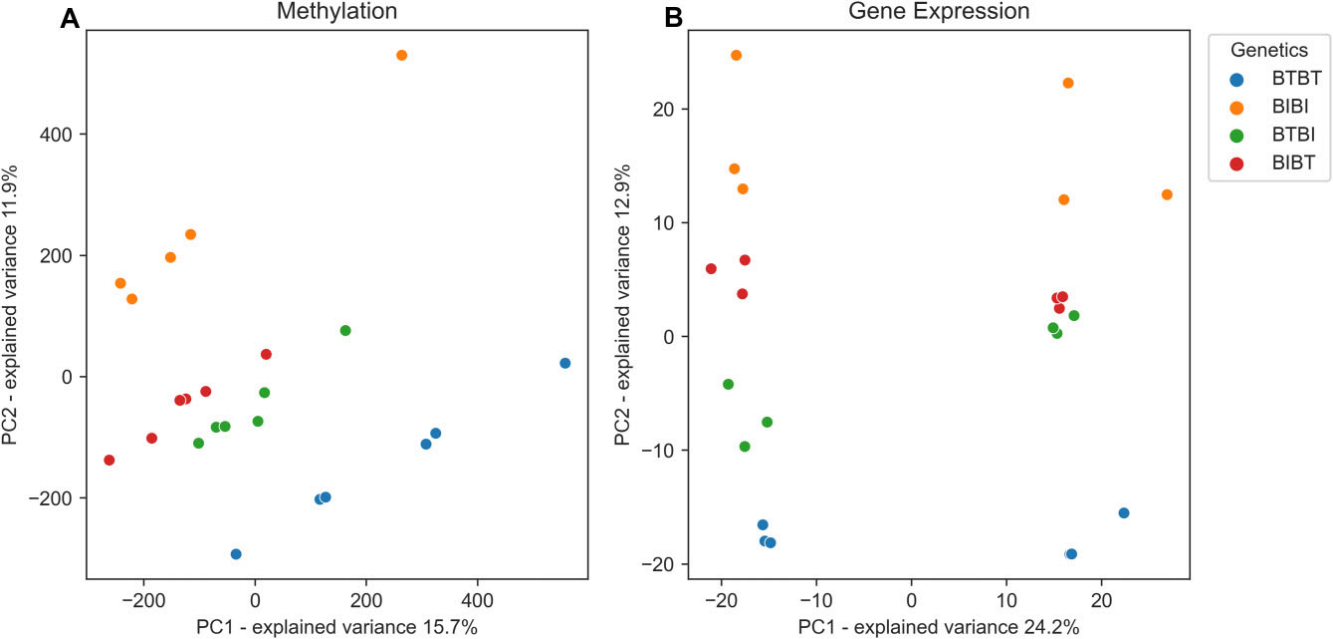
(A) PCA plot showing separation of genetic groups by methylation. Blue represents BTBT, orange represents BIBI, green represents BTBI, and red represents BIBT. The x-axis is principal component 1, and the y-axis is principal component 2. (B) PCA plot showing separation of genetic groups by gene expression data; colors are same as A. The x-axis is the first dimension of the logCPM, and the y-axis is the second dimension of the logCPM.

We performed a principal component analysis (PCA) of the 24 samples using CpG sites covered by at least 10 reads in all samples (Fig. 2A). BTBT and BIBI formed distinct clusters distant from one another, with the 2 hybrid genetic groups clustering much closer together and between the 2 parental genetic groups. Nevertheless, the hybrid groups were clearly separated on the PCA plot (Fig. 2A). The separation of groups we observed from the methylation data was similar to that seen for the gene expression data (Fig. 2B), indicating a potential parent-of-origin effect.

### Overview of DNA methylation patterns

Samples had a global mean CpG methylation of between 47% and 62%, with most samples ranging from 49% to 54% (Supplementary Table S4). Mean exon CpG methylation was 45% to 54% for all samples, with most samples ranging from 48% to 54% methylation (Supplementary Table S5). The 5′UTRs and promoter regions had the lowest mean CpG methylation percentage across all samples, between 10%–13% and 24%–31%, respectively (Supplementary Table S5). The intergenic regions displayed mean methylation levels that ranged from 47% to 65% (Supplementary Table S5), with most samples ranging from 49% to 57%, similar to the global mean. The introns exhibited slightly higher methylation levels, with means ranging from 50% to 64% (Supplementary Table S5) and most samples in the 53% to 59% range. The 3′UTRs revealed the highest overall CpG methylation levels, 57% to 69% (Supplementary Table S5). Lastly, the predicted enhancers, according to MacPhillamy et al. [58], exhibited CpG methylation levels ranging from 42% to 53%, with most samples within 43% to 48% methylated (Supplementary Table S5). Similar methylation patterns were observed using only shared CpGs in exons, 5′UTRs, intergenic regions, introns, promoters, predicted enhancers, and 3′UTRs, regardless of the reference genome used.

### Shared and breed-specific CpGs

We were able to confidently identify 74% to 75% of CpGs in the Brahman and Angus genomes that were shared between the 2 breeds (Table 2; Supplementary Table S6). We found that around 4% of CpG alignments contained an SNP between reference genomes (Supplementary Table S6; Supplementary Fig. S2) (i.e., were breed-specific). About 22% of CpGs could not be confidently assigned as shared or breed-specific and so were not considered in the shared CpG analysis. By definition, breed-specific regions with CpG sites did not align when the other breed genome was used as the reference. We found ∼1% of such CpG sites. In total, the SNP change and breed-specific categories of CpG sites constituted 4.7% and 4.9% of CpGs between the Angus and Brahman reference genomes, respectively, and were considered breed-specific. It should be noted that in this study, we consider breed-specific CpGs that appear in one reference genome and not the other. The limitation of this is that neither reference genome likely captures all variation present within the 2 breeds.

**Table 2:** Number of CpGs in the Angus and Brahman reference genomes

	Angus	Brahman
Total CpGs^a^	25,712,300	25,799,151
CpGs aligned to other reference^b^	25,209,966	25,228,509
CpGs shared in other genome^c^	18,813,726	18,781,688
CpGs affected by SNP	993,318	1,003,167
Unresolved CpGs^d^	5,402,922	5,443,654

a Total number of CpGs present within the genome.

b Number of CpGs that could be aligned from one genome to the other using Minimap2 [59].

c Number of CpGs in footnote b (i.e., the previous row) that were CpGs in both species.

d Number of CpGs in footnote b that could not be confidently assigned as either shared or an SNP.

### Enrichment of SNPs affecting CpG sites

Using the autosomal SNPs identified by Minimap2 and PAFtools.js [59], we observed that Brahman and Angus autosomal sequences differ by an average of ∼0.4% (Supplementary Table S7). SNPs in CpG sites were enriched by ∼13 times compared to the genomic background (chi-square test of independence, adjusted *P* < $5 \times {10}^{ - 324}$), which means SNPs between these 2 breeds affect CpG sites disproportionately more than other autosomal sites.

Looking more closely at the CpG SNP changes, we found that most (∼81%) of them were either C to T or G to A changes (Supplementary Fig. S2), which is very similar to the number of CpG SNP changes detected in humans (80.7%) [60]. The remaining SNP changes combined comprised ∼19% of the total observed mutations at CpG sites (Supplementary Fig. S2; Supplementary Table S6).

### Increased number of CpGs within structural variants

Next, we tested whether CpGs were enriched in SVs compared to the rest of the genome. Using the Brahman genome as the reference, we observed 16,011 SVs between Brahman and Angus, making up ∼15 Mb of sequence. We observed 1.18-fold more CpGs within SVs than in non-SVs (Mann–Whitney *U*-test, *P* = $8.91 \times {10}^{ - 5}$). When considering CpGs affected or introduced by SNPs and SVs, the CpG mutation rate is approximately 6.7% between Brahman and Angus compared to the genome-wide mutation rate of around 1%.

### Choice of reference genome influences methylome results

**Figure 3:**
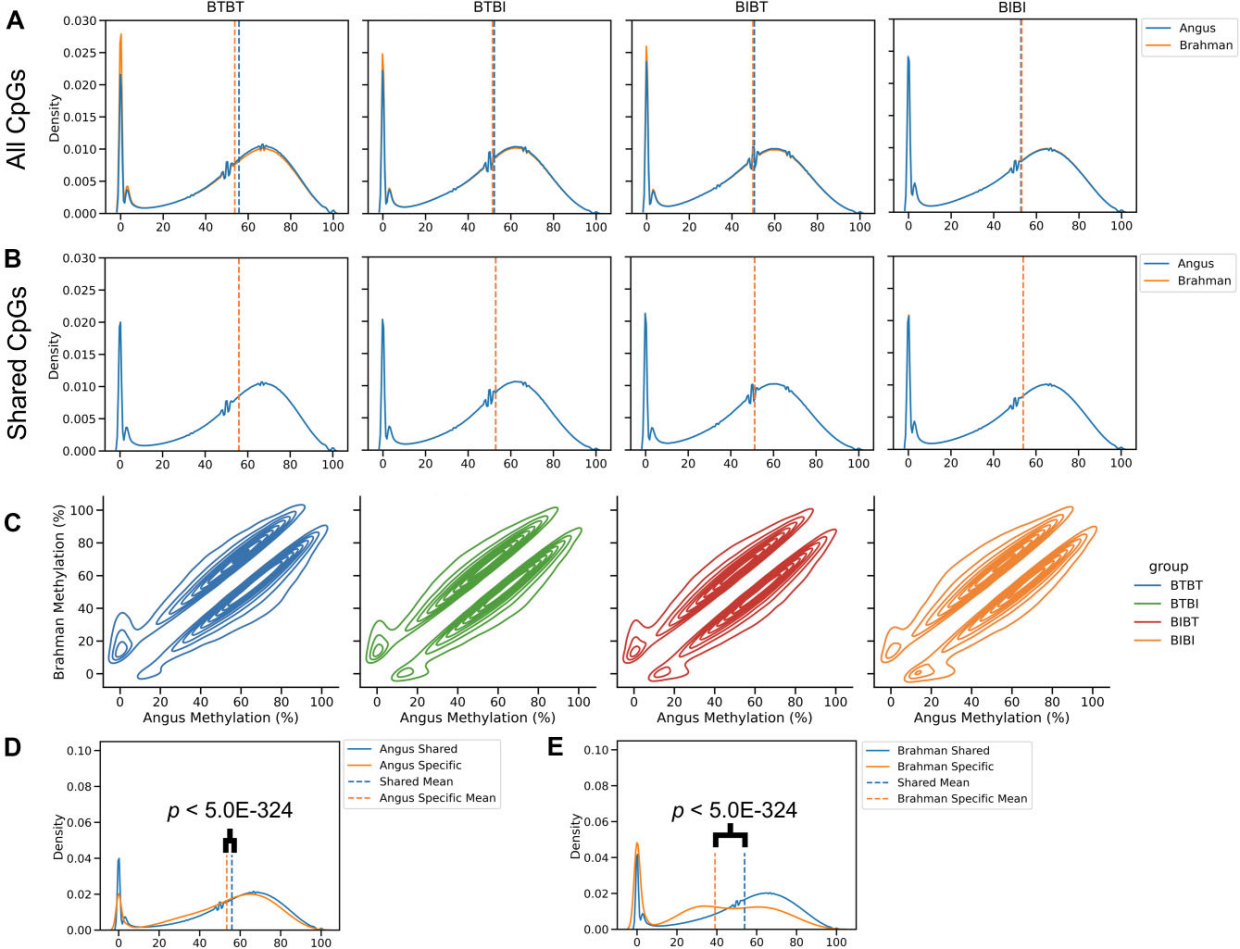
(A) Kernel density estimate (KDE) plots showing global CpG methylation using all CpG sites for all samples in the 4 genetic groups. Each panel represents a genetic group in the order BTBT, BTBI, BIBT, and BIBI. The x-axis represents the methylation percentage for a given CpG site. The y-axis represents the density. Each reference genome is represented by a different color, blue for Angus and orange for Brahman. (B) Same as A; however, only the shared CpGs were considered. Associated *P*-values for (A) and (B) can be found in Table 3. (C) KDE plot showing the methylation values of matched CpGs when mapped to the Angus reference (x-axis) and Brahman reference (y-axis) for all chromosomes. Only CpGs that differed by >10 are plotted (0.18% of all CpGs from all samples). (D) KDE plot illustrating the difference in methylation distribution between the shared and Angus-specific CpG sites when aligning Angus samples to the Angus reference. Blue represents shared CpG sites. Orange represents Angus-specific CpG sites. *P*-values were determined with a Wilcoxon signed-rank test. The x-axis denotes the methylation percentage. The y-axis represents the probability density. (E) KDE plot illustrating the difference in methylation distribution between the shared and Brahman-specific CpG sites when aligning Brahman samples to the Brahman reference. Blue represents shared CpG sites. Orange represents Brahman-specific CpG sites. *P*-values were determined with a Wilcoxon signed-rank test. The x- and y-axes are the same as (D).

We examined the CpG methylation differences between each sample when mapped to Angus and Brahman reference genomes and observed a statistically significant difference in CpG methylation levels in all samples when using all CpGs with ≥10× coverage (2-sample Kolmogorov–Smirnov test, adjusted *P* < 0.05) (Table 3; Supplementary Table S8). When only considering the shared CpGs, we observed a significant quantification bias in all samples, although it was much smaller (2-sample Kolmogorov–Smirnov test, adjusted *P* < 0.05) (Table 3; Supplementary Table S8). When we pooled all CpG methylation values for a given group and compared the Brahman and Angus references, we observed a statistically significant quantification bias in all 4 groups (2-sample Kolmogorov–Smirnov test, adjusted *P* < 0.05) (Fig. 3A; Table 3; Supplementary Fig. S3A; Supplementary Table S8). When comparing only the shared CpGs, we observed a much weaker quantification bias between genomes, although it was still significant at the group level (2-sample Kolmogorov–Smirnov test, adjusted *P* < 0.05) (Fig. 3B; Table 3; Supplementary Fig. S3B; Supplementary Table S8). When comparing global CpG methylation differences between samples mapped to Brahman and those mapped to Angus, the largest quantification bias was ∼2% for BTBT. The other quantification biases were ∼0.8%, ∼0.7%, and ∼0.3% for BTBI, BIBT, and BIBI samples, respectively (Fig. 3A; Table 3; Supplementary Fig. S3A; Supplementary Table S8). When using only shared CpGs, the quantification bias was reduced to ∼0.6%, ∼0.5%, ∼0.4%, and ∼0.2% in BTBT, BTBI, BIBT, and BIBI, respectively (Fig. 3B; Table 3; Supplementary Fig. S3B; Supplementary Table S8).

**Table 3:** Sample-wise and group-wise methylation quantification biases

Sample-wise				Group-wise			
ID	Difference^a^	*P*-value	Adj. *P*-value	ID	Difference^a^	*P*-value	Adj. *P*-value
All CpGs^b^				All CpGs^c^			
F103	1.99	5.00E-324	5.00E-324	BTBT	1.98	5.00E-324	5.00E-324
F105	2.16	5.00E-324	5.00E-324				
F52	2.25	5.00E-324	5.00E-324				
F53	1.88	5.00E-324	5.00E-324				
F60	1.73	5.00E-324	5.00E-324				
F7	1.87	5.00E-324	5.00E-324				
F100	1.15	5.00E-324	5.00E-324	BTBI	0.79	5.00E-324	5.00E-324
F104	0.83	5.00E-324	5.00E-324				
F106	0.85	5.00E-324	5.00E-324				
F61	1.06	5.00E-324	5.00E-324				
F74	0.86	5.00E-324	5.00E-324				
F97	0.01	3.69E-02	3.69E-02				
F13	0.66	5.00E-324	5.00E-324	BIBT	0.69	5.00E-324	5.00E-324
F62	0.63	5.00E-324	5.00E-324				
F77	0.73	5.00E-324	5.00E-324				
F80	0.71	5.00E-324	5.00E-324				
F8	0.75	5.00E-324	5.00E-324				
F91	0.65	5.00E-324	5.00E-324				
F22	0.34	5.00E-324	5.00E-324	BIBI	0.34	5.00E-324	5.00E-324
F46	0.34	8.02E-301	8.37E-301				
F56	0.29	5.81E-301	6.34E-301				
F65	0.40	5.00E-324	5.00E-324				
F78	0.36	5.00E-324	5.00E-324				
F99	0.34	5.00E-324	5.00E-324				
Shared^d^				Shared^e^			
F103	0.06	1.24E-09	7.41E-09	BTBT	0.05	1.65E-40	6.61E-40
F105	0.06	3.78E-13	9.06E-12				
F52	0.06	2.12E-12	2.55E-11				
F53	0.05	1.36E-08	6.55E-08				
F60	0.04	4.05E-08	1.62E-07				
F7	0.05	6.76E-10	5.41E-09				
F100	0.04	5.22E-06	1.79E-05	BTBI	0.04	1.52E-18	3.04E-18
F104	0.03	2.15E-04	4.29E-04				
F106	0.04	3.88E-04	7.16E-04				
F61	0.04	6.40E-06	1.92E-05				
F74	0.04	2.93E-05	7.82E-05				
F97	0.03	3.89E-02	5.19E-02				
F13	0.03	1.83E-03	2.92E-03	BIBT	0.03	1.85E-16	2.47E-16
F62	0.03	6.50E-03	9.18E-03				
F77	0.03	1.07E-03	1.83E-03				
F80	0.04	3.82E-05	9.17E-05				
F8	0.04	1.34E-04	2.92E-04				
F91	0.03	3.82E-03	5.73E-03				
F22	0.02	1.09E-01	1.14E-01	BIBI	0.02	2.94E-16	2.94E-16
F46	0.03	5.05E-02	6.38E-02				
F56	0.02	7.49E-02	8.99E-02				
F65	0.02	1.65E-01	1.65E-01				
F78	0.02	8.18E-02	9.35E-02				
F99	0.02	1.03E-01	1.13E-01				

a Refers to the absolute percentage difference of the mean CpG methylation when mapped to Angus compared to Brahman for a given sample.

b Denotes sample-wise values calculated using all (21,432,071) CpG sites. Refers to Fig. 3A.

c Denotes group-wise values calculated using all CpG sites in all samples within a group (i.e., pooled) (21,432,071 × 6 = 128,592,426). Refers to Fig. 3A.

d Denotes sample-wise values using only shared (16,204,834) CpG sites. Refers to Fig. 3B.

e Denotes group-wise values calculated using only shared CpG sites in all samples within a group (i.e., pooled) (16,204,834 × 6 = 97,229,004) CpG sites. Refers to Fig. 3B.

Next, we examined whether the shared CpGs exhibited correlated methylation levels regardless of the reference genome used. For example, we wanted to determine whether a given CpG in the Brahman reference has the same methylation percentage as the corresponding CpG in the Angus reference for a given sample. We observed root mean square error (RMSE) values of around 0.01 for groups (Supplementary Fig. S4), with most CpGs exhibiting less than 10% difference in methylation between the reference genomes. Interestingly, we observed several CpGs that appeared sensitive to reference genome choice. For example, 264,023 CpGs had an absolute methylation difference of least 10%, with 429 CpGs having an absolute difference of at least 50% depending on the reference genome used (Supplementary Table S9).

To further investigate the influence of the reference genome on downstream analyses, we compared differentially methylated regions (DMRs) identified by the 2 reference genomes to evaluate if the direction of methylation changed (i.e., hypermethylated became hypomethylated and vice versa). DMRs from Angus were mapped to the Brahman reference, and we found approximately 12% (28,922) of Angus DMRs overlapped with Brahman DMRs by at least 90% of their length. Of these DMRs that mapped to the Angus reference, 3,575 showed changes in methylation direction when mapped to the Brahman reference (Supplementary Table S10). That is, a DMR that was observed as hypomethylated in Angus samples relative to Brahman when mapped to the Angus reference was observed to be hypermethylated in Angus relative to Brahman when mapped to the Brahman reference. We observed similar numbers (3,581) when lifting DMRs from Brahman to Angus (Supplementary Table S10). There were no methylation direction changes when we considered differentially methylated cytosines (DMCs).

Lastly, we compared homologous regions between the Angus and Brahman reference genomes to determine whether any differentially methylated regions existed between these 2 genomes for each group (Methods). Using a window size of 100 kb, we observed 41, 31, 30, and 36 windows being significantly differentially methylated when using BTBT, BTBI, BIBT, and BIBI samples, respectively (paired Wilcoxon test, adjusted *P* < 0.05) (Supplementary Table S11).

### Breed-specific CpGs show distinct methylation patterns

Looking more closely at the breed-specific CpGs, we first determined the background methylation for Angus samples as the methylation of all shared CpG sites when mapped to the Angus reference. We then extracted the methylation for all Angus samples from CpG sites that were identified as breed-specific. We then compared the background methylation distribution against the Angus-specific distribution. This was repeated for the Brahman using the Brahman reference. Breed-specific CpG sites were significantly more likely to have lower methylation than the background methylation for Angus (Wilcoxon signed rank test, *P* < $5.0\ \times {10}^{ - 324}$) and Brahman (Wilcoxon signed rank test, *P* < $\ 5.0 \times {10}^{ - 324}$), respectively (Fig. D, E).

### DMRs between breeds show limited overlap with differentially expressed gene promoters

As we observed a quantification bias when using all CpGs mapped against each reference genome, we restricted breed-specific and POE analyses to those CpGs identified as shared. Additionally, we examined the number of DMRs at the 25% and 50% difference thresholds, that is, more stringent thresholds for calling DMRs, which substantially reduced the numbers (Supplementary Table S12). Given that minor changes of less than 10% to 15% in methylation have been observed to influence gene expression and phenotype [61, 62], we used a difference threshold of 10% to interpret the results.

Using Brahman as the reference, we identified 123,602 DMRs and 1,549 differentially expressed genes (DEGs) (Supplementary Tables S12 and S13). Of the 123,602 DMRs observed, ∼19% (23,575) overlapped with the surrounding region of the significant DEGs, with around twice as many DMRs falling within the putative-enhancer region compared to the promoter region after normalizing for the length difference between the enhancer regions and promoter regions (Supplementary Table S14). Only 68 DMRs overlapped with promoters of DEGs, despite 99% of significant DEGs being overlapped by a DMR. When the Angus reference was used, of the 125,544 DMRs identified, ∼25% (31,252) of those overlapped with a DEG. Around 1.6 times as many DMRs fell into an enhancer region compared to DEG promoter regions (Supplementary Table S14), despite substantially more (1,872) DEGs observed (Supplementary Table S13) and 99% overlapping with a DMR.

We then examined the overlap of DMRs and imprinted genes, first using Brahman as the reference. Here, ∼1% (1,182) of the DMRs identified between BIBI and BTBT overlapped 79 imprinted genes. Only 1 imprinted gene, Par-6 family cell polarity regulator gamma (*PARD6G*), did not overlap with any DMR. Most DMRs (∼79% of the 1,182) that overlapped an imprinted gene fell into putative enhancer regions. Five imprinted genes were significantly differentially expressed when comparing BIBI and BTBT (Table 4). These genes were DS cell adhesion molecule (*DSCAM*), neuronatin (*NNAT*), Lin-28 homolog B (*LIN2B*), and protein phosphate 1 regulatory subunit 9A (*PPP1R9A*). *DSCAM* and *NNAT* had higher expression in BTBT, and the remaining 3 DEGs, diacylglycerol O-acyltransferase 1 (*DGAT1*), *LIN2B*, and *PPP1R9A*, had higher expression in BIBI. We again observed 5 imprinted DEGs using the Angus reference; however, 1 gene was a novel gene. The remaining genes (*NNAT, LIN28B, DGAT1*, and *PP1R9A*) showed the same expression pattern as when mapped to Brahman (Table 5).

**Table 4:** Significant imprinted DEGs and their overlap with DMRs when using the Brahman reference genome

Gene ID	Gene name	Protein name	Increased expression in Brahman^a^	Number of hypo-DMRs in Brahman	Number of hyper-DMRs in Brahman
Breed comparison					
ENSBIXG00005007073	*DSCAM*	DS cell adhesion molecule	No	61	18
ENSBIXG00005012203	*NNAT*	Neuronatin	No	37	1
ENSBIXG00005029958	*LIN28B*	Lin-28 homolog B	Yes	8	1
ENSBIXG00005009822	*DGAT1*	Diacylglycerol O-acyltransferase 1	Yes	3	2
ENSBIXG00005007141	*PPP1R9A*	Protein phosphatase 1 regulatory subunit 9A	Yes	11	5
Dam of origin comparison					
ENSBIXG00005019306	*ZC3H12C*	Zinc finger CCCH-type containing 12C	Yes	5	1
ENSBIXG00005007141	*PPP1R9A*	Protein phosphatase 1 regulatory subunit 9A	Yes	3	2
ENSBIXG00005029958	*LIN28B*	Lin-28 homolog B	Yes	1	0
ENSBIXG00005015804	*RTL1*	Retrotransposon Gag like 1	No	3	0
Sire of origin comparison					
ENSBIXG00005007073	*DSCAM*	DS cell adhesion molecule	No	38	5
ENSBIXG00005021735	*HTR2A*	5-hydroxytryptamine receptor 2A	Yes	18	2
ENSBIXG00005012203	*NNAT*	Neuronatin	No	8	0
ENSBIXG00005009822	*DGAT1*	Diacylglycerol O-acyltransferase 1	Yes	0	1
ENSBIXG00005025714	*MKRN3*	Makorin ring finger protein 3	No	0	0
ENSBIXG00005025694	*NDN*	Necdin MAGE family member	No	0	1
ENSBIXG00005024991	*SLC22A18*	Solute-carrier family 22 member 18	Yes	0	0
ENSBIXG00005013434	*TFPI2*	Tissue factor pathway inhibitor 2	No	25	0

* Increased expression in Brahman denotes genes that were significantly more highly expressed in Brahman than in Angus. “No” denotes that gene was significantly more highly expressed in Angus.

**Table 5:** Significant imprinted DEGs and their overlap with DMRs when using the Angus reference genome

Gene ID	Gene name	Protein name	Increased expression in Angus^a^	Number of hypo-DMRs in Angus	Number of hyper-DMRs in Angus
Breed comparison					
ENSBIXG00000027129	Novel gene		Yes	20	66
ENSBIXG00000021864	*NNAT*	Neuronatin	Yes	2	32
ENSBIXG00000002586	*LIN28B*	Lin-28 homolog B	No	0	6
ENSBIXG00000012321	*DGAT1*	Diacylglycerol O-acyltransferase 1	No	3	3
ENSBIXG00000005197	*PPP1R9A*	Protein phosphatase 1 regulatory subunit 9A	No	9	15
Dam of origin comparison					
ENSBIXG00000011151	*ZC3H12C*	Zinc finger CCCH-type containing 12C	No	0	4
ENSBIXG00000005197	*PPP1R9A*	Protein phosphatase 1 regulatory subunit 9A	No	6	3
ENSBIXG00000002586	*LIN28B*	Lin-28 homolog B	No	0	1
Sire of origin comparison					
ENSBIXG00000027129	*DSCAM*	DS cell adhesion molecule	Yes	5	35
ENSBIXG00000008539	*HTR2A*	5-hydroxytryptamine receptor 2A	No	1	17
ENSBIXG00000021864	*NNAT*	Neuronatin	Yes	1	11
ENSBIXG00000012321	*DGAT1*	Diacylglycerol O-acyltransferase 1	No	0	0
ENSBIXG00000015087	*MKRN3*	Makorin ring finger protein 3	Yes	1	1
ENSBIXG00000015080	*NDN*	Necdin MAGE family member	Yes	1	0
ENSBIXG00000028529	*SLC22A18*	Solute-carrier family 22 member 18	No	0	2

a Increased expression in Angus denotes genes that were significantly more highly expressed in Angus than in Brahman. “No” denotes that gene was significantly more highly expressed in Brahman.

### Dam-of-origin methylation shows less overlap with imprinted genes

To investigate the dam-of-origin effects (DOEs), we compared samples with Brahman dams (BIBI and BTBI) with those with Angus dams (BTBT and BIBT). Using the Brahman genome as the reference, 457 DEGs were identified in the DOE comparison. Around 6% (1,236) of the DMRs overlapped with DEGs, with ∼78% of DEGs being overlapped by a DMR. Approximately 2.4 times as many DMRs fell into putative enhancer regions compared to DEG promoter regions (Supplementary Table S14). There were 52 imprinted genes that overlapped with ∼1% (190) of the DMRs identified in the comparison. Of the 190 DMRs that overlapped with an imprinted gene, 128 DMRs overlapped with the putative enhancer region. Four imprinted genes were significantly differentially expressed (Table 4). Zinc finger CCCH-type containing 12C (*ZC3H12C*), *PPP1R9A*, and *LIN28B* had higher expression in samples with Brahman mothers. Using Angus as the reference, we observed 1,254 (∼5%) DMRs overlap with the 358 DEGs and ∼88% of DEGs covered by a DMR. Around 2.30-fold more DMRs fell into putative enhancer regions compared to DEG promoter regions (Supplementary Table S14). Three imprinted DEGs (*ZC3H12C, PPP1R9A*, and *LIN28B*) were identified using the Angus genome as the reference (Table 5).

### Sire-of-origin methylation may be driving differential gene expression

To investigate the sire-of-origin effects (SOEs), we compared samples with Brahman sires (BIBI and BIBT) with those with Angus sires (BTBT and BTBI). Using the Brahman reference, we identified 62,056 DMRs and 1,190 DEGs in the sire group comparison using shared CpGs (Supplementary Tables S12 and S13), with 2.4 times more DMRs overlapping a putative enhancer region than promoter region (Supplementary Table S14)). There were 63,516 DMRs identified using the Angus reference, but substantially more DEGs (1,568) were identified (Supplementary Table S13). Around 12% (7,840) of the DMRs overlapped with a significant DEG; ∼93% of DEGs were overlapped by a DMR. Around 19% (∼12,251) of DMRs overlapped a DEG and ∼96% of DEGs overlapped with a DMR; ∼75% of the DMRs overlapped a putative enhancer region. This was approximately twice as many DMRs overlapped with a putative enhancer compared to a promoter when using the Angus reference.

We observed 73 imprinted genes that overlapped DMRs identified between the 2 different sire groups. Less than 1% (586) of DMRs overlapped the 73 imprinted genes, with most (∼73% of 586) occurring in the putative enhancer region. Eight imprinted genes were significantly differentially expressed and overlapped with a DMR (Table 4). These genes were *DSCAM*, 5-hydroxytryptamine receptor 2A (*HTR2A*), *NNAT, DGAT1*, necdin MAGE family member (*NDN*), and tissue factor pathway inhibitor 2 (*TFPI2*). *DSCAM, NNAT, NDN* (Makorin ring finger protein 3), *MKRN3*, and *TFPI2* had higher expression in samples with Angus sires, with the other genes (*HTR2A, DGAT1*, solute-carrier family 22 member 18 [*SLC22A18*]) being more highly expressed in samples with Brahman sires. *SLC22A18* showed high expression in the Brahman sire group, and *MKRN3* showed higher expression in the Angus sire group, but neither overlapped with any DMRs. Six significantly differentially expressed imprinted genes were observed when using the Angus reference (Table 5). In this case, the 6 significantly differentially expressed imprinted genes with DMR overlap were *DSCAM, HTR2A, NNAT, MKRN3, NDN*, and *SLC22A18. DSCAM, NNAT, MKRN3*, and *NDN* had higher expression in samples with an Angus sire. The remaining 3 genes (*HTR2A, DGAT1*, and *SLC22A18*) had higher expression in samples with a Brahman sire. *DGAT1* did not overlap any DMRs when using the Angus reference.

## Discussion

In the present study, we observed genome-wide CpG methylation correlations among replicates that ranged from 75% to 82% between groups and from 81% to 87% within groups. These correlations were similar to a recent study in mice where genome-wide CpG methylation correlations among replicates ranged from 73% to greater than 80% [63]. Moreover, we observed levels of liver global CpG methylation between 47% and 62% in the present study, which is similar to previous studies of human [64], mouse [63], and cattle [65].

Mapping statistics can provide an insight into how the choice of reference genome will affect downstream analyses [66]. However, we observed negligible differences in raw mapping statistics regardless of whether the Angus or Brahman reference genomes were used. Additionally, the global methylation quantification bias observed was less than 2%, depending on the reference genome used. This quantification bias is lower than the 7% to 9% quantification bias found in the mouse genome, depending on the reference genome used [15]. The extent of this bias is influenced by the divergence between reference genomes and whether the breed-specific CpGs tend to be hypo- or hypermethylated. Brahman and Angus have a CpG divergence of ∼4%, whereas the mouse genomes analyzed by Wulfridge et al. [15] had a CpG divergence of 10.7%. The bias we observed was greatest in the BTBT samples when the Brahman genome was used as the reference, most likely because the Angus-specific CpG sites tended to be hypermethylated. Conversely, the quantification bias was lower in the other genetic groups, possibly due to the hypomethylation in Brahman-specific CpG sites.

Spontaneous deamination of methylated CpG to TpG is the most common dinucleotide mutation in the mammalian genome [40, 41]. We observed around 81% of SNPs between Brahman and Angus as being C-T or G-A mutations. A recent study observed 34,677 SNPs affecting CpG sites between indicine and taurine genomes [67]. The difference in the number of SNPs between the 2 studies is likely due to Capra et al. [67] having used reduced representation bisulfite sequencing with substantially lower coverage than the present study and that they only considered SNPs that affected CpG sites. When differential methylation analysis is performed, a breed that has lost the C (mutated to T) will be reported as having 0% methylation at that site when, in fact, there is no CpG present. This incorrect identification of an unmethylated site can then severely impact the interpretation of results.

SVs have been associated with various traits in humans, including HIV-1 susceptibility [68], autism [69–71], and carcinogen metabolism [72]. In livestock, SVs have been implicated in diverse traits ranging from horn (polled) status [73, 74] to bulldog calf syndrome [75]. The SVs between Brahman and Angus have significantly more CpGs than the background genome, potentially introducing CpGs with important regulatory effects. However, due to their presence in only 1 subspecies, a single reference genome will fail to account for these breed-specific CpG sites. Therefore, the phenotypic differences between the 2 breeds may be influenced by CpGs that cannot be compared accurately with a single reference genome if they are in breed-specific regions.

We observed relatively few changes in methylation direction, and these were likely to be an artifact of how the genome was tiled and possible erroneous alignments in the coordinate conversion. A common step of some DMR callers is to perform window tiling of the genome to identify DMRs or to enable analysis when coverage is low [76–78]. SNPs and SVs can potentially complicate analyses when genome tiling is used to identify DMRs, as a single reference genome cannot account for these variants. Although we observed no directional changes when considering DMCs, it is possible that SNPs impacting CpGs in otherwise shared regions affected the quantification of DMRs. For example, MethylKit uses a genome tiling approach to identify DMRs, and so any mutation that impacts a CpG site, particularly spontaneous deamination (C > T), is likely to erroneously identify a region as hypomethylated in the group that has the spontaneous deamination when, in fact, no CpG exists in that group. As such, researchers should be careful when using genome tiling in methylation analyses that compare breeds, strains, or populations. Moreover, we demonstrated several CpG sites that appear sensitive to reference genome choice, differing by more than 10% between the 2 genomes. Additionally, we demonstrated that multiple 100-kb regions are differentially methylated between the genomes when using a single group of samples. It is likely that these CpG sites and variable regions would confound downstream analysis, especially if they overlap with regions of interest, such as *cis*-regulatory elements. Thus, this demonstrates that quantification bias can remain even when attempting to control for reference genome differences.

While DNA methylation analyses between breeds and strains are challenging, exciting solutions are on the horizon. Indeed, with long-read sequencing like Oxford Nanopore becoming more cost-effective and given it has the ability to capture DNA methylation with no additional sample preparation, it is much more likely that researchers will be able to follow the recommendations of Wulfridge et al. [15] and examine DNA methylation of individuals with personalized reference genomes. Long-read sequencing has the potential to simplify DNA methylation analyses of diverse populations substantially.

Most DMRs identified in this study did not overlap with DEGs. However, of the DEGs that overlapped with a DMR, there was a tendency for the overlap to occur more frequently in the putative enhancer region than in promoters or DEG bodies. This trend suggests that differential methylation of enhancers may impact gene expression differences in bovine fetal liver. Indeed, a growing body of evidence suggests that enhancer methylation is important in embryonic and fetal development [63, 79–81].

There were more significant DEGs when mapping to the Angus reference than the Brahman reference. Interestingly, genes that were differentially expressed (DE) using the Brahman reference were not always DE when using the Angus reference. The choice of reference genome has been shown to impact differential expression analysis in rice [82], bacteria [83], and human [84, 85] when using short-read RNA sequencing (RNA-seq). When the reference genome better represents the individuals being studied, more reads can be uniquely aligned to the correct position, providing a more accurate estimate of gene expression.

We identified several interesting DEGs associated with DMRs, particularly imprinted genes. Among these was *DGAT1*, which is involved in fat metabolism in milk production [86], feed conversion, and adipogenesis [86, 87]. Several studies have investigated the role of *DGAT1* in weight gain [88, 89]; expression of *DGAT1* is necessary for weight gain, especially when the caloric density of food is high [89]. We found differential expression of *DGAT1*, with higher expression in Brahman than Angus (BIBI vs. BTBT) and when Brahman was the sire (BIBI, BIBT vs. BTBT, BTBI). Taken together, the comparison of the breed and sire of origin suggests that the breed of the sire may be an important determinant in the expression of this gene. Higher *DGAT1* expression may result from adaptation to poor feed quality; for example, Elzo et al. [90] observed better feed conversion efficiency in Brahman compared to Angus and Brahman × Angus cattle. Regulation of *DGAT1* expression may occur via DNA methylation, as there is a DMR ∼42 kb downstream of the transcription start site, which was identified in both the breed-specific and SOE comparisons.

Parent-of-origin DMRs may change how *cis*-regulatory elements interact with target genes and influence gene expression in the offspring [45, 91]. It has been observed that parent-specific methylation can alter the *cis*-regulatory landscape around certain genes, such as *IGF*2 [92, 93]. DMRs may influence the DEGs and, ultimately, help drive the differences in phenotype. However, to confidently assign gene expression and DMRs to a particular parent, long-read sequencing [94] is needed to identify variations that link the sequences to the parent of origin. Additionally, the use of reciprocal crosses will enable one to investigate if a combination of breed and sex of the parent impacts which transcript is expressed.

SNPs and SVs have been shown to complicate and bias analyses in several studies [15, 82–85]. In our analysis, we observed an enrichment of SNPs affecting CpGs between Brahman and Angus. Capra et al. [67] also reported a higher frequency of breed-specific SNPs around DMCs in a study of indicine and taurine cattle. This finding suggests that genetic differences between the 2 breeds may contribute to epigenetic variations. Using individual animal genomes in the study to account for genetic variations, as Wulfridge et al. [15] suggested, would enhance the accuracy for each individual. However, despite decreasing sequencing costs, the cost will likely be prohibitive in most livestock contexts. A possible solution was explored in a recent study comparing methylation in taurine and indicine cattle [67]. Here, the authors used genotyping by sequencing to exclude SNPs affecting CpG sites from the analysis [67]. While this simplifies downstream analysis, it may also remove CpGs involved in the phenotypic differences between the 2 breeds, representing a limitation of the present study and that of Capra et al. [67]. An alternative approach to using single reference genomes is the utilisation of pangenomes, which encompass the majority of variations within the population [20, 95]. This feature is particularly important in the context of DNA methylation studies where, demonstrated in our study, SNPs at CpG sites can exert substantial effects on local methylation information.

## Conclusions

This study generated a substantial WGBS dataset derived from 2 phenotypically diverse cattle breeds that are representative of the 2 cattle subspecies and highlighted the importance of reference genome choice in methylation analyses. Our findings suggest that the DMRs may primarily exert their influence on enhancer elements rather than promoters. We also identified 11 genes that might be under DMR control. The results underscore the advantages of using the appropriate reference genome for the dataset and provide additional evidence supporting the incorporation of genome graphs to improve analyses of populations with high genetic divergence.

## Methods

### Study animals and sample collection

All animal experiments and procedures described in this study complied with Australian guidelines, were approved by the University of Adelaide Animal Ethics Committee, and followed the ARRIVE (Animal Research: Reporting of In Vivo Experiments) Guidelines [96] (Approval No. S-094-2005). Liver tissue samples from concepti were the same as those described in Liu et al. [97]. Briefly, the parents were purebred Angus (*B. t. taurus*) and purebred Brahman (*B. t. indicus*), herein denoted as BT and BI. Fetuses were sired by 3 BTBT bulls and 2 BIBI bulls. Primiparous females and their fetuses were ethically sacrificed at day 153 ± 1 of gestation. Concepti were dissected and tissue samples snap-frozen in liquid nitrogen and stored at −80°C until further use. Liver samples from 3 female and 3 male individuals from each of the 4 genetic combinations (BT × BT, BT × BI, BI × BT, and BI × BI) were used.

### DNA extraction and sequencing

DNA was extracted from frozen fetal liver tissues using the Qiagen® Dneasy® Blood & Tissue Kit following the manufacturer’s instructions and sent to BGI for WGBS library preparation and sequencing. Bisulfite conversion was performed using the Zymo Research™ (RRID:SCR_008968) EZ DNA Methylation™—Gold Kit (D5005). All samples were sequenced in a single batch, and each sample was sequenced to ∼30× coverage using the BGI DNB-seq.

RNA was extracted from frozen fetal liver tissues using Illumina® (RRID:SCR_010233) RiboZero Gold kits following the manufacturer’s instructions and prepared for Illumina RNA-seq short-read sequencing. The RNA-seq protocol and data availability (GEO accession number: GSE148909) have been described in our previous work [97].

The same tissue samples were used in both the RNA-seq and WGBS. Individual sample names and their corresponding genetic group are given in Supplementary Table S11.

### WGBS mapping

WGBS reads were mapped using the MethylSeq Nextflow pipeline (v. 1.6.1) [98] with the “—zymo” trimming parameter. The reads were first checked for quality with FastQC (v. 0.11.9) (RRID:SCR_014583) [99], then adapters were trimmed using Trim Galore (v. 0.6.6) (RRID:SCR_011847) [100], and the reads were reassessed for quality posttrimming. Trimmed reads passing *q* ≥ 20 were mapped to the Brahman (GCA_003369695.2) and Angus (GCA_003369685.2) genomes [39] using BWA-Meth (0.2.2) (arXiv:1401.1129). The non-pseudo-autosomal region of the Angus Y chromosome was added to the Brahman reference. This step enabled us to include the Y chromosome sequence while avoiding duplication of the pseudoautosomal region. Both Brahman and Angus chromosome sequences were reorientated to match the orientation of ARS-UCD1.2 chromosomes [101]. After sorting the alignment files with SAMtools (v. 1.11) (RRID:SCR_002105) [102], duplicates were marked with Picard (v. 2.25.4) (RRID:SCR_006525) [103]. Bam file quality control was performed with the bamqc function from QualiMap (v. 2.2.2d) (RRID:SCR_001209) [104] by setting the “-gd” parameter to HUMAN. Methylation calls were extracted using MethylDackel (v. 0.5.2) [105] extract with the parameter “—minDepth 10” and output in MethylKit [76] format (“—methylKit”) and a more generic cytosine report (“—cytosine_report”). In-house scripts were used to convert the MethylDackel output for use with DNMTools [106] (see [107]). All samples had a bisulfite conversion efficiency of >99%. All downstream analyses only used CpGs from autosomes with ≥10× coverage.

### Identification of shared and breed-specific CpG sites

For a given autosome, we extracted 1,000 bp around all CpGs that were not in the first 500 bp or last 500 bp of the chromosome; this yielded sequences that were 1,002 bp long. We then mapped the 1,002-bp CpG sequences from one subspecies to the reference of the other. We used minimap2 (v. 2.24) [59] with the “map-hifi” preset to align CpGs from a given chromosome in one breed to the same chromosome in the other breed; alignments were sorted using SAMtools (v 1.11) [102]. Once the long sequences were aligned, we filtered the BAM file and considered all alignments where at least 900 bp were successfully aligned to the reference. We then used the Align package from BioPython (v 1.80) (RRID:SCR_007173) [108] to perform a local alignment between the 102-bp sequences taken from the midpoint of the query and reference. We then recorded which CpG sites were shared between Brahman and Angus, which CpG sites differed, and which could not be aligned during the initial minimap2 alignment step (Supplementary Table S6). We performed subsequent analyses using all CpGs present on the autosomes for each reference and again using only the shared CpGs that passed the ≥10× coverage criteria. The CpG sites that could not be aligned in the initial alignment step with minimap2 (i.e., a genomic region that is present in one breed but missing in the other breed) or constituted a SNP were considered breed-specific CpG sites. All steps described in this section were performed for both Brahman and Angus reference genomes.

### Identification of SNPs and SVs between genomes

To determine the accuracy of minimap2 alignments in identifying SNPs between the Brahman and Angus genomes, we first introduced artificial mutations into each genome using SNP Mutator [109]. The Angus and Brahman genomes have previously been reported to differ by ∼1% [39]. Therefore, to determine how well minimap2 alignments can be used to detect SNPs between sequences that are divergent by ∼1%, we first simulated mutations in each autosome for both species. For example, chromosome 1 in the Angus reference genome is 157,005,132 bp long, so we set the number of substitutions to 1,570,051 SNPs. In addition, the random seed was set to 12, and the number of times each autosome was mutated was set to 1. We repeated this for all autosomes in the Brahman and Angus genomes, adjusting the number of SNPs to maintain the 1% divergence in each autosome. We then mapped the mutated autosomal sequence to the original sequence for each autosome and breed, giving us 58 “replicates.” Alignments from minimap2 [59] were used as input to PAFtools, which was used to identify variants between the original and mutated sequences. The minimap2 mapping parameters used were “-x asm10, -c, –cs,” followed by PAFtools “call –f,” where the file provided to the –f argument was the original, unmutated autosomal sequence. Minimap2 and PAFtools showed a mean accuracy of ∼99% across the 58 autosomes, suggesting a good ability to identify SNPs between the 2 breeds (Supplementary Table S15). We then aligned each autosome from Brahman to each autosome from Angus, using minimap2 with the parameters “-cx asm10” and “—cs.” The output from minimap2 was then used as the input to the paftools.js call with the parameter “-f <reference_autosome.fa>,” where reference_autosome.fa refers to the autosome that was supplied first to minimap2 (i.e., the reference sequence, not the query). We then used the output VCF files to determine the SNP and SVs between the 2 genomes.

### SNP and SV enrichment

To determine whether CpGs were significantly impacted by SNPs, we identified all SNPs between Brahman and Angus that impacted a CpG site. As Brahman autosomes were used as the reference to minimap2, we used the coordinates of all CpGs within the Brahman genome to identify which SNPs in the VCF file had altered a CpG site. We constructed 2 × 2 contingency tables to perform a chi-square test of independence per chromosome to determine whether CpGs had a higher likelihood of being affected by a SNP than non-CpGs. The 4 categories were “CpG affected by SNP,” “non-CpG affected by SNP,” “unaffected CpG,” and “unaffected non-CpG.” We then adjusted the *P*-values to correct for multiple testing using the Benjamini–Hochberg correction procedure.

To assess whether CpGs were significantly enriched within SVs identified between the 2 genomes, we counted the number of CpG dinucleotides occurring within SV sequences and compared that against the number of CpGs that occurred in non-SV regions. To determine the probability of a CpG occurring outside an SV, we first identified the SV coordinates from the VCF file produced by PAFtools and constructed a bed file of SVs for each reference genome. Next, we used BEDTools [110] to generate coordinates of the complementary regions (i.e., the non-SV regions of each genome). We then extracted the fasta sequence of these regions for each genome. We then counted the number of CpGs that occurred in these regions on each chromosome and divided them by the combined length of all SVs on the given chromosome to determine the proportion of CpGs occurring in the SV regions; this value was added to a vector. We repeated this for CpGs occurring in non-SV regions, adding the proportions to a difference vector. With these 2 vectors, we then performed a Mann–Whitney *U* test to determine whether SVs were more likely to introduce CpGs than non-SV regions.

### Determining quantification bias between genomes

To determine whether there was a significant quantification bias between the Brahman and Angus reference genomes for a given sample, we compared the vector of all CpG sites with at least 10× coverage when mapped to Angus in sample *I* against the vector of all CpG sites with at least 10× coverage when mapped to Brahman. To ensure the vectors were equal, we randomly subset the larger vector to be the same length as the smaller, that is, if the Brahman reference genome had more CpG sites with 10× coverage for that sample, the Brahman vector was randomly subset to match the number of CpG sites in the Angus vector for that sample. We repeated this for all samples using all CpGs and again with just the CpG sites marked as shared (Table 3; Supplementary Table S8). We then pooled all CpG sites for each sample within a group (e.g., all samples from BTBT) and determined whether the CpG methylation differed significantly for that group when mapped to Angus and Brahman. The *P*-value was determined using a Wilcoxon rank sum test and adjusted for multiple testing using the Benjamini–Hochberg procedure. We identified variable CpGs by matching the shared CpGs between reference genomes and then identifying those with an absolute methylation difference greater than 10%.

To further assess the presence of a quantification bias, we generated non-overlapping windows of both Angus and Brahman reference genomes with a length of 100 kb. We then aligned these 100-kb sequences to the opposite reference genome with “minimap2 –t 8 –c –x map-ont.” We then used the output PAF files to identify homologous 100-kb windows from each genome. We extracted the methylation values from each window for a given group when mapped to each genome. For example, consider homologous region “A” (HR-A), identified between the Angus and Brahman reference genomes. To identify whether this region exhibits differential methylation between reference genomes and thus exhibits quantification bias, we quantified all methylation values that fell within the Angus coordinates of HR-A using the methylation values for BTBT samples mapped to the Angus reference. We then quantified the methylation values that fell within the Brahman coordinates of HR-A using the methylation values for BTBT samples mapped to the Brahman reference. This gave 2 vectors of methylation values, which were then used as input to a Wilcoxon signed-rank test to determine whether the methylation values in this region were significantly different between genomes. The Benjamini–Hochberg false discovery procedure was used to adjust for multiple testing. This was repeated for each of the 4 groups.

### Identification of differentially methylated regions

The methylKit package (v. 1.22.0) [76] was used to identify DMRs between breed and POE groups. We investigated breed effects by comparing BIBI samples with BTBT samples, maternal effects by comparing samples with BIBI dams (BIBI; BTBI) and those with BTBT dams (BTBT; BIBT), and paternal effects by comparing samples with BIBI sires (BIBI; BIBT) to those with BTBT sires (BTBT; BTBI) (Supplementary Table S16). The reference group was always the breed that matched the reference genome. For example, when BIBI and BTBT WGBS reads were aligned to the Brahman reference genome, BIBI samples were treated as the control group and BTBT as the treatment group.

We followed the pipeline described by the methylKit authors for DMR analysis [76]. Briefly, we only considered CpGs that were identified as shared. We then removed all CpG sites with less than 10× coverage and more than the 99.9th percentile of coverage. Reads with too high coverage (e.g., from PCR duplication bias) can impair the accurate determination of the methylation percentage at that site and is a recommended preprocessing step for the methylKit [76]. We then normalized the coverage using the default methylKit normalization strategy. We merged the CpG counts per group using the “unite” function with “destrand = T” and “min.per.group = 5L” so that a given CpG site had to be covered by at least 10 reads in 5 out of 6 samples per group. For the parent of origin DMR analyses, we set “min.per.group = 10L.”

We then identified differentially methylated cytosines between groups using the “calculateDiffMeth” function, with sex as a covariate in the model. To determine differentially methylated regions, we used the “tileMethylCounts” function with default parameters to divide the genome into regions for differential methylation analysis. This step allowed methylKit to divide the genome into non-overlapping regions based on the tiling windows. MethylKit then models the methylation at a given cytosine or region by fitting a logistic regression:


\begin{eqnarray*}
\log \left( {\frac{{{P}_i}}{{1 - {P}_i}}} \right) = {\beta }_0 + {\beta }_1*{T}_i + {{\mathrm{a}}}_{{\mathrm{sex}}}*{\mathrm{Se}}{{\mathrm{x}}}_i
\end{eqnarray*}

*P_i_* denotes the methylation proportion for sample *i* in samples 1,…, *n*, where *n* is the number of samples across both groups in the comparison [76]. *T_i_* represents the groups (0 for control, 1 for treatment). ${\beta }_0$ denotes the log odds of the control group (fraction of reads reporting C/1 – the fraction of reads reporting C). ${\beta }_1$ denotes the log odds ratio between the control and treatment. ${{\mathrm{\alpha }}}_{{\mathrm{sex}}}$ denotes the parameter for the sex covariate and ${\mathrm{Se}}{{\mathrm{x}}}_i\ $ denotes the sex (0 = male; 1 = female) for sample *i*. For further details, refer to Akalin et al. [76]. This design resulted in 6 different logistic models being fit: model 1A (breed comparison when aligned to the Angus reference), model 1B (breed comparison when aligned to the Brahman reference), model 2A (dam of origin comparison when aligned to the Angus reference), model 2B (dam of origin when aligned to the Brahman reference), model 3A (sire of origin when aligned to the Angus reference), and model 3B (sire of origin when aligned to the Brahman reference) (Supplementary Table S16). Any DMRs identified were either hypo- or hypermethylated with respect to the control group. We retained all DMRs with a difference in methylation of ≥10% and a *q*-value of ≤0.01 for further analysis. The *q*-values were obtained using the sliding linear model (SLIM) method [111]. We repeated this pipeline to identify DMCs, with the exclusion of the tiling step. An overview of the samples, reference genomes, types of CpGs, and DMR analysis is given in Fig. 1A–G.

### DMR coordinate conversion

To determine if a given DMR changed methylation direction between genomes, we had to convert the coordinates of DMRs identified by alignment with the Angus genome to Brahman coordinates and vice versa. We considered a DMR as changing methylation direction if, for example, it is hypomethylated in BIBI samples compared to BTBT samples when using the Brahman reference but becomes hypermethylated in BIBI using the Angus reference genome. To investigate this, we first converted the DMR bed files to GTF files and then used Liftoff (v.1.6.2) [112] to transfer coordinates from one reference genome to the other. We then identified DMRs reciprocally overlapping one another by at least 90% between the 2 genomes, with these DMRs being considered successfully lifted over. DMRs that did not overlap by 90% were not considered for the methylation direction change analysis.

### RNA-seq mapping and preprocessing

RNA-seq reads were mapped to the Brahman and Angus genomes as in the WGBS mapping step. Briefly, reads were checked for quality using FastQC (v. 0.11.4) [99] before being trimmed with Trim Galore (v. 0.4.2) [100] with the parameters “–quality 10” and “–length 100.” Reads were mapped using HiSAT2 (v. 2.1.0) to both the Brahman and Angus reference genomes [39]; alignment files were sorted using SAMtools (v. 1.10) [102]. FeatureCount from the Rsubread package (v. 2.10.5) [113] was used to count how many reads mapped to genes.

### Differential gene expression

Differential gene expression analysis was performed using an in-house R script with the DESeq2 (v. 1.40.2) [114] R package. The genome annotation was based on Ensembl v.104 for Brahman and Angus. The orientation of the genes was reversed where necessary to correspond with the orientation of the chromosomes of ARS-UCD1.2. In the breed comparison, where the number of samples in each group was 6, we retained genes that had a count ≥10 in at least 3 samples. In the POE comparisons, we retained genes that had a count ≥10 in at least 6 samples. The group mean parameterization method was used to identify differentially expressed genes between groups with the following model design: “∼0 + Genetics + Sex + Batch” [97]. DESeq2 then estimated size factors and dispersion and finally fit a negative binomial generalized linear model to identify DEGs. We then compared differential gene expression between purebred Angus and Brahman, Angus dams and Brahman dams, and Angus sires and Brahman sires. Genes with significant differences in gene expression at an adjusted *P* ≤ 0.05 were retained for further analysis.

Furthermore, we performed canonical correlation analysis (CCA) to estimate the canonical correlation between the gene expression and methylation data. The first 5 canonical correlations were >0.7 with *P* < 0.05 (Supplementary Table S17).

### Identifying imprinted genes

We downloaded a list of genes with evidence of imprinting in human, mouse, and cattle from Morison et al. [115, 116]. We then used OrthoFinder (RRID:SCR_017118) to identify human orthologs of both Brahman and Angus genes [117], allowing us to assign Human Genome Organisation Gene Nomenclature Committee (HGNC) symbols to genes in each breed. To do this, we first identified which Brahman proteins had orthologs in human. We then identified the genes that encoded these proteins and used this information to assign human and Brahman genes as orthologs. We repeated the process for the Angus genes. We then identified all genes that could be assigned an HGNC symbol from the Brahman Ensembl annotation version 104 that were also present in the imprinted gene list (Supplementary Table S18). This filtering gave us 80 imprinted genes for Brahman autosomes. We repeated the process for Angus using the Angus Ensembl annotation version 104 and identified 79 imprinted genes. The discrepancy is due to 1 imprinted gene for Angus occurring on an unplaced scaffold.

### Linking DMRs to DEGs

For each DEG, we considered 5 different regions in and around the gene where DMRs might have an influence. These regions included putative enhancer regions, 5 kb outside the gene body, and the gene body itself (Supplementary Fig. S3). The upstream putative enhancer region started 130 kb upstream of the gene and then stopped 5 kb upstream of the gene body for a total length of 125 kb. We repeated this for the downstream putative enhancer region, starting 5 kb downstream of the gene body and extending out 125 kb. This number was based on the median distance between enhancers and their gene targets [118]. The 5-kb region was from upstream of the start of the gene body to the start of the gene body. Again, this was repeated for the downstream 5-kb region. The gene body was the region annotated as “gene” in the Ensembl annotation file. We then found all DMRs that overlapped these regions by at least 90% of their length using “bedtools intersect” with the “-f” and “-F” arguments, both set at 0.9 and the “-e” argument set to True.

## Availability of Source Code and Requirements

Project name: Brahman_Angus_WGBS

Project homepage: https://github.com/DaviesCentreInformatics/Brahman_Angus_WGBS

Operating system(s): Linux and MacOS

Programming language: Python, R

Other requirements: NA

License: MIT

Any restrictions to use by nonacademics: No restrictions

RRID: NA

bio.tools ID: NA

## Supplementary Material

giae061_GIGA-D-23-00314_Original_Submission

giae061_GIGA-D-23-00314_Revision_1

giae061_GIGA-D-23-00314_Revision_2

giae061_GIGA-D-23-00314_Revision_3

giae061_Response_to_Reviewer_Comments_Original_Submission

giae061_Response_to_Reviewer_Comments_Revision_1

giae061_Response_to_Reviewer_Comments_Revision_2

giae061_Reviewer_1_Report_Original_Submission Wenbin Guo -- 12/18/2023 Reviewed

giae061_Reviewer_1_Report_Revision_1 Wenbin Guo -- 3/27/2024 Reviewed

giae061_Reviewer_2_Report_Original_Submission Alan L Archibald -- 12/19/2023 Reviewed

giae061_Supplemental_Files

## Data Availability

The datasets generated and analyzed during the current study are available in the NCBI SRA repository under BioProject: PRJNA626458. Snapshots of our code and other data further supporting this work are openly available in the *GigaScience* repository, GiagDB [119].

## Additional Files

**Additional File 1**. Supplementary figures referred to in the main text.

**Additional File 2**. WGBS mapping statistics.

**Additional File 3**. Table describing the number of CpGs in each reference and what percentage of those have 10× coverage.

**Additional File 4**. Mean global CpG coverage.

**Additional File 5**. Global CpG methylation.

**Additional File 6**. Methylation of CpGs in different genomic regions.

**Additional File 7**. Alignment statistics of cross-reference genome CpG mapping.

**Additional File 8**. SNP and SV enrichment at CpG sites.

**Additional File 9**. Methylation quantification bias between genomes.

**Additional File 10**. Table containing the variable CpG sites between the genomes.

**Additional File 11**. Table of differentially methylated regions that exhibited direction changes.

**Additional File 12**. The 100-kb windows that display quantification bias when mapped between the 2 genomes.

**Additional File 13**. Number of DMRs in each comparison.

**Additional File 14**. Number of up- and downregulated genes in each comparison.

**Additional File 15**. DMR–DEG overlap after normalizing for length.

**Additional File 16**. Minimap2 accuracy in detecting SNPs introduced to the reference genome.

**Additional File 17**. Sample information.

**Additional File 18**. Results from the canonical correlation analysis.

**Additional File 19**. List of imprinted genes.

**Supplementary Fig. S1**. Correlation heatmaps of all 24 samples mapped to Brahman and Angus reference genomes. Left: Correlation heatmap for all 24 samples when mapped to the Brahman reference and using all CpGs with ≥10× coverage in all samples. Darker colors denote lower correlation. Right: Correlation heatmap for all 24 samples when mapped to the Angus reference using all CpGs with ≥10× coverage in all samples. Darker colors denote lower correlation. Dark blue denotes BTBT samples, cyan denotes BTBI samples, green denotes BIBT samples, and orange denotes BIBI samples.

**Supplementary Fig. S2**. Number of C > T, G > A, and other SNPs between Brahman and Angus reference genomes. Horizontal bar graph showing the number of SNPs that affected a CpG site. Most (∼81%) SNPs that occur in the CpG site are either C > T or G > A with other alterations making up ∼19% of SNPs.

**Supplementary Fig. S3**. Comparison of global methylation per sample when mapped to Angus and Brahman reference genomes. (A) Strip plot showing difference in global methylation between each sample when mapped to Angus vs. Brahman reference genomes. Each sample is denoted by a different color. Each panel represents one of the genetic groups in the order BTBT, BTBI, BIBT, and BIBI. The x-axis denotes the reference genome and the y-axis denotes the global methylation. (B) Same as A except only shared CpG sites were considered.

**Supplementary Fig. S4**. Two-dimensional KDE plots of shared CpG methylation variation. KDE plots comparing methylation of a given CpG site when mapped to Angus (x-axis) compared to the methylation of that CpG site when mapped to Brahman (y-axis). This is using all CpG sites that passed our filtering criteria.

**Supplementary Fig. S5**. Graphical representation of search region. For each feature labeled as “gene” in the GTF files for Brahman and Angus, we took 130 kb either side of the gene body. The outermost region in green represents the putative enhancer regions; these were 125 kb in length, regardless of the size of the gene body. Next were the 5-kb regions, which extended 5 kb up or downstream of the gene body. Lastly, the gene body was kept as the length that it was in the GTF file.

## Abbreviations

BIBI: Brahman sire and Brahman dam; BIBT: Brahman sire and Angus dam; BTBI: Angus sire and Brahman dam; BTBT: Angus sire and Angus dam; CCA: canonical correlation analysis; CpG: cytosine phosphodiester-linkage guanine; DEGs: differentially expressed genes; *DGAT1*: diacylglycerol O-acyltransferase 1; *DSCAM*: DS cell adhesion molecule; HGNC: Human Genome Organisation Gene Nomenclature Committee; *HTR2A*: 5-hydroxytryptamine receptor 2A; *IGF2*: insulin-like growth factor 2; indel: insertion/deletion; *LIN2B*: Lin-28 homologous B; *MKRN3*: Makorin ring finger protein 3; *NDN*: necdin MAGE family member; *NNAT*: neuronatin; *PARD6G*: Par-6 family cell polarity regulator gamma; POE: parent-of-origin effect; *PPP1R9A*: protein phosphate 1 regulatory subunit 9A; *SLC22A1*: Solute-carrier family 22 member 18; SNP: single nulceotide polymorphism; SV: structural variant; *TFPI2*: tissue factor pathway inhibitor 2; TpG: thymine phosphodiester-linkage guanine; UTR: untranslated region; WGBS: whole-genome bisulfite sequencing; *ZC3H12C*: zinc finger CCCH-type containing 12C.
