# Supplementary material for: DNA methylation analysis to differentiate reference, breed, and parent-of-origin effects in the bovine pangenome era
Source: Gigascience. 2024 Oct 17;13:giae061. doi: 10.1093/gigascience/giae061 (PMC11484048; doi:10.1093/gigascience/giae061)
Supplement: giae061_GIGA-D-23-00314_Original_Submission [file giae061_giga-d-23-00314_original_submission.pdf]

GigaScience

The genetics of epigenetics in the bovine pangenome era

--Manuscript Draft--

**Manuscript Number:**	GIGA-D-23-00314
**Full Title:**	The genetics of epigenetics in the bovine pangenome era
**Article Type:**	Research
**Funding Information:**	JS Davies Bequest — Not applicable
**Abstract:**	Background<br>Most DNA methylation studies have used a single reference genome with little attention paid to the bias introduced due to the reference chosen. Genetic variation, including single nucleotide polymorphism (SNPs) and structural variants (SVs), can lead to differences in methylation sites (CpGs) between individuals of the same species. We analysed whole genome bisulfite sequencing (WGBS) data from the fetal liver of Angus (Bos taurus taurus), Brahman (Bos taurus indicus) and reciprocally crossed samples. Using reference genomes for each breed from the Bovine Pangenome Consortium, we investigated the influence of reference genome choice on the breed- and parent-of-origin effects in methylome analyses.<br>Results<br>Our findings revealed that about 75% of CpG sites were shared between Angus and Brahman, ~5% were breed-specific, and ~20% were unresolved. We demonstrated up to 2% quantification bias in global methylation when an incorrect reference genome was used. Furthermore, we found that SNPs and SVs were 8-fold (p-value <) and 1.13-fold (p-value <) higher in CpGs, respectively, compared to the rest of the genome. We found a poor association between differentially methylated regions (DMRs) and differentially expressed genes (DEGs) and suggest that DMRs may be impacting enhancers that target these DEGs. DMRs overlapped with imprinted genes, of which one, Dgat1, which is important for fat metabolism and weight gain, was found in the breed-specific and sire-of-origin comparisons.<br>Conclusions<br>This work demonstrates the need to consider reference genome effects to explore genetic and epigenetic differences accurately and identify DMRs involved in controlling certain genes.
**Corresponding Author:**	Callum MacPhillamy<br>The University of Adelaide<br>Roseworthy, SA AUSTRALIA
**Corresponding Author Secondary Information:**	
**Corresponding Author's Institution:**	The University of Adelaide
**Corresponding Author's Secondary Institution:**	
**First Author:**	Callum MacPhillamy
**First Author Secondary Information:**	
**Order of Authors:**	Callum MacPhillamy
	Tong Chen
	Stefan Hiendleder
	John Williams
	Hamid Alinejad-Rokny
	Wai Low
**Order of Authors Secondary Information:**	
**Additional Information:**	

Powered by Editorial Manager® and ProduXion Manager® from Aries Systems Corporation

Question	Response
Are you submitting this manuscript to a special series or article collection?	No
**Experimental design and statistics**<br><br>Full details of the experimental design and statistical methods used should be given in the Methods section, as detailed in our Minimum Standards Reporting Checklist. Information essential to interpreting the data presented should be made available in the figure legends.<br><br>Have you included all the information requested in your manuscript?	Yes
**Resources**<br><br>A description of all resources used, including antibodies, cell lines, animals and software tools, with enough information to allow them to be uniquely identified, should be included in the Methods section. Authors are strongly encouraged to cite Research Resource Identifiers (RRIDs) for antibodies, model organisms and tools, where possible.<br><br>Have you included the information requested as detailed in our Minimum Standards Reporting Checklist?	Yes
**Availability of data and materials**<br><br>All datasets and code on which the conclusions of the paper rely must be either included in your submission or deposited in publicly available repositories (where available and ethically appropriate), referencing such data using a unique identifier in the references and in the "Availability of Data and Materials" section of your manuscript.	Yes

Have you have met the above requirement as detailed in our Minimum Standards Reporting Checklist?	

Powered by Editorial Manager® and ProduXion Manager® from Aries Systems Corporation

Manuscript

Click here to access/download;Manuscript;Methylation_paper_clean.docx

The genetics of epigenetics in the bovine pangenome era

Authors:

Callum MacPhillamy[1], Tong Chen[1], Stefan Hiendleder[1,2], John L. Williams[1,3], Hamid Alinejad-Rokny[4], Wai Yee Low[1]

[1]The Davies Research Centre, School of Animal and Veterinary Sciences, University of Adelaide, Roseworthy, SA 5371, Australia

[2]Robinson Research Institute, The University of Adelaide, North Adelaide, SA 5006, Australia

[3]Department of Animal Science, Food and Nutrition, Università Cattolica del Sacro Cuore, 29122 Piacenza, Italy

[4]BioMedical Machine Learning Lab, The Graduate School of Biomedical Engineering, UNSW, Sydney, NSW 2052, Australia

Abstract

Background

Most DNA methylation studies have used a single reference genome with little attention paid to the bias introduced due to the reference chosen. Genetic variation, including single nucleotide polymorphism (SNPs) and structural variants (SVs), can lead to differences in methylation sites (CpGs) between individuals of the same species. We analysed whole genome bisulfite sequencing (WGBS) data from the fetal liver of Angus (*Bos taurus taurus*), Brahman (*Bos taurus indicus*) and reciprocally crossed samples. Using reference genomes for each breed from the Bovine Pangenome Consortium, we investigated the influence of reference genome choice on the breed- and parent-of-origin effects in methylome analyses.

Results

Our findings revealed that about 75% of CpG sites were shared between Angus and Brahman, ~5% were breed-specific, and ~20% were unresolved. We demonstrated up to 2% quantification bias in global methylation when an incorrect reference genome was used. Furthermore, we found that SNPs and SVs were 8-fold (p-value < 5×10^{-324}) and 1.13-fold (p-value < 5×10^{-324}) higher in CpGs, respectively, compared to the rest of the genome. We found a poor association between differentially methylated regions (DMRs) and differentially expressed genes (DEGs) and suggest that DMRs may be impacting enhancers that target these DEGs. DMRs overlapped with imprinted genes, of which one, *Dgat1,* which is important for fat metabolism and weight gain, was found in the breed-specific and sire-of-origin comparisons.

Conclusions

This work demonstrates the need to consider reference genome effects to explore genetic and epigenetic differences accurately and identify DMRs involved in controlling certain genes.

Keywords

Bisulfite sequencing, methylation, CpG, structural variants, *Dgat1*, differentially methylated region, bovine pangenome

Background

DNA methylation is a key epigenetic modification that plays a vital role in regulating gene expression, repression of transposable elements, and parental chromosome specific regulation through genomic imprinting and X-chromosome inactivation [1, 2]. In mammals, DNA methylation primarily occurs at C-phosphate-G dinucleotides (CpGs) [3, 4]**.** DNA methylation influences gene expression either by recruiting proteins involved in gene repression or by blocking transcription factor binding sites (TFBSs) within promoter regions [5]. Hypomethylation of a promoter has been associated with the increased expression of the corresponding gene [6]. However, recent work has shown that promoter hypermethylation can also lead to gene expression [7]. The relationship between DNA methylation and gene expression is complicated by the role of enhancer methylation in regulating gene expression [8, 9]. In the presence of high DNA methylation, enhancers have been observed to be associated with high levels of the histone modification H3K27ac [10], which is often associated with active gene transcription [11-14].

Most DNA methylation studies have used a single reference genome with little or no knowledge of the impact of reference genome choice on the interpretation of methylome differences. The choice of reference genome has been shown to have an impact on DNA methylation analyses, with up to a nine per cent bias reported when the incorrect reference is used (Wulfridge, Langmead [15]. Using a single reference genome has been shown to bias read mapping in favour of reads with high similarity to the reference [16-20]. This bias occurs because reads containing non-reference alleles or regions that are divergent from the reference either align poorly, align to the wrong genomic region, or fail to align. This reference bias has been shown to affect analyses of cattle breeds [21, 22], humans [17, 23], and sheep [18].

The majority of mammalian methylation occurs in the CpG context. Consequently, a single nucleotide polymorphism (SNP) can remove a methylation site, thus introducing a reference bias if the individuals being studied do not possess the same SNPs as the individual used to generate the reference. In addition to SNPs, structural variations (SVs) among individuals may remove or introduce CpG sites. The disparity between CpG sites can confound analyses by identifying a methylated CpG in one individual when another individual has no CpG at that position. As a result, SNPs and SVs can both introduce bias, as reads may be unambiguously assigned in duplicated regions not found in the reference and mismatches in reads can result in the loss of some reads. Moreover, if individuals have insertion SVs that carry CpG sites, reads that originate from the insertion/deletion (indel) regions can only be mapped if the complete sequence data for the population is available. We consider SNPs and SVs that alter CpGs as genetic changes with potential effects on epigenetic regulation. We use the term ‘genetics of epigenetics’ to describe this phenomenon.

As more genomes for a given species become available, the research community is gradually shifting toward using pangenomes to account for genetic variation within a population more accurately. A pangenome is a collection of the genomes of multiple individuals, representing all genetic variation within that population and is thus a more accurate way to represent genetic diversity than a single reference genome [24]. Current pangenome projects include human [24, 25], cattle [26], and maize [27]. As genetic differences within a population can result in CpG differences, these pangenomes provide a valuable resource to study DNA methylation changes between diverse groups of individuals of the same species.

The two main lineages of modern cattle breeds are generally accepted to have been derived from two separate domestication events of the wild auroch (*Bos primigenius*) [28]. The first domestication event occurred in the Fertile Crescent around 10,000 years ago and gave rise to *Bos taurus taurus* from the wild auroch, *B. p. primigenius* [29-31]. A second domestication event occurred in the Indus Valley, ~1,500 years later, from *B. p. nomadicus*, which separated from the *B. p. primigenius* around 250-330,000 years ago [32] and gave rise to *Bos taurus indicus*. The subspecies are referred to here as taurine and indicine cattle, respectively [28], where the Angus breed represents taurine cattle, and Brahman is representative of indicine cattle. Angus and Brahman have contrasting phenotypes, e.g., Angus have been bred for meat production traits [33], whereas Brahman have superior heat and disease tolerance traits [34, 35]. DNA methylation differences may partly be responsible for the phenotypic differences between these two breeds.

As expected from their domestication history, Angus and Brahman cattle represent genetically highly diverged subspecies [36, 37]. However, as they produce fertile offspring when mated [38], they are an appropriate model to investigate the impact of using a single reference genome on methylome analysis of two genetically diverse populations. We have previously produced high-quality haplotype-resolved reference genomes for Angus and Brahman [39], which are genomes included in the Bovine Pangenome Consortium project [26] and are used in the present study.

Breed-specific differences in CpGs may occur due to a SNP, such as those caused by spontaneous deamination [40, 41], or may result from SVs. A single SNP affecting a CpG site has been shown to drastically alter the methylation state of the *Igf2* gene in pigs leading to changes in muscle development [42]. SVs have been associated with decreased methylation in cancers [43] and with changes in the methylation of the kappa opioid receptor (*kor)* promoter associated with KOR dysfunction and schizophrenia [44].

Parent-of-origin effects (POEs) occur when only one allele is expressed, and the phenotype in the offspring may depend on which parent contributed the expressed allele [45]. Reciprocal crossing is necessary to elucidate how each parent contributes to a particular phenotype. POEs have been observed in hybrids of mice [46], cattle [47] and pigs [48], and there is increasing evidence that fetal development is influenced by POEs [49-53]. Given the similarity in gestation period between cattle and human and the single fetus with similar development trajectory, cattle are an attractive model species to study human reproductive and developmental biology [54-57].

To investigate the potential impact of reference genome choice on methylome analyses and to improve our understanding of the genetic and epigenetic factors driving the phenotypic differences between cattle subspecies, we used WGBS data from 24 fetal liver samples of purebred Brahman and Angus cattle and their reciprocal-crosses to perform a comprehensive assessment of the impact of reference genome choice on differential methylation and gene expression. This study serves as an example of how to investigate epigenetic differences between breeds, strains, and populations within species and informs about reference genome effects on the interpretation of methylome analyses.

Results

Mapping of WGBS data and calling CpG

Each of the 24 samples representing the four genetic groups (Fig 1A; S. table 1) was sequenced for WGBS analysis to at least 30X coverage and then mapped separately to the Brahman and Angus genomes (Fig 1B). An average mapping rate of ~95% was achieved when reads were mapped from each sample to the Brahman reference genome (Table 1; S. table 1). All samples had at least 10X coverage for 93% of the Brahman sequence. Using the Angus reference, all samples had 10X coverage for at least 90% of the sequence (Table 1; S. table 1).

We performed all analyses twice for each reference genome, first using all CpGs with ≥10X in each reference genome and again where we retained only CpG sites with ≥10X that we could confidently assign as being shared between both breeds. Between 85% and 88% of autosomal CpG sites had coverage ≥ 10X when considering all CpG sites on both the Brahman and Angus reference and shared CpG sites (Table 1; S. table 2). Median coverage of CpG sites across all samples ranged from 25-34X regardless of reference and CpG sites considered (i.e., shared or all) (Table 1; S. table 3).

Clustering of genetic groups

Comparing the methylation patterns between the genetic groups, we found that samples within a genetic group were more similar to each other than with samples from other groups. For example, samples from the BTBT group had higher correlations with other BTBT samples than BIBI samples. BTBT had the highest within-group Pearson correlations (r between 0.81 and 0.88) (S. figure 1). The samples that were least correlated with one another were those belonging to BTBT and BIBI, with correlations between 0.75 and 0.78. Samples from the reciprocal cross groups (BIBT; BTBI) had similar correlations with other samples within their own group (r between 0.81 and 0.83) as well as with samples from the alternative reciprocal cross (r between 0.80 and 0.83). Overall, correlations were high within each genetic group ($r \geq 0.8$) (S. figure 1).

We performed a principal component analysis of the 24 samples using CpG sites covered by at least 10 reads in all samples (Fig 2A). BTBT and BIBI formed distinct clusters distant from one another, with the two hybrid genetic groups clustering much closer together and between the two parental genetic groups. Nevertheless, the hybrid groups were clearly separated on the PCA plot (Fig 2A). The separation of groups we observed from the methylation data was similar to that seen for the gene expression data (Fig 2B).

Overview of DNA methylation patterns

Samples had global mean CpG methylation of between 47-62%, with most samples ranging from 49-54% (Fig 3A; S. table 4). Mean exon CpG methylation was 48-58% for all samples, with most samples ranging from 48-54% methylation (S. Fig 2; S. table 5). The 5’ UTRs and promoter regions had the lowest mean CpG methylation percentage across all samples, between 9-14% and 14-19%, respectively (S. Fig 3-4; S. table 5). The intergenic regions displayed mean methylation levels that ranged from 47 – 65% (S. Fig 5; S. table 5), with most samples ranging from 49-57%, similar to the global mean. The introns exhibited slightly higher methylation levels, with means ranging from 51-66% (S. Fig 6; S. table 5), and most samples in the 53-59% range. The 3’ UTRs revealed the highest overall CpG methylation levels, 57-69% (S. Fig 7; S. table 5). Lastly, the predicted enhancers, according to MacPhillamy, Alinejad-Rokny [58], exhibited CpG methylation levels ranging from 42-53%, with most samples within 43-48% methylated (S. Fig 8; S. table 5). Similar methylation patterns were observed using only shared CpGs in exons, 5’UTRs, intergenic, introns, promoters, predicted enhancers, and 3’UTRs, regardless of the reference genome used.

Shared and breed-specific CpGs

We were able to confidently identify 74-75% of CpGs in the Brahman and Angus genomes that were shared between the two breeds (Table 2; S. table 6). We found that around four per cent of CpG alignments contained a SNP between reference genomes (S. table 6; S. figure 9), i.e., were breed-specific. About 22% of CpGs could not be confidently assigned as shared or breed-specific and so were not considered in the shared CpG analysis. By definition, breed-specific regions with CpG sites did not align when the other breed genome was used as the reference. We found ~1% of such CpG sites. In total, the SNP change and breed-specific categories of CpG sites constituted 4.7% and 4.9% of CpGs between the Angus and Brahman reference genomes, respectively, and were considered breed-specific.

Enrichment of SNPs affecting CpG sites

Using the autosomal SNPs identified by MUMmer, we observed that Brahman and Angus autosomal sequences differ by an average of ~0.6% (S. table 7). SNPs in CpG sites were found to be enriched by ~8 times compared to the genome-wide average (binomial test, p-value < 5×10^{-324}), which means there is a higher level of divergence between Angus and Brahman at CpG sites than at other autosomal sites. Looking more closely at the CpG SNP changes, we found that most (~81%) of the CpG SNP were either C to T or G to A changes (S. figure 7). The remaining SNP changes individually comprised less than ~10% of the total observed mutations at CpG sites (S figure 9; S. table 6).

Increased number of CpGs within structural variants

Next, we tested whether CpGs were enriched in SVs compared to the rest of the genome. Using the Brahman genome as the reference, we observed 25,009 SVs between Brahman and Angus, making up ~27Mb of sequence. We observed 1.13-fold more CpGs within SVs than in non-SVs (binomial test, p-value < 5×10^{-324}). When considering CpGs affected by SNPs and SVs, the CpG mutation rate is approximately 4% between Brahman and Angus compared to the genome-wide mutation rate of around 1.7%.

Choice of reference genome influences methylome results

We observed a statistically significant difference in global CpG methylation in BTBT, BIBT and BIBI depending on whether the Angus or Brahman genome was used to quantify methylation at 10X coverage (paired Wilcoxon test, p-value < 0.05) (Figure 3A; S. figure 10). Although not statistically significant, the BTBI group (paired Wilcoxon test, p-value = 0.06) approaches significance. The reference genomes showed no difference in global methylation when only shared CpG sites were considered (Figure 3B). When comparing global CpG methylation differences between samples mapped to Brahman versus those mapped to Angus, the largest quantification bias was ~2% for BTBT. The other quantification biases were ~0.8%, ~0.7% and ~0.3% for BTBI, BIBT and BIBI samples, respectively (Figure 3A).

To further investigate the influence of the reference genome on downstream analyses, we compared DMRs identified by the two reference genomes to evaluate if the direction of methylation changed, i.e., hypermethylated vs hypomethylated and vice versa. Approximately 12% (28,922) of Angus DMRs overlapped with Brahman DMRs by at least 90% of their length. Of the DMRs that mapped to the Angus reference, 3,575 showed changes in methylation direction when mapped to the Brahman reference (S. table 8). We observed similar numbers (3,581) when lifting DMRs from Brahman to Angus (S. table 8). There were no methylation direction changes when we considered differentially methylated cytosines (DMCs).

Breed-specific CpGs show distinct methylation patterns

Looking more closely at the breed-specific CpGs, we first determined whether a given breed-specific CpG was methylated or not. We binned individual CpGs into "unmethylated" (CpG methylation ≤ 35%) and "methylated" (CpG methylation ≥ 65%). CpGs that were between 35% and 65% were considered "hemimethylated" and were excluded from this analysis. We only considered breed-specific CpGs with at least 10X coverage in all purebred samples mapped to their respective genome. We then randomly sampled 1000 CpG sites for 100 iterations, recording the number of "methylated" and "unmethylated" CpGs for each breed. We observed significantly more Brahman-specific CpGs as hypomethylated than hypermethylated (Mann-Whitney U-test, p =2.5×10^{-34}). Interestingly, we observed the inverse when considering Angus-specific CpGs; significantly more Angus-specific CpGs were hypermethylated than hypomethylated (Mann-Whitney U-test, p =2.5×10^{-34}) (Figure 3C).

Breed-specific DMRs show poor association with DEGs

As we observed a quantification bias when using all CpGs mapped against each reference genome, we restricted breed-specific and POE analyses to those CpGs identified as shared. Additionally, we examined the number of DMRs at the 25% and 50% difference thresholds, i.e., more stringent thresholds for calling DMRs which substantially reduced the numbers (S. table 9). Given that minor changes of less than 10-15% in methylation have been observed to influence gene expression and phenotype [59, 60], we used a difference threshold of 10% to interpret the results.

Using Brahman as the reference, we identified 123,602 DMRs and 1,397 DEGs (S. table 9; S. table 10). Of the 123,602 DMRs observed, ~20% (25,367) overlapped with the surrounding region of the significant DEGs, with most (~69%) falling within the putative-enhancer region. Only 0.8% of the DMRs overlapped with promoters of DEGs. When the Angus reference was used, of the 125,544 DMRs identified, ~32% (40,787) of those overlapped with a DEG. Most (~64%) of these DMRs fell into the putative enhancer region, while only 0.8% of the DMRs overlapped with a DEG promoter, despite substantially more (2,151) DEGs observed (S. table 10).

We then examined the overlap of DMRs and imprinted genes, first using Brahman as the reference. Here, ~1% (1,282) of the DMRs identified between BIBI and BTBT overlapped 79 imprinted genes. Only one imprinted gene, Par-6 family cell polarity regulator gamma (*Pard6g*), did not overlap with any DMR. Most DMRs (~72% of the 1,282) that overlapped an imprinted gene fell into putative enhancer regions. Seven imprinted genes were significantly differentially expressed when comparing BIBI and BTBT (Table 3). These genes were SPARC related modular calcium-binding 1 (*Smoc1*), neuronatin (*Nnat*), ganglioside induced differentiation associated protein 1 like 1 (*Gdap1l1*), diacylglycerol O-acyltransferase 1 (*Dgat1*), solute-carrier family 22 member 18 (*Slc22a18*), potassium voltage-gated channel subfamily Q member 1 (*Kcnq1*) and protein phosphatase 1 regulatory subunit 9A (*Ppp1r9a*). *Nnat* and *Gdap1l1* had higher expression in BTBT, and the remaining five DEGs, *Smoc1*, *Dgat1*, *Slc22a18*, *Kcnq1* and *Ppp1r9a*, had higher expression in BIBI. We observed seven imprinted DEGs using the Angus reference; however, the genes identified differed: estrogen receptor 2 (*Esr2*), *Ppp1r9a,* and DLG-associated protein 2 (*Dlgap2*) had higher expression in BIBI (Table 4), while *Nnat* and zinc finger protein 90 (*Zfp90*) had higher expression in BTBT.

Dam-of-origin methylation shows less overlap with imprinted genes

To investigate the dam-of-origin effects (DOEs), we compared samples with Brahman dams (BIBI and BTBI) with those with Angus dams (BTBT and BIBT). Using the Brahman genome as the reference, 243 DEGs were identified in the DOE comparison. Around 3% (497) of the DMRs overlapped with DEGs. Most DMRs (~357) fell into the putative enhancer region; 0.9% of DMRs overlapped with a DEG promoter (S. table 9; S. table 10). There were 52 imprinted genes that overlapped with ~1% (188) of the DMRs identified in the comparison. Of the 188 DMRs that overlapped with an imprinted gene, 125 DMRs overlapped with the putative enhancer region. Only two imprinted genes were significantly differentially expressed (Table 3). Zinc finger CCCH-type containing 12C (*Zc3h12c*) and *Ppp1r9a* had higher expression in samples with Brahman mothers. Using Angus as the reference, we observed 809 (~4%) DMRs overlap with the 260 DEGs. Most (592) of these DMRs fell into the putative enhancer regions. The same imprinted DEGs (*Zc3h12c* and *Ppp1r9a*) were identified using the Angus genome as the reference (Table 4).

Sire-of-origin methylation may be driving differential gene expression

To investigate the sire-of-origin effects (SOEs), we compared samples with Brahman sires (BIBI and BIBT) with those with Angus sires (BTBT and BTBI). Using the Brahman reference, we identified 62,056 DMRs and 1,364 DEGs in the sire group comparison using shared CpGs (S. table 9; S. table 10). There were 63,516 DMRs identified using the Angus reference, but substantially more DEGs (2,137) were identified (S. table 10). Around 15% (~9,300) of the DMRs overlap with a significant DEG; most (72% of the 9,300) of those overlaps were in a putative enhancer region. Using the Angus reference, ~27% (~17,100) of DMRs overlapped a DEG, with most (~70%) of the DMRs overlapping a putative enhancer region. One per cent and 0.7% of DMRs overlapped with a DEG promoter when using either the Brahman or Angus as the reference.

We observed 73 imprinted genes that overlapped DMRs identified between the two different sire groups. Less than 1% (586) of DMRs overlapped the 73 imprinted genes, with most (~73% of 586) occurring in the putative enhancer region. Seven imprinted genes were significantly differentially expressed and overlapped with a DMR (Table 3). These genes were DS cell adhesion molecule (*Dscam*), 5-hydroxytryptamine receptor 2A (*Htr2a*), *Nnat*, *Dgat1*, AXL receptor tyrosine kinase (*Axl*), necdin MAGE family member (*Ndn*), and tissue factor pathway inhibitor 2 (*Tfpi2*). *Dscam*, *Nnat*, *Ndn* and *Tfpi2* had higher expression in samples with Angus sires, with the other genes being more highly expressed in samples with Brahman sires. *Slc22a18* showed high expression in the Brahman sire group but did not overlap with any DMRs. More significantly differentially expressed imprinted genes were observed using the Angus than the Brahman reference (Table 4). In this case, the ten significantly differentially expressed imprinted genes with DMR overlap were *Dscam*, *Esr2*, *Htr2a*, *Nnat*, succinate dehydrogenase complex subunit D (*Sdhd*), *Axl*, makorin ring finger protein 3 (*Mkrn3*), *Ndn*, *Dlgap2* and *Slc22a18*. *Dscam*, *Nnat*, *Sdhd*, *Mkrn3* and *Ndn* had higher expression in samples with an Angus sire. The remaining five genes (*Esr2*, *Htr2a*, *Axl*, *Dlgap2*, *Slc22a18*) had higher expression in samples with a Brahman sire. *Dgat1* did not overlap any DMRs when using the Angus reference.

Discussion

In the present study, we observed genome-wide CpG methylation correlations among replicates that ranged from 75% to 82% between groups and from 81% to 87% within groups. These correlations were similar to a recent study in mice where genome-wide CpG methylation correlations among replicates ranged from 73% to greater than 80% [61]. Moreover, we observed levels of liver global CpG methylation between 47-62% in the present study, which is similar to previous studies of human [62], mouse [61] and cattle [63].

Mapping statistics can provide an insight into how the choice of reference genome will affect downstream analyses [64]. However, we observed negligible differences in raw mapping statistics regardless of whether the Angus or Brahman reference genomes were used. Additionally, the global methylation quantification bias observed was less than 2%, depending on the reference genome used. This quantification bias is lower than the 7-9% quantification bias found in the mouse genome, depending on the reference genome used (Wulfridge, Langmead [15]. The extent of this bias is influenced by the divergence between reference genomes and whether the breed-specific CpGs tend to be hypo- or hypermethylated. Brahman and Angus have a CpG divergence of ~4%, whereas the mouse genomes analysed by Wulfridge, Langmead [15] had a CpG divergence of 10.7%. The bias we observed was greatest in the BTBT samples when the Brahman genome was used as the reference, most likely because the Angus-specific CpG sites tended to be hypermethylated. Conversely, the quantification bias was lower in the other genetic groups, possibly due to the hypomethylation in Brahman-specific CpG sites.

Spontaneous deamination of methylated CpG to TpG is the most common dinucleotide mutation in the mammalian genome [40, 41]. We observed around 800,000 C-T or G-A mutations between Brahman and Angus. A recent study observed 34,677 SNPs affecting CpG sites between indicine and taurine genomes [65]. The difference in the number of SNPs between the two studies is likely due to Capra, Lazzari [65] having used reduced representation bisulfite sequencing with substantially lower coverage than the present study and that they only considered SNPs that affected CpG sites. When differential methylation analysis is performed, a breed that has lost the C (mutated to T) will be reported as having 0% methylation at that site when, in fact, there is no CpG present. This incorrect identification of an unmethylated site can then severely impact the interpretation of results.

SVs have been associated with various traits in humans, including HIV-1 susceptibility [66], autism [67-69] and carcinogen metabolism [70]. In livestock, SVs have been implicated in diverse traits ranging from horn (polled) status [71, 72] to bulldog calf syndrome [73]. The SVs between Brahman and Angus have significantly more CpGs than the background genome, potentially introducing CpGs with important regulatory effects. However, due to their presence in only one subspecies, a single reference genome will fail to account for these breed-specific CpG sites. Therefore, the phenotypic differences between the two breeds may be influenced by CpGs that cannot be compared accurately with a single reference genome if they are in breed-specific regions.

We observed relatively few changes in methylation direction, and these were likely to be an artifact of how the genome was tiled and possible erroneous alignments in the coordinate conversion. A common step of some DMR callers is to perform window tiling of the genome to identify DMRs or to enable analysis when coverage is low [74-76]. SNPs and SVs can potentially complicate analyses when genome tiling is used to identify DMRs, as a single reference genome cannot account for these variants. Although we observed no directional changes when considering DMCs, it is possible that some DMRs were specific to the reference genome, which affected the analysis. Researchers should be careful when using genome tiling in methylation analyses that compare breeds, strains or populations.

Most DMRs identified in this study were not associated with DEGs. However, of the DEGs that overlapped with a DMR, there was a tendency for the overlap to occur more frequently in the putative enhancer region than in promoters or DEG bodies. This trend suggests that differential methylation of enhancers may impact gene expression differences in bovine fetal liver. Indeed, a growing body of evidence suggests that enhancer methylation is important in embryonic and fetal development [61, 77-79].

There were more significant DEGs when mapping to the Angus reference than the Brahman reference. Interestingly, genes that were DE using the Brahman reference were not always DE when using the Angus reference. The choice of reference genome has been shown to impact differential expression analysis in rice [80], bacteria [81] and human [82, 83] when using short-read RNA-seq. When the reference genome better represents the individuals being studied, more reads can be uniquely aligned to the correct position, providing a more accurate estimate of gene expression.

We identified several interesting DEGs associated with DMRs, particularly imprinted genes. Among these was *Dgat1,* which is involved in fat metabolism in milk production [84], feed conversion and adipogenesis [84, 85]. Several studies have investigated the role of *Dgat1* in weight gain [86, 87]; expression of *Dgat1* is necessary for weight gain, especially when the caloric density of food is high [87]. We found differential expression of *Dgat1*, with higher expression in Brahman than Angus (BIBI vs BTBT) and when Brahman was the sire (BIBI, BIBT vs BTBT, BTBI). Taken together, the breed and sire of origin comparisons suggest that the breed of the sire may be an important determinant in the expression of this gene. Higher *Dgat1* expression may result from adaptation to poor feed quality, e.g. Elzo, Riley [88] observed better feed conversion efficiency in Brahman compared to Angus and Brahman x Angus cattle. Regulation of *Dgat1* expression may occur via DNA methylation, as there is a DMR ~42kb downstream of the transcription start site, which was identified in both the breed-specific and SOE comparisons.

Parent-of-origin DMRs may change how cis-regulatory elements interact with target genes and influence gene expression in the offspring [45, 89]. It has been observed that parent-specific methylation can alter the cis-regulatory landscape around certain genes, such as *Igf*2 [90, 91]. DMRs may influence the DEGs and, ultimately, help drive the differences in phenotype. However, to confidently assign gene expression and DMRs to a particular parent, long-read sequencing [92] is needed to identify variations that link the sequences to the parent of origin. Additionally, the use of reciprocal crosses will enable one to investigate if a combination of breed and sex of the parent impacts which transcript is expressed.

SNPs and SVs have been shown to complicate and bias analyses in several studies [15, 80-83]. In our analysis, we observed an enrichment of SNPs affecting CpGs between Brahman and Angus. Capra, Lazzari [65] also reported a higher frequency of breed-specific SNPs around DMCs in a study of indicine and taurine cattle. This finding suggests that genetic differences between the two breeds may contribute to epigenetic variations. Using individual animal genomes in the study to account for genetic variations, as Wulfridge, Langmead [15] suggested, would enhance the accuracy for each individual. However, despite decreasing sequencing costs, the cost will likely be prohibitive in most livestock contexts. A possible solution was explored in a recent study comparing methylation in taurine and indicine cattle [65]. Here the authors used genotyping by sequencing to exclude SNPs affecting CpG sites from the analysis [65]. While this simplifies downstream analysis, it may also remove CpGs involved in the phenotypic differences between the two breeds, representing a limitation of the present study and that of Capra, Lazzari [65]. An alternative approach to using single reference genomes is the utilisation of pan-genomes, which encompass the majority of variations within the population [20, 93]**.** This feature is particularly important in the context of DNA methylation studies where, demonstrated in our study, SNPs at CpG sites can exert substantial effects on local methylation information.

Conclusions

This study generated a substantial WGBS dataset derived from two phenotypically diverse cattle breeds which are representative of the two cattle subspecies and highlighted the importance of reference genome choice in methylation analyses. Our findings suggest that the DMRs may primarily exert their influence on enhancer elements rather than promoters. We also identified 11 genes that might be under DMR control. The results underscore the advantages of using the appropriate reference genome for the data set and provide additional evidence supporting the incorporation of genome graphs to improve analyses of populations with high genetic divergence.

Methods

Study Animals and Sample Collection

All animal experiments and procedures described in this study complied with Australian guidelines, approved by the University of Adelaide Animal Ethics Committee and followed the ARRIVE Guidelines (https://arriveguidelines.org/) (Approval No. S-094-2005). Liver tissue samples from concepti were the same as those described in Liu, Tearle [94]. Briefly, the parents were purebred Angus (*B. t. taurus*) and purebred Brahman (*B. t. indicus*), herein denoted as BT and BI. Primiparous females and their fetuses were ethically sacrificed at day 153±1 of gestation. Concepti were dissected, and tissue samples snap-frozen in liquid nitrogen and stored at -80°C until further use. Liver samples from three female and three male individuals from each of the four genetic combinations: BT x BT, BT x BI, BI x BT, and BI x BI were used.

DNA extraction and sequencing

DNA was extracted from frozen fetal liver tissues using Qiagen® DNeasy® Blood & Tissue Kit following the manufacturer's instruction and sent to BGI Hong Kong, China, for WGBS library preparation and sequencing. Bisulfite conversion was performed using the Zymo Research™ EZ DNA Methylation™ - Gold Kit (D5005). All samples were sequenced in a single batch, and each sample was sequenced to ~30X coverage using the BGI DNB-seq.

RNA was extracted from frozen fetal liver tissues using Illumina® RiboZero Gold kits following the manufacturer's instruction and prepared for Illumina RNA-seq short-read sequencing. The RNA-seq protocol and data availability (GEO accession number: GSE148909) have been described in our previous work [94].

The same tissue samples were used in both the RNA-seq and WGBS. Individual sample names and their corresponding genetic group are given in Supplementary Table 11.

WGBS mapping

WGBS reads were mapped using the MethylSeq Nextflow pipeline (v. 1.6.1) [95] with the '--zymo' trimming parameter. The reads were first checked for quality with FastQC (v. 0.11.9) (https://www.bioinformatics.babraham.ac.uk/projects/fastqc/), then adapters were trimmed using Trim Galore (v. 0.6.6) (https://github.com/FelixKrueger/TrimGalore), and the reads were reassessed for quality post-trimming. Trimmed reads passing qvalue ≥ 20 were mapped to the Brahman (GCA_003369695.2) and

Angus (GCA_003369685.2) genomes [39] using BWA-Meth (0.2.2) (arXiv:1401.1129). The non-pseudo autosomal region of the Angus Y chromosome was added to the Brahman reference. This step enabled us to include the Y chromosome sequence whilst avoiding duplication of the pseudoautosomal region. Both Brahman and Angus chromosome sequences were reorientated to match the orientation of ARS-UCD1.2 chromosomes [96]. After sorting the alignment files with SAMtools (v. 1.11) [97], duplicates were marked with Picard (v. 2.25.4) (https://broadinstitute.github.io/picard/). Bam file quality control was performed with the bamqc function from qualimap (v. 2.2.2d) [98] by setting the '-gd' parameter to HUMAN. Methylation calls were extracted using MethylDackel (v. 0.5.2) (https://github.com/dpryan79/MethylDackel) extract with the parameter '—minDepth 10' and output in MethylKit [74] format ('—methylKit') and a more generic cytosine report ('—cytosine_report'). In-house scripts were used to convert the MethylDackel output for use with DNMTools https://dnmtools.readthedocs.io/en/latest/ (see https://github.com/DaviesCentreInformatics/Brahman_Angus_WGBS). All samples had a bisulfite conversion efficiency of >99%. All downstream analyses only used CpGs from autosomes with $\geq$ 10X coverage.

Identification of shared and breed-specific CpG sites

For a given autosome, we extracted 1000bp around all CpGs that were not in the first 500bp or last 500bp of the chromosome; this yielded sequences that were 1002bp long. We then mapped the 1002bp CpG sequences from one subspecies to the reference of the other. We used minimap2 (v. 2.24) [99] with the 'map-hifi' preset to align CpGs from a given chromosome in one breed to the same chromosome in the other breed; alignments were sorted using SAMtools (v 1.11) [97]. Once the long sequences were aligned, we filtered the BAM file and considered all alignments where at least 900 bp were successfully aligned to the reference. We then used the Align package from BioPython (v 1.80) [100] to perform a local alignment between the 102bp sequences taken from the midpoint of the query and reference. We then recorded which CpG sites were shared between Brahman and Angus, which CpG sites differed, and which could not be aligned during the initial minimap2 alignment step (S. table 6). We performed subsequent analyses using all CpGs present on the autosomes for each reference and again using only the shared CpGs that passed the ≥ 10X coverage criteria. The CpG sites that could not be aligned in the initial alignment step with minimap2 (i.e. a genomic region that is present in one breed but missing in the other breed) or constituted a SNP were considered breed-specific CpG sites. All steps described in this section were performed for both Brahman and Angus reference genomes.

Identification of differentially methylated regions

The methylKit package (v. 1.22.0) [74] was used to identify DMRs between breed and POE groups. We investigated breed effects by comparing BIBI samples with BTBT samples. The reference group was always the breed that matched the reference genome. i.e., BIBI samples were the reference group when reads were aligned to the Brahman reference. Any DMRs identified were either hypo- or hypermethylated with respect to this reference group. To study POEs, we investigated the maternal effects by comparing samples with BIBI dams (BIBI; BTBI) and those with BTBT dams (BTBT; BIBT). Similarly, to study the paternal effects, we compared those with BIBI sires (BIBI; BIBT) and those with BTBT sires (BTBT; BTBI). For each comparison, we removed all CpG sites with less than 10X coverage and more than the 99.9th percentile of coverage. Reads with too high coverage (e.g. from PCR duplication bias) can impair the accurate determination of the methylation percentage at that site [74]. We then normalized the coverage using the default methylKit normalization strategy. We merged the CpG counts per group using the 'unite' function with 'destrand = T' and 'min.per.group = 5L' so that a given CpG site had to be covered by at least ten reads in five out of six samples per group. For the parent of origin DMR analysis, we set 'min.per.group = 10L'.

We then identified differentially methylated cytosines between groups using the 'calculateDiffMeth' function, with sex as a covariate in the model. To determine differentially methylated regions, we used the 'tileMethylCounts' function with default parameters to divide the genome into regions for differential methylation analysis. This step allowed methylKit to divide the genome into non-overlapping regions based on the tiling windows. Briefly, methylKit models the methylation at a given cytosine or region by fitting a logistic regression:

$$\log\left(\frac{P_i}{1-P_i}\right) = \beta_0 + \beta_1 * T_i$$

P_i denotes the methylation proportion for sample i in samples 1,…,*n*, where *n* is the number of samples across both groups in the comparison [74]. T_i represents the groups (0 for group 1, 1 for group 2). β_0 denotes the log odds of group 1 (fraction of reads reporting C / 1 – the fraction of reads reporting C). β_1 denotes the log odds ratio between the groups. For further details, refer to Akalin, Kormaksson [74]. We retained all DMRs with a difference in methylation of ≥ 10% and a qvalue of ≤ 0.01 for further analysis. An overview of the samples, reference genomes, types of CpGs and DMR analysis is given in Fig 1A-G.

Identification of SNPs and SVs between genomes

We used MUMmer (v. 4.0.0) [101] to identify SNPs and generate input files for Assemblytics (v. 1.2.1) [102]. Briefly, 'dnadiff' from MUMmer with default parameters was used to align the Brahman and Angus autosomes. We extracted SNPs for all autosomes from the '.snps' file generated by 'dnadiff'. The delta file generated in this step was used as input to Assemblytics. The output from Assemblytics was then used to identify SVs.

DMR coordinate conversion

To determine if a given DMR changed methylation direction between genomes, we had to convert the coordinates of DMRs identified by alignment with the Angus genome to Brahman coordinates and vice versa. We considered a DMR as changing methylation direction if, for example, it is hypomethylated in BIBI samples compared to BTBT samples when using the Brahman reference but becomes hypermethylated in BIBI using the Angus reference genome. To investigate this, we first converted the DMR bed files to GTF files and then used Liftoff (v.1.6.2) [103] to transfer coordinates from one reference genome to the other. We then identified DMRs reciprocally overlapping one another by at least 90% between the two genomes, with these DMRs being considered successfully lifted over. DMRs that did not overlap by 90% were not considered for the methylation direction change analysis.

RNA-seq mapping and pre-processing

RNA-seq reads were mapped to the Brahman and Angus genomes as in the WGBS mapping step. Briefly, reads were checked for quality using FastQC (v. 0.11.4) (https://www.bioinformatics.babraham.ac.uk/projects/fastqc/) before being trimmed with Trim Galore (v. 0.4.2) (https://github.com/FelixKrueger/TrimGalore) with the parameters '--quality 10' and '--length 100'. Reads were mapped using HiSAT2 (v. 2.1.0) to both the Brahman and Angus reference genomes [39]; alignment files were sorted using SAMtools (v. 1.10) [97]. FeatureCount from the Rsubread package (v. 2.10.5) [104] was used to count how many reads mapped to genes.

Differential gene expression

Differential gene expression analysis was performed using an in-house R script similar to previously published work [94], with the edgeR (v. 3.38.4) [105] and limma (v. 3.52.4) [106] R packages. The genome annotation was based on Ensembl v.104 for Brahman and Angus. The orientation of the genes was reversed where necessary to correspond with the orientation of the chromosomes of ARS-UCD1.2. Briefly, genes that had fewer than 0.5 counts per million (CPM) in fewer than three samples were removed from the dataset. Reads were normalized using the trimmed mean of M-values and then weighted using ‘voomWithQualityWeights’. As two samples were sequenced in a separate batch, we added the batch as a term to the model to account for any variability introduced by the separate sequencing run. We then compared differential gene expression between purebred Angus and Brahman, Angus dams and Brahman dams, and Angus sires and Brahman sires. Genes with significant differences in gene expression at an adjusted p-value ≤ 0.05 were retained for further analysis.

Identifying imprinted genes

We downloaded a list of genes with evidence of imprinting in human, mouse and cattle from Morison, Ramsay [107] and https://www.geneimprint.org. We then used OrthoFinder to identify human orthologs of both Brahman and Angus genes [108], allowing us to assign Human Genome Organisation Gene Nomenclature Committee (HGNC) symbols to genes in each breed. To do this, we first identified which Brahman proteins had orthologs in human. We then identified the genes that encoded these proteins and used this information to assign human and Brahman genes as orthologs. We repeated the process for the Angus genes. We then identified all genes that could be assigned an HGNC symbol from the Brahman Ensembl annotation version 104 that were also present in the imprinted gene list (S. table 12). This filtering gave us 80 imprinted genes for Brahman autosomes. We repeated the process for Angus using the Angus Ensembl annotation version 104 and identified 79 imprinted genes. The discrepancy is due to one imprinted gene for Angus occurring on an unplaced scaffold.

Linking DMRs to DEGs

For each DEG, we considered five different regions in and around the gene where DMRs might have an influence. These regions included putative enhancer regions, 5kb outside the gene body, and the gene body itself (S. figure 11). The upstream putative enhancer region started 130kb upstream of the gene and then stopped 5kb upstream of the gene body for a total length of 125kb. We repeated this for the downstream putative enhancer region, starting 5kb downstream of the gene body and extending out 125kb. This number was based on the median distance between enhancers and their gene targets [109]. The 5kb region was from upstream of the start of the gene body to the start of the gene body. Again, this was repeated for the downstream 5kb region. The gene body was the region annotated as "gene" in the Ensembl annotation file. We then found all DMRs that overlapped these regions by at least 90% of their length using 'bedtools intersect' with the '-f' and '-F' arguments, both set at 0.9 and the '-e' argument set to True.

Declarations

Ethics approval

All animal experiments and procedures described in this study complied with Australian guidelines, approved by the University of Adelaide Animal Ethics Committee and followed the ARRIVE Guidelines (https://arriveguidelines.org/) (Approval No. S-094-2005).

Consent for publication

Not applicable

Availability of data and materials

The datasets generated and analysed during the current study are available in the NCBI SRA repository under BioProject: PRJNA626458. Code used to analyse the data is available at: https://github.com/DaviesCentreInformatics/Brahman_Angus_WGBS.

Competing interests

The authors declare that they have no competing interests.

Funding

The study was funded by the JS Davies bequest through the Davies Livestock Research Centre.

Authors' contributions

WYL, JLW and SH conceived and managed the project. SH designed and obtained *Bos taurus* and *Bos indicus* fetal resources. TC extracted WGBS samples and performed QC. CM performed all analyses; CM, SH, WYL and HAR interpreted data. CM and WYL drafted the manuscript, and all authors read, edited and approved the final manuscript.

Acknowledgements

We thank Yan Ren for uploading and performing the initial QC of WGBS data on the University of Adelaide's Phoenix HPC.

Supplementary information

Additional file 1. Supplementary figures referred to in the main text.

Additional file 2. WGBS mapping statistics.

Additional file 3. Table describing the number of CpGs in each reference and what percentage of those have 10X coverage.

Additional file 4. Mean global CpG coverage.

Additional file 5. Global CpG methylation.

Additional file 6. Methylation of CpGs in different genomic regions.

Additional file 7. Alignment statistics of cross-reference genome CpG mapping

Additional file 8. SNP and SV enrichment at CpG sites.

Additional file 9. Table of differentially methylated regions that exhibited direction changes.

Additional file 10. Number of DMRs in each comparison.

Additional file 11. Number of up and down-regulated genes in each comparison.

Additional file 12. Sample information.

Additional file 13. List of imprinted genes.

References

1. Jansz N. DNA methylation dynamics at transposable elements in mammals. Essays in Biochemistry. 2019;63 6:677-89. doi:10.1042/ebc20190039.

2. Li E and Zhang Y. DNA methylation in mammals. Cold Spring Harb Perspect Biol. 2014;6 5:a019133. doi:10.1101/cshperspect.a019133.

3. Ramsahoye BH, Biniszkiewicz D, Lyko F, Clark V, Bird AP and Jaenisch R. Non-CpG methylation is prevalent in embryonic stem cells and may be mediated by DNA methyltransferase 3a. Proceedings of the National Academy of Sciences. 2000;97 10:5237-42. doi:doi:10.1073/pnas.97.10.5237.

4. Ziller MJ, Müller F, Liao J, Zhang Y, Gu H, Bock C, et al. Genomic Distribution and Inter-Sample Variation of Non-CpG Methylation across Human Cell Types. PLoS Genet. 2011;7 12:e1002389. doi:10.1371/journal.pgen.1002389.

5. Moore LD, Le T and Fan G. DNA Methylation and Its Basic Function. Neuropsychopharmacology. 2013;38 1:23-38. doi:10.1038/npp.2012.112.

6. Kass SU, Landsberger N and Wolffe AP. DNA methylation directs a time-dependent repression of transcription initiation. Curr Biol. 1997;7 3:157-65. doi:10.1016/s0960-9822(97)70086-1.

7. Smith J, Sen S, Weeks RJ, Eccles MR and Chatterjee A. Promoter DNA Hypermethylation and Paradoxical Gene Activation. Trends in Cancer. 2020;6 5:392-406. doi:https://doi.org/10.1016/j.trecan.2020.02.007.

8. Cho J-W, Shim HS, Lee CY, Park SY, Hong MH, Lee I, et al. The importance of enhancer methylation for epigenetic regulation of tumorigenesis in squamous lung cancer. Experimental & Molecular Medicine. 2022;54 1:12-22. doi:10.1038/s12276-021-00718-4.

9. Spainhour JCG, Lim HS, Yi SV and Qiu P. Correlation Patterns Between DNA Methylation and Gene Expression in The Cancer Genome Atlas. Cancer Informatics. 2019;18 doi:10.1177/1176935119828776.

10. Charlet J, Duymich Christopher E, Lay Fides D, Mundbjerg K, Dalsgaard Sørensen K, Liang G, et al. Bivalent Regions of Cytosine Methylation and H3K27 Acetylation Suggest an Active Role for DNA Methylation at Enhancers. Mol Cell. 2016;62 3:422-31. doi:10.1016/j.molcel.2016.03.033.

11. Creyghton MP, Cheng AW, Welstead GG, Kooistra T, Carey BW, Steine EJ, et al. Histone H3K27ac separates active from poised enhancers and predicts developmental state. Proceedings of the National Academy of Sciences. 2010;107 50:21931-6. doi:10.1073/pnas.1016071107.

12. Kang Y, Kim YW, Kang J and Kim A. Histone H3K4me1 and H3K27ac play roles in nucleosome eviction and eRNA transcription, respectively, at enhancers. The FASEB Journal. 2021;35 8 doi:10.1096/fj.202100488r.

13. Wang M, Hancock TP, MacLeod IM, Pryce JE, Cocks BG and Hayes BJ. Putative enhancer sites in the bovine genome are enriched with variants affecting complex traits. Genet Sel Evol. 2017;49 1:56. doi:10.1186/s12711-017-0331-4.

14. Zhu Y, Zhou Z, Huang T, Zhang Z, Li W, Ling Z, et al. Mapping and analysis of a spatiotemporal H3K27ac and gene expression spectrum in pigs. Sci China Life Sci. 2022;65 8:1517-34. doi:10.1007/s11427-021-2034-5.

15. Wulfridge P, Langmead B, Feinberg AP and Hansen KD. Analyzing whole genome bisulfite sequencing data from highly divergent genotypes. Nucleic Acids Res. 2019;47 19:e117-e. doi:10.1093/nar/gkz674.

16. Brandt DYC, Aguiar VRC, Bitarello BD, Nunes K, Goudet J and Meyer D. Mapping Bias Overestimates Reference Allele Frequencies at the HLA Genes in the 1000 Genomes Project Phase I Data. G3 Genes|Genomes|Genetics. 2015;5 5:931-41. doi:10.1534/g3.114.015784.

17. Degner JF, Marioni JC, Pai AA, Pickrell JK, Nkadori E, Gilad Y, et al. Effect of read-mapping biases on detecting allele-specific expression from RNA-sequencing data. Bioinformatics. 2009;25 24:3207-12. doi:10.1093/bioinformatics/btp579.

18. Salavati M, Bush SJ, Palma-Vera S, McCulloch MEB, Hume DA and Clark EL. Elimination of Reference Mapping Bias Reveals Robust Immune Related Allele-Specific Expression in Crossbred Sheep. Frontiers in Genetics. 2019;10 doi:10.3389/fgene.2019.00863.

19. Chen N-C, Solomon B, Mun T, Iyer S and Langmead B. Reference flow: reducing reference bias using multiple population genomes. Genome Biology. 2021;22 1:8. doi:10.1186/s13059-020-02229-3.

20. Groza C, Kwan T, Soranzo N, Pastinen T and Bourque G. Personalized and graph genomes reveal missing signal in epigenomic data. Genome Biology. 2020;21 1:124. doi:10.1186/s13059-020-02038-8.

21. Crysnanto D and Pausch H. Bovine breed-specific augmented reference graphs facilitate accurate sequence read mapping and unbiased variant discovery. Genome Biology. 2020;21 1:184. doi:10.1186/s13059-020-02105-0.

22. Lloret-Villas A, Bhati M, Kadri NK, Fries R and Pausch H. Investigating the impact of reference assembly choice on genomic analyses in a cattle breed. BMC Genomics. 2021;22 1 doi:10.1186/s12864-021-07554-w.

23. Günther T and Nettelblad C. The presence and impact of reference bias on population genomic studies of prehistoric human populations. PLoS Genet. 2019;15 7:e1008302. doi:10.1371/journal.pgen.1008302.

24. Wang T, Antonacci-Fulton L, Howe K, Lawson HA, Lucas JK, Phillippy AM, et al. The Human Pangenome Project: a global resource to map genomic diversity. Nature. 2022;604 7906:437-46. doi:10.1038/s41586-022-04601-8.

25. Liao W-W, Asri M, Ebler J, Doerr D, Haukness M, Hickey G, et al. A draft human pangenome reference. Nature. 2023;617 7960:312-24. doi:10.1038/s41586-023-05896-x.

26. Smith TPL, Bickhart DM, Boichard D, Chamberlain AJ, Djikeng A, Jiang Y, et al. The Bovine Pangenome Consortium: democratizing production and accessibility of genome assemblies for global cattle breeds and other bovine species. Genome Biology. 2023;24 1:139. doi:10.1186/s13059-023-02975-0.

27. Woodhouse MR, Cannon EK, Portwood JL, Harper LC, Gardiner JM, Schaeffer ML, et al. A pan-genomic approach to genome databases using maize as a model system. BMC Plant Biol. 2021;21 1:385. doi:10.1186/s12870-021-03173-5.

28. McTavish EJ, Decker JE, Schnabel RD, Taylor JF and Hillis DM. New World cattle show ancestry from multiple independent domestication events. Proceedings of the National Academy of Sciences. 2013;110 15:E1398-E406. doi:doi:10.1073/pnas.1303367110.

29. Bruford MW, Bradley DG and Luikart G. DNA markers reveal the complexity of livestock domestication. Nature Reviews Genetics. 2003;4 11:900-10. doi:10.1038/nrg1203.

30. Ajmone-Marsan P, Garcia JF and Lenstra JA. On the origin of cattle: How aurochs became cattle and colonized the world. Evolutionary Anthropology: Issues, News, and Reviews. 2010;19 4:148-57. doi:https://doi.org/10.1002/evan.20267.

31. MacHugh DE, Larson G and Orlando L. Taming the Past: Ancient DNA and the Study of Animal Domestication. Annual Review of Animal Biosciences. 2017;5 1:329-51. doi:10.1146/annurev-animal-022516-022747.

32. Loftus RT, MacHugh DE, Bradley DG, Sharp PM and Cunningham P. Evidence for two independent domestications of cattle. Proceedings of the National Academy of Sciences. 1994;91 7:2757-61. doi:doi:10.1073/pnas.91.7.2757.

33. Elzo MA, Johnson DD, Wasdin JG and Driver JD. Carcass and meat palatability breed differences and heterosis effects in an Angus–Brahman multibreed population. Meat Science. 2012;90 1:87-92. doi:https://doi.org/10.1016/j.meatsci.2011.06.010.

34. Dikmen S, Mateescu RG, Elzo MA and Hansen PJ. Determination of the optimum contribution of Brahman genetics in an Angus-Brahman multibreed herd for regulation of body temperature during hot weather. J Anim Sci. 2018;96 6:2175-83. doi:10.1093/jas/sky133.

35. Goszczynski DE, Corbi-Botto CM, Durand HM, Rogberg-Muñoz A, Munilla S, Peral-Garcia P, et al. Evidence of positive selection towards Zebuine haplotypes in the BoLA region of Brangus cattle. Animal. 2018;12 2:215-23. doi:https://doi.org/10.1017/S1751731117001380.

36. Koren S, Rhie A, Walenz BP, Dilthey AT, Bickhart DM, Kingan SB, et al. De novo assembly of haplotype-resolved genomes with trio binning. Nat Biotechnol. 2018;36 12:1174-82. doi:10.1038/nbt.4277.

37. Decker JE, Mckay SD, Rolf MM, Kim J, Molina Alcalá A, Sonstegard TS, et al. Worldwide Patterns of Ancestry, Divergence, and Admixture in Domesticated Cattle. PLoS Genet. 2014;10 3:e1004254. doi:10.1371/journal.pgen.1004254.

38. Hiendleder S, Lewalski H and Janke A. Complete mitochondrial genomes of Bos taurus and Bos indicus provide new insights into intra-species variation, taxonomy and domestication. Cytogenet Genome Res. 2008;120 1-2:150-6. doi:10.1159/000118756.

39. Low WY, Tearle R, Liu R, Koren S, Rhie A, Bickhart DM, et al. Haplotype-resolved genomes provide insights into structural variation and gene content in Angus and Brahman cattle. Nature Communications. 2020;11 1:1-14.

40. Yang J, Horton JR, Akdemir KC, Li J, Huang Y, Kumar J, et al. Preferential CEBP binding to T:G mismatches and increased C-to-T human somatic mutations. Nucleic Acids Res. 2021;49 9:5084-94. doi:10.1093/nar/gkab276.

41. Żemojtel T, Kiełbasa SM, Arndt PF, Behrens S, Bourque G and Vingron M. CpG Deamination Creates Transcription Factor–Binding Sites with High Efficiency. Genome Biology and Evolution. 2011;3 0:1304-11. doi:10.1093/gbe/evr107.

42. Van Laere A-S, Nguyen M, Braunschweig M, Nezer C, Collette C, Moreau L, et al. A regulatory mutation in IGF2 causes a major QTL effect on muscle growth in the pig. Nature. 2003;425 6960:832-6. doi:10.1038/nature02064.

43. Zhang YQ, Yang LX, Kucherlapati M, Hadjipanayis A, Pantazi A, Bristow CA, et al. Global impact of somatic structural variation on the DNA methylome of human cancers. Genome Biology. 2019;20 1 doi:10.1186/s13059-019-1818-9.

44. Lutz PE, Almeid D, Belzeaux R, Yalcin I and Turecki G. Epigenetic regulation of the kappa opioid receptor gene by an insertion-deletion in the promoter region. European Neuropsychopharmacology. 2018;28 2:334-40. doi:10.1016/j.euroneuro.2017.12.013.

45. Lawson HA, Cheverud JM and Wolf JB. Genomic imprinting and parent-of-origin effects on complex traits. Nat Rev Genet. 2013;14 9:609-17. doi:10.1038/nrg3543.

46. Shi W, Krella A, Orth A, Yu Y and Fundele R. Widespread disruption of genomic imprinting in adult interspecies mouse (Mus) hybrids. Genesis. 2005;43 3:100-8. doi:10.1002/gene.20161.

47. Vaughn RN, Kochan KJ, Torres AK, Du M, Riley DG, Gill CA, et al. Skeletal Muscle Expression of Actinin-3 (ACTN3) in Relation to Feed Efficiency Phenotype of F-2 Bos indicus-Bos taurus Steers. Frontiers in Genetics. 2022;13 doi:10.3389/fgene.2022.796038.

48. Pan ZX, Zhang JL, Zhang JB, Zhou B, Chen J, Jiang ZH, et al. Expression Profiles of the Insulin-like Growth Factor System Components in Liver Tissue during Embryonic and Postnatal Growth of Erhualian and Yorkshire Reciprocal Cross F-1 Pigs. Asian-Australasian Journal of Animal Sciences. 2012;25 7:903-12. doi:10.5713/ajas.2011.11385.

49. Moore GE, Ishida M, Demetriou C, Al-Olabi L, Leon LJ, Thomas AC, et al. The role and interaction of imprinted genes in human fetal growth. Philos Trans R Soc Lond B Biol Sci. 2015;370 1663:20140074. doi:10.1098/rstb.2014.0074.

50. Eggermann T, Davies JH, Tauber M, van den Akker E, Hokken-Koelega A, Johansson G, et al. Growth Restriction and Genomic Imprinting-Overlapping Phenotypes Support the Concept of an Imprinting Network. Genes. 2021;12 4 doi:10.3390/genes12040585.

51. Yuen RKC, Jiang R, Penaherrera MS, McFadden DE and Robinson WP. Genome-wide mapping of imprinted differentially methylated regions by DNA methylation profiling of human placentas from triploidies. Epigenetics & Chromatin. 2011;4 doi:10.1186/1756-8935-4-10.

52. Doria S, Sousa M, Fernandes S, Ramalho C, Brandao O, Matias A, et al. Gene expression pattern of IGF2, PHLDA2, PEG10 and CDKN1C imprinted genes in spontaneous miscarriages or fetal deaths. Epigenetics. 2010;5 5:444-50. doi:10.4161/epi.5.5.12118.

53. Piedrahita JA. The Role of Imprinted Genes in Fetal Growth Abnormalities. Birth Defects Research Part a-Clinical and Molecular Teratology. 2011;91 8:682-92. doi:10.1002/bdra.20795.

54. Amat S, Dahlen CR, Swanson KC, Ward AK, Reynolds LP and Caton JS. Bovine Animal Model for Studying the Maternal Microbiome, in utero Microbial Colonization and Their Role in Offspring Development and Fetal Programming. Front Microbiol. 2022;13:854453. doi:10.3389/fmicb.2022.854453.

55. Peruffo A and Cozzi B. Bovine Brain: An in vitro Translational Model in Developmental Neuroscience and Neurodegenerative Research. Front Pediatr. 2014;2:74. doi:10.3389/fped.2014.00074.

56. Chen Z, Robbins KM, Wells KD and Rivera RM. Large offspring syndrome: a bovine model for the human loss-of-imprinting overgrowth syndrome Beckwith-Wiedemann. Epigenetics. 2013;8 6:591-601.

57. Malhi PS, Adams GP and Singh J. Bovine Model for the Study of Reproductive Aging in Women: Follicular, Luteal, and Endocrine Characteristics1. Biol Reprod. 2005;73 1:45-53. doi:10.1095/biolreprod.104.038745.

58. MacPhillamy C, Alinejad-Rokny H, Pitchford WS and Low WY. Cross-species enhancer prediction using machine learning. Genomics. 2022;114 5:110454. doi:10.1016/j.ygeno.2022.110454.

59. Leenen FAD, Muller CP and Turner JD. DNA methylation: conducting the orchestra from exposure to phenotype? Clinical Epigenetics. 2016;8 1:92. doi:10.1186/s13148-016-0256-8.

60. Thomson K, Game J, Karouta C, Morgan IG and Ashby R. Correlation between small-scale methylation changes and gene expression during the development of myopia. The FASEB Journal. 2022;36 1:e22129. doi:https://doi.org/10.1096/fj.202101487R.

61. He Y, Hariharan M, Gorkin DU, Dickel DE, Luo C, Castanon RG, et al. Spatiotemporal DNA methylome dynamics of the developing mouse fetus. Nature. 2020;583 7818:752-9. doi:10.1038/s41586-020-2119-x.

62. Hama N, Totoki Y, Miura F, Tatsuno K, Saito-Adachi M, Nakamura H, et al. Epigenetic landscape influences the liver cancer genome architecture. Nature Communications. 2018;9 doi:10.1038/s41467-018-03999-y.

63. Zhou Y, Liu S, Hu Y, Fang L, Gao Y, Xia H, et al. Comparative whole genome DNA methylation profiling across cattle tissues reveals global and tissue-specific methylation patterns. BMC Biol. 2020;18 1:85. doi:10.1186/s12915-020-00793-5.

64. Valiente-Mullor C, Beamud B, Ansari I, Francés-Cuesta C, García-González N, Mejía L, et al. One is not enough: On the effects of reference genome for the mapping and subsequent analyses of short-reads. PLoS Comp Biol. 2021;17 1:e1008678. doi:10.1371/journal.pcbi.1008678.

65. Capra E, Lazzari B, Milanesi M, Nogueira GP, Garcia Jf, Utsunomiya YT, et al. Comparison between indicine and taurine cattle DNA methylation reveals epigenetic variation associated to differences in morphological adaptive traits. Epigenetics. 2023;18 1:2163363. doi:10.1080/15592294.2022.2163363.

66. Gonzalez E, Kulkarni H, Bolivar H, Mangano A, Sanchez R, Catano G, et al. The influence of CCL3L1 gene-containing segmental duplications on HIV-1/AIDS susceptibility. Science. 2005;307 5714:1434-40. doi:10.1126/science.1101160.

67. Marshall CR, Noor A, Vincent JB, Lionel AC, Feuk L, Skaug J, et al. Structural variation of chromosomes in autism spectrum disorder. Am J Hum Genet. 2008;82 2:477-88. doi:10.1016/j.ajhg.2007.12.009.

68. Kumar RA, KaraMohamed S, Sudi J, Conrad DF, Brune C, Badner JA, et al. Recurrent 16p11.2 microdeletions in autism. Hum Mol Genet. 2008;17 4:628-38. doi:10.1093/hmg/ddm376.

69. Weiss LA, Shen Y, Korn JM, Arking DE, Miller DT, Fossdal R, et al. Association between microdeletion and microduplication at 16p11.2 and autism. N Engl J Med. 2008;358 7:667-75. doi:10.1056/NEJMoa075974.

70. Bell DA, Taylor JA, Paulson DF, Robertson CN, Mohler JL and Lucier GW. Genetic risk and carcinogen exposure: a common inherited defect of the carcinogen-metabolism gene glutathione S-transferase M1 (GSTM1) that increases susceptibility to bladder cancer. J Natl Cancer Inst. 1993;85 14:1159-64. doi:10.1093/jnci/85.14.1159.

71. Lamb HJ, Ross EM, Nguyen LT, Lyons RE, Moore SS and Hayes BJ. Characterization of the poll allele in Brahman cattle using long-read Oxford Nanopore sequencing. J Anim Sci. 2020;98 5 doi:10.1093/jas/skaa127.

72. Rothammer S, Capitan A, Mullaart E, Seichter D, Russ I and Medugorac I. The 80-kb DNA duplication on BTA1 is the only remaining candidate mutation for the polled phenotype of Friesian origin. Genet Sel Evol. 2014;46 1:44. doi:10.1186/1297-9686-46-44.

73. Jacinto JGP, Häfliger IM, Letko A, Drögemüller C and Agerholm JS. A large deletion in the COL2A1 gene expands the spectrum of pathogenic variants causing bulldog calf syndrome in cattle. Acta Vet Scand. 2020;62 1:49. doi:10.1186/s13028-020-00548-w.

74. Akalin A, Kormaksson M, Li S, Garrett-Bakelman FE, Figueroa ME, Melnick A, et al. methylKit: a comprehensive R package for the analysis of genome-wide DNA methylation profiles. Genome Biology. 2012;13 10:R87. doi:10.1186/gb-2012-13-10-r87.

75. Kishore K, de Pretis S, Lister R, Morelli MJ, Bianchi V, Amati B, et al. methylPipe and compEpiTools: a suite of R packages for the integrative analysis of epigenomics data. BMC Bioinformatics. 2015;16 1:313. doi:10.1186/s12859-015-0742-6.

76. Park Y, Figueroa ME, Rozek LS and Sartor MA. MethylSig: a whole genome DNA methylation analysis pipeline. Bioinformatics. 2014;30 17:2414-22. doi:10.1093/bioinformatics/btu339.

77. Lee HJ, Lowdon RF, Maricque B, Zhang B, Stevens M, Li D, et al. Developmental enhancers revealed by extensive DNA methylome maps of zebrafish early embryos. Nature Communications. 2015;6 1:6315. doi:10.1038/ncomms7315.

78. Alajem A, Roth H, Ratgauzer S, Bavli D, Motzik A, Lahav S, et al. DNA methylation patterns expose variations in enhancer-chromatin modifications during embryonic stem cell differentiation. PLoS Genet. 2021;17 4:e1009498. doi:10.1371/journal.pgen.1009498.

79. Slieker RC, Roost MS, van Iperen L, Suchiman HE, Tobi EW, Carlotti F, et al. DNA Methylation Landscapes of Human Fetal Development. PLoS Genet. 2015;11 10:e1005583. doi:10.1371/journal.pgen.1005583.

80. Slabaugh E, Desai JS, Sartor RC, Lawas LMF, Jagadish SVK and Doherty CJ. Analysis of differential gene expression and alternative splicing is significantly influenced by choice of reference genome. RNA. 2019;25 6:669-84. doi:10.1261/rna.070227.118.

81. Price A and Gibas C. The quantitative impact of read mapping to non-native reference genomes in comparative RNA-Seq studies. PLOS ONE. 2017;12 7:e0180904. doi:10.1371/journal.pone.0180904.

82. Kaminow B, Ballouz S, Gillis J and Dobin A. Pan-human consensus genome significantly improves the accuracy of RNA-seq analyses. Genome Res. 2022;32 4:738-49. doi:10.1101/gr.275613.121.

83. Wu P-Y, Phan JH and Wang MD. Assessing the impact of human genome annotation choice on RNA-seq expression estimates. BMC Bioinformatics. 2013;14 11:S8. doi:10.1186/1471-2105-14-S11-S8.

84. Khan MZ, Ma Y, Ma J, Xiao J, Liu Y, Liu S, et al. Association of DGAT1 With Cattle, Buffalo, Goat, and Sheep Milk and Meat Production Traits. Frontiers in Veterinary Science. 2021;8 doi:10.3389/fvets.2021.712470.

85. Abeel T, Van de Peer Y and Saeys Y. Toward a gold standard for promoter prediction evaluation. Bioinformatics. 2009;25 12:I313-I20. doi:10.1093/bioinformatics/btp191.

86. Tsuda N, Kumadaki S, Higashi C, Ozawa M, Shinozaki M, Kato Y, et al. Intestine-Targeted DGAT1 Inhibition Improves Obesity and Insulin Resistance without Skin Aberrations in Mice. PLOS ONE. 2014;9 11:e112027. doi:10.1371/journal.pone.0112027.

87. Zhang XD, Yan JW, Yan GR, Sun XY, Ji J, Li YM, et al. Pharmacological inhibition of diacylglycerol acyltransferase 1 reduces body weight gain, hyperlipidemia, and hepatic steatosis in db/db mice. Acta Pharmacol Sin. 2010;31 11:1470-7. doi:10.1038/aps.2010.104.

88. Elzo MA, Riley DG, Hansen GR, Johnson DD, Myer RO, Coleman SW, et al. Effect of breed composition on phenotypic residual feed intake and growth in Angus, Brahman, and Angus x Brahman crossbred cattle. J Anim Sci. 2009;87 12:3877-86. doi:10.2527/jas.2008-1553.

89. Giannoukakis N, Deal C, Paquette J, Goodyer CG and Polychronakos C. Parental genomic imprinting of the human IGF2 gene. Nat Genet. 1993;4 1:98-101.

90. Szabo PE, Tang SHE, Rentsendorj A, Pfeifer GP and Mann JR. Maternal-specific footprints at putative CTCF sites in the H19 imprinting control region give evidence for insulator function. Curr Biol. 2000;10 10:607-10. doi:10.1016/s0960-9822(00)00489-9.

91. Yang YW, Hu JF, Ulaner GA, Li T, Yao XM, Vu TH, et al. Epigenetic regulation of Igf2/H19 imprinting at CTCF insulator binding sites. J Cell Biochem. 2003;90 5:1038-55. doi:10.1002/jcb.10684.

92. Ren Y, Tseng E, Smith TPL, Hiendleder S, Williams JL and Low WY. Long read isoform sequencing reveals hidden transcriptional complexity between cattle subspecies. BMC Genomics. 2023;24 1:108. doi:10.1186/s12864-023-09212-9.

93. Paten B, Novak AM, Eizenga JM and Garrison E. Genome graphs and the evolution of genome inference. Genome Res. 2017;27 5:665-76. doi:10.1101/gr.214155.116.

94. Liu R, Tearle R, Low WY, Chen T, Thomsen D, Smith TPL, et al. Distinctive gene expression patterns and imprinting signatures revealed in reciprocal crosses between cattle sub-species. BMC Genomics. 2021;22 1 doi:10.1186/s12864-021-07667-2.

95. Di Tommaso P, Chatzou M, Floden EW, Barja PP, Palumbo E and Notredame C. Nextflow enables reproducible computational workflows. Nat Biotechnol. 2017;35 4:316-9. doi:10.1038/nbt.3820.

96. Rosen BD, Bickhart DM, Schnabel RD, Koren S, Elsik CG, Tseng E, et al. De novo assembly of the cattle reference genome with single-molecule sequencing. Gigascience. 2020;9 3:giaa021-giaa. doi:10.1093/gigascience/giaa021.

97. Li H, Handsaker B, Wysoker A, Fennell T, Ruan J, Homer N, et al. The Sequence Alignment/Map format and SAMtools. Bioinformatics. 2009;25 16:2078-9. doi:10.1093/bioinformatics/btp352.

98. Okonechnikov K, Conesa A and García-Alcalde F. Qualimap 2: advanced multi-sample quality control for high-throughput sequencing data. Bioinformatics. 2016;32 2:292-4. doi:10.1093/bioinformatics/btv566.

99. Li H. Minimap2: pairwise alignment for nucleotide sequences. Bioinformatics. 2018;34 18:3094-100.

100. Cock PJA, Antao T, Chang JT, Chapman BA, Cox CJ, Dalke A, et al. Biopython: freely available Python tools for computational molecular biology and bioinformatics. Bioinformatics. 2009;25 11:1422-3. doi:10.1093/bioinformatics/btp163.

101. Marçais G, Delcher AL, Phillippy AM, Coston R, Salzberg SL and Zimin A. MUMmer4: A fast and versatile genome alignment system. PLoS Comp Biol. 2018;14 1:e1005944. doi:10.1371/journal.pcbi.1005944.

102. Nattestad M and Schatz MC. Assemblytics: a web analytics tool for the detection of variants from an assembly. Bioinformatics. 2016;32 19:3021-3. doi:10.1093/bioinformatics/btw369.

103. Shumate A and Salzberg SL. Liftoff: accurate mapping of gene annotations. Bioinformatics. 2021;37 12:1639-43. doi:10.1093/bioinformatics/btaa1016.

104. Liao Y, Smyth GK and Shi W. The R package Rsubread is easier, faster, cheaper and better for alignment and quantification of RNA sequencing reads. Nucleic Acids Res. 2019;47 8:e47-e. doi:10.1093/nar/gkz114.

105. Robinson MD, McCarthy DJ and Smyth GK. edgeR: a Bioconductor package for differential expression analysis of digital gene expression data. Bioinformatics. 2009;26 1:139-40. doi:10.1093/bioinformatics/btp616.

106. Ritchie ME, Phipson B, Wu D, Hu Y, Law CW, Shi W, et al. limma powers differential expression analyses for RNA-sequencing and microarray studies. Nucleic Acids Res. 2015;43 7:e47-e. doi:10.1093/nar/gkv007.

107. Morison IM, Ramsay JP and Spencer HG. A census of mammalian imprinting. Trends Genet. 2005;21 8:457-65. doi:10.1016/j.tig.2005.06.008.

108. Emms DM and Kelly S. OrthoFinder: phylogenetic orthology inference for comparative genomics. Genome Biology. 2019;20 1:238. doi:10.1186/s13059-019-1832-y.

109. Jin F, Li Y, Dixon JR, Selvaraj S, Ye Z, Lee AY, et al. A high-resolution map of the three-dimensional chromatin interactome in human cells. Nature. 2013;503 7475:290-4. doi:10.1038/nature12644.

Tables

Table 1. Mapping statistics of Angus and Brahman reference genomes.

	Angus	Brahman
Mapped reads*	1,455,481,398	1,457,794,807
Duplication rate (%)*	10	14
CpGs with $\geq$ 10X coverage in all samples	22,116,287	21,962,589
CpG coverage*	30	30

* Mean of all samples.

Table 2. Number of CpGs in the Angus and Brahman reference genomes.

	Angus	Brahman
Total CpGs[A]	25,712,300	25,799,151
CpGs aligned to other reference[B]	25,209,966	25,228,509
CpGs shared in other genome[C]	18,813,726	18,781,688
CpGs affected by SNP[D]	993,318	1,003,167
Unresolved CpGs[E]	5,402,922	5,443,654

[A] Total number of CpGs present within the genome.

[B] Number of CpGs that could be aligned from one genome to the other using Minimap2 [99].

[C] Number of CpGs in B that were CpGs in both species.

[D] Number of CpGs in B that were a CpG in one species but are no longer CpGs in the other.

[E] Number of CpGs in B that could not be confidently assigned as either shared or a SNP.

Table 3. Significant imprinted DEGs and their overlap with DMRs when using the Brahman reference genome.

Gene ID	Gene name	Protein name	Increased expression in Brahman*	Number of hypo-DMRs in Brahman	Number of hyper-DMRs in Brahman
Breed comparison					
ENSBIXG00005001873	*Smoc1*	SPARC-related modular calcium-binding 1	Yes	16	1
ENSBIXG00005012203	*Nnat*	Neuronatin	No	37	1
ENSBIXG00005014559	*Gdap1l1*	Ganglioside-induced differentiation-associated protein 1 like 1	No	30	2
ENSBIXG00005009822	*Dgat1*	Diacylglycerol O-acyltransferase 1	Yes	3	2
ENSBIXG00005024991	*Slc22a18*	Solute-carrier family 22 member 18	Yes	2	3
ENSBIXG00005004279	*Kcnq1*	Potassium voltage-gated channel subfamily Q member 1	Yes	9	5
ENSBIXG00005007141	*Ppp1r9a*	Protein phosphatase 1 regulatory subunit 9A	Yes	11	5
Dam of origin comparison					
ENSBIXG00005019306	*Zc3h12c*	Zinc finger CCCH-type containing 12C	Yes	5	1
ENSBIXG00005007141	*Ppp1r9a*	Protein phosphatase 1 regulatory subunit 9A	Yes	3	2
Sire of origin comparison					
ENSBIXG00005007073	*Dscam*	DS cell adhesion molecule	No	38	5
ENSBIXG00005021735	*Htr2a*	5-hydroxytryptamine receptor 2A	Yes	18	2
ENSBIXG00005012203	*Nnat*	Neuronatin	No	8	0
ENSBIXG00005009822	*Dgat1*	Diacylglycerol O-acyltransferase 1	Yes	0	1
ENSBIXG00005016997	*Axl*	AXL receptor tyrosine kinase	Yes	9	1
ENSBIXG00005025694	*Ndn*	Necdin MAGE family member	No	0	1

ENSBIXG00005024991	*Slc22a18*	Solute-carrier family 22 member 18	Yes	0	0
ENSBIXG00005013434	*Tfpi2*	Tissue factor pathway inhibitor 2	No	25	0

* Increased expression in Brahman denotes genes that were significantly more highly expressed in Brahman than in Angus. “No” denotes that gene was significantly more highly expressed in Angus.

Table 4. Significant imprinted DEGs and their overlap with DMRs when using the Angus reference genome.

Gene ID	Gene name	Protein name	Increased expression in Angus*	Number of hypo-DMRs in Angus	Number of hyper-DMRs in Angus
Breed comparison					
ENSBIXG00000024138	*Esr2*	Estrogen receptor 2	No	4	8
ENSBIXG00000021864	*Nnat*	Neuronatin	Yes	2	32
ENSBIXG00000015750	*Zfp90*	Zinc finger protein 90	Yes	2	1
ENSBIXG00000012277	*Dlgap2*	DLG-associated protein 2	No	11	23
ENSBIXG00000005197	*Ppp1r9a*	Protein phosphatase 1 regulatory subunit 9A	No	9	15
Dam of origin comparison					
ENSBIXG00000011151	*Zc3h12c*	Zinc finger CCCH-type containing 12C	No	0	4
ENSBIXG00000005197	*Ppp1r9a*	Protein phosphatase 1 regulatory subunit 9A	No	6	3
Sire of origin comparison					
ENSBIXG00000027129	*Dscam*	DS cell adhesion molecule	Yes	5	35
ENSBIXG00000024138	*Esr2*	Estrogen receptor 2	No	3	1
ENSBIXG00000008539	*Htr2a*	5-hydroxytryptamine receptor 2A	No	1	17
ENSBIXG00000021864	*Nnat*	Neuronatin	Yes	1	11
ENSBIXG00000012321	*Dgat1*	Diacylglycerol O-acyltransferase 1	No	0	0
ENSBIXG00000010895	*Sdhd*	Succinate dehydrogenase complex subunit D	Yes	0	5
ENSBIXG00000016809	*Axl*	AXL receptor tyrosine kinase	No	0	8
ENSBIXG00000015087	*Mkrn3*	Makorin ring finger protein 3	Yes	1	1
ENSBIXG00000015080	*Ndn*	Necdin MAGE family member	Yes	1	0

ENSBIXG00000012277	*Dlgap2*	DLG-associated protein 2	No	1	11
ENSBIXG00000028529	*Slc22a18*	Solute-carrier family 22 member 18	No	0	2

* Increased expression in Angus denotes genes that were significantly more highly expressed in Angus than in Brahman. “No” denotes that gene was significantly more highly expressed in Brahman.

Figures

Figure 1. Overview of methods. A.) Representation of the four genetic groups used in this study. The blue cow represents pure Angus individuals (BTBT). The blue then orange cow represents individuals with an Angus sire and Brahman dam (BTBI). The orange then blue cow represents individuals with a Brahman sire and Angus dam (BIBT). The orange cow represents pure Brahman individuals (BIBI). **B.)** Process of mapping WGBS reads (light green-blue), and RNA-seq reads (green) to both the Brahman and Angus reference genomes. **C.)** Simple representation of shared and breed-specific CpG sites between Brahman and Angus reference genomes. **D.)** Breed-specific CpGs arise from a single nucleotide polymorphism between Brahman and Angus, such as spontaneous deamination of the C to a T. Structural variants, such as indels between the two genomes, can introduce or remove CpGs in one genome relative to the other. **E.)** Simple representation of how differential methylation can be influenced by breed-specific CpGs. The grey boxes demonstrate how a differentially methylated cytosine is identified when both breeds share that site. Essentially, one compares the number of Cs and Ts in group 1 against the number of Cs and Ts in group 2. If one group reports significantly more Cs than the other, it is considered differentially methylated. The yellow boxes represent a breed-specific CpG where only samples from one group have that CpG, so differential methylation cannot be determined. The red boxes represent a situation where the CpG is present in one subspecies, but spontaneous deamination has mutated the CpG site into a TpG site in the other subspecies. In this case, differential methylation can be calculated. However, it will be erroneous as only one group has a true CpG at that site. **F.)** Graphical representation of how breed differences were determined. We compared methylation and gene expression between BTBT and BIBI samples. **G.)** Graphical representation of how we determined parent-of-origin effects (POEs). Maternal POEs were determined by comparing BTBT and BIBT against BIBI and BTBI. Paternal POEs were determined by comparing BTBT and BTBI against BIBI and BIBT.

Figure 2. A.) PCA plot showing separation of genetic groups by methylation. Blue represents BTBT, orange represents BIBI, green represents BTBI and red represents BIBT. The X axis is principal component 1, and the Y axis is principal component 2. **B.)** PCA plot showing separation of genetic groups by gene expression data; colours are same as **A**. The X axis is the first dimension of the logFC, and the Y axis is the second dimension of the logFC.

Figure 3. A.) Boxplot showing mean global CpG methylation for samples belonging to the four genetic groups. When Brahman and Angus are mapped to their respective genomes, they tend to be more methylated than when mapped to the incorrect reference. The hybrids (BTBI and BIBT) tend toward hypermethylation when mapped to the Angus reference though this is only significantly different in BIBT. * denotes p-value < 0.05, Wilcox test. **B.)** Boxplot showing mean global CpG methylation for samples belonging to each of the four genetic groups mapped to each reference genome; however, this time, only the shared CpGs were considered. Here, there is no significant difference in methylation within genetic groups regardless of which reference genome is used. **C.)** Boxplot showing the mean frequencies methylation states observed in Angus and Brahman after 100 permutations. Dark blue represents hypomethylated CpG sites (methylation $\leq$ 35%). Dark orange bars represent hypermethylated CpG sites (methylation $\geq$ 65%). * denotes p-value < 0.05, Mann-Whitney U-test. The X-axis denotes the breed, either Angus or Brahman. The Y-axis represents the count, i.e., the number of sites that fell into the hypo- or hypermethylated categories.

Figure 1

Click here to access/download;Figure;Figure 1 - methylation-Overview_figure.png

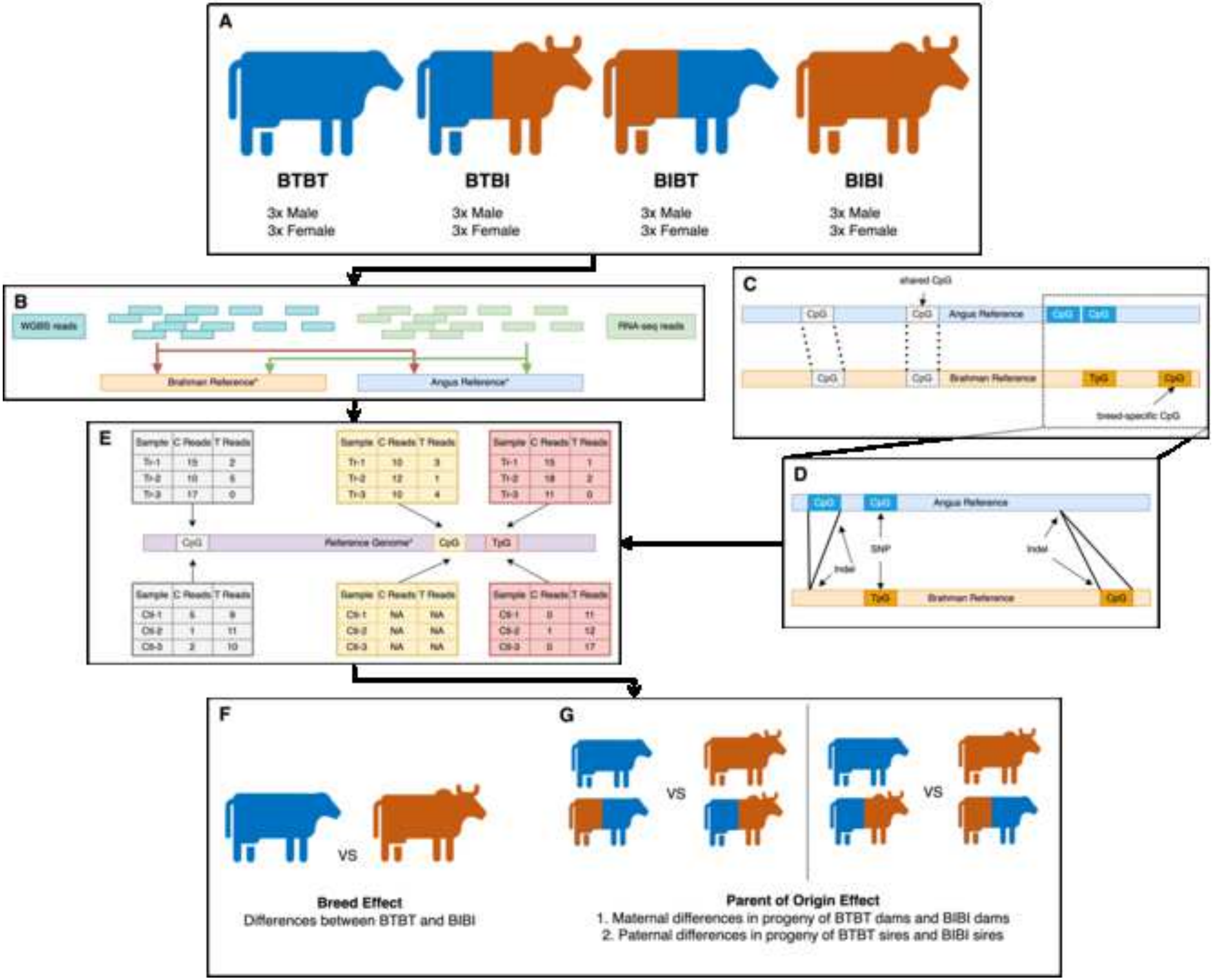

Figure 2

Click here to access/download;Figure;Figure 2 - PCA.Brahman.meth_and_rna.png

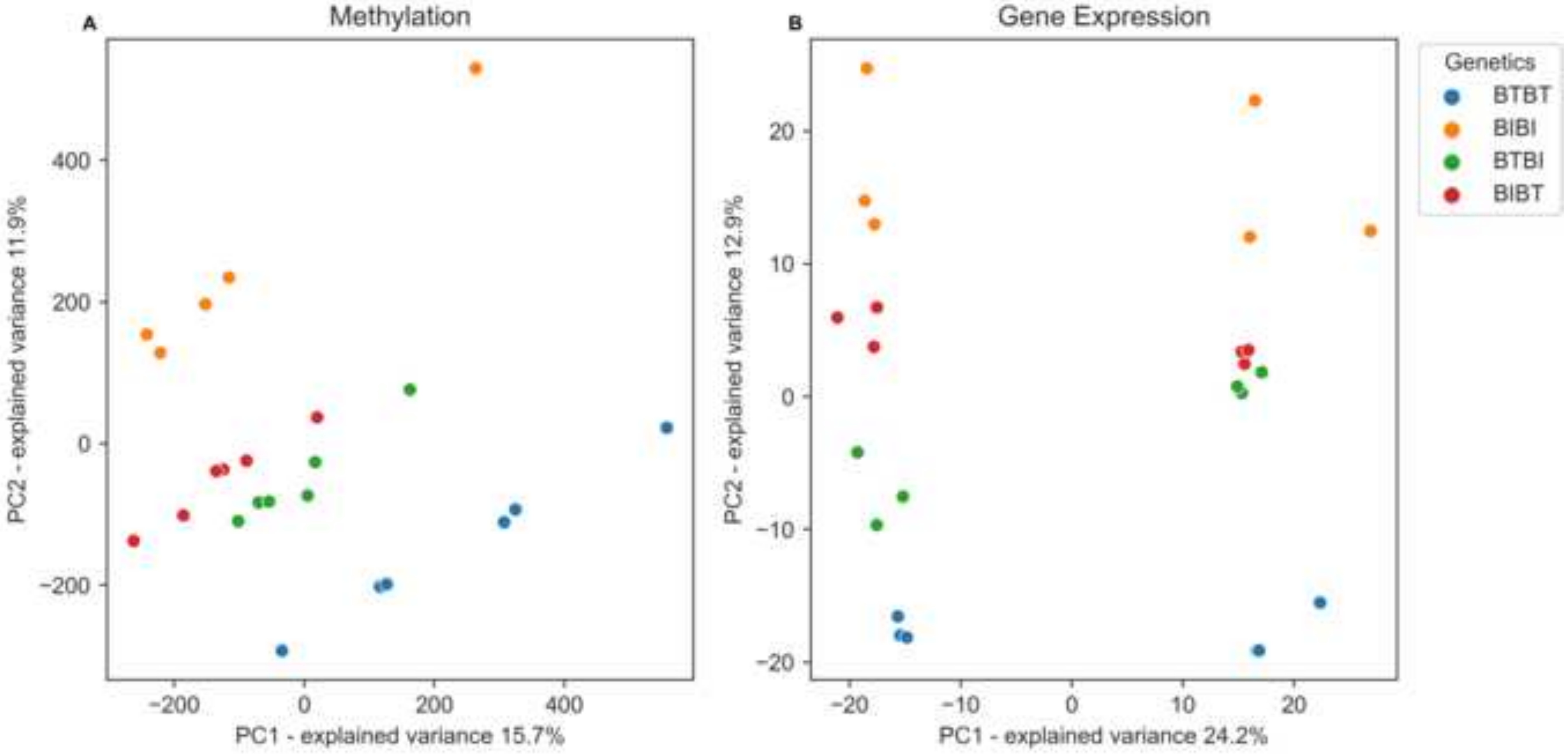

Figure 3

Click here to access/download;Figure;Figure 3 - genome_effect.png

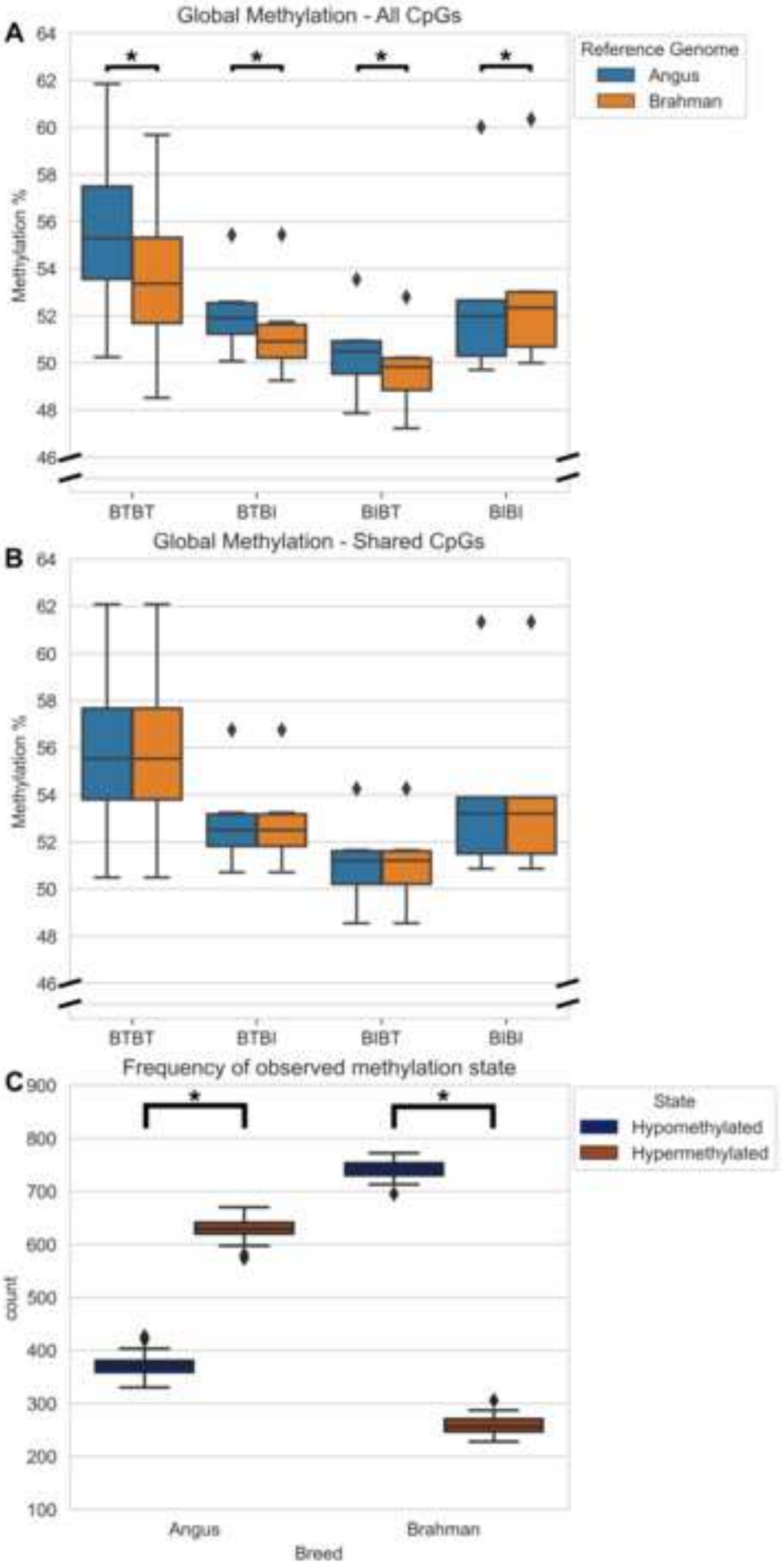

Supplementary table 1

Click here to access/download
Supplementary Material
S. table 1 - WGBS mapping stats.xlsx

Supplementary table 2

Click here to access/download
Supplementary Material
S. table 2 - global coverage stats.xlsx

Supplementary table 3

Click here to access/download
Supplementary Material
S. table 3 - CpG coverage stats.xlsx

Supplementary table 4

Click here to access/download
Supplementary Material
S. table 4 - global methylation.xlsx

Supplementary table 5

Click here to access/download
Supplementary Material
S. table 5 - group regional methylation means.xlsx

Supplementary table 6

Click here to access/download
Supplementary Material
S. table 6 - CpG Mapping between refs.xlsx

Supplementary table 7

Click here to access/download
Supplementary Material
S. table 7 - SNPs and SV enrichment.xlsx

Supplementary table 8

Click here to access/download
Supplementary Material
S. table 8 - meth change.xlsx

Supplementary table 9

Click here to access/download
Supplementary Material
S. table 9 - DMRs.xlsx

Supplementary table 10

Click here to access/download
Supplementary Material
S. table 10 - DEGs.xlsx

Supplementary table 11

Click here to access/download
Supplementary Material
S. table 11- sample info.xlsx

Supplementary table 12

Click here to access/download
Supplementary Material
S. table 12 - imprinted_genes.xlsx

Supplementary figure 1

Click here to access/download
Supplementary Material
S. fig 1 - Brahman.Angus.corr.heatmap.png

Supplementary figure 2

Click here to access/download
Supplementary Material
S. fig 2 - exons.methylation.png

Supplementary figure 3

Click here to access/download
Supplementary Material
S. fig 3 - 5utr.methylation.png

Supplementary figure 4

Click here to access/download
Supplementary Material
S. fig 4 - promoters.methylation.png

Supplementary figure 5

Click here to access/download
Supplementary Material
S. fig 5 - intergenic.methylation.png

Supplementary figure 6

Click here to access/download
Supplementary Material
S. fig 6- introns.methylation.png

Supplementary figure 7

Click here to access/download
Supplementary Material
S. fig 7 - 3utr.methylation.png

Supplementary figure 8

Click here to access/download
Supplementary Material
S. fig 8 - predicted_enhancer.methylation.png

Supplementary figure 9

Click here to access/download
Supplementary Material
S. fig 9 - dinucleotide_snps_breed_comparison.png

Supplementary figure 10

Click here to access/download
Supplementary Material
S. fig 10 - genome effect.png

Supplementary figure 11

Click here to access/download
Supplementary Material
S. fig 11 - search region.png
