# Supplementary material for: DNA methylation analysis to differentiate reference, breed, and parent-of-origin effects in the bovine pangenome era
Source: Gigascience. 2024 Oct 17;13:giae061. doi: 10.1093/gigascience/giae061 (PMC11484048; doi:10.1093/gigascience/giae061)
Supplement: giae061_GIGA-D-23-00314_Revision_1 [file giae061_giga-d-23-00314_revision_1.pdf]

GigaScience

DNA methylation analysis to differentiate reference, breed and parent-of-origin effects in the bovine pangenome era

--Manuscript Draft--

**Manuscript Number:**	GIGA-D-23-00314R1
**Full Title:**	DNA methylation analysis to differentiate reference, breed and parent-of-origin effects in the bovine pangenome era
**Article Type:**	Research
**Funding Information:**	JS Davies Bequest — Not applicable
**Abstract:**	Background<br>Most DNA methylation studies have used a single reference genome with little attention paid to the bias introduced due to the reference chosen. Genetic variation, including single nucleotide polymorphism (SNPs) and structural variants (SVs), can lead to differences in methylation sites (CpGs) between individuals of the same species. We analysed whole genome bisulfite sequencing (WGBS) data from the fetal liver of Angus (Bos taurus taurus), Brahman (Bos taurus indicus) and reciprocally crossed samples. Using reference genomes for each breed from the Bovine Pangenome Consortium, we investigated the influence of reference genome choice on the breed- and parent-of-origin effects in methylome analyses.<br>Results<br>Our findings revealed that about 75% of CpG sites were shared between Angus and Brahman, ~5% were breed-specific, and ~20% were unresolved. We demonstrated up to ~2% quantification bias in global methylation when an incorrect reference genome was used. Furthermore, we found that SNPs and SVs were 14-fold (p-value <) and 1.18-fold (p-value <) higher in CpGs, respectively, compared to the rest of the genome. We found a poor association between differentially methylated regions (DMRs) and differentially expressed genes (DEGs) and suggest that DMRs may be impacting enhancers that target these DEGs. DMRs overlapped with imprinted genes, of which one, DGAT1, which is important for fat metabolism and weight gain, was found in the breed-specific and sire-of-origin comparisons.<br>Conclusions<br>This work demonstrates the need to consider reference genome effects to explore genetic and epigenetic differences accurately and identify DMRs involved in controlling certain genes.
**Corresponding Author:**	Callum MacPhillamy<br>The University of Adelaide<br>Roseworthy, SA AUSTRALIA
**Corresponding Author Secondary Information:**	
**Corresponding Author's Institution:**	The University of Adelaide
**Corresponding Author's Secondary Institution:**	
**First Author:**	Callum MacPhillamy
**First Author Secondary Information:**	
**Order of Authors:**	Callum MacPhillamy
	Tong Chen
	Stefan Hiendleder
	John Williams
	Hamid Alinejad-Rokny
	Wai Low

Powered by Editorial Manager® and ProduXion Manager® from Aries Systems Corporation

Order of Authors Secondary Information:	
Response to Reviewers:	GIGA-D-23-00314 Authors’ responses to reviewers<br><br>Reviewer 1<br>In this study, the authors investigate the impact of reference genome choice on downstream epigenetic analysis. To explore this impact, the authors sequenced and analyzed the Whole Genome Bisulfite Sequencing (WGBS) data from two cattle breeds, Bos taurus taurus (BT) and Bos taurus indicus (BI), as well as their reciprocal cross offspring (BTBI, BIBT). Through the analysis, the authors found (1) up to 2% quantification bias in global methylation when using an incorrect reference genome. (2) SNPs and SVs were 8-fold and 1.13-fold higher in CpGs than in the background, respectively, with enrichment p-values both smaller than 5 * 10^-324. (3) There was little overlap between DMR and DEG surroundings regarding genomic coordinates. The first two findings emphasized the importance of choosing the correct reference genome. Overall, the research topic is important as it can reveal/emphasize the potential computational artifacts in epigenetic analyses, and the research design is clear and straightforward. However, we have several questions and comments to present regarding the manuscript.<br><br>Major points:<br>In the manuscript's title, the phrase "genetics of epigenetics" is employed to highlight the phenomenon that reference genome choice can impact methylation signals, potentially resulting in inaccurate findings. Nevertheless, this phrase is overly expansive and not entirely reflective of the content covered in this study. The term "genetics" typically implies a connection to inheritance, making the phrase more pertinent for research examining the genetic factors associated with/regulating DNA methylation, such as methylation Quantitative Trait Loci (meQTL) studies or genetics causality studies. On the other hand, though "genetics" denotes changes in DNA sequence, it usually refers to the variations in the individual genomes, not on the reference genomes. Therefore, to enhance clarity and specificity, it is recommended to revise the title to a more precise expression reflecting the paper's key message.<br>We appreciated the reviewer’s comments regarding the clarity of the title and have altered it to “DNA methylation analysis to differentiate reference, breed and parent-of-origin effects in the bovine pangenome era”.<br><br>In the section `Overview of DNA methylation patterns`, the authors summarize the methylation levels of different genomic regions using the global mean of methylation. This information is too limited. The authors should consider displaying the whole distribution of methylation levels using density plots or violin plots, which contain more information (mean, median, quantiles, and skewness). It is also suggested that the descriptive statistics of genomic regions be organized into a table.<br><br>Response: We appreciate the reviewers' comments regarding the methylation overview section. We agree that descriptive statistics such as mean, variance, skewness would be helpful and have added a supplementary table (S. Table 5). This table contains descriptive stats for each of the seven genomic regions (5’UTR, 3’UTR, enhancer, exon, intergenic, intron, promoter) among genetic groups (BTBT, BTBI, BIBT, BIBI) for Brahman and Angus reference genome.<br><br>However, we maintain the presentation of the mean methylation with slight changes in the main text figure (Figure 3A-B). Wulfridge et al. 2019 also presented global methylation differences between samples mapped to different genomes in similar way as we did in figure 3A-B. Additionally, to plot the distribution of all CpG sites per sample would result in a violin plot that spans from 0 – 100% methylation with the widest point around the global mean for that sample and would not be very informative in that way, which was why we adopted the approach of Wulfridge et al. 2019. We altered the figures to display each sample from each group and their median methylation level when mapped to both reference genomes. We performed a Wilcoxon rank sum test on each sample to compare the distribution of methylation between the two genomes and have reported the p-values in Table 3.<br><br>In the section `Enrichment of SNPs affecting CpG sites`, the p-value calculation is problematic. Based on the Jupyter Notebook code in the Github link of supplementary table 7, the enrichment significance is computed by the following code:

`stats.binomtest(k=(cpg_snps - cpg_snps_sv), n=total_snps, p=(total_snps / brahman_len), alternative='greater')`. Here, the authors assume the `p=total_snps / brahman_len` to be the background probability of having a mutation in the CpG sites. However, this does not seem right since the formula denotes that p is the probability of having a mutation in a single nucleotide, not for a CpG dinucleotide. Similarly, the SV's p-value calculation should be corrected as well. The authors should also explicitly write the enrichment analysis details in the method section.

Response: We thank the reviewers for taking the time to review the supplied code. When determining the probability that a CpG site was affected by a mutation, it is only necessary to consider single mutations, as any mutation of the 'C' alters the CpG site. Similarly, any mutation of the G also alters the CpG site. If both 'C' and 'G' in the CpG were mutated, we scored this as two SNPs occurring in this CpG site. Therefore, using the denominator SNP rates at single nucleotide level is appropriate as we effectively scored the CpG at single nucleotide level. For SV's p-value calculation, we used a different way to calculate enrichment of SVs. This essentially was a binomial test comparing CpG in SVs versus CpG not in SVs. The null hypothesis was CpG proportion was equal in SVs and non-SVs, which was rejected at $p < 0.001$. We have amended the methods to explicitly state these details of the enrichment analysis at lines 642-667.

"SNP and SV Enrichment
To determine whether CpGs were significantly impacted by SNPs, we identified all SNPs between Brahman and Angus that impacted a CpG site. As Brahman autosomes were used as the reference to minimap2, we used the coordinates of all CpGs within the Brahman genome to identify which SNPs in the VCF file were altering a CpG site. We determined the probability of a SNP occurring as the number of SNPs (n) identified between a given pair of autosomes divided by the length of the autosome (l), P("SNP_occurring") = n/l. As a CpG site only needs a single SNP to destroy it, one need only consider the probability of a single base changing, I.e., either the 'C' or the 'G'. To test whether CpGs were significantly more likely to be affected by a SNP, we performed a binomial test for each autosome where k was equal to the number of SNPs affecting a CpG site, n was the number of SNPs that occurred, and P was the probability of a SNP occurring.

To assess whether CpGs were significantly enriched within SVs identified between the two genomes, we counted the number of CpG dinucleotides occurring within SV sequences and compared that against the number of CpGs that occurred in non-SV regions. To determine the probability of a CpG occurring outside an SV, we first identified the SV coordinates from the VCF file produced by PAFtools and constructed a bed file of SVs for each reference genome. Next, we used BEDTools [104] to generate coordinates of the complementary regions, i.e., the non-SV regions of each genome. We then extracted the fasta sequence of these regions for each genome. We then counted the number of CpGs that occurred in these regions and divided them by the combined length of each region to determine the probability of a CpG occurring in the non-SV portion of the genome. We then performed a binomial test to determine if CpGs were more likely to occur within an SV than non-SV regions."

Additionally, as the SNPs between reference genomes are estimated using MUMmer, whose results are not guaranteed to be perfect, some validations of MUMer output accuracy are needed. Such validations are essential for this study because if MUMer outputs inaccurate SNP results, the background mutation probability will be distorted, leading to imprecise enrichment p-values. MUMer's accuracy can be obtained from the literature if there is any, or evaluated by the authors themselves (e.g., supplying two copies of the same input reference genome to MUMmer, any SNP found should be false positive; and supplying a reference genome and a variation of the reference genome with bases randomly mutated at a given rate, and evaluate if the software can accurately recovers the mutation frequency).

Response: We appreciate the reviewer's comments about the potential unreliability of MUMmer. We tested the evaluated how well MUMmer is able to identify SNPs and found it to be poor. We evaluated Minimap2 as an alternative to identify SNPs and found this to be far superior (Supplementary Table 13). The steps of this evaluation have been added to the methods at lines 616-640. We then reanalysed all related

sections using SNPs identified by Minimap2 and PAFtools.js.

“Identification of SNPs and SVs between genomes
To determine the accuracy of minimap2 in identifying SNPs between the Brahman and Angus genomes, we first introduced artificial mutations into each genome using SNP Mutator [103]. The Angus and Brahman genomes have previously been reported to differ by ~1% [39]. Therefore, to determine how well minimap2 can detect SNPs between sequences that are divergent by ~1%, we first simulated mutations in each autosome for both species. For example, chromosome 1 in the Angus reference genome is 157,005,132 bp long, so we set the number of substitutions to 1,570,051 SNPs. In addition, the random seed was set to 12, and the number of times each autosome was mutated was set to 1. We repeated this for all autosomes in the Brahman and Angus genomes, adjusting the number of SNPs to maintain the 1% divergence in each autosome. We then mapped the mutated autosomal sequence to the original sequence for each autosome and breed, giving us 58 “replicates”. Minimap2 [59] and PAFtools were used to identify variants between the original and mutated sequences. The minimap2 mapping parameters used were ‘-x asm10, -c, --cs’, followed by PAFtools ‘call —f’, where the file provided to the —f argument was the original, unmutated autosomal sequence. Minimap2 and PAFtools showed a mean accuracy of ~99% across the 58 autosomes, suggesting a good ability to identify SNPs between the two breeds (S. Table 13). We then aligned each autosome from Brahman to each autosome from Angus, using minimap2 with the parameters ‘-cx asm10’ and ‘—cs’. The output from minimap2 was then used as input to paftools.js call with the parameter ‘-f <reference_autosome.fa>’, where reference_autosome.fa refers to the autosome that was supplied first to minimap2, i.e. the reference sequence, not the query. We then used the output VCF files to determine the SNP and SVs between the two genomes.”

The authors found that over 80% of CpG SNP were either C2T or G2A. Although a potential explanation from the spontaneous deamination perspective is provided, the ratio still looks high. The authors would need to provide additional support for this finding, e.g., theoretical support where probabilistic calculations show the high ratio falls into a certain confidence interval, or empirical support where a different method yields a similar high ratio in other mammals. Having either support would eliminate the concern that this high ratio reflects computational artifacts.

Response: We appreciate the reviewers concerns and have included a reference to a human study that found similarly high levels of spontaneous deamination in SNPs affecting CpG sites. This can be found at line 241-243, which is as follows: “Looking more closely at the CpG SNP changes, we found that most (~81%) of the CpG SNP were either C to T or G to A changes (S. figure 9), which is very similar to the number of CpG SNP changes detected in humans (80.7%) [60]” .

The section `Choice of reference genome influences methylome results` is the primary section supporting the manuscript's main conclusion; there is room for improvement, and more details should be provided to support the conclusion better. Specifically,
The authors used boxplots to present the overall CpG methylation difference caused by reference choices. The boxplot can be improved. Raw data points should be shown on the plot, with a line connecting the data point between groups, indicating they are the same biological sample but used different reference genomes. It is also advised to report the exact p-value in the figure, not just the significance symbol. Besides, it is better to compare the whole distribution, not just the global mean, as also denoted in previous comments.

Response: We believe we have addressed this in the revised Figure 3, which now includes mean global methylation for each sample per genetic group and partitioned by reference genomes used. As per our first response to comment 2 above, we prefer to show mean global methylation following Wulfridge et al. 2019. We have included the mean, variance and p-value of different genetic group comparisons in S Table 8. Additionally, we have added how the p-values presented in Table 3 were determined at lines 669-684.

“Determining quantification bias between genomes
To determine whether there was a significant quantification bias between the Brahman

and Angus reference genomes for a given sample, we compared the vector of all CpG sites with at least 10X coverage when mapped to Angus in sample I against the vector of all CpG sites with at least 10X coverage when mapped to Brahman. To ensure the vectors were equal, we randomly subset the larger vector to be the same length as the smaller, i.e., if the Brahman reference genome had more CpG sites with 10X coverage for that sample, the Brahman vector was randomly subset to match the number of CpG sites in the Angus vector for that sample. We repeated this for all samples using all CpGs and again with just the CpG sites marked as shared (Table 3; S. table 8). We then pooled all CpG sites for each sample within a group, e.g. all samples from BTBT, and determined whether the CpG methylation differed significantly for that group when mapped to Angus and Brahman. The p-value was determined using a Wilcoxon Rank Sum test and adjusted for multiple testing using the Benjamini-Hochberg procedure. We identified variable CpGs by matching the shared CpGs between reference genomes and then identifying those with an absolute methylation difference greater than 10%."

Only the global methylation of a sample is presented, which is too abstract to be practically useful. Since most CpG sites have methylation levels close to 0 or 1, the global methylation change may only represent different compositions of CpG sites in the two reference genomes. It may not be related to how methylation levels would change on the site level. The site level methylation is more worthwhile to examine than the global methylation as it is the basis of epigenetic analysis; if there is no change on the shared sites (which is the majority of sites) no matter which reference genome is used, then the significance of this manuscript's conclusion is greatly diminished. Therefore, It is highly suggested to focus on the site level methylation analysis, e.g., plot the scatter plot/2d-density plot where the x-axis is the methylation level using one reference genome, and the y-axis is the methylation level using another reference genome, each dot is the shared CpG sites in 2 references, and summarize the similarity using correlation/R square, Rooted Mean Squared Error (RMSE), etc.

To follow up on bullet point b, the authors can also examine what specific CpG sites have relatively large deviations in methylation level when incorrect reference is used and explore the potential reason and consequence for the difference from genomic and biological pathway perspectives. Another good side for this disentangling is that this would also yield a set of confidence sites (sites not sensitive to the reference choice) and variable sites (sites sensitive to the reference choice). If the variable sites account for a large proportion of all sites, this would be strong evidence for the manuscript's conclusion. The disentangling would also be helpful to the bovine research community (say whether a published study used the correct reference genome is unclear, how much of their conclusion and what proportion of their findings in the epigenetic analysis should be trusted? Is it necessary to reanalyze everything?)

Response: The authors thank the reviewers for their comments in 6b and 6c. As a summary of the overall differences among groups when using different reference genomes, we believe global methylation is useful similar to Wulfridge et al 2019's DNA methylation quantification bias in mice. We have included Figure 3C and provided a table to summarize the number of variable CpG sites (S. table 9). From Figure 3C, even among shared CpG sites, there are sites that deviate from the diagonal, which indicate reference genome effects. The shared CpG affected by reference genome effect is 264,023 sites or 1.87%. This is based on using an absolute methylation difference threshold of 10% to call the CpG sites as variable because a difference of 10% could have biological effect is based on Leenen et al., 2016 and Thomson et al., 2022. Although the proportion of shared CpG sites affected by reference genome effect is small, the breed specific CpG sites is ~1.1 million, which is substantial and hence, a pangenome approach is needed to capture the methylation level. We provided a Supplementary table 9 of variable CpG sites for the bovine research community. We have added to the discussion to address this at lines 459-464. "Moreover, we demonstrated a number of CpG sites that appear to be sensitive to reference genome choice, differing by more than 10% between the two genomes. It is likely that these CpG sites would confound downstream analysis, especially if they overlap with regions of interest such as cis-regulatory elements. Thus, this demonstrates that even by attempting to control for reference genome differences, quantification bias can remain."

It is hard to guess what the DMR analysis is about in the section `Choice of reference genome influences methylome results` (lines 260-262). A more precise explanation of the DMR analysis design is needed. Besides, more research should investigate the reference genome's influence on downstream DMR analysis. Some directions include (1) Intra-breed perspective: For the samples in the same breed, if their data are aligned to 2 reference genomes forming 2 groups, will these 2 groups have DMR identified on the shared CpG regions? (2) Inter-breed perspective: For analyzing the DMR when comparing one breed (BTBT) to another breed (BIBI), there are 4 possibilities in total (1 correct case: BTBT used BT reference, BIBI used BI reference; and 3 incorrect cases where the matching between samples and reference genomes is not exact). How will the DMR results differ between the correct case and incorrect cases? The Venn diagram is suggested to show the concordance of DMR results in different comparison scenarios.

Response: We agree that the section 'Identification of differentially methylated regions' is not clear at describing how the DMR analysis was carried out and what models were fitted. We have now included the design matrices of all fitted models as part of Supplementary table 14. Additionally, we have rearranged the paragraph in this section to improve clarity lines 686-728 (also pasted below). There were 6 models: 2 for breed effect, 2 for maternal effect and 2 for paternal effect. These were all logistic regression models and sex was always fitted as a covariate. The convention in differential methylation analysis and likewise, gene expression analysis with RNA-seq, is to choose a single reference genome to start the analysis. As such, to our knowledge, no tool can compare the impact of same samples called on different reference genomes. For example, the reviewers mentioned 1 correct and 4 incorrect cases: A) BTBT on Angus ref vs BIBI on Angus ref, B) BTBT on Angus ref vs BIBI on Brahman ref, C) BTBT on Brahman ref vs BIBI on Angus ref, and D) BTBT on Brahman ref vs BIBI on Brahman ref. The correct case is B but it is impossible to do with existing tools. We could do case A and D.

"Identification of differentially methylated regions
The methylKit package (v. 1.22.0) [77] was used to identify DMRs between breed and POE groups. We investigated breed effects by comparing BIBI samples with BTBT samples, maternal effects by comparing samples with BIBI dams (BIBI; BTBI) and those with BTBT dams (BTBT; BIBT) and paternal effects by comparing samples with BIBI sires (BIBI; BIBT) to those with BTBT sires (BTBT; BTBI) (S. table 14). The reference group was always the breed that matched the reference genome. For example, when BIBI and BTBT WGBS reads were aligned to the Brahman reference genome, BIBI samples were treated as the control group and BTBT as the treatment group.
We followed the pipeline described by the methylKit authors for DMR analysis [77]. Briefly, we only considered CpGs that were identified as shared. We then removed all CpG sites with less than 10X coverage and more than the 99.9th percentile of coverage. Reads with too high coverage (e.g. from PCR duplication bias) can impair the accurate determination of the methylation percentage at that site and is a recommended pre-processing step for methylKit [77]. We then normalized the coverage using the default methylKit normalization strategy. We merged the CpG counts per group using the 'unite' function with 'destrand = T' and 'min.per.group = 5L' so that a given CpG site had to be covered by at least ten reads in five out of six samples per group. For the parent of origin DMR analyses, we set 'min.per.group = 10L'.
We then identified differentially methylated cytosines between groups using the 'calculateDiffMeth' function, with sex as a covariate in the model. To determine differentially methylated regions, we used the 'tileMethylCounts' function with default parameters to divide the genome into regions for differential methylation analysis. This step allowed methylKit to divide the genome into non-overlapping regions based on the tiling windows. MethylKit then models the methylation at a given cytosine or region by fitting a logistic regression:
log(P_i/(1-P_i))=β_0+β_1*T_i+a_"sex" * 〖"Sex" 〗_i

Pi denotes the methylation proportion for sample i in samples 1,…,n, where n is the number of samples across both groups in the comparison [77]. Ti represents the groups (0 for control, 1 for treatment). β_0 denotes the log odds of the control group (fraction of reads reporting C / 1 – the fraction of reads reporting C). β_1 denotes the

Powered by Editorial Manager® and ProduXion Manager® from Aries Systems Corporation

log odds ratio between the control and treatment. α_"sex" denotes the parameter for the sex covariate and 〖"Sex" 〗_i denotes the sex (0 = male; 1 = female) for sample i. For further details, refer to Akalin, Kormaksson [77]. This design resulted in six different logistic models being fit: model 1A (breed comparison when aligned to the Angus reference), model 1B (breed comparison when aligned to the Brahman reference), model 2A (dam of origin comparison when aligned to the Angus reference) model 2B (dam of origin when aligned to the Brahman reference), model 3A (sire of origin when aligned to the Angus reference) and model 3B (sire of origin when aligned to the Brahman reference) (S. table 14). Any DMRs identified were either hypo- or hypermethylated with respect to the control group. We retained all DMRs with a difference in methylation of 10% and a qvalue of 0.01 for further analysis. An overview of the samples, reference genomes, types of CpGs and DMR analysis is given in Figure 1A-G."
In the section `Breed-specific CpGs show distinct methylation patterns`, the threshold choice of defining methylated (>0.65) and unmethylated (<0.35) should be justified. Is the threshold based on the data distribution or based on subjective choice? What is the rationale behind it? Do the shared CpGs also show a distinct methylation pattern similar to the breed-specific CpGs when different reference genomes are used?

Response: We have performed this analysis with the 35 and 65% thresholds as well as at 25 and 75%. We observed much the same pattern in both scenarios and have updated the manuscript at lines 304-306 ("We repeated this with "unmethylated" thresholds of 25% and "methylated" thresholds of 75%, with the CpG methylation values in between being considered hemimethylated [61].") and at lines 309-315 ("At both the 25 and 75%, and 35 and 65% thresholds, we observed significantly more Brahman-specific CpGs as hypomethylated than hypermethylated (Mann-Whitney U-test, p = 2.5 10-34) (Mann-Whitney U-test, p = 2.5 10-34), respectively Interestingly, we observed the inverse when considering Angus-specific CpGs; significantly more Angus-specific CpGs were hypermethylated than hypomethylated (Mann-Whitney U-test, p = 3.0 10-34) (Mann-Whitney U-test, p = 2.5 10-34), respectively (Figure 3D-E).")

We demonstrated at lines 266-269 that the shared CpGs tend to follow the same methylation pattern regardless of the genome used. However, there are some that still differ substantially (lines 282-285).

In the section `Breed-specific DMRs show poor association with DEGs`, the authors found that a small portion (~20%) of DMRs overlapped with DEG surroundings when comparing breeds. The authors should also report the portion of DEG whose surroundings overlapped with DMR. If both numbers are small, there will be concerns about whether such discordance is due to intrinsic biological or other technical reasons. For example, we noticed that in the DMR analysis, the authors controlled the sex effect when comparing the breed difference. While in the DEG analysis, the authors did not mention if they correct sex effects or not. Nevertheless, given that the RNA-seq and WGBS data are sequenced at different time points with different sequencing platforms, some sanity checks should be performed to ensure the group differences presented in both data are mainly the breed differences. Otherwise, the overlap analysis is pointless. To solve this problem, the authors can consider quantifying how much of the variance in transcriptome can be explained by the variance in the methylome (compute the canonical correlation between these 2 data modalities); alternatively, the authors can perform pathway enrichment analysis for both the DMR and DEG respectively and confirm if the differential analysis results are concordant in the pathway levels despite their difference in the genomic coordinates.

Response: We have added the number of DEGs that overlapped with a DMR at lines 331-332, 334-336, 356-357, 364-366, 376-378 and 378-380 (see below for responses). The number of DEGs that overlapped with a DMR was high, >88%. Given this result, we have re-written the title of this Method's section as "DMRs between breeds show limited overlap with DEG promoters" and modify our results to emphasize that DMRs are near or in gene bodies of DEG but showed poor overlap with promoters. We have fitted sex as a covariate in both methylation and gene expression (RNA-seq) analyses. We appreciate the reviewers' comments and concerns around how much variance in methylation explains the variation gene expression. To address this, we performed canonical cross correlation analysis, as suggested, to determine the correlation

Powered by Editorial Manager® and ProduXion Manager® from Aries Systems Corporation

between the RNA-seq and methylation data. We used DESeq2 to transform the RNA-seq counts data into a more appropriate format for CCA. We found that the R2 between the two datasets is 0.18, which is a moderate correlation; we have added this at lines 769-771. Given that methylome is only one of the factors influencing gene expression, this result is not surprising.

“Furthermore, we performed canonical correlation analysis (CCA) to estimate the coefficient of determination between gene expression and DNA methylation, R2 = 0.18, suggesting a moderate relationship between gene expression and DNA methylation.”

Furthermore, to avoid confusion with the DEG results presented, we reanalysed the DEGs using DESeq2. The model formula given to DESeq2 was `~0 + Genetics + Sex + Batch`. We have updated the methods at lines 762-763.

Lines 331-332: Only 0.3% of the DMRs overlapped with promoters of DEGs, despite 99% of significant DEGs being overlapped by a DMR.

Lines 334-336: Most (~73%) of these DMRs fell into the putative enhancer region, while only 0.3% of the DMRs overlapped with a DEG promoter, despite substantially more (1,872) DEGs observed (S. table 10) and 99% overlapping with a DMR.

Lines 356-357: Around 6% (1,236) of the DMRs overlapped with DEGs with ~78% of DEGs being overlapped by a DMR.

Lines 364-366: Using Angus as the reference, we observed 1,254 (~5%) DMRs overlap with the 358 DEGs and ~88% of DEGs covered by a DMR.

Lines 376-378: Around 6% (1,236) of the DMRs overlapped with DEGs with ~78% of DEGs being overlapped by a DMR.

Lines 378-380: Using the Angus reference, ~19% (~12,251) of DMRs overlapped a DEG and ~96% of DEGs overlapped with a DMR; ~75% of the DMRs overlapped a putative enhancer region.

In the discussion (lines 431-433), the authors explained the potential reason why there is no directional change found when considering DMC, but there are direction changes when considering DMRs. The authors reasoned that the cause might be that some DMRs are specific to the reference genome. However, this explanation is not as convincing. We think it might be more plausible that the number of tests in the DMC analysis is way larger than the number of tests in the DMR analysis. As a result, when correcting p-values for multiple hypothesis testing, the threshold will be more stringent in DMC analysis than that of DMR analysis, rendering no Cytoscine pass that threshold. The authors should explicitly write what p-value adjustment method is used or justify if the p-value correction method is suitable for the problem setting.

Response: We appreciate the reviewers’ comments regarding the DMR inversion results. We reported no p-values for this result. The point of this section was to illustrate that when using a tiling method as part of DMR identification, the influence of breed-specific CpGs will likely play a role in determining methylation status of that region. For example, methylKit examines the number of Cs reported, the number of Ts reported and the overall coverage at that region. Using a tiling approach (which MethylKit does), it is likely that the CpGs affected by SNPs will be included in these regions. Therefore, some positions will be erroneously reported as unmethylated (high portion of Ts) in the non-reference genome group. We have better explained this reasoning in the discussion at lines 456-459.

Minor points:
The current analysis is purely based on real data, where the underlying true genome responsible for generating the WGBS data remains unidentified. For a comprehensive understanding, validating the conclusion through in-silico simulations is suggested. This involves creating synthetic bisulfite sequencing reads, simulating from BT and BI reference genomes to represent BTBT and BIBI samples, respectively (reciprocal cross-offspring samples can be fabricated via the in-silico mixture of synthetic BTBT

Powered by Editorial Manager® and ProduXion Manager® from Aries Systems Corporation

and BIBI samples). These synthetic reads should then be aligned to the BT and BI reference genomes, respectively. In such simulations, the reference genome serves as the known true genome. Consequently, when comparing downstream analysis results of a particular sample where different reference genomes are used, any observed discrepancies can be attributed to the differences in the reference genomes. This controlled simulation provides a clean environment to establish a theoretical limit on the accuracy attainable when employing the correct or incorrect reference genome, thereby revealing the true significance of selecting the appropriate reference genome. On the other hand, since the genomic origins of the synthetic reads are known, it offers an opportunity to investigate what specific factors (SNPs, SVs, repetitive elements or telomere regions, etc.) in the reference genome and how they contribute to the observed discrepancy.
The authors appreciate this suggestion but argue this would be an entirely new study in itself and is beyond the scope of this work. Moreover, the authors suggest a more appropriate alternative would be to employ long-read sequencing technologies like Oxford Nanopore, where we are able to generate and assemble the true genome of each individual and identify DNA methylation without the risk of DNA damage that can occur using bisulfite based methods.

For the parent-of-origin effects analysis (lines 589-593), the maternal effects are investigated by comparing samples with BIBI dams (BIBI; BTBI) and those with BTBT dams (BTBT, BIBT); the parental effects are investigated in a similar way. The comparison design has an underlying linear assumption that the parental and maternal chromosomes of different breeds have no synergy effects. Under that assumption, when comparing samples with BIBI dams (BIBI; BTBI) and those with BTBT dams (BTBT, BIBT), the parental effects are averaged out, thus all the differences can be attributed to the maternal effects. This assumption should be acceptable in general, but we think a better idea to bypass the assumption is to construct in-silico mixture samples where half of the sequencing reads are sampled from BTBT and half from BIBI; in this scenario, the methylation profile is equally contributed by BT and BI breed (the null case when the parental and maternal effects do not exist). Then, the maternal effects can be investigated by comparing the BTBI to the in silico samples, and the parental effects can be investigated by comparing the BIBT samples to in silico samples. This suggestion is not required to be implemented since it is not the main focus of the manuscript, but it would be interesting to explore.
The authors thank the reviewers for this suggestion and agree it would be interesting to explore. However, as with the previous suggestion, we consider it to be beyond the scope of this study and suggest it would be more appropriate to investigate with long-read sequencing technologies where the paternal and maternal genomes can be more easily disentangled.

For CpG sites filtering (line 594), the authors mentioned the sites were excluded if their coverage are greater than the 99.9th percentile and reasoned that the extremely high coverage is due to the PCR duplicates. However, since the authors already marked the PCR duplicates in the WGBS data processing, the high coverage should no longer be due to PCR duplicates. We speculate it might be due to the repetitive nature of some regions in the genome (or alignment artifacts). Perhaps the authors can check if the genomic coordinates of these extremely high coverage sites are consistent across all the samples in the same breed (if it is due to PCR duplicates, these coordinates might be more stochastic; if it is the nature of the reference genome, then the coordinates might be more enriched/restricted to certain regions)
While the filter for coverage greater than 99.9th percentile may not be due to PCR duplicates, this step is recommended by the authors of MethylKit. We have clarified this in the methods at lines 832-833 ("... site and is a recommend pre-processing step for methylKit [77].")

In supplementary table 1, the WGBS mapping stats should contain more information about the read mapping summary for each sample, e.g., the total number of reads, mapped reads (%), and mapped reads after marking duplicates (%), etc.
We have updated supplementary table 1 to include the total number of reads and reads mapped after deduplicating.

Word suggestions:

Powered by Editorial Manager® and ProduXion Manager® from Aries Systems Corporation

In the subtitle `Breed-specific DMRs show poor association with DEGs`, the term `Breed-specific` is not an accurate expression for what it tries to describe. The term breed-specific means something exists in one breed but not in another, e.g. (the authors used `Breed-specific CpGs` to denote CpG sites in one breed, but not in another in the previous section). It looks like the `Breed-specific` is abused here, where it actually means the regions or genes exist in both breeds, but they are different in breeds. In that sense, the authors could consider changing it to something like `breed-informative` or `breed-distinguishing` or the equivalent.
We have rewritten this header as “DMRs between breeds show limited overlap with DEG promoters”.

We also suggest replacing all `poor association` with `poor overlap` since the association between DMR and DEG is never examined; only the overlap is examined.
We have addressed this in our previous response above.

In the Figure 2B caption, the sentence `The X axis is the first dimension of the logFC, and the Y axis is the second dimension of the logFC` might be inaccurate; should the logFC be logCPM? If not, the authors should explain the definition of logFC.
The authors appreciate the reviewers highlighting this. We have corrected it to logCPM.
Reviewer 2
This manuscript is a well-written and readable account of a study of methylation in two subspecies of cattle - Bos taurus and Bos Indicus. There is a specific focus on the effect of which reference genome assembly is used as the framework for the analyses. The authors demonstrate that the reference genome used can introduce a bias, albeit a relatively modest bias in global terms. Whilst the 2% bias can be considered modest at a whole genome scale, it can be critical when considering individual genes or regulatory sequences.
The average mapping rate of CpG with at least 10% coverage to the Brahman and Angus reference assemblies is reported in S Table 1 (94.95% and 92.49%, respectively), but only the average mapping rate to the Brahman assembly is reported in the main text.
Interestingly, a higher proportion of the Angus (Bos taurus) CpGs with at least 10x coverage map to the Brahman assembly than to the Angus assembly (94.83% and 92.75% respectively on average). In contrast, as one might predict a smaller proportion of the Brahman CpGs with at least 10x coverage map to the Angus assembly than to the Brahman assembly. Some comment on the apparent anomaly in the mapping rate for the Angus reads might be appropriate.
The Angus lacks X chromosome due to the use of trio binning genome assembly method. As a result, reads that belonged to the X chromosome were not mapped. Our analysis focused on the autosomes, hence the sex chromosomes impact was not included.

The authors highlight the bias that may be introduced by using an inappropriate assembly as the framework for epigenetic and other analyses. The coming pangenomes are presented as a potential solution without demonstrating that solution in this particular study. Perhaps a comment on other solutions such as the generation of experimental population specific genome assemblies as a framework for analyses. What will be the impact of the use of long read sequencing data that includes scoring of modified bases in epigenetic analyses?
We have added this to the discussion at lines 468-475. “While DNA methylation analyses between breeds and strains are challenging, exciting solutions are on the horizon. Indeed, with long-read sequencing like Oxford Nanopore becoming more cost-effective and given it has the ability to capture DNA methylation with no additional sample preparation, it is much more likely that researchers will be able to follow the recommendations of Wulfridge, Langmead [15] and examine DNA methylation of individuals with personalised reference genomes. Long read sequencing has the potential to simplify DNA methylation analyses of diverse populations substantially.”

The manuscript is suitable for publication after minor changes.

Given the small number of individuals used in this study the term "breed-specific" should be used with caution. Perhaps the term and its usage in the paper should be explained, complete with caveats, in the introductory text.

Powered by Editorial Manager® and ProduXion Manager® from Aries Systems Corporation

	We have added this disclaimer at lines 221-224. "551 noted that in this study, we consider breed-specific as CpGs that appear in one reference genome and not the other. The limitation of this being neither reference genome likely captures all variation present within the two breeds."<br><br>Historically, a sample size of 20-25 UNRELATED individuals was considered an appropriate sample of a population in breed genetic diversity studies.<br>The number of individuals sampled per breed or crossbreeds in this study is a fraction of this recommended sample size. Moreover, no information is provided on the degree of relatedness between the individuals sampled.<br>We appreciate the reviewer's comments regarding sample size. Taurine and indicine cattle are known to have high phenotypic and genotypic divergence even when sample sizes are small. Moreover, we based our sample size of 3 males and 3 females per group on Liu et al., 2021. We have added information regarding the relatedness of the individuals at line 551-552 (". Fetuses were sired by three BTBT bulls and 2 BIBI bulls.").<br><br>The WGBS and transcriptomics data used for the analyses are available in the public domain at PRJNA626458.<br><br>The gene symbol nomenclature for cattle follows the nomenclature style used for human genes. Thus, all gene symbols should be fully capitalised as well as italicised. We have amended the manuscript accordingly.
**Additional Information:**	
**Question**	**Response**
Are you submitting this manuscript to a special series or article collection?	No
**Experimental design and statistics**<br><br>Full details of the experimental design and statistical methods used should be given in the Methods section, as detailed in our Minimum Standards Reporting Checklist. Information essential to interpreting the data presented should be made available in the figure legends.<br><br>Have you included all the information requested in your manuscript?	Yes
**Resources**<br><br>A description of all resources used, including antibodies, cell lines, animals and software tools, with enough information to allow them to be uniquely identified, should be included in the Methods section. Authors are strongly encouraged to cite Research Resource Identifiers (RRIDs) for antibodies, model organisms and tools, where possible.	Yes

Have you included the information requested as detailed in our Minimum Standards Reporting Checklist?	
**Availability of data and materials**<br><br>All datasets and code on which the conclusions of the paper rely must be either included in your submission or deposited in publicly available repositories (where available and ethically appropriate), referencing such data using a unique identifier in the references and in the “Availability of Data and Materials” section of your manuscript.<br><br>Have you have met the above requirement as detailed in our Minimum Standards Reporting Checklist?	Yes

Powered by Editorial Manager® and ProduXion Manager® from Aries Systems Corporation

Manuscript Click here to access/download;Manuscript;Methylation_paper_clean_revisio

DNA methylation analysis to differentiate reference, breed and parent-of-origin effects in the bovine pangenome era

Authors:

Callum MacPhillamy[1], Tong Chen[1], Stefan Hiendleder[1,2], John L. Williams[1,3], Hamid Alinejad-Rokny[4], Wai Yee Low[1]

[1]The Davies Research Centre, School of Animal and Veterinary Sciences, University of Adelaide, Roseworthy, SA 5371, Australia

[2]Robinson Research Institute, The University of Adelaide, North Adelaide, SA 5006, Australia

[3]Department of Animal Science, Food and Nutrition, Università Cattolica del Sacro Cuore, 29122 Piacenza, Italy

[4]BioMedical Machine Learning Lab, The Graduate School of Biomedical Engineering, UNSW, Sydney, NSW 2052, Australia

Abstract

Background

Most DNA methylation studies have used a single reference genome with little attention paid to the bias introduced due to the reference chosen. Genetic variation, including single nucleotide polymorphism (SNPs) and structural variants (SVs), can lead to differences in methylation sites (CpGs) between individuals of the same species. We analysed whole genome bisulfite sequencing (WGBS) data from the fetal liver of Angus (*Bos taurus taurus*), Brahman (*Bos taurus indicus*) and reciprocally crossed samples. Using reference genomes for each breed from the Bovine Pangenome Consortium, we investigated the influence of reference genome choice on the breed- and parent-of-origin effects in methylome analyses.

Results

Our findings revealed that about 75% of CpG sites were shared between Angus and Brahman, ~5% were breed-specific, and ~20% were unresolved. We demonstrated up to ~2% quantification bias in global methylation when an incorrect reference genome was used. Furthermore, we found that SNPs and SVs were 14-fold (p-value $< 5 \times 10^{-324}$) and 1.18-fold (p-value $< 5 \times 10^{-324}$) higher in CpGs, respectively, compared to the rest of the genome. We found a poor association between differentially methylated regions (DMRs) and differentially expressed genes (DEGs) and suggest that DMRs may be impacting enhancers that target these DEGs. DMRs overlapped with imprinted genes, of which one, *DGAT1,* which is important for fat metabolism and weight gain, was found in the breed-specific and sire-of-origin comparisons.

Conclusions

This work demonstrates the need to consider reference genome effects to explore genetic and epigenetic differences accurately and identify DMRs involved in controlling certain genes.

Keywords

Bisulfite sequencing, methylation, CpG, structural variants, *Dgat1*, differentially methylated region, bovine pangenome

Background

DNA methylation is a key epigenetic modification that plays a vital role in regulating gene expression, repression of transposable elements, and parental chromosome specific regulation through genomic imprinting and X-chromosome inactivation [1, 2]. In mammals, DNA methylation primarily occurs at C-phosphate-G dinucleotides (CpGs) [3, 4]**.** DNA methylation influences gene expression either by recruiting proteins involved in gene repression or by blocking transcription factor binding sites (TFBSs) within promoter regions [5]. Hypomethylation of a promoter has been associated with the increased expression of the corresponding gene [6]. However, recent work has shown that promoter hypermethylation can also lead to gene expression [7]. The relationship between DNA methylation and gene expression is complicated by the role of enhancer methylation in regulating gene expression [8, 9]. In the presence of high DNA methylation, enhancers have been observed to be associated with high levels of the histone modification H3K27ac [10], which is often associated with active gene transcription [11-14].

Most DNA methylation studies have used a single reference genome with little or no knowledge of the impact of reference genome choice on the interpretation of methylome differences. The choice of reference genome has been shown to have an impact on DNA methylation analyses, with up to a nine per cent bias reported when the incorrect reference is used (Wulfridge, Langmead [15]. Using a single reference genome has been shown to bias read mapping in favour of reads with high similarity to the reference [16-20]. This bias occurs because reads containing non-reference alleles or regions that are divergent from the reference either align poorly, align to the wrong genomic region, or fail to align. This reference bias has been shown to affect analyses of cattle breeds [21, 22], humans [17, 23], and sheep [18].

The majority of mammalian methylation occurs in the CpG context. Consequently, a single nucleotide polymorphism (SNP) can remove a methylation site, thus introducing a reference bias if the individuals being studied do not possess the same SNPs as the individual used to generate the reference. In addition to SNPs, structural variations (SVs) among individuals may remove or introduce CpG sites. The disparity between CpG sites can confound analyses by identifying a methylated CpG in one individual when another individual has no CpG at that position. As a result, SNPs and SVs can both introduce bias, as reads may be unambiguously assigned in duplicated regions not found in the reference and mismatches in reads can result in the loss of some reads. Moreover, if individuals have insertion SVs that carry CpG sites, reads that originate from the insertion/deletion (indel) regions can only be mapped if the complete sequence data for the population is available. We consider SNPs and SVs that alter CpGs as genetic changes with potential effects on epigenetic regulation. We use the term ‘genetics of epigenetics’ to describe this phenomenon.

As more genomes for a given species become available, the research community is gradually shifting toward using pangenomes to account for genetic variation within a population more accurately. A pangenome is a collection of the genomes of multiple individuals, representing all genetic variation within that population and is thus a more accurate way to represent genetic diversity than a single reference genome [24]. Current pangenome projects include human [24, 25], cattle [26], and maize [27]. As genetic differences within a population can result in CpG differences, these pangenomes provide a valuable resource to study DNA methylation changes between diverse groups of individuals of the same species.

The two main lineages of modern cattle breeds are generally accepted to have been derived from two separate domestication events of the wild auroch (*Bos primigenius*) [28]. The first domestication event occurred in the Fertile Crescent around 10,000 years ago and gave rise to *Bos taurus taurus* from the wild auroch, *B. p. primigenius* [29-31]. A second domestication event occurred in the Indus Valley, ~1,500 years later, from *B. p. nomadicus*, which separated from *B. p. primigenius* around 250-330,000 years ago [32] and gave rise to *Bos taurus indicus*. The subspecies are referred to here as taurine and indicine cattle, respectively [28], where the Angus breed represents taurine cattle, and Brahman is representative of indicine cattle. Angus and Brahman have contrasting phenotypes, e.g., Angus have been bred for meat production traits [33], whereas Brahman have superior heat and disease tolerance traits [34, 35]. DNA methylation differences may partly be responsible for the phenotypic differences between these two breeds.

As expected from their domestication history, Angus and Brahman cattle represent genetically highly diverged subspecies [36, 37]. However, as they produce fertile offspring when mated [38], they are an appropriate model to investigate the impact of using a single reference genome on methylome analysis of two genetically diverse populations. We have previously produced high-quality haplotype-resolved reference genomes for Angus and Brahman [39], which are genomes included in the Bovine Pangenome Consortium project [26] and are used in the present study.

Breed-specific differences in CpGs may occur due to a SNP, such as those caused by spontaneous deamination [40, 41], or may result from SVs. A single SNP affecting a CpG site has been shown to drastically alter the methylation state of the *IGF2* gene in pigs, leading to changes in muscle development [42]. SVs have been associated with decreased methylation in cancers [43] and with changes in the methylation of the kappa opioid receptor (*KOR)* promoter associated with KOR dysfunction and schizophrenia [44].

Parent-of-origin effects (POEs) occur when only one allele is expressed, and the phenotype in the offspring may depend on which parent contributed the expressed allele [45]. Reciprocal crossing is necessary to elucidate how each parent contributes to a particular phenotype. POEs have been observed in hybrids of mice [46], cattle [47] and pigs [48], and there is increasing evidence that fetal development is influenced by POEs [49-53]. Given the similarity in gestation period between cattle and human and the single fetus with similar development trajectory, cattle are an attractive model species to study human reproductive and developmental biology [54-57].

To investigate the potential impact of reference genome choice on methylome analyses and to improve our understanding of the genetic and epigenetic factors driving the phenotypic differences between cattle subspecies, we used WGBS data from 24 fetal liver samples of purebred Brahman and Angus cattle and their reciprocal-crosses to perform a comprehensive assessment of the impact of reference genome choice on differential methylation and gene expression. This study serves as an example of how to investigate epigenetic differences between breeds, strains, and populations within species and informs about reference genome effects on the interpretation of methylome analyses.

Results

Mapping of WGBS data and calling CpG

Each of the 24 samples representing the four genetic groups (Fig 1A; S. table 1) was sequenced for WGBS analysis to at least 30X coverage and then mapped separately to the Brahman and Angus genomes (Fig 1B). An average mapping rate of ~95% was achieved when reads were mapped from each sample to the Brahman reference genome (Table 1; S. table 1). All samples had at least 10X coverage for 93% of the Brahman sequence. Using the Angus reference, all samples had 10X coverage for at least 90% of the sequence (Table 1; S. table 1).

We performed all analyses twice for each reference genome, first using all CpGs with ≥10X in each reference genome and again where we retained only CpG sites with ≥10X that we could confidently assign as being shared between both breeds. Between 85% and 88% of autosomal CpG sites had coverage ≥ 10X when considering all CpG sites on both the Brahman and Angus reference and shared CpG sites (Table 1; S. table 2). Median coverage of CpG sites across all samples ranged from 25-34X regardless of reference and CpG sites considered (i.e., shared or all) (Table 1; S. table 3).

Table 1. Mapping statistics of Angus and Brahman reference genomes.

	Angus	Brahman
Mapped reads*	1,455,481,398	1,457,794,807
Duplication rate (%)*	10	14
CpGs with ≥ 10X coverage in all samples	22,116,287	21,962,589
CpG coverage*	30	30

* Mean of all samples.

Clustering of genetic groups

Comparing the methylation patterns between the genetic groups, we found that samples within a genetic group were more similar to each other than with samples from other groups. For example, samples from the BTBT group had higher correlations with other BTBT samples than BIBI samples. BTBT had the highest within-group Pearson correlations (*r* between 0.81 and 0.88) (S. figure 1). The samples that were least correlated with one another were those belonging to BTBT and BIBI, with correlations between 0.75 and 0.78. Samples from the reciprocal cross groups (BIBT; BTBI) had similar correlations with other samples within their own group (*r* between 0.81 and 0.83) as well as with samples from the alternative reciprocal cross (*r* between 0.80 and 0.83). Overall, correlations were high within each genetic group ($r \geq 0.8$) (S. figure 1).

We performed a principal component analysis of the 24 samples using CpG sites covered by at least 10 reads in all samples (Fig 2A). BTBT and BIBI formed distinct clusters distant from one another, with the two hybrid genetic groups clustering much closer together and between the two parental genetic groups. Nevertheless, the hybrid groups were clearly separated on the PCA plot (Fig 2A). The separation of groups we observed from the methylation data was similar to that seen for the gene expression data (Fig 2B).

Overview of DNA methylation patterns

Samples had global mean CpG methylation of between 47-62%, with most samples ranging from 49-54% (Figure 3A; S. table 4). Mean exon CpG methylation was 45-54% for all samples, with most samples ranging from 48-54% methylation (S. table 5). The 5' UTRs and promoter regions had the lowest mean CpG methylation percentage across all samples, between 10-13% and 24-31%, respectively (S. table 5). The intergenic regions displayed mean methylation levels that ranged from 47 – 65% (S. table 5), with most samples ranging from 49-57%, similar to the global mean. The introns exhibited slightly higher methylation levels, with means ranging from 50-64% (S. table 5), and most samples in the 53-59% range. The 3' UTRs revealed the highest overall CpG methylation levels, 57-69% (S. table 5). Lastly, the predicted enhancers, according to MacPhillamy, Alinejad-Rokny [58], exhibited CpG methylation levels ranging from 42-53%, with most samples within 43-48% methylated (S. table 5). Similar methylation patterns were observed using only shared CpGs in exons, 5'UTRs, intergenic, introns, promoters, predicted enhancers, and 3'UTRs, regardless of the reference genome used.

Shared and breed-specific CpGs

We were able to confidently identify 74-75% of CpGs in the Brahman and Angus genomes that were shared between the two breeds (Table 2; S. table 6). We found that around four per cent of CpG alignments contained a SNP between reference genomes (S. table 6; S. figure 2), i.e., were breed-specific. About 22% of CpGs could not be confidently assigned as shared or breed-specific and so were not considered in the shared CpG analysis. By definition, breed-specific regions with CpG sites did not align when the other breed genome was used as the reference. We found ~1% of such CpG sites. In total, the SNP change and breed-specific categories of CpG sites constituted 4.7% and 4.9% of CpGs between the Angus and Brahman reference genomes, respectively, and were considered breed-specific. It should be noted that in this study, we consider breed-specific as CpGs that appear in one reference genome and not the other. The limitation of this being neither reference genome likely captures all variation present within the two breeds.

Table 2. Number of CpGs in the Angus and Brahman reference genomes.

	Angus	Brahman
Total CpGs[A]	25,712,300	25,799,151
CpGs aligned to other reference[B]	25,209,966	25,228,509
CpGs shared in other genome[C]	18,813,726	18,781,688
CpGs affected by SNP[D]	993,318	1,003,167
Unresolved CpGs[E]	5,402,922	5,443,654

[A] Total number of CpGs present within the genome.
[B] Number of CpGs that could be aligned from one genome to the other using Minimap2 [59].
[C] Number of CpGs in B that were CpGs in both species.
[D] Number of CpGs in B that were a CpG in one species but are no longer CpGs in the other.
[E] Number of CpGs in B that could not be confidently assigned as either shared or a SNP.

Enrichment of SNPs affecting CpG sites

Using the autosomal SNPs identified by Minimap2 and PAFtools.js [59], we observed that Brahman and Angus autosomal sequences differ by an average of ~0.4% (S. table 7). SNPs in CpG sites were found to be enriched by ~14 times compared to the genome-wide average (binomial test, p-value < 5×10^{-324}), which means there is a higher level of divergence between Angus and Brahman at CpG sites than at other autosomal sites.

Looking more closely at the CpG SNP changes, we found that most (~81%) of the CpG SNP were either C to T or G to A changes (S. figure 2), which is very similar to the number of CpG SNP changes detected in humans (80.7%) [60]. The remaining SNP changes combined comprised ~19% of the total observed mutations at CpG sites (S. figure 2; S. table 6).

Increased number of CpGs within structural variants

Next, we tested whether CpGs were enriched in SVs compared to the rest of the genome. Using the Brahman genome as the reference, we observed 16,011 SVs between Brahman and Angus, making up ~15Mb of sequence. We observed 1.18-fold more CpGs within SVs than in non-SVs (binomial test, p-value < 5×10^{-324}). When considering CpGs affected or introduced by SNPs and SVs, the CpG mutation rate is approximately 6.7% between Brahman and Angus compared to the genome-wide mutation rate of around 1%.

Choice of reference genome influences methylome results

We examined the CpG methylation differences between each sample when mapped to Angus and Brahman reference genomes and observed a statistically significant difference in CpG methylation levels in all samples when using all CpGs with $\geq$10X coverage (paired Wilcoxon test, adjusted p-value < 0.05) (Table 3; S. table 8). When only considering the shared CpGs, we observed a significant quantification bias in most samples, though it was much smaller (paired Wilcoxon test, adjusted p-value < 0.05) (Table 3; S. table 8). When we pooled all CpG methylation values for a given group and compared the Brahman and Angus references, we observed a statistically significant quantification bias in all four groups (paired Wilcoxon test, adjusted p-value < 0.05) (Figure 3A; Table 3; S. table 8). When comparing only the shared CpGs, we observed a much weaker quantification bias between genomes, though it was still significant at the group level (paired Wilcoxon test, adjusted p-value < 0.05) (Figure 3B; Table 3; S. table 8). When comparing global CpG methylation differences between samples mapped to Brahman versus those mapped to Angus, the largest quantification bias was ~2% for BTBT. The other quantification biases were ~0.8%, ~0.7% and ~0.3% for BTBI, BIBT and BIBI samples, respectively (Figure 3A; Table 3). When using only shared CpGs, the quantification bias was reduced to ~0.6%, ~0.5%, ~0.4%, and ~0.2% in BTBT, BTBI, BIBT and BIBI, respectively (Figure 3B; Table 3).

Next, we examined whether the shared CpGs exhibited correlated methylation levels regardless of the reference genome used. For example, we wanted to determine whether a given CpG in the Brahman reference has the same methylation percentage as the corresponding CpG in the Angus reference for a given sample. We observed R^2 scores of around 0.99 for groups (Figure 3C), with most CpGs exhibiting less than 10% difference in methylation between the reference genomes. Interestingly, we observed several CpGs that appeared sensitive to reference genome choice. For example, 264,023 CpGs had an absolute methylation difference of least 10%, with 429 CpGs having an absolute difference of at least 50% depending on the reference genome used (S. table 9).

To further investigate the influence of the reference genome on downstream analyses, we compared DMRs identified by the two reference genomes to evaluate if the direction of methylation changed, i.e., hypermethylated became hypomethylated and vice versa. DMRs from Angus were mapped to the Brahman reference and we found approximately 12% (28,922) of Angus DMRs overlapped with Brahman DMRs by at least 90% of their length. Of these DMRs that mapped to the Angus reference, 3,575 showed changes in methylation direction when mapped to the Brahman reference (S. table 10). That is, a DMR that was observed as hypomethylated in Angus samples relative to Brahman when mapped to the Angus reference was observed to be hypermethylated in Angus relative to Brahman when mapped to the Brahman reference. We observed similar numbers (3,581) when lifting DMRs from Brahman to Angus (S. table 10). There were no methylation direction changes when we considered differentially methylated cytosines (DMCs).

Breed-specific CpGs show distinct methylation patterns

Looking more closely at the breed-specific CpGs, we first determined whether a given breed-specific CpG was methylated or not. We binned individual CpGs into “unmethylated” (CpG methylation ≤ 35%) and “methylated” (CpG methylation ≥ 65%). CpGs that were between 35% and 65% were considered “hemimethylated” and were excluded from this analysis. We repeated this with “unmethylated” thresholds of ≤ 25% and “methylated” thresholds of ≥ 75%, with the CpG methylation values in between being considered hemimethylated [61]. We only considered breed-specific CpGs with at least 10X coverage in all purebred samples mapped to their respective genome. We then randomly sampled 1000 CpG sites for 100 iterations, recording the number of “methylated” and “unmethylated” CpGs for each breed. At both the 25 and 75%, and 35 and 65% thresholds, we observed significantly more Brahman-specific CpGs as hypomethylated than hypermethylated (Mann-Whitney U-test, p = 2.5×10^{-34}) (Mann-Whitney U-test, p = 2.5×10^{-34}), respectively Interestingly, we observed the inverse when considering Angus-specific CpGs; significantly more Angus-specific CpGs were hypermethylated than hypomethylated (Mann-Whitney U-test, p = 3.0×10^{-34}) (Mann-Whitney U-test, p =2.5×10^{-34}), respectively (Figure 3D-E).

DMRs between breeds show limited overlap with DEG promoters

As we observed a quantification bias when using all CpGs mapped against each reference genome, we restricted breed-specific and POE analyses to those CpGs identified as shared. Additionally, we examined the number of DMRs at the 25% and 50% difference thresholds, i.e., more stringent thresholds for calling DMRs which substantially reduced the numbers (S. table 11). Given that minor changes of less than 10-15% in methylation have been observed to influence gene expression and phenotype [62, 63], we used a difference threshold of 10% to interpret the results.

Using Brahman as the reference, we identified 123,602 DMRs and 1,549 DEGs (S. table 11; S. table 12). Of the 123,602 DMRs observed, ~19% (23,575) overlapped with the surrounding region of the significant DEGs, with most (~77%) falling within the putative-enhancer region. Only 0.3% of the DMRs overlapped with promoters of DEGs, despite 99% of significant DEGs being overlapped by a DMR. When the Angus reference was used, of the 125,544 DMRs identified, ~25% (31,252) of those overlapped with a DEG. Most (~73%) of these DMRs fell into the putative enhancer region, while only 0.3% of the DMRs overlapped with a DEG promoter, despite substantially more (1,872) DEGs observed (S. table 12) and 99% overlapping with a DMR.

We then examined the overlap of DMRs and imprinted genes, first using Brahman as the reference. Here, ~1% (1,182) of the DMRs identified between BIBI and BTBT overlapped 79 imprinted genes. Only one imprinted gene, Par-6 family cell polarity regulator gamma (*PARD6G*), did not overlap with any DMR. Most DMRs (~79% of the 1,182) that overlapped an imprinted gene fell into putative enhancer regions. Five imprinted genes were significantly differentially expressed when comparing BIBI and BTBT (Table 4). These genes were DS cell adhesion molecule (*DSCAM*), neuronatin (*NNAT*), Lin-28 homolog B (*LIN2B*) and protein phosphate 1 regulatory subunit 9A (*PPP1R9A*). *DSCAM* and *NNAT* had higher expression in BTBT, and the remaining three DEGs, *DGAT1*, *LIN2B* and *PPP1R9A*, had higher expression in BIBI. We again observed five imprinted DEGs using the Angus reference; however, one gene was a novel gene. The remaining genes (*NNAT*, *LIN28B*, *DGAT1* and *PP1R9A*) showed the same expression pattern as when mapped to Brahman (Table 5).

Dam-of-origin methylation shows less overlap with imprinted genes

To investigate the dam-of-origin effects (DOEs), we compared samples with Brahman dams (BIBI and BTBI) with those with Angus dams (BTBT and BIBT). Using the Brahman genome as the reference, 457 DEGs were identified in the DOE comparison. Around 6% (1,236) of the DMRs overlapped with DEGs, with ~78% of DEGs being overlapped by a DMR. Most DMRs (~910) fell into the putative enhancer region; 0.24% of DMRs overlapped with a DEG promoter (S. table 11; S. table 12). There were 52 imprinted genes that overlapped with ~1% (190) of the DMRs identified in the comparison. Of the 190 DMRs that overlapped with an imprinted gene, 128 DMRs overlapped with the putative enhancer region. Four imprinted genes were significantly differentially expressed (Table 4). Zinc finger CCCH-type containing 12C (*ZC3H12C*), *PPP1R9A* and *LIN28B* had higher expression in samples with Brahman mothers. Using Angus as the reference, we observed 1,254 (~5%) DMRs overlap with the 358 DEGs and ~88% of DEGs covered by a DMR. Most (870) of these DMRs fell into the putative enhancer regions. Three imprinted DEGs (*ZC3H12C*, *PPP1R9A* and *LIN28B*) were identified using the Angus genome as the reference (Table 5).

Sire-of-origin methylation may be driving differential gene expression

To investigate the sire-of-origin effects (SOEs), we compared samples with Brahman sires (BIBI and BIBT) with those with Angus sires (BTBT and BTBI). Using the Brahman reference, we identified 62,056 DMRs and 1,190 DEGs in the sire group comparison using shared CpGs (S. table 11; S. table 12). There were 63,516 DMRs identified using the Angus reference, but substantially more DEGs (1,568) were identified (S. table 12). Around 12% (7,840) of the DMRs overlap with a significant DEG; ~93% of DEGs were overlapped by a DMR, with most (80% of the 7,840) of those overlaps occurring in a putative enhancer region. Using the Angus reference, ~19% (~12,251) of DMRs overlapped a DEG and ~96% of DEGs overlapped with a DMR; ~75% of the DMRs overlapped a putative enhancer region. Around 0.2% of DMRs overlapped with a DEG promoter when using either the Brahman or Angus as the reference.

We observed 73 imprinted genes that overlapped DMRs identified between the two different sire groups. Less than 1% (586) of DMRs overlapped the 73 imprinted genes, with most (~73% of 586) occurring in the putative enhancer region. Eight imprinted genes were significantly differentially expressed and overlapped with a DMR (Table 4). These genes were *DSCAM* 5-hydroxytryptamine receptor 2A (*HTR2A*), *NNAT*, *DGAT1*, necdin MAGE family member (*NDN*), and tissue factor pathway inhibitor 2 (*TFPI2*). *DSCAM*, *NNAT*, *NDN, MKRN3* and *TFPI2* had higher expression in samples with Angus sires, with the other genes (*HTR2A*, *DGAT1*, *SLC22A18*) being more highly expressed in samples with Brahman sires. *SLC22A18* showed high expression in the Brahman sire group, and *MKRN3* showed higher expression in the Angus sire group, but neither overlapped with any DMRs. Seven significantly differentially expressed imprinted genes were observed when using the Angus reference (Table 5). In this case, the seven significantly differentially expressed imprinted genes with DMR overlap were *DSCAM*, *HTR2A*, *NNAT*, *MKRN3*, *NDN*, and *SLC22A18*. *DSCAM*, *NNAT*, *MKRN3* and *NDN* had higher expression in samples with an Angus sire. The remaining three genes (*HTR2A*, *DGAT1* and *SLC22A18*) had higher expression in samples with a Brahman sire. *DGAT1* did not overlap any DMRs when using the Angus reference.

Discussion

In the present study, we observed genome-wide CpG methylation correlations among replicates that ranged from 75% to 82% between groups and from 81% to 87% within groups. These correlations were similar to a recent study in mice where genome-wide CpG methylation correlations among replicates ranged from 73% to greater than 80% [64]. Moreover, we observed levels of liver global CpG methylation between 47-62% in the present study, which is similar to previous studies of human [65], mouse [64] and cattle [66].

Mapping statistics can provide an insight into how the choice of reference genome will affect downstream analyses [67]. However, we observed negligible differences in raw mapping statistics regardless of whether the Angus or Brahman reference genomes were used. Additionally, the global methylation quantification bias observed was less than 2%, depending on the reference genome used. This quantification bias is lower than the 7-9% quantification bias found in the mouse genome, depending on the reference genome used (Wulfridge, Langmead [15]. The extent of this bias is influenced by the divergence between reference genomes and whether the breed-specific CpGs tend to be hypo- or hypermethylated. Brahman and Angus have a CpG divergence of ~4%, whereas the mouse genomes analysed by Wulfridge, Langmead [15] had a CpG divergence of 10.7%. The bias we observed was greatest in the BTBT samples when the Brahman genome was used as the reference, most likely because the Angus-specific CpG sites tended to be hypermethylated. Conversely, the quantification bias was lower in the other genetic groups, possibly due to the hypomethylation in Brahman-specific CpG sites.

Spontaneous deamination of methylated CpG to TpG is the most common dinucleotide mutation in the mammalian genome [40, 41]. We observed around 81% of SNPs between Brahman and Angus as being C-T or G-A mutations. A recent study observed 34,677 SNPs affecting CpG sites between indicine and taurine genomes [68]. The difference in the number of SNPs between the two studies is likely due to Capra, Lazzari [68] having used reduced representation bisulfite sequencing with substantially lower coverage than the present study and that they only considered SNPs that affected CpG sites. When differential methylation analysis is performed, a breed that has lost the C (mutated to T) will be reported as having 0% methylation at that site when, in fact, there is no CpG present. This incorrect identification of an unmethylated site can then severely impact the interpretation of results.

SVs have been associated with various traits in humans, including HIV-1 susceptibility [69], autism [70-72] and carcinogen metabolism [73]. In livestock, SVs have been implicated in diverse traits ranging from horn (polled) status [74, 75] to bulldog calf syndrome [76]. The SVs between Brahman and Angus have significantly more CpGs than the background genome, potentially introducing CpGs with important regulatory effects. However, due to their presence in only one subspecies, a single reference genome will fail to account for these breed-specific CpG sites. Therefore, the phenotypic differences between the two breeds may be influenced by CpGs that cannot be compared accurately with a single reference genome if they are in breed-specific regions.

We observed relatively few changes in methylation direction, and these were likely to be an artifact of how the genome was tiled and possible erroneous alignments in the coordinate conversion. A common step of some DMR callers is to perform window tiling of the genome to identify DMRs or to enable analysis when coverage is low [77-79]. SNPs and SVs can potentially complicate analyses when genome tiling is used to identify DMRs, as a single reference genome cannot account for these variants. Although we observed no directional changes when considering DMCs, it is possible that SNPs impacting CpGs in otherwise shared regions affected the quantification of DMRs. For example, MethylKit uses a genome tiling approach to identify DMRs and so any mutation that impacts a CpG site, particularly spontaneous deamination (C > T), is likely to erroneously identify a region as hypomethylated in the group that has the spontaneous deamination when in fact, no CpG exists in that group. As such, researchers should be careful when using genome tiling in methylation analyses that compare breeds, strains or populations. Moreover, we demonstrated several CpG sites that appear sensitive to reference genome choice, differing by more than 10% between the two genomes. It is likely that these CpG sites would confound downstream analysis, especially if they overlap with regions of interest, such as cis-regulatory elements. Thus, this demonstrates that quantification bias can remain even when attempting to control for reference genome differences.

While DNA methylation analyses between breeds and strains are challenging, exciting solutions are on the horizon. Indeed, with long-read sequencing like Oxford Nanopore becoming more cost-effective and given it has the ability to capture DNA methylation with no additional sample preparation, it is much more likely that researchers will be able to follow the recommendations of Wulfridge, Langmead [15] and examine DNA methylation of individuals with personalised reference genomes. Long read sequencing has the potential to simplify DNA methylation analyses of diverse populations substantially.

Most DMRs identified in this study were not associated with DEGs. However, of the DEGs that overlapped with a DMR, there was a tendency for the overlap to occur more frequently in the putative enhancer region than in promoters or DEG bodies. This trend suggests that differential methylation of enhancers may impact gene expression differences in bovine fetal liver. Indeed, a growing body of evidence suggests that enhancer methylation is important in embryonic and fetal development [64, 80-82].

There were more significant DEGs when mapping to the Angus reference than the Brahman reference. Interestingly, genes that were DE using the Brahman reference were not always DE when using the Angus reference. The choice of reference genome has been shown to impact differential expression analysis in rice [83], bacteria [84] and human [85, 86] when using short-read RNA-seq. When the reference genome better represents the individuals being studied, more reads can be uniquely aligned to the correct position, providing a more accurate estimate of gene expression.

We identified several interesting DEGs associated with DMRs, particularly imprinted genes. Among these was *DGAT1,* which is involved in fat metabolism in milk production [87], feed conversion and adipogenesis [87, 88]. Several studies have investigated the role of *DGAT1* in weight gain [89, 90]; expression of *DGAT1* is necessary for weight gain, especially when the caloric density of food is high [90]. We found differential expression of *DGAT1*, with higher expression in Brahman than Angus (BIBI vs BTBT) and when Brahman was the sire (BIBI, BIBT vs BTBT, BTBI). Taken together, the comparison of the breed and sire of origin suggests that the breed of the sire may be an important determinant in the expression of this gene. Higher *DGAT1* expression may result from adaptation to poor feed quality; e.g. Elzo, Riley [91] observed better feed conversion efficiency in Brahman compared to Angus and Brahman x Angus cattle. Regulation of *DGAT1* expression may occur via DNA methylation, as there is a DMR ~42kb downstream of the transcription start site, which was identified in both the breed-specific and SOE comparisons.

Parent-of-origin DMRs may change how cis-regulatory elements interact with target genes and influence gene expression in the offspring [45, 92]. It has been observed that parent-specific methylation can alter the cis-regulatory landscape around certain genes, such as *IGF*2 [93, 94]. DMRs may influence the DEGs and, ultimately, help drive the differences in phenotype. However, to confidently assign gene expression and DMRs to a particular parent, long-read sequencing [95] is needed to identify variations that link the sequences to the parent of origin. Additionally, the use of reciprocal crosses will enable one to investigate if a combination of breed and sex of the parent impacts which transcript is expressed.

SNPs and SVs have been shown to complicate and bias analyses in several studies [15, 83-86]. In our analysis, we observed an enrichment of SNPs affecting CpGs between Brahman and Angus. Capra, Lazzari [68] also reported a higher frequency of breed-specific SNPs around DMCs in a study of indicine and taurine cattle. This finding suggests that genetic differences between the two breeds may contribute to epigenetic variations. Using individual animal genomes in the study to account for genetic variations, as Wulfridge, Langmead [15] suggested, would enhance the accuracy for each individual. However, despite decreasing sequencing costs, the cost will likely be prohibitive in most livestock contexts. A possible solution was explored in a recent study comparing methylation in taurine and indicine cattle [68]. Here, the authors used genotyping by sequencing to exclude SNPs affecting CpG sites from the analysis [68]. While this simplifies downstream analysis, it may also remove CpGs involved in the phenotypic differences between the two breeds, representing a limitation of the present study and that of Capra, Lazzari [68]. An alternative approach to using single reference genomes is the utilisation of pan-genomes, which encompass the majority of variations within the population [20, 96]. This feature is particularly important in the context of DNA methylation studies where, demonstrated in our study, SNPs at CpG sites can exert substantial effects on local methylation information.

Conclusions

This study generated a substantial WGBS dataset derived from two phenotypically diverse cattle breeds which are representative of the two cattle subspecies and highlighted the importance of reference genome choice in methylation analyses. Our findings suggest that the DMRs may primarily exert their influence on enhancer elements rather than promoters. We also identified 11 genes that might be under DMR control. The results underscore the advantages of using the appropriate reference genome for the data set and provide additional evidence supporting the incorporation of genome graphs to improve analyses of populations with high genetic divergence.

Methods

Study Animals and Sample Collection

All animal experiments and procedures described in this study complied with Australian guidelines, approved by the University of Adelaide Animal Ethics Committee and followed the ARRIVE Guidelines (https://arriveguidelines.org/) (Approval No. S-094-2005). Liver tissue samples from concepti were the same as those described in Liu, Tearle [97]. Briefly, the parents were purebred Angus (*B. t. taurus*) and purebred Brahman (*B. t. indicus*), herein denoted as BT and BI. Fetuses were sired by three BTBT bulls and 2 BIBI bulls. Primiparous females and their fetuses were ethically sacrificed at day 153±1 of gestation. Concepti were dissected, and tissue samples snap-frozen in liquid nitrogen and stored at -80°C until further use. Liver samples from three female and three male individuals from each of the four genetic combinations: BT x BT, BT x BI, BI x BT, and BI x BI were used.

DNA extraction and sequencing

DNA was extracted from frozen fetal liver tissues using Qiagen® Dneasy® Blood & Tissue Kit following the manufacturer's instruction and sent to BGI Hong Kong, China, for WGBS library preparation and sequencing. Bisulfite conversion was performed using the Zymo Research™ EZ DNA Methylation™ - Gold Kit (D5005). All samples were sequenced in a single batch, and each sample was sequenced to ~30X coverage using the BGI DNB-seq.

RNA was extracted from frozen fetal liver tissues using Illumina® RiboZero Gold kits following the manufacturer's instruction and prepared for Illumina RNA-seq short-read sequencing. The RNA-seq protocol and data availability (GEO accession number: GSE148909) have been described in our previous work [97].

The same tissue samples were used in both the RNA-seq and WGBS. Individual sample names and their corresponding genetic group are given in Supplementary Table 11.

WGBS mapping

WGBS reads were mapped using the MethylSeq Nextflow pipeline (v. 1.6.1) [98] with the '—zymo' trimming parameter. The reads were first checked for quality with FastQC (v. 0.11.9) (https://www.bioinformatics.babraham.ac.uk/projects/fastqc/), then adapters were trimmed using Trim Galore (v. 0.6.6) (https://github.com/FelixKrueger/TrimGalore), and the reads were reassessed for quality post-trimming. Trimmed reads passing qvalue $\geq$ 20 were mapped to the Brahman (GCA_003369695.2) and

Angus (GCA_003369685.2) genomes [39] using BWA-Meth (0.2.2) (arXiv:1401.1129). The non-pseudo autosomal region of the Angus Y chromosome was added to the Brahman reference. This step enabled us to include the Y chromosome sequence whilst avoiding duplication of the pseudoautosomal region. Both Brahman and Angus chromosome sequences were reorientated to match the orientation of ARS-UCD1.2 chromosomes [99]. After sorting the alignment files with SAMtools (v. 1.11) [100], duplicates were marked with Picard (v. 2.25.4) (https://broadinstitute.github.io/picard/). Bam file quality control was performed with the bamqc function from qualimap (v. 2.2.2d) [101] by setting the '-gd' parameter to HUMAN. Methylation calls were extracted using MethylDackel (v. 0.5.2) (https://github.com/dpryan79/MethylDackel) extract with the parameter '—minDepth 10' and output in MethylKit [77] format ('—methylKit') and a more generic cytosine report ('—cytosine_report'). In-house scripts were used to convert the MethylDackel output for use with DNMTools https://dnmtools.readthedocs.io/en/latest/ (see https://github.com/DaviesCentreInformatics/Brahman_Angus_WGBS). All samples had a bisulfite conversion efficiency of >99%. All downstream analyses only used CpGs from autosomes with ≥ 10X coverage.

Identification of shared and breed-specific CpG sites

For a given autosome, we extracted 1000bp around all CpGs that were not in the first 500bp or last 500bp of the chromosome; this yielded sequences that were 1002bp long. We then mapped the 1002bp CpG sequences from one subspecies to the reference of the other. We used minimap2 (v. 2.24) [59] with the ‘map-hifi’ preset to align CpGs from a given chromosome in one breed to the same chromosome in the other breed; alignments were sorted using SAMtools (v 1.11) [100]. Once the long sequences were aligned, we filtered the BAM file and considered all alignments where at least 900 bp were successfully aligned to the reference. We then used the Align package from BioPython (v 1.80) [102] to perform a local alignment between the 102bp sequences taken from the midpoint of the query and reference. We then recorded which CpG sites were shared between Brahman and Angus, which CpG sites differed, and which could not be aligned during the initial minimap2 alignment step (S. table 6). We performed subsequent analyses using all CpGs present on the autosomes for each reference and again using only the shared CpGs that passed the ≥ 10X coverage criteria. The CpG sites that could not be aligned in the initial alignment step with minimap2 (i.e. a genomic region that is present in one breed but missing in the other breed) or constituted a SNP were considered breed-specific CpG sites. All steps described in this section were performed for both Brahman and Angus reference genomes.

Identification of SNPs and SVs between genomes

To determine the accuracy of minimap2 in identifying SNPs between the Brahman and Angus genomes, we first introduced artificial mutations into each genome using SNP Mutator [103]. The Angus and Brahman genomes have previously been reported to differ by ~1% [39]. Therefore, to determine how well minimap2 can detect SNPs between sequences that are divergent by ~1%, we first simulated mutations in each autosome for both species. For example, chromosome 1 in the Angus reference genome is 157,005,132 bp long, so we set the number of substitutions to 1,570,051 SNPs. In addition, the random seed was set to 12, and the number of times each autosome was mutated was set to 1. We repeated this for all autosomes in the Brahman and Angus genomes, adjusting the number of SNPs to maintain the 1% divergence in each autosome. We then mapped the mutated autosomal sequence to the original sequence for each autosome and breed, giving us 58 “replicates”. Minimap2 [59] and PAFtools were used to identify variants between the original and mutated sequences. The minimap2 mapping parameters used were ‘-x asm10, -c, --cs’, followed by PAFtools ‘call —f’, where the file provided to the —f argument was the original, unmutated autosomal sequence. Minimap2 and PAFtools showed a mean accuracy of ~99% across the 58 autosomes, suggesting a good ability to identify SNPs between the two breeds (S. table 13). We then aligned each autosome from Brahman to each autosome from Angus, using minimap2 with the parameters ‘-cx asm10’ and ‘—cs’. The output from minimap2 was then used as input to paftools.js call with the parameter ‘-f <reference_autosome.fa>’, where reference_autosome.fa refers to the autosome that was supplied first to minimap2, i.e. the reference sequence, not the query. We then used the output VCF files to determine the SNP and SVs between the two genomes.

SNP and SV Enrichment

To determine whether CpGs were significantly impacted by SNPs, we identified all SNPs between Brahman and Angus that impacted a CpG site. As Brahman autosomes were used as the reference to minimap2, we used the coordinates of all CpGs within the Brahman genome to identify which SNPs in the VCF file were altering a CpG site. We determined the probability of a SNP occurring as the number of SNPs (n) identified between a given pair of autosomes divided by the length of the autosome (l), $P(\text{SNP\_occurring}) = \frac{n}{l}$. As a CpG site only needs a single SNP to destroy it, one need only consider the probability of a single base changing, I.e., either the ‘C’ or the ‘G’. To test whether CpGs were significantly more likely to be affected by a SNP, we performed a binomial test for each autosome where k was equal to the number of SNPs affecting a CpG site, n was the number of SNPs that occurred, and P was the probability of a SNP occurring.

To assess whether CpGs were significantly enriched within SVs identified between the two genomes, we counted the number of CpG dinucleotides occurring within SV sequences and compared that against the number of CpGs that occurred in non-SV regions. To determine the probability of a CpG occurring outside an SV, we first identified the SV coordinates from the VCF file produced by PAFtools and constructed a bed file of SVs for each reference genome. Next, we used BEDTools [104] to generate coordinates of the complementary regions, i.e., the non-SV regions of each genome. We then extracted the fasta sequence of these regions for each genome. We then counted the number of CpGs that occurred in these regions and divided them by the combined length of each region to determine the probability of a CpG occurring in the non-SV portion of the genome. We then performed a binomial test to determine if CpGs were more likely to occur within an SV than non-SV regions.

Determining quantification bias between genomes

To determine whether there was a significant quantification bias between the Brahman and Angus reference genomes for a given sample, we compared the vector of all CpG sites with at least 10X coverage when mapped to Angus in sample *I* against the vector of all CpG sites with at least 10X coverage when mapped to Brahman. To ensure the vectors were equal, we randomly subset the larger vector to be the same length as the smaller, i.e., if the Brahman reference genome had more CpG sites with 10X coverage for that sample, the Brahman vector was randomly subset to match the number of CpG sites in the Angus vector for that sample. We repeated this for all samples using all CpGs and again with just the CpG sites marked as shared (Table 3; S. table 8). We then pooled all CpG sites for each sample within a group, e.g. all samples from BTBT, and determined whether the CpG methylation differed significantly for that group when mapped to Angus and Brahman. The p-value was determined using a Wilcoxon Rank Sum test and adjusted for multiple testing using the Benjamini-Hochberg procedure. We identified variable CpGs by matching the shared CpGs between reference genomes and then identifying those with an absolute methylation difference greater than 10%.

Identification of differentially methylated regions

The methylKit package (v. 1.22.0) [77] was used to identify DMRs between breed and POE groups. We investigated breed effects by comparing BIBI samples with BTBT samples, maternal effects by comparing samples with BIBI dams (BIBI; BTBI) and those with BTBT dams (BTBT; BIBT) and paternal effects by comparing samples with BIBI sires (BIBI; BIBT) to those with BTBT sires (BTBT; BTBI) (S. table 14). The reference group was always the breed that matched the reference genome. For example, when BIBI and BTBT WGBS reads were aligned to the Brahman reference genome, BIBI samples were treated as the control group and BTBT as the treatment group.

We followed the pipeline described by the methylKit authors for DMR analysis [77]. Briefly, we only considered CpGs that were identified as shared. We then removed all CpG sites with less than 10X coverage and more than the 99.9th percentile of coverage. Reads with too high coverage (e.g. from PCR duplication bias) can impair the accurate determination of the methylation percentage at that site and is a recommended pre-processing step for methylKit [77]. We then normalized the coverage using the default methylKit normalization strategy. We merged the CpG counts per group using the ‘unite’ function with ‘destrand = T’ and ‘min.per.group = 5L’ so that a given CpG site had to be covered by at least ten reads in five out of six samples per group. For the parent of origin DMR analyses, we set ‘min.per.group = 10L’.

We then identified differentially methylated cytosines between groups using the ‘calculateDiffMeth’ function, with sex as a covariate in the model. To determine differentially methylated regions, we used the ‘tileMethylCounts’ function with default parameters to divide the genome into regions for differential methylation analysis. This step allowed methylKit to divide the genome into non-overlapping regions based on the tiling windows. MethylKit then models the methylation at a given cytosine or region by fitting a logistic regression:

$$\log\left(\frac{P_i}{1 - P_i}\right) = \beta_0 + \beta_1 * T_i + \mathrm{a}_{\mathrm{sex}} * \mathrm{Sex}_i$$

P_i denotes the methylation proportion for sample i in samples 1,…,*n*, where *n* is the number of samples across both groups in the comparison [77]. T_i represents the groups (0 for control, 1 for treatment). β_0 denotes the log odds of the control group (fraction of reads reporting C / 1 – the fraction of reads reporting C). β_1 denotes the log odds ratio between the control and treatment. α_{sex} denotes the parameter for the sex covariate and Sex_i denotes the sex (0 = male; 1 = female) for sample *i*. For further details, refer to Akalin, Kormaksson [77]. This design resulted in six different logistic models being fit: model 1A (breed comparison when aligned to the Angus reference), model 1B (breed comparison when aligned to the Brahman reference), model 2A (dam of origin comparison when aligned to the Angus reference) model 2B (dam of origin when aligned to the Brahman reference), model 3A (sire of origin when aligned to the Angus reference) and model 3B (sire of origin when aligned to the Brahman reference) (S. table 14). Any DMRs identified were either hypo- or hypermethylated with respect to the control group. We retained all DMRs with a difference in methylation of ≥ 10% and a qvalue of ≤ 0.01 for further analysis. An overview of the samples, reference genomes, types of CpGs and DMR analysis is given in Figure 1A-G.

DMR coordinate conversion

To determine if a given DMR changed methylation direction between genomes, we had to convert the coordinates of DMRs identified by alignment with the Angus genome to Brahman coordinates and vice versa. We considered a DMR as changing methylation direction if, for example, it is hypomethylated in BIBI samples compared to BTBT samples when using the Brahman reference but becomes hypermethylated in BIBI using the Angus reference genome. To investigate this, we first converted the DMR bed files to GTF files and then used Liftoff (v.1.6.2) [105] to transfer coordinates from one reference genome to the other. We then identified DMRs reciprocally overlapping one another by at least 90% between the two genomes, with these DMRs being considered successfully lifted over. DMRs that did not overlap by 90% were not considered for the methylation direction change analysis.

RNA-seq mapping and pre-processing

RNA-seq reads were mapped to the Brahman and Angus genomes as in the WGBS mapping step. Briefly, reads were checked for quality using FastQC (v. 0.11.4) (https://www.bioinformatics.babraham.ac.uk/projects/fastqc/) before being trimmed with Trim Galore (v. 0.4.2) (https://github.com/FelixKrueger/TrimGalore) with the parameters '--quality 10' and '--length 100'. Reads were mapped using HiSAT2 (v. 2.1.0) to both the Brahman and Angus reference genomes [39]; alignment files were sorted using SAMtools (v. 1.10) [100]. FeatureCount from the Rsubread package (v. 2.10.5) [106] was used to count how many reads mapped to genes.

Differential gene expression

Differential gene expression analysis was performed using an in-house R script with the DESeq2 (v. 1.40.2) [107] R package. The genome annotation was based on Ensembl v.104 for Brahman and Angus. The orientation of the genes was reversed where necessary to correspond with the orientation of the chromosomes of ARS-UCD1.2. In the breed comparison, where the number of samples in each group was six, we retained genes that had a count ≥ 10 in at least three samples. In the POE comparisons, we retained genes that had a count ≥ 10 in at least six samples. The model design used to fit the DESeq2 model was '~0 + Genetics + Sex + Batch'. DESeq2 then estimated size factors, dispersion and finally fits a negative binomial generalised linear model to identify DEGs. We then compared differential gene expression between purebred Angus and Brahman, Angus dams and Brahman dams, and Angus sires and Brahman sires. Genes with significant differences in gene expression at an adjusted p-value ≤ 0.05 were retained for further analysis. Furthermore, we performed canonical correlation analysis (CCA) to estimate the coefficient of determination between gene expression and DNA methylation, R^2 = 0.18, suggesting a moderate relationship between gene expression and DNA methylation.

Identifying imprinted genes

We downloaded a list of genes with evidence of imprinting in human, mouse and cattle from Morison, Ramsay and Spencer [108] and https://www.geneimprint.org. We then used OrthoFinder to identify human orthologs of both Brahman and Angus genes [109], allowing us to assign Human Genome Organisation Gene Nomenclature Committee (HGNC) symbols to genes in each breed. To do this, we first identified which Brahman proteins had orthologs in human. We then identified the genes that encoded these proteins and used this information to assign human and Brahman genes as orthologs. We repeated the process for the Angus genes. We then identified all genes that could be assigned an HGNC symbol from the Brahman Ensembl annotation version 104 that were also present in the imprinted gene list (S. table 15). This filtering gave us 80 imprinted genes for Brahman autosomes. We repeated the process for Angus using the Angus Ensembl annotation version 104 and identified 79 imprinted genes. The discrepancy is due to one imprinted gene for Angus occurring on an unplaced scaffold.

Linking DMRs to DEGs

For each DEG, we considered five different regions in and around the gene where DMRs might have an influence. These regions included putative enhancer regions, 5kb outside the gene body, and the gene body itself (S. figure 3). The upstream putative enhancer region started 130kb upstream of the gene and then stopped 5kb upstream of the gene body for a total length of 125kb. We repeated this for the downstream putative enhancer region, starting 5kb downstream of the gene body and extending out 125kb. This number was based on the median distance between enhancers and their gene targets [110]. The 5kb region was from upstream of the start of the gene body to the start of the gene body. Again, this was repeated for the downstream 5kb region. The gene body was the region annotated as "gene" in the Ensembl annotation file. We then found all DMRs that overlapped these regions by at least 90% of their length using 'bedtools intersect' with the '-f' and '-F' arguments, both set at 0.9 and the '-e' argument set to True.

Declarations

Ethics approval

All animal experiments and procedures described in this study complied with Australian guidelines, approved by the University of Adelaide Animal Ethics Committee and followed the ARRIVE Guidelines (https://arriveguidelines.org/) (Approval No. S-094-2005).

Consent for publication

Not applicable

Availability of data and materials

The datasets generated and analysed during the current study are available in the NCBI SRA repository under BioProject: PRJNA626458. Code used to analyse the data is available at: https://github.com/DaviesCentreInformatics/Brahman_Angus_WGBS.

Competing interests

The authors declare that they have no competing interests.

Funding

The study was funded by the JS Davies bequest through the Davies Livestock Research Centre.

Authors' contributions

WYL, JLW and SH conceived and managed the project. SH designed and obtained *Bos taurus* and *Bos indicus* fetal resources. TC extracted WGBS samples and performed QC. CM performed all analyses; CM, SH, WYL and HAR interpreted data. CM and WYL drafted the manuscript, and all authors read, edited and approved the final manuscript.

Acknowledgements

We thank Yan Ren for uploading and performing the initial QC of WGBS data on the University of Adelaide's Phoenix HPC.

Supplementary information

Additional file 1. Supplementary figures referred to in the main text.

Additional file 2. WGBS mapping statistics.

Additional file 3. Table describing the number of CpGs in each reference and what percentage of those have 10X coverage.

Additional file 4. Mean global CpG coverage.

Additional file 5. Global CpG methylation.

Additional file 6. Methylation of CpGs in different genomic regions.

Additional file 7. Alignment statistics of cross-reference genome CpG mapping

Additional file 8. SNP and SV enrichment at CpG sites.

Additional file 9. Table containing the variable CpG sites between the genomes.

Additional file 10. Table of differentially methylated regions that exhibited direction changes.

Additional file 11. Number of DMRs in each comparison.

Additional file 12. Number of up and down-regulated genes in each comparison.

Additional file 13. Minimap2 accuracy in detecting SNPs introduced to the reference genome.

Additional file 14. Sample information.

Additional file 15. List of imprinted genes.

References

1. Jansz N. DNA methylation dynamics at transposable elements in mammals. Essays in Biochemistry. 2019;63 6:677-89. doi:10.1042/ebc20190039.

2. Li E and Zhang Y. DNA methylation in mammals. Cold Spring Harb Perspect Biol. 2014;6 5:a019133. doi:10.1101/cshperspect.a019133.

3. Ramsahoye BH, Biniszkiewicz D, Lyko F, Clark V, Bird AP and Jaenisch R. Non-CpG methylation is prevalent in embryonic stem cells and may be mediated by DNA methyltransferase 3a. Proceedings of the National Academy of Sciences. 2000;97 10:5237-42. doi:doi:10.1073/pnas.97.10.5237.

4. Ziller MJ, Müller F, Liao J, Zhang Y, Gu H, Bock C, et al. Genomic Distribution and Inter-Sample Variation of Non-CpG Methylation across Human Cell Types. PLOS Genetics. 2011;7 12:e1002389. doi:10.1371/journal.pgen.1002389.

5. Moore LD, Le T and Fan G. DNA Methylation and Its Basic Function. Neuropsychopharmacology. 2013;38 1:23-38. doi:10.1038/npp.2012.112.

6. Kass SU, Landsberger N and Wolffe AP. DNA methylation directs a time-dependent repression of transcription initiation. Curr Biol. 1997;7 3:157-65. doi:10.1016/s0960-9822(97)70086-1.

7. Smith J, Sen S, Weeks RJ, Eccles MR and Chatterjee A. Promoter DNA Hypermethylation and Paradoxical Gene Activation. Trends in Cancer. 2020;6 5:392-406. doi:https://doi.org/10.1016/j.trecan.2020.02.007.

8. Cho J-W, Shim HS, Lee CY, Park SY, Hong MH, Lee I and Kim HR. The importance of enhancer methylation for epigenetic regulation of tumorigenesis in squamous lung cancer. Experimental & Molecular Medicine. 2022;54 1:12-22. doi:10.1038/s12276-021-00718-4.

9. Spainhour JCG, Lim HS, Yi SV and Qiu P. Correlation Patterns Between DNA Methylation and Gene Expression in The Cancer Genome Atlas. Cancer Informatics. 2019;18 doi:10.1177/1176935119828776.

10. Charlet J, Duymich Christopher E, Lay Fides D, Mundbjerg K, Dalsgaard Sørensen K, Liang G and Jones Peter A. Bivalent Regions of Cytosine Methylation and H3K27 Acetylation Suggest an Active Role for DNA Methylation at Enhancers. Molecular Cell. 2016;62 3:422-31. doi:10.1016/j.molcel.2016.03.033.

11. Creyghton MP, Cheng AW, Welstead GG, Kooistra T, Carey BW, Steine EJ, et al. Histone H3K27ac separates active from poised enhancers and predicts developmental state. Proceedings of the National Academy of Sciences. 2010;107 50:21931-6. doi:10.1073/pnas.1016071107.

12. Kang Y, Kim YW, Kang J and Kim A. Histone H3K4me1 and H3K27ac play roles in nucleosome eviction and eRNA transcription, respectively, at enhancers. The FASEB Journal. 2021;35 8 doi:10.1096/fj.202100488r.

13. Wang M, Hancock TP, MacLeod IM, Pryce JE, Cocks BG and Hayes BJ. Putative enhancer sites in the bovine genome are enriched with variants affecting complex traits. Genet Sel Evol. 2017;49 1:56. doi:10.1186/s12711-017-0331-4.

14. Zhu Y, Zhou Z, Huang T, Zhang Z, Li W, Ling Z, et al. Mapping and analysis of a spatiotemporal H3K27ac and gene expression spectrum in pigs. Sci China Life Sci. 2022;65 8:1517-34. doi:10.1007/s11427-021-2034-5.

15. Wulfridge P, Langmead B, Feinberg AP and Hansen KD. Analyzing whole genome bisulfite sequencing data from highly divergent genotypes. Nucleic Acids Research. 2019;47 19:e117-e. doi:10.1093/nar/gkz674.

16. Brandt DYC, Aguiar VRC, Bitarello BD, Nunes K, Goudet J and Meyer D. Mapping Bias Overestimates Reference Allele Frequencies at the HLA Genes in the 1000 Genomes Project Phase I Data. G3 Genes|Genomes|Genetics. 2015;5 5:931-41. doi:10.1534/g3.114.015784.

17. Degner JF, Marioni JC, Pai AA, Pickrell JK, Nkadori E, Gilad Y and Pritchard JK. Effect of read-mapping biases on detecting allele-specific expression from RNA-sequencing data. Bioinformatics. 2009;25 24:3207-12. doi:10.1093/bioinformatics/btp579.

18. Salavati M, Bush SJ, Palma-Vera S, McCulloch MEB, Hume DA and Clark EL. Elimination of Reference Mapping Bias Reveals Robust Immune Related Allele-Specific Expression in Crossbred Sheep. Frontiers in Genetics. 2019;10 doi:10.3389/fgene.2019.00863.

19. Chen N-C, Solomon B, Mun T, Iyer S and Langmead B. Reference flow: reducing reference bias using multiple population genomes. Genome Biology. 2021;22 1:8. doi:10.1186/s13059-020-02229-3.

20. Groza C, Kwan T, Soranzo N, Pastinen T and Bourque G. Personalized and graph genomes reveal missing signal in epigenomic data. Genome Biology. 2020;21 1:124. doi:10.1186/s13059-020-02038-8.

21. Crysnanto D and Pausch H. Bovine breed-specific augmented reference graphs facilitate accurate sequence read mapping and unbiased variant discovery. Genome Biology. 2020;21 1:184. doi:10.1186/s13059-020-02105-0.

22. Lloret-Villas A, Bhati M, Kadri NK, Fries R and Pausch H. Investigating the impact of reference assembly choice on genomic analyses in a cattle breed. BMC Genomics. 2021;22 1 doi:10.1186/s12864-021-07554-w.

23. Günther T and Nettelblad C. The presence and impact of reference bias on population genomic studies of prehistoric human populations. PLOS Genetics. 2019;15 7:e1008302. doi:10.1371/journal.pgen.1008302.

24. Wang T, Antonacci-Fulton L, Howe K, Lawson HA, Lucas JK, Phillippy AM, et al. The Human Pangenome Project: a global resource to map genomic diversity. Nature. 2022;604 7906:437-46. doi:10.1038/s41586-022-04601-8.

25. Liao W-W, Asri M, Ebler J, Doerr D, Haukness M, Hickey G, et al. A draft human pangenome reference. Nature. 2023;617 7960:312-24. doi:10.1038/s41586-023-05896-x.

26. Smith TPL, Bickhart DM, Boichard D, Chamberlain AJ, Djikeng A, Jiang Y, et al. The Bovine Pangenome Consortium: democratizing production and accessibility of genome assemblies for global cattle breeds and other bovine species. Genome Biology. 2023;24 1:139. doi:10.1186/s13059-023-02975-0.

27. Woodhouse MR, Cannon EK, Portwood JL, Harper LC, Gardiner JM, Schaeffer ML and Andorf CM. A pan-genomic approach to genome databases using maize as a model system. BMC Plant Biology. 2021;21 1:385. doi:10.1186/s12870-021-03173-5.

28. McTavish EJ, Decker JE, Schnabel RD, Taylor JF and Hillis DM. New World cattle show ancestry from multiple independent domestication events. Proceedings of the National Academy of Sciences. 2013;110 15:E1398-E406. doi:doi:10.1073/pnas.1303367110.

29. Bruford MW, Bradley DG and Luikart G. DNA markers reveal the complexity of livestock domestication. Nature Reviews Genetics. 2003;4 11:900-10. doi:10.1038/nrg1203.

30. Ajmone-Marsan P, Garcia JF and Lenstra JA. On the origin of cattle: How aurochs became cattle and colonized the world. Evolutionary Anthropology: Issues, News, and Reviews. 2010;19 4:148-57. doi:https://doi.org/10.1002/evan.20267.

31. MacHugh DE, Larson G and Orlando L. Taming the Past: Ancient DNA and the Study of Animal Domestication. Annual Review of Animal Biosciences. 2017;5 1:329-51. doi:10.1146/annurev-animal-022516-022747.

32. Loftus RT, MacHugh DE, Bradley DG, Sharp PM and Cunningham P. Evidence for two independent domestications of cattle. Proceedings of the National Academy of Sciences. 1994;91 7:2757-61. doi:doi:10.1073/pnas.91.7.2757.

33. Elzo MA, Johnson DD, Wasdin JG and Driver JD. Carcass and meat palatability breed differences and heterosis effects in an Angus–Brahman multibreed population. Meat Science. 2012;90 1:87-92. doi:https://doi.org/10.1016/j.meatsci.2011.06.010.

34. Dikmen S, Mateescu RG, Elzo MA and Hansen PJ. Determination of the optimum contribution of Brahman genetics in an Angus-Brahman multibreed herd for regulation of body temperature during hot weather. Journal of Animal Science. 2018;96 6:2175-83. doi:10.1093/jas/sky133.

35. Goszczynski DE, Corbi-Botto CM, Durand HM, Rogberg-Muñoz A, Munilla S, Peral-Garcia P, et al. Evidence of positive selection towards Zebuine haplotypes in the BoLA region of Brangus cattle. Animal. 2018;12 2:215-23. doi:https://doi.org/10.1017/S1751731117001380.

36. Koren S, Rhie A, Walenz BP, Dilthey AT, Bickhart DM, Kingan SB, et al. De novo assembly of haplotype-resolved genomes with trio binning. Nat Biotechnol. 2018;36 12:1174-82. doi:10.1038/nbt.4277.

37. Decker JE, Mckay SD, Rolf MM, Kim J, Molina Alcalá A, Sonstegard TS, et al. Worldwide Patterns of Ancestry, Divergence, and Admixture in Domesticated Cattle. PLoS Genetics. 2014;10 3:e1004254. doi:10.1371/journal.pgen.1004254.

38. Hiendleder S, Lewalski H and Janke A. Complete mitochondrial genomes of Bos taurus and Bos indicus provide new insights into intra-species variation, taxonomy and domestication. Cytogenetic and Genome Research. 2008;120 1-2:150-6. doi:10.1159/000118756.

39. Low WY, Tearle R, Liu R, Koren S, Rhie A, Bickhart DM, et al. Haplotype-resolved genomes provide insights into structural variation and gene content in Angus and Brahman cattle. Nature Communications. 2020;11 1:1-14.

40. Yang J, Horton JR, Akdemir KC, Li J, Huang Y, Kumar J, et al. Preferential CEBP binding to T:G mismatches and increased C-to-T human somatic mutations. Nucleic Acids Research. 2021;49 9:5084-94. doi:10.1093/nar/gkab276.

41. Żemojtel T, Kiełbasa SM, Arndt PF, Behrens S, Bourque G and Vingron M. CpG Deamination Creates Transcription Factor–Binding Sites with High Efficiency. Genome Biology and Evolution. 2011;3 0:1304-11. doi:10.1093/gbe/evr107.

42. Van Laere A-S, Nguyen M, Braunschweig M, Nezer C, Collette C, Moreau L, et al. A regulatory mutation in IGF2 causes a major QTL effect on muscle growth in the pig. Nature. 2003;425 6960:832-6. doi:10.1038/nature02064.

43. Zhang YQ, Yang LX, Kucherlapati M, Hadjipanayis A, Pantazi A, Bristow CA, et al. Global impact of somatic structural variation on the DNA methylome of human cancers. Genome Biology. 2019;20 1 doi:10.1186/s13059-019-1818-9.

44. Lutz PE, Almeid D, Belzeaux R, Yalcin I and Turecki G. Epigenetic regulation of the kappa opioid receptor gene by an insertion-deletion in the promoter region. European Neuropsychopharmacology. 2018;28 2:334-40. doi:10.1016/j.euroneuro.2017.12.013.

45. Lawson HA, Cheverud JM and Wolf JB. Genomic imprinting and parent-of-origin effects on complex traits. Nat Rev Genet. 2013;14 9:609-17. doi:10.1038/nrg3543.

46. Shi W, Krella A, Orth A, Yu Y and Fundele R. Widespread disruption of genomic imprinting in adult interspecies mouse (Mus) hybrids. Genesis. 2005;43 3:100-8. doi:10.1002/gene.20161.

47. Vaughn RN, Kochan KJ, Torres AK, Du M, Riley DG, Gill CA, et al. Skeletal Muscle Expression of Actinin-3 (ACTN3) in Relation to Feed Efficiency Phenotype of F-2 Bos indicus-Bos taurus Steers. Frontiers in Genetics. 2022;13 doi:10.3389/fgene.2022.796038.

48. Pan ZX, Zhang JL, Zhang JB, Zhou B, Chen J, Jiang ZH and Liu HL. Expression Profiles of the Insulin-like Growth Factor System Components in Liver Tissue during Embryonic and Postnatal Growth of Erhualian and Yorkshire Reciprocal Cross F-1 Pigs. Asian-Australasian Journal of Animal Sciences. 2012;25 7:903-12. doi:10.5713/ajas.2011.11385.

49. Moore GE, Ishida M, Demetriou C, Al-Olabi L, Leon LJ, Thomas AC, et al. The role and interaction of imprinted genes in human fetal growth. Philos Trans R Soc Lond B Biol Sci. 2015;370 1663:20140074. doi:10.1098/rstb.2014.0074.

50. Eggermann T, Davies JH, Tauber M, van den Akker E, Hokken-Koelega A, Johansson G and Netchine I. Growth Restriction and Genomic Imprinting-Overlapping Phenotypes Support the Concept of an Imprinting Network. Genes. 2021;12 4 doi:10.3390/genes12040585.

51. Yuen RKC, Jiang R, Penaherrera MS, McFadden DE and Robinson WP. Genome-wide mapping of imprinted differentially methylated regions by DNA methylation profiling of human placentas from triploidies. Epigenetics & Chromatin. 2011;4 doi:10.1186/1756-8935-4-10.

52. Doria S, Sousa M, Fernandes S, Ramalho C, Brandao O, Matias A, et al. Gene expression pattern of IGF2, PHLDA2, PEG10 and CDKN1C imprinted genes in spontaneous miscarriages or fetal deaths. Epigenetics. 2010;5 5:444-50. doi:10.4161/epi.5.5.12118.

53. Piedrahita JA. The Role of Imprinted Genes in Fetal Growth Abnormalities. Birth Defects Research Part a-Clinical and Molecular Teratology. 2011;91 8:682-92. doi:10.1002/bdra.20795.

54. Amat S, Dahlen CR, Swanson KC, Ward AK, Reynolds LP and Caton JS. Bovine Animal Model for Studying the Maternal Microbiome, in utero Microbial Colonization and Their Role in Offspring Development and Fetal Programming. Front Microbiol. 2022;13:854453. doi:10.3389/fmicb.2022.854453.

55. Peruffo A and Cozzi B. Bovine Brain: An in vitro Translational Model in Developmental Neuroscience and Neurodegenerative Research. Front Pediatr. 2014;2:74. doi:10.3389/fped.2014.00074.

56. Chen Z, Robbins KM, Wells KD and Rivera RM. Large offspring syndrome: a bovine model for the human loss-of-imprinting overgrowth syndrome Beckwith-Wiedemann. Epigenetics. 2013;8 6:591-601.

57. Malhi PS, Adams GP and Singh J. Bovine Model for the Study of Reproductive Aging in Women: Follicular, Luteal, and Endocrine Characteristics1. Biology of Reproduction. 2005;73 1:45-53. doi:10.1095/biolreprod.104.038745.

58. MacPhillamy C, Alinejad-Rokny H, Pitchford WS and Low WY. Cross-species enhancer prediction using machine learning. Genomics. 2022;114 5:110454. doi:10.1016/j.ygeno.2022.110454.

59. Li H. Minimap2: pairwise alignment for nucleotide sequences. Bioinformatics. 2018;34 18:3094-100.

60. Zhou D, Li Z, Yu D, Wan L, Zhu Y, Lai M and Zhang D. Polymorphisms involving gain or loss of CpG sites are significantly enriched in trait-associated SNPs. Oncotarget. 2015;6 37.

61. Wang Z, Wu X and Wang Y. A framework for analyzing DNA methylation data from Illumina Infinium HumanMethylation450 BeadChip. BMC Bioinformatics. 2018;19 Suppl 5:115. doi:10.1186/s12859-018-2096-3.

62. Leenen FAD, Muller CP and Turner JD. DNA methylation: conducting the orchestra from exposure to phenotype? Clinical Epigenetics. 2016;8 1:92. doi:10.1186/s13148-016-0256-8.

63. Thomson K, Game J, Karouta C, Morgan IG and Ashby R. Correlation between small-scale methylation changes and gene expression during the development of myopia. The FASEB Journal. 2022;36 1:e22129. doi:https://doi.org/10.1096/fj.202101487R.

64. He Y, Hariharan M, Gorkin DU, Dickel DE, Luo C, Castanon RG, et al. Spatiotemporal DNA methylome dynamics of the developing mouse fetus. Nature. 2020;583 7818:752-9. doi:10.1038/s41586-020-2119-x.

65. Hama N, Totoki Y, Miura F, Tatsuno K, Saito-Adachi M, Nakamura H, et al. Epigenetic landscape influences the liver cancer genome architecture. Nature Communications. 2018;9 doi:10.1038/s41467-018-03999-y.

66. Zhou Y, Liu S, Hu Y, Fang L, Gao Y, Xia H, et al. Comparative whole genome DNA methylation profiling across cattle tissues reveals global and tissue-specific methylation patterns. BMC Biology. 2020;18 1:85. doi:10.1186/s12915-020-00793-5.

67. Valiente-Mullor C, Beamud B, Ansari I, Francés-Cuesta C, García-González N, Mejía L, et al. One is not enough: On the effects of reference genome for the mapping and subsequent analyses of short-reads. PLOS Computational Biology. 2021;17 1:e1008678. doi:10.1371/journal.pcbi.1008678.

68. Capra E, Lazzari B, Milanesi M, Nogueira GP, Garcia Jf, Utsunomiya YT, et al. Comparison between indicine and taurine cattle DNA methylation reveals epigenetic variation associated to differences in morphological adaptive traits. Epigenetics. 2023;18 1:2163363. doi:10.1080/15592294.2022.2163363.

69. Gonzalez E, Kulkarni H, Bolivar H, Mangano A, Sanchez R, Catano G, et al. The influence of CCL3L1 gene-containing segmental duplications on HIV-1/AIDS susceptibility. Science. 2005;307 5714:1434-40. doi:10.1126/science.1101160.

70. Marshall CR, Noor A, Vincent JB, Lionel AC, Feuk L, Skaug J, et al. Structural variation of chromosomes in autism spectrum disorder. Am J Hum Genet. 2008;82 2:477-88. doi:10.1016/j.ajhg.2007.12.009.

71. Kumar RA, KaraMohamed S, Sudi J, Conrad DF, Brune C, Badner JA, et al. Recurrent 16p11.2 microdeletions in autism. Hum Mol Genet. 2008;17 4:628-38. doi:10.1093/hmg/ddm376.

72. Weiss LA, Shen Y, Korn JM, Arking DE, Miller DT, Fossdal R, et al. Association between microdeletion and microduplication at 16p11.2 and autism. N Engl J Med. 2008;358 7:667-75. doi:10.1056/NEJMoa075974.

73. Bell DA, Taylor JA, Paulson DF, Robertson CN, Mohler JL and Lucier GW. Genetic risk and carcinogen exposure: a common inherited defect of the carcinogen-metabolism gene glutathione S-transferase M1 (GSTM1) that increases susceptibility to bladder cancer. J Natl Cancer Inst. 1993;85 14:1159-64. doi:10.1093/jnci/85.14.1159.

74. Lamb HJ, Ross EM, Nguyen LT, Lyons RE, Moore SS and Hayes BJ. Characterization of the poll allele in Brahman cattle using long-read Oxford Nanopore sequencing. Journal of Animal Science. 2020;98 5 doi:10.1093/jas/skaa127.

75. Rothammer S, Capitan A, Mullaart E, Seichter D, Russ I and Medugorac I. The 80-kb DNA duplication on BTA1 is the only remaining candidate mutation for the polled phenotype of Friesian origin. Genet Sel Evol. 2014;46 1:44. doi:10.1186/1297-9686-46-44.

76. Jacinto JGP, Häfliger IM, Letko A, Drögemüller C and Agerholm JS. A large deletion in the COL2A1 gene expands the spectrum of pathogenic variants causing bulldog calf syndrome in cattle. Acta Vet Scand. 2020;62 1:49. doi:10.1186/s13028-020-00548-w.

77. Akalin A, Kormaksson M, Li S, Garrett-Bakelman FE, Figueroa ME, Melnick A and Mason CE. methylKit: a comprehensive R package for the analysis of genome-wide DNA methylation profiles. Genome Biology. 2012;13 10:R87. doi:10.1186/gb-2012-13-10-r87.

78. Kishore K, de Pretis S, Lister R, Morelli MJ, Bianchi V, Amati B, et al. methylPipe and compEpiTools: a suite of R packages for the integrative analysis of epigenomics data. BMC Bioinformatics. 2015;16 1:313. doi:10.1186/s12859-015-0742-6.

79. Park Y, Figueroa ME, Rozek LS and Sartor MA. MethylSig: a whole genome DNA methylation analysis pipeline. Bioinformatics. 2014;30 17:2414-22. doi:10.1093/bioinformatics/btu339.

80. Lee HJ, Lowdon RF, Maricque B, Zhang B, Stevens M, Li D, et al. Developmental enhancers revealed by extensive DNA methylome maps of zebrafish early embryos. Nature Communications. 2015;6 1:6315. doi:10.1038/ncomms7315.

81. Alajem A, Roth H, Ratgauzer S, Bavli D, Motzik A, Lahav S, et al. DNA methylation patterns expose variations in enhancer-chromatin modifications during embryonic stem cell differentiation. PLOS Genetics. 2021;17 4:e1009498. doi:10.1371/journal.pgen.1009498.

82. Slieker RC, Roost MS, van Iperen L, Suchiman HE, Tobi EW, Carlotti F, et al. DNA Methylation Landscapes of Human Fetal Development. PLoS Genet. 2015;11 10:e1005583. doi:10.1371/journal.pgen.1005583.

83. Slabaugh E, Desai JS, Sartor RC, Lawas LMF, Jagadish SVK and Doherty CJ. Analysis of differential gene expression and alternative splicing is significantly influenced by choice of reference genome. Rna. 2019;25 6:669-84. doi:10.1261/rna.070227.118.

84. Price A and Gibas C. The quantitative impact of read mapping to non-native reference genomes in comparative RNA-Seq studies. PLOS ONE. 2017;12 7:e0180904. doi:10.1371/journal.pone.0180904.

85. Kaminow B, Ballouz S, Gillis J and Dobin A. Pan-human consensus genome significantly improves the accuracy of RNA-seq analyses. Genome Research. 2022;32 4:738-49. doi:10.1101/gr.275613.121.

86. Wu P-Y, Phan JH and Wang MD. Assessing the impact of human genome annotation choice on RNA-seq expression estimates. BMC Bioinformatics. 2013;14 11:S8. doi:10.1186/1471-2105-14-S11-S8.

87. Khan MZ, Ma Y, Ma J, Xiao J, Liu Y, Liu S, et al. Association of DGAT1 With Cattle, Buffalo, Goat, and Sheep Milk and Meat Production Traits. Frontiers in Veterinary Science. 2021;8 doi:10.3389/fvets.2021.712470.

88. Abeel T, Van de Peer Y and Saeys Y. Toward a gold standard for promoter prediction evaluation. Bioinformatics. 2009;25 12:I313-I20. doi:10.1093/bioinformatics/btp191.

89. Tsuda N, Kumadaki S, Higashi C, Ozawa M, Shinozaki M, Kato Y, et al. Intestine-Targeted DGAT1 Inhibition Improves Obesity and Insulin Resistance without Skin Aberrations in Mice. PLOS ONE. 2014;9 11:e112027. doi:10.1371/journal.pone.0112027.

90. Zhang XD, Yan JW, Yan GR, Sun XY, Ji J, Li YM, et al. Pharmacological inhibition of diacylglycerol acyltransferase 1 reduces body weight gain, hyperlipidemia, and hepatic steatosis in db/db mice. Acta Pharmacol Sin. 2010;31 11:1470-7. doi:10.1038/aps.2010.104.

91. Elzo MA, Riley DG, Hansen GR, Johnson DD, Myer RO, Coleman SW, et al. Effect of breed composition on phenotypic residual feed intake and growth in Angus, Brahman, and Angus x Brahman crossbred cattle. J Anim Sci. 2009;87 12:3877-86. doi:10.2527/jas.2008-1553.

92. Giannoukakis N, Deal C, Paquette J, Goodyer CG and Polychronakos C. Parental genomic imprinting of the human IGF2 gene. Nature genetics. 1993;4 1:98-101.

93. Szabo PE, Tang SHE, Rentsendorj A, Pfeifer GP and Mann JR. Maternal-specific footprints at putative CTCF sites in the H19 imprinting control region give evidence for insulator function. Current Biology. 2000;10 10:607-10. doi:10.1016/s0960-9822(00)00489-9.

94. Yang YW, Hu JF, Ulaner GA, Li T, Yao XM, Vu TH and Hoffman AR. Epigenetic regulation of Igf2/H19 imprinting at CTCF insulator binding sites. Journal of Cellular Biochemistry. 2003;90 5:1038-55. doi:10.1002/jcb.10684.

95. Ren Y, Tseng E, Smith TPL, Hiendleder S, Williams JL and Low WY. Long read isoform sequencing reveals hidden transcriptional complexity between cattle subspecies. BMC Genomics. 2023;24 1:108. doi:10.1186/s12864-023-09212-9.

96. Paten B, Novak AM, Eizenga JM and Garrison E. Genome graphs and the evolution of genome inference. Genome Res. 2017;27 5:665-76. doi:10.1101/gr.214155.116.

97. Liu R, Tearle R, Low WY, Chen T, Thomsen D, Smith TPL, et al. Distinctive gene expression patterns and imprinting signatures revealed in reciprocal crosses between cattle sub-species. BMC Genomics. 2021;22 1 doi:10.1186/s12864-021-07667-2.

98. Di Tommaso P, Chatzou M, Floden EW, Barja PP, Palumbo E and Notredame C. Nextflow enables reproducible computational workflows. Nature Biotechnology. 2017;35 4:316-9. doi:10.1038/nbt.3820.

99. Rosen BD, Bickhart DM, Schnabel RD, Koren S, Elsik CG, Tseng E, et al. De novo assembly of the cattle reference genome with single-molecule sequencing. Gigascience. 2020;9 3:giaa021-giaa. doi:10.1093/gigascience/giaa021.

100. Li H, Handsaker B, Wysoker A, Fennell T, Ruan J, Homer N, et al. The Sequence Alignment/Map format and SAMtools. Bioinformatics. 2009;25 16:2078-9. doi:10.1093/bioinformatics/btp352.

101. Okonechnikov K, Conesa A and García-Alcalde F. Qualimap 2: advanced multi-sample quality control for high-throughput sequencing data. Bioinformatics. 2016;32 2:292-4. doi:10.1093/bioinformatics/btv566.

102. Cock PJA, Antao T, Chang JT, Chapman BA, Cox CJ, Dalke A, et al. Biopython: freely available Python tools for computational molecular biology and bioinformatics. Bioinformatics. 2009;25 11:1422-3. doi:10.1093/bioinformatics/btp163.

103. Davis S, Pettengill JB, Luo Y, Payne J, Shpuntoff A, Rand H and Strain E. CFSAN SNP Pipeline: an automated method for constructing SNP matrices from next-generation sequence data. PeerJ Computer Science. 2015;1:e20.

104. Quinlan AR and Hall IM. BEDTools: a flexible suite of utilities for comparing genomic features. Bioinformatics. 2010;26 6:841-2. doi:10.1093/bioinformatics/btq033.

105. Shumate A and Salzberg SL. Liftoff: accurate mapping of gene annotations. Bioinformatics. 2021;37 12:1639-43. doi:10.1093/bioinformatics/btaa1016.

106. Liao Y, Smyth GK and Shi W. The R package Rsubread is easier, faster, cheaper and better for alignment and quantification of RNA sequencing reads. Nucleic Acids Research. 2019;47 8:e47-e. doi:10.1093/nar/gkz114.

107. Love MI, Huber W and Anders S. Moderated estimation of fold change and dispersion for RNA-seq data with DESeq2. Genome Biology. 2014;15 12:550. doi:10.1186/s13059-014-0550-8.

108. Morison IM, Ramsay JP and Spencer HG. A census of mammalian imprinting. Trends in Genetics. 2005;21 8:457-65. doi:10.1016/j.tig.2005.06.008.

109. Emms DM and Kelly S. OrthoFinder: phylogenetic orthology inference for comparative genomics. Genome Biology. 2019;20 1:238. doi:10.1186/s13059-019-1832-y.

110. Jin F, Li Y, Dixon JR, Selvaraj S, Ye Z, Lee AY, et al. A high-resolution map of the three-dimensional chromatin interactome in human cells. Nature. 2013;503 7475:290-4. doi:10.1038/nature12644.

Tables

Table 3. Sample-wise and group-wise methylation quantification biases.

Sample-wise				Group-wise			
**ID**	**Difference[A]**	**p-value**	**Adj. p-value**	**ID**	**Difference[A]**	**p-value**	**Adj. p-value**
All CpGs[B]				All CpGs[C]			
F103	1.99	5.00E-324	5.00E-324	BTBT	1.98	5.00E-324	5.00E-324
F105	2.16	5.00E-324	5.00E-324				
F52	2.25	5.00E-324	5.00E-324				
F53	1.88	5.00E-324	5.00E-324				
F60	1.73	5.00E-324	5.00E-324				
F7	1.87	5.00E-324	5.00E-324				
F100	1.15	5.00E-324	5.00E-324	BTBI	0.79	5.00E-324	5.00E-324
F104	0.83	5.00E-324	5.00E-324				
F106	0.85	5.00E-324	5.00E-324				
F61	1.06	5.00E-324	5.00E-324				
F74	0.86	5.00E-324	5.00E-324				
F97	0.01	3.69E-02	3.69E-02				
F13	0.66	5.00E-324	5.00E-324	BIBT	0.69	5.00E-324	5.00E-324
F62	0.63	5.00E-324	5.00E-324				
F77	0.73	5.00E-324	5.00E-324				
F80	0.71	5.00E-324	5.00E-324				
F8	0.75	5.00E-324	5.00E-324				
F91	0.65	5.00E-324	5.00E-324				
F22	0.34	5.00E-324	5.00E-324	BIBI	0.34	5.00E-324	5.00E-324
F46	0.34	8.02E-301	8.37E-301				
F56	0.29	5.81E-301	6.34E-301				
F65	0.40	5.00E-324	5.00E-324				

F78	0.36	5.00E-324	5.00E-324				
F99	0.34	5.00E-324	5.00E-324				
Shared[D]				*Shared*[E]			
F103	0.06	1.24E-09	7.41E-09	BTBT	0.05	3.54E-54	1.42E-53
F105	0.06	3.78E-13	9.06E-12				
F52	0.06	2.12E-12	2.55E-11				
F53	0.05	1.36E-08	6.55E-08				
F60	0.04	4.05E-08	1.62E-07				
F7	0.05	6.76E-10	5.41E-09				
F100	0.04	5.22E-06	1.79E-05	BTBI	0.04	3.12E-22	6.24E-22
F104	0.03	2.15E-04	4.29E-04				
F106	0.04	3.88E-04	7.16E-04				
F61	0.04	6.40E-06	1.92E-05				
F74	0.04	2.93E-05	7.82E-05				
F97	0.03	3.89E-02	5.19E-02				
F13	0.03	1.83E-03	2.92E-03	BIBT	0.03	2.47E-17	3.30E-17
F62	0.03	6.50E-03	9.18E-03				
F77	0.03	1.07E-03	1.83E-03				
F80	0.04	3.82E-05	9.17E-05				
F8	0.04	1.34E-04	2.92E-04				
F91	0.03	3.82E-03	5.73E-03				
F22	0.02	1.09E-01	1.14E-01	BIBI	0.02	5.79E-05	5.79E-05
F46	0.03	5.05E-02	6.38E-02				
F56	0.02	7.49E-02	8.99E-02				
F65	0.02	1.65E-01	1.65E-01				

F78	0.02	8.18E-02	9.35E-02				
F99	0.02	1.03E-01	1.13E-01				

[A] Refers to the absolute percentage difference of the mean CpG methylation when mapped to Angus compared to Brahman for a given sample
[B] denotes sample-wise values calculated using all (21,432,071) CpG sites. Refers to Figure 3A.
[C] denotes group-wise values calculated using all CpG sites in all samples within a group (i.e. pooled) (21,432,071 $\times$ 6 = 128,592,426). Refers to Figure 3A.
[D] denotes sample-wise values using only shared (16,204,834) CpG sites. Refers to Figure 3B.
[E] denotes group-wise values calculated using only shared CpG sites in all samples within a group (i.e. pooled) (16,204,834 $\times$ 6 = 97,229,004) CpG sites. Refers to Figure 3B.

Table 4. Significant imprinted DEGs and their overlap with DMRs when using the Brahman reference genome.

Gene ID	Gene name	Protein name	Increased expression in Brahman*	Number of hypo-DMRs in Brahman	Number of hyper-DMRs in Brahman
Breed comparison					
ENSBIXG00005007073	*DSCAM*	DS cell adhesion molecule	No	61	18
ENSBIXG00005012203	*NNAT*	Neuronatin	No	37	1
ENSBIXG00005029958	*LIN28B*	Lin-28 homolog B	Yes	8	1
ENSBIXG00005009822	*DGAT1*	Diacylglycerol O-acyltransferase 1	Yes	3	2
ENSBIXG00005007141	*PPP1R9A*	Protein phosphatase 1 regulatory subunit 9A	Yes	11	5
Dam of origin comparison					
ENSBIXG00005019306	*ZC3H12C*	Zinc finger CCCH-type containing 12C	Yes	5	1
ENSBIXG00005007141	*PPP1R9A*	Protein phosphatase 1 regulatory subunit 9A	Yes	3	2
ENSBIXG00005029958	*LIN28B*	Lin-28 homolog B	Yes	1	0
ENSBIXG00005015804	*RTL1*	Retrotransposon Gag like 1	No	3	0
Sire of origin comparison					
ENSBIXG00005007073	*DSCAM*	DS cell adhesion molecule	No	38	5
ENSBIXG00005021735	*HTR2A*	5-hydroxytryptamine receptor 2A	Yes	18	2
ENSBIXG00005012203	*NNAT*	Neuronatin	No	8	0

ENSBIXG00005009822	*DGAT1*	Diacylglycerol O-acyltransferase 1	Yes	0	1
ENSBIXG00005025714	*MKRN3*	Makorin ring finger protein 3	No	0	0
ENSBIXG00005025694	*NDN*	Necdin MAGE family member	No	0	1
ENSBIXG00005024991	*SLC22A18*	Solute-carrier family 22 member 18	Yes	0	0
ENSBIXG00005013434	*TFPI2*	Tissue factor pathway inhibitor 2	No	25	0

* Increased expression in Brahman denotes genes that were significantly more highly expressed in Brahman than in Angus. “No” denotes that gene was significantly more highly expressed in Angus.

Table 5. Significant imprinted DEGs and their overlap with DMRs when using the Angus reference genome.

Gene ID	Gene name	Protein name	Increased expression in Angus*	Num of hypo-DMRs in Angus	Num of hyper-DMRs in Angus
Breed comparison					
ENSBIXG00000027129	Novel gene		Yes	20	66
ENSBIXG00000021864	*NNAT*	Neuronatin	Yes	2	32
ENSBIXG00000002586	*LIN28B*	Lin-28 homolog B	No	0	6
ENSBIXG00000012321	*DGAT1*	Diacylglycerol O-acyltransferase 1	No	3	3
ENSBIXG00000005197	*PPP1R9A*	Protein phosphatase 1 regulatory subunit 9A	No	9	15
Dam of origin comparison					
ENSBIXG00000011151	*ZC3H12C*	Zinc finger CCCH-type containing 12C	No	0	4
ENSBIXG00000005197	*PPP1R9A*	Protein phosphatase 1 regulatory subunit 9A	No	6	3
ENSBIXG00000002586	*LIN28B*	Lin-28 homolog B	No	0	1
Sire of origin comparison					
ENSBIXG00000027129	*DSCAM*	DS cell adhesion molecule	Yes	5	35
ENSBIXG00000008539	*HTR2A*	5-hydroxytryptamine receptor 2A	No	1	17
ENSBIXG00000021864	*NNAT*	Neuronatin	Yes	1	11
ENSBIXG00000012321	*DGAT1*	Diacylglycerol O-acyltransferase 1	No	0	0
ENSBIXG00000015087	*MKRN3*	Makorin ring finger protein 3	Yes	1	1
ENSBIXG00000015080	*NDN*	Necdin MAGE family member	Yes	1	0
ENSBIXG00000028529	*SLC22A18*	Solute-carrier family 22 member 18	No	0	2

* Increased expression in Angus denotes genes that were significantly more highly expressed in Angus than in Brahman. “No” denotes that gene was significantly more highly expressed in Brahman.

Figures

Figure 1. Overview of methods. A.) Representation of the four genetic groups used in this study. The blue cow represents pure Angus individuals (BTBT). The blue then orange cow represents individuals with an Angus sire and Brahman dam (BTBI). The orange then blue cow represents individuals with a Brahman sire and Angus dam (BIBT). The orange cow represents pure Brahman individuals (BIBI). **B.)** Process of mapping WGBS reads (light green-blue) and RNA-seq reads (green) to both the Brahman and Angus reference genomes. **C.)** Simple representation of shared and breed-specific CpG sites between Brahman and Angus reference genomes. **D.)** Breed-specific CpGs arise from a single nucleotide polymorphism between Brahman and Angus, such as spontaneous deamination of the C to a T. Structural variants, such as indels between the two genomes, can introduce or remove CpGs in one genome relative to the other. **E.)** Simple representation of how differential methylation can be influenced by breed-specific CpGs. The grey boxes demonstrate how a differentially methylated cytosine is identified when both breeds share that site. Essentially, one compares the number of Cs and Ts in group 1 against the number of Cs and Ts in group 2. If one group reports significantly more Cs than the other, it is considered differentially methylated. The yellow boxes represent a breed-specific CpG where only samples from one group have that CpG, so differential methylation cannot be determined. The red boxes represent a situation where the CpG is present in one subspecies, but spontaneous deamination has mutated the CpG site into a TpG site in the other subspecies. In this case, differential methylation can be calculated. However, it will be erroneous as only one group has a true CpG at that site. **F.)** Graphical representation of how breed differences were determined. We compared methylation and gene expression between BTBT and BIBI samples. **G.)** Graphical representation of how we determined parent-of-origin effects (POEs). Maternal POEs were determined by comparing BTBT and BIBT against BIBI and BTBI. Paternal POEs were determined by comparing BTBT and BTBI against BIBI and BIBT.

Figure 2. A.) PCA plot showing separation of genetic groups by methylation. Blue represents BTBT, orange represents BIBI, green represents BTBI and red represents BIBT. The X axis is principal component 1, and the Y axis is principal component 2. **B.)** PCA plot showing separation of genetic groups by gene expression data; colours are same as **A**. The X axis is the first dimension of the logCPM, and the Y axis is the second dimension of the logCPM.

Figure 3. A.) Strip plots showing mean global CpG methylation for all samples in the four genetic groups. Each panel represents a genetic group in the order BTBT, BTBI, BIBT and BIBI. The X-axis represents the reference that was used for mapping. The Y-axis represents the methylation percentage. Each sample is represented by a different colour. When Brahman and Angus are mapped to their respective genomes, they tend to be more methylated than when mapped to the incorrect reference. The hybrids (BTBI and BIBT) tend toward hypermethylation when mapped to the Angus reference. P-values can be found in Table 3 **B.)** Strip plots showing mean global CpG methylation for all samples belonging to each of the four genetic groups mapped to each reference genome; however, this time, only the shared CpGs were considered. P-values can be found in Table 3. As with **A**, the X-axis represents the reference genome used for alignment, and the Y-axis represents the methylation percentage **C.)** Scatterplots showing the methylation values of shared CpGs when mapped to the Angus reference (X-axis) and Brahman reference (Y-axis) for chromosome 29. Each point on the plot represents a single CpG site for a given sample. Each panel represents a different genetic group. The top row contains BTBT and BTBI, and the bottom row contains BIBT and BIBI. Orange dots represent CpG sites with an absolute methylation percentage difference $\leq$10% difference between reference genomes. Blue dots represent CpG sites that differ by >10% absolute methylation percentage difference. **D.)** Boxplots illustrating the mean frequencies of methylation states observed in Angus and Brahman after 100 permutations. Blue represents hypomethylated CpG sites (methylation $\leq$ 25%). Orange bars represent hypermethylated CpG sites (methylation $\geq$ 75%). P-values were determined with a Mann-Whitney U-test. The X-axis denotes the breed, either Angus or Brahman. The Y-axis represents the count, i.e., the number of sites that fell into the hypo- or hypermethylated categories. **E.)** Same as **D,** but hypomethylated sites were $\leq$ 35%, and hypermethylated sites were $\geq$ 65%. P-values were determined with a Mann-Whitney U-test.

Figure 1

Click here to access/download;Figure;Figure 1 - methylation-Overview_figure.png

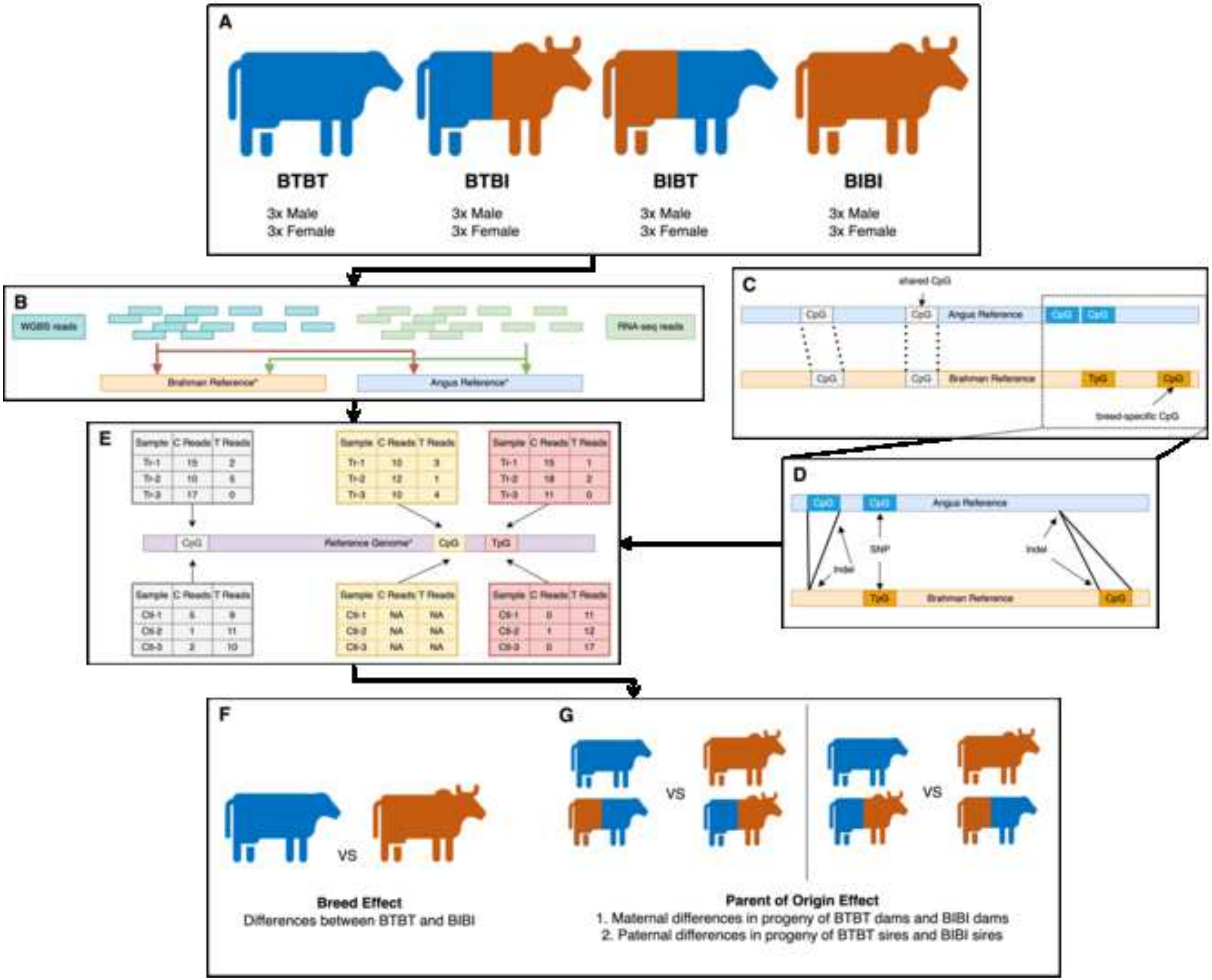

Figure 2

Click here to access/download;Figure;Figure 2 - PCA.Brahman.meth_and_rna.png

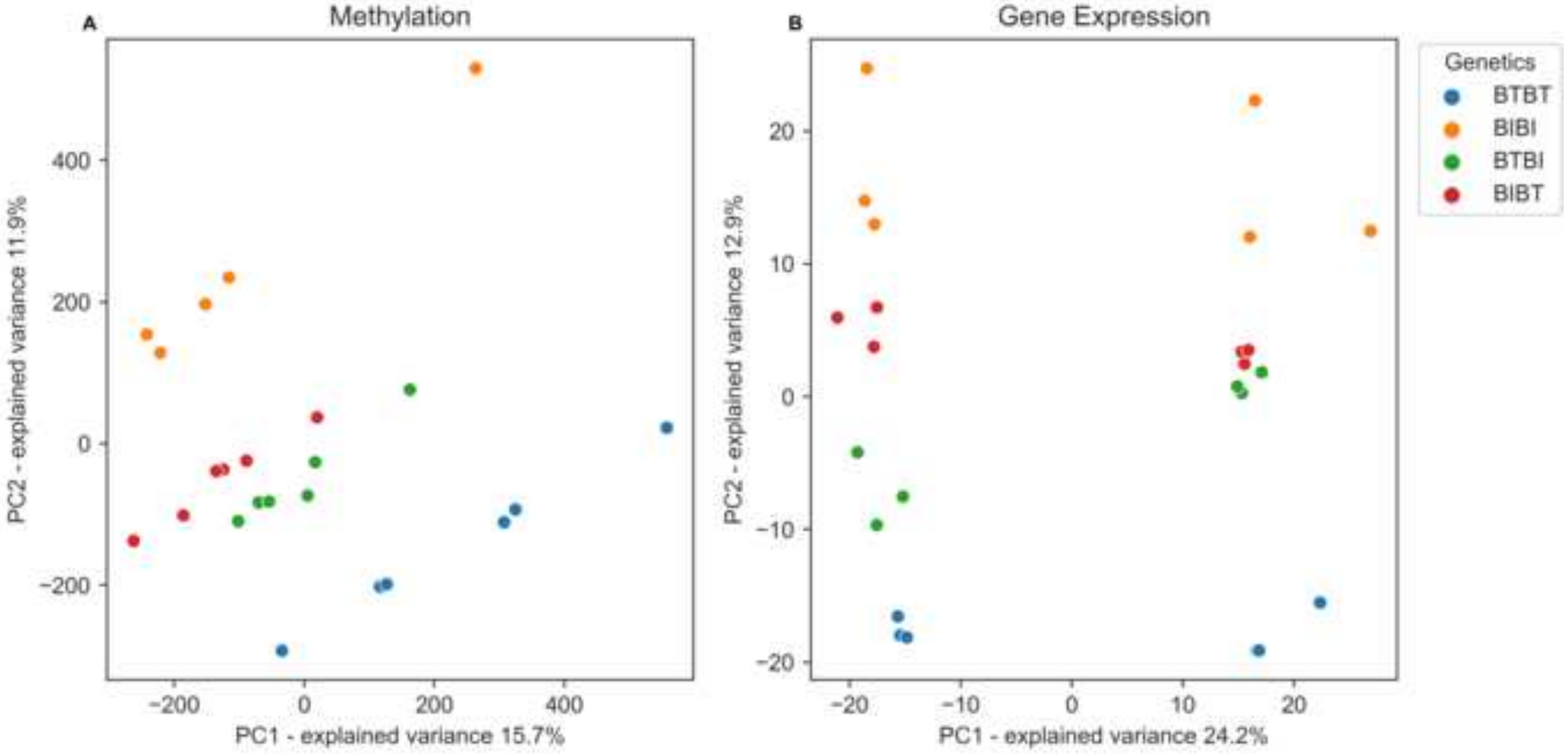

Click here to access/download;Figure;figure3.pdf

Figure 3

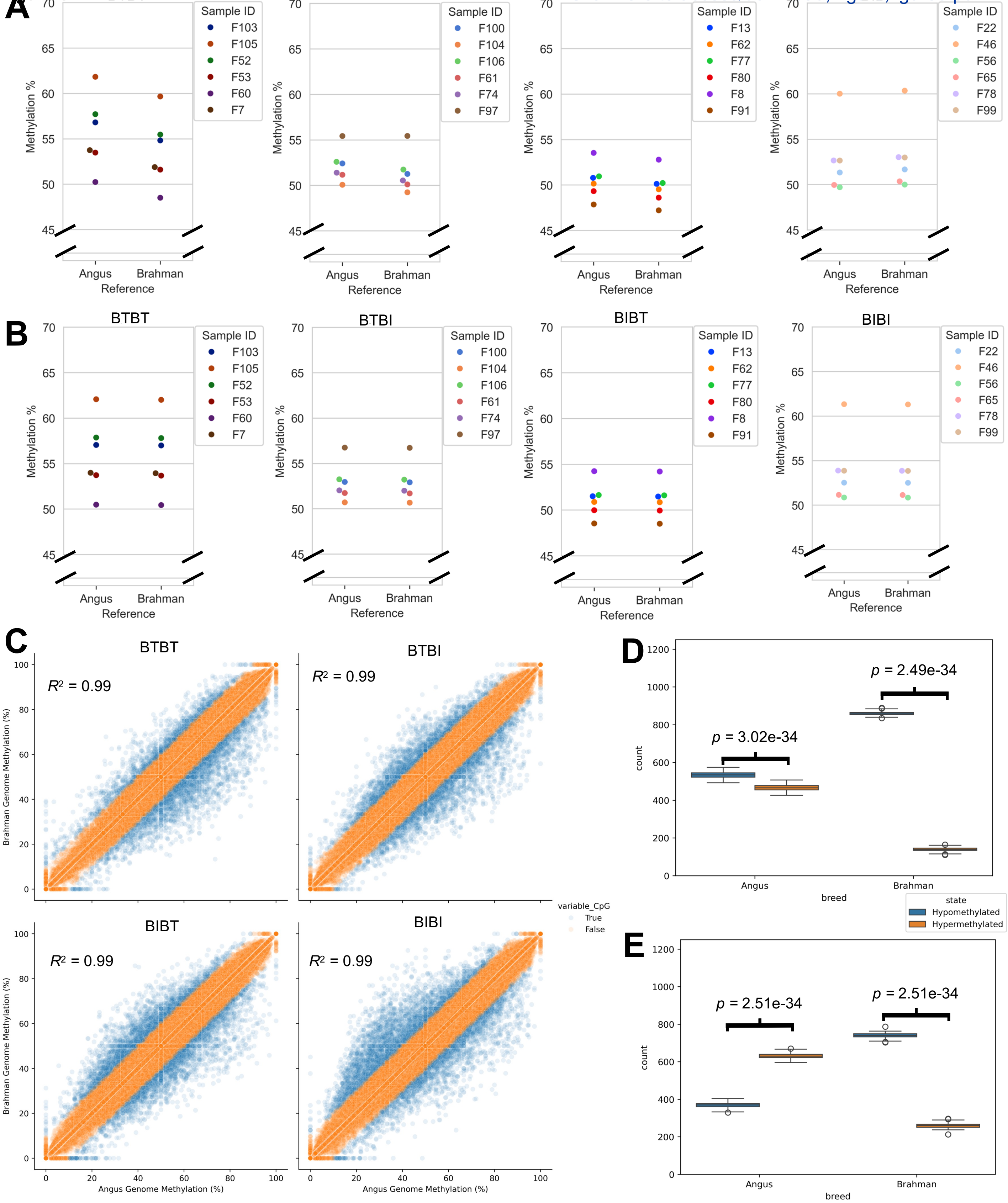

Supplementary table 1

Click here to access/download
Supplementary Material
S. table 1 - WGBS mapping stats.xlsx

Supplementary table 2

Click here to access/download
Supplementary Material
S. table 2 - global coverage stats.xlsx

Supplementary table 3

Click here to access/download
Supplementary Material
S. table 3 - CpG coverage stats.xlsx

Supplementary table 4

Click here to access/download

Supplementary Material

S. table 4 - global methylation.xlsx

Supplementary table 5

Click here to access/download
Supplementary Material
S. table 5 - Descriptive stats regional meth.xlsx

Supplementary table 6

Click here to access/download
Supplementary Material
S. table 6 - CpG Mapping between refs.xlsx

Supplementary table 7

Click here to access/download
Supplementary Material
S. table 7 - SNPs and SV enrichment.xlsx

Supplementary table 8

Click here to access/download
Supplementary Material
S. table 8 - CpG reference bias.xlsx

Supplementary table 9

Click here to access/download
Supplementary Material
S. table 9 - variable_cpgs.xlsx

Supplementary table 10

Click here to access/download
Supplementary Material
S. table 10 - meth change.xlsx

Supplementary table 11

Click here to access/download
Supplementary Material
S. table 11 - DMRs.xlsx

Supplementary table 12

Click here to access/download
Supplementary Material
S. table 12 - DEGs_DESeq2.xlsx

Supplementary table 13

Click here to access/download
Supplementary Material
S. table 13 - minimap2_accuarcy.xlsx

Supplementary table 14

Click here to access/download
Supplementary Material
S. table 14 - sample info.xlsx

Supplementary table 15

Click here to access/download
Supplementary Material
S. table 15 - imprinted_genes.xlsx

Supplementary figures

Click here to access/download
Supplementary Material
Supplementary figures.docx

Click here to access/download
Supplementary Material
Response to reviewers_v3.docx
