# Supplementary material for: DNA methylation analysis to differentiate reference, breed, and parent-of-origin effects in the bovine pangenome era
Source: Gigascience. 2024 Oct 17;13:giae061. doi: 10.1093/gigascience/giae061 (PMC11484048; doi:10.1093/gigascience/giae061)
Supplement: giae061_GIGA-D-23-00314_Revision_2 [file giae061_giga-d-23-00314_revision_2.pdf]

GigaScience

DNA methylation analysis to differentiate reference, breed and parent-of-origin effects in the bovine pangenome era

--Manuscript Draft--

**Manuscript Number:**	GIGA-D-23-00314R2
**Full Title:**	DNA methylation analysis to differentiate reference, breed and parent-of-origin effects in the bovine pangenome era
**Article Type:**	Research
**Funding Information:**	JS Davies Bequest — Not applicable
**Abstract:**	Background<br>Most DNA methylation studies have used a single reference genome with little attention paid to the bias introduced due to the reference chosen. Reference genome artefacts and genetic variation, including single nucleotide polymorphism (SNPs) and structural variants (SVs), can lead to differences in methylation sites (CpGs) between individuals of the same species. We analysed whole genome bisulfite sequencing (WGBS) data from the fetal liver of Angus (Bos taurus taurus), Brahman (Bos taurus indicus) and reciprocally crossed samples. Using reference genomes for each breed from the Bovine Pangenome Consortium, we investigated the influence of reference genome choice on the breed- and parent-of-origin effects in methylome analyses.<br>Results<br>Our findings revealed that ~75% of CpG sites were shared between Angus and Brahman, ~5% were breed-specific, and ~20% were unresolved. We demonstrated up to ~2% quantification bias in global methylation when an incorrect reference genome was used. Furthermore, we found that SNPs impacted CpGs 13 times more than other autosomal sites (p-value <) and SVs contained 1.18 times (p-value <) more CpGs than non-SVs. We found a poor overlap between differentially methylated regions (DMRs) and differentially expressed genes (DEGs) and suggest that DMRs may be impacting enhancers that target these DEGs. DMRs overlapped with imprinted genes, of which one, DGAT1, which is important for fat metabolism and weight gain, was found in the breed-specific and sire-of-origin comparisons.<br>Conclusions<br>This work demonstrates the need to consider reference genome effects to explore genetic and epigenetic differences accurately and identify DMRs involved in controlling certain genes.
**Corresponding Author:**	Callum MacPhillamy<br>The University of Adelaide<br>Roseworthy, SA AUSTRALIA
**Corresponding Author Secondary Information:**	
**Corresponding Author's Institution:**	The University of Adelaide
**Corresponding Author's Secondary Institution:**	
**First Author:**	Callum MacPhillamy
**First Author Secondary Information:**	
**Order of Authors:**	Callum MacPhillamy<br>Tong Chen<br>Stefan Hiendleder<br>John Williams<br>Hamid Alinejad-Rokny<br>Wai Low

Powered by Editorial Manager® and ProduXion Manager® from Aries Systems Corporation

Order of Authors Secondary Information:	
Response to Reviewers:	PLEASE NOTE: We hav also attached our responses as a word document.<br><br>Response to reviewers – second round.<br>Authors responses in BLUE.<br><br>We acknowledged the authors provide additional summaries and test results, which partially addressed the question. As for the visualization (Figure 3A/3B), we agreed the global mean is useful. But we still humbly suggest displaying the distributions (e.g. density or ridge plot) if possible, which provides a more comprehensive view of the data. Our suggestion stems from the following consideration: Global mean may be a good representation when the data follows a Gaussian distribution. But when the underlying distribution is bimodal (e.g. in human's DNA methylation data, most of the sites' methylation level are either close to 0 or 1), global mean is no longer sufficient to represent the whole data. By knowing the global mean, we still cannot say much about the data distribution. As for implementation, If the key point is to display the reference genome's effects on quantification, the authors can consider keeping the current panel layout, where each subplot still represents a group (BTBT, BTBI, etc). Within each subplot, samples in the same group with the same reference genome are summarized by average, leading to the averaged methylation level per site, thus a methylation distribution of all sites can be plotted using a density curve for each reference genome. Such treatment enables a statistical comparison where the significance of 2 density curves' difference can be computed using Kolmogorov-Smirnov test, echoing the quantification bias introduced by reference genome, if there is any. Notably, the global mean (or median) can also be annotated onto the density curve using vertical lines. In that sense, the suggested figure still contains global mean information. Although the per-sample differences (shown in current Figure 3A/3B) are not present in the suggested figure, they are less informative with respect to the key message and can be considered as supplementary figure.<br><br>We appreciate the reviewer's detailed comments and suggestions regarding the presentation of figure 3. We have replotted figure 3 as a series of kernel density estimate plots as suggested. Moreover, we also calculated a 2 sample Kolmogorov-Smirnov test for each group to test whether the methylation distribution was different within a given group when using a different reference genome. We have included this at lines 252-277.<br><br>"We examined the CpG methylation differences between each sample when mapped to Angus and Brahman reference genomes and observed a statistically significant difference in CpG methylation levels in all samples when using all CpGs with 10X coverage (two-sample Kolmogorov-Smirnov test, adjusted p-value < 0.05) (Table 3; S. table 8). When only considering the shared CpGs, we observed a significant quantification bias in all samples, though it was much smaller (two-sample Kolmogorov-Smirnov test, adjusted p-value < 0.05) (Table 3; S. table 8). When we pooled all CpG methylation values for a given group and compared the Brahman and Angus references, we observed a statistically significant quantification bias in all four groups (two-sample Kolmogorov-Smirnov test, adjusted p-value < 0.05) (Figure 3A; Table 3; S. figure 3A; S. table 8). When comparing only the shared CpGs, we observed a much weaker quantification bias between genomes, though it was still significant at the group level (two-sample Kolmogorov-Smirnov test, adjusted p-value < 0.05) (Figure 3B; Table 3; S. figure 3B; S. table 8)."<br><br>Inspecting the per-sample differences in Figure 3A/3B, we noticed there is always one sample that has bigger global mean than the rest in each group no matter what reference used (such as sample F97, F8, F46, which behave like outliers as the rest are closer to each other). Is there a potential explanation for this phenomenon? Is it due to biological (origin/age) or technical (different coverage/sequencing depth) reasons?<br><br>It is possible those samples had somewhat different sampling from the others. There could be differences in timing, type and number of cells collected from each liver. These spatiotemporal factors may be influencing the difference in methylation of these samples. However, we are not sure why these three samples tend to have a higher global methylation mean than the other samples.

Per author’s comment “a violin plot that spans from 0 – 100% methylation with the widest point around the global mean”, this makes us wonder what the distribution/density curve looks like and why it’s different from other mammals. In other mammals (mouse, human, dog), the majority sites are around 1 or 0, few of them are in the middle, displaying a bimodal distribution. Is it due to the species / tissue / age difference?

We do see a bimodal distribution, though the peaks are around 0 and 0.6 rather than 0 and 1. It could be a combination of tissue and age causing this particular bimodal distribution of methylation. He et al. (2020) investigated methylation levels in the developing mouse embryo across a variety of tissues. For most tissues and timepoints from embryo day 10.5 to adult, the authors observed global methylation levels between 70-80%. Liver was the exception, with global methylation levels dropping to ~60-65% methylation from embryonic day 12.5-16.5. This developmental timepoint in mice is similar to the developmental timepoint of the foetuses in the present study, suggesting the observed methylation pattern may be a hallmark of fetal liver development. At day 153, the cattle fetus begins log phase growth and so cell division is high. Given that hemimethylation increases during DNA synthesis, it is possible that this higher degree of hemimethylation is drawing the global mean methylation more toward 0.5 than 1. We cannot find any comparable studies in cattle with which we can compare our findings to.

We hold a different opinion with author’s argument on mutation rate. Specifically, if we assume the single nucleotide’s mutation rate is uniform across nucleotides (denoted as p) and the mutation events are independent. Then for a dinucleotide, the probability of **not** carrying a mutation is (1-p)^2. In other words, the probability of containing a mutation in a dinucleotide is **1-(1-p)^2**, which is different from the used background mutation rate (p). Such calculation, although oversimplified by making assumptions, shows the current procedure might not be precise.

Nevertheless, since the authors obtained a set of SNPs and want to test if they are enriched in CpG sites. We think an appropriate test is to construct a 2 by 2 contingency table, where the rows record whether a base is in CpG context, and columns denote whether that base is a SNP site or not. The enrichment p-value can then be calculated using chi-squared test.

SNPNot SNPTotal
In CpGaba+b
Not In CpGcdc+d
Totala+cb+da+b+c+d

For SV’s enrichment, if the goal is to test whether the proportion of CpGs in SV region is higher than that of non-SV region, a natural idea is to calculate the proportions of CpGs for each SV region and each non-SV region respectively, with these 2 groups of proportions, the authors can perform a t-test or Wilcoxon test.

We think these test approaches are better than the binomial test currently employed by the authors since the binomial test heavily reply on the assumptions that the true background probability is known and the CpG occurrence events are independent (that’s why a 1.18-fold will yield p-values as small as lower than 5*10^-324). Such assumptions are too strong and might easily fail in reality, especially for the first one which used the estimated background probability as the true probability and ignores the risk that the background probability could be far from the unobserved truth. The tests we suggested above for SNP and SV respectively have weaker assumptions and should be more suitable for calculating the enrichment significance.

Besides, the enrichment fold is 14 and 1.18 for SNP and SV in the manuscript but is 8.3 and 1.13 in the Jupyter Notebook, we suggested to check and update accordingly.

The Jupyter Notebook was not updated at the time you read it, now it is. The fold

Powered by Editorial Manager® and ProduXion Manager® from Aries Systems Corporation

	enrichment is 14 and 1.18 for SNP and SV, respectively. The authors appreciate the suggestion and effort taken by the reviewers to provide suggestions for this analysis. As suggested, we reanalysed the SNP enrichment, using a 2x2 contingency table, followed by a Chi-square test. We have amended the manuscript at lines 238-241 and lines 654-660 to reflect these changes.<br><br>Lines 238-241:<br>“SNPs in CpG sites were enriched by ~13 times compared to the genomic background (Chi-square test of independence, adjusted p-value < 5×10^(-324)), which means SNPs between these two breeds affect CpG sites disproportionately more than other autosomal sites.”<br><br>Lines 654-660:<br>“We constructed 22 contingency tables to perform a Chi-square test of independence per chromosome to determine whether CpGs had a higher likelihood of being affected by a SNP than non-CpGs. The four categories where ‘CpG affected by SNP’, ‘non-CpG affected by SNP’, ‘unaffected CpG’ and ‘unaffected non-CpG’. We then adjusted the p-values to correct for multiple testing using the Benjamini-Hochberg correction procedure.”<br><br>We have also reanalysed the SV CpG enrichment results as suggested and have amended the manuscript at lines 251 to 252 and lines 669-675.<br><br>Lines 251-252:<br>“We observed 1.18-fold more CpGs within SVs than in non-SVs (Mann Whitney U-test, p-value = 8.91×10^(-5)).”<br><br>Lines 669-675:<br>“We then counted the number of CpGs that occurred in these regions on each chromosome and divided them by the combined length of all SVs on the given chromosome to determine the proportion of CpGs occurring in the SV regions; this value was added to a vector. We repeated this for CpGs occurring in non-SV regions, adding the proportions to a difference vector. With these two vectors we then performed a Mann-Whitney U test to determine whether SVs were more likely to introduce CpGs than non-SV regions.”<br><br>The authors provided additional data (Figure 3C and tables) which can effectively address our question in 6b. We only have small plot suggestions. The current scatter plot cannot show where the data points mainly accumulate around, although authors tried to adjust the dot’s transparency, but with too many overlapped points, it failed to deliver such information. A replacement with 2d density plot with a diagonal dashed line is suggested. Such density plot is useful to reflect what would happen to most sites if a different reference genome were used (e.g. if the density peak lies above the diagonal line, it shows that using the alternative reference genome would generally inflate the methylation levels). With density plot, all CpG sites can be plotted instead of only that of chromosome 29 as the figure 3C currently shows.<br><br>Besides the R2, we recommend reporting the RMSE on the figure, this is because if the key message is that reference genome brings methylation quantification difference, then the overall difference resulted by reference genome is more cared about than the concordance (represented by R2).<br><br>We appreciate the suggestion to change Figure 3C to a 2D density plot. We have replotted figure 3C as a KDE plot. While we did use all chromosomes, we present only CpGs that were different by > 10 in Figure 3C. The figure using all CpGs from all chromosomes can be found in supplementary figure 4. We have updated the manuscript at lines 282-284.<br><br>“We observed RMSE values of around 0.01 for groups (S. figure 4), with most CpGs exhibiting less than 10% difference in methylation between the reference genomes.”

Powered by Editorial Manager® and ProduXion Manager® from Aries Systems Corporation

The authors provided additional description and increased clarity, thus addressed our first question/concern in 6d.

For the second and third part, our point is that if we can map the features from one reference genome to another (For shared CpG sites, it’s the coordinate mapping derived by the authors; For shared genes, the gene id or gene name), then the methylation level /gene expression matrix that are quantified using one reference genome can be lifted to another reference genome. As a result, the same sample called on different reference genomes can be compared and analyzed on the shared sites/genes. (by treating the quantification results of same biological sample from 2 different reference genomes as paired samples, the usual DMR or DEG analysis strategy can be applied)

Based on the manuscript that less than 2% of shared CpG sites exhibits >=10% methylation when different reference genome used, we speculate there might be few DMRs when comparing the same samples between 2 different reference genomes. Does this suggest that Brahman/Angus reference genome choices are mostly consistent on downstream differential analysis results for the shared sites or genes? And the observed difference is mainly caused by the breed specific CpGs/genes? If that’s the case, the authors should explicitly report/discuss it.

We thank the reviewers for their detailed responses. To address this comment, we made non-overlapping 100kb windows of each genome and aligned them to one another. We then filtered the alignments to retain only those that aligned to the same chromosome (e.g. a window from chromosome 1 in Angus aligned to a region of chromosome 1 in Brahman) and those with mapq = 60. With this completed for both references, we could then match 100kb windows between Brahman and Angus. It should be noted here that ~2000 of these windows could not be aligned, thus immediately demonstrating a reference genome effect. For the remaining windows that could be compared, we performed differential methylation analysis. We have updated the manuscript at lines 303-308 and lines 694-710.

Lines 303-308:
“Lastly, we compared homologous regions between the Angus and Brahman reference genomes to determine whether any differentially methylated regions existed between these two genomes for each group (Methods). Using a window size of 100kb, we observed 41, 31, 30 and 36 windows being significantly differentially methylated when using BTBT, BTBI, BIBT and BIBI samples, respectively (paired Wilcoxon test, adjusted p-value < 0.05) (S. Table 11).”

Lines 694-710:
“To further assess the presence of a quantification bias, we generated non-overlapping windows of both Angus and Brahman reference genomes with a length of 100 kb. We then aligned these 100 kb sequences to the opposite reference genome with `minimap2 –t 8 –c –x map-ont`. We then used the output PAF files to identify homolgous 100 kb windows from each genome. We extracted the methylation values from each window for a given group when mapped to each genome. For example, consider homologous-region “A” (HR-A), identified between the Angus and Brahman reference genomes. To identify whether this region exhibits differential methylation between reference genomes and thus exhibits quantification bias, we quantified all methylation values that fell within the Angus coordinates of HR-A using the methylation values for BTBT samples mapped to the Angus reference. We then quantified the methylation values that fell within the Brahman coordinates of HR-A using the methylation values for BTBT samples mapped to the Brahman reference. This gave two vectors of methylation values, which were then used as input to a Wilcoxon signed-rank test to determine whether the methylation values in this region were significantly different between genomes. The Benjamini-Hochberg false discovery procedure was used to adjust for multiple testing. This was repeated for each of the four groups.”

We reckoned there might be some miscommunications on this question. Let us rephrase the question. This section titled “Breed-specific CpGs show distinct

Powered by Editorial Manager® and ProduXion Manager® from Aries Systems Corporation

methylation patterns". By "distinct", we assume the authors imply one or both of the following: 1. the methylation pattern in breed-specific CpGs is different from that of the breed-shared CpGs, 2. the methylation pattern in Brahman-specific CpGs is different from that of the Angus-specific CpGs.

To support the first claim, the authors would need to directly compare the breed specific CpGs methylation levels to the baseline background (shared CpGs) and see if there is a difference and what's the direction of the difference. To support the second claim, the authors should compare the Brahman-specific CpGs to that of Angus-specific CpGs. Either claim should involve comparing the original values (without bining) of the entire set of CpGs (without subsampling).

Nevertheless, the current procedure, first binned the methylation level into hyper/hypo-methylation by thresholding and then repeated sampling to count the occurrence of hyper/hypo-methylated sites, is essentially showing if the count (or proportion) of hypomethylation sites is higher/lower than that of hypermethylation sites for a given set. Take the figure 3D as an example, the figure shows for breed-specific CpGs, #hypomethylation sites is more than the #hypermethylation sites in both breeds. Our original review questions are: 1. The findings seem to be sensitive to the choice of hyper/hypo-methylation threshold, how was the threshold decided, will the findings still hold if a different threshold used? 2. Using the same threshold, will we get the same findings (#hypomethylation sites is more than the #hypermethylation sites) on the breed-shared sites? If the answer is yes, the authors probably shouldn't claim it to be a distinct pattern for breed-specific sites, since the background (shared sites) also have the same pattern.

In the first response, the authors adjusted the threshold from to 0.25/0.75 and found one reference genome displayed a opposite pattern. This confirmed our first concern that the findings are not robust to threshold choices, leading to concerns that the conclusion claim is not solid or sound. As a result, the current analysis and response **did not** fully resolve the previous questions/concerns.

Additionally, as we denoted above, the current testing procedure (repeated-sampling + Mann-Whitney U-test) is essentially trying to validate if the proportion of hypermethylation sites is the same as that of hypomethylation sites. But this procedure has an issue that the Mann-Whitney U-test's iid assumption will no longer hold under the repeated-sampling procedure. With sufficient repetitions, this procedure will give very small p-values even if the proportion difference is minute. We suggested to switch to testing for the entire set of sites using chi-square test (if the binning procedure is used) or the Wilcoxon test (if the raw methylation values are used).

We have replotted this figure using the raw methylation values via a KDE plot and performed a Wilcoxon test as suggested. We have amended the manuscript at lines 311-319 and lines 1345-1354.

Lines 311-319:
"Looking more closely at the breed-specific CpGs, we first determined the background methylation for Angus samples as the methylation of all shared CpG sites when mapped to the Angus reference. We then extracted the methylation for all Angus samples from CpG sites that were identified as breed-specific. We then compared the background methylation distribution against the Angus-specific distribution. This was repeated for the Brahman using the Brahman reference. Breed -specific CpG sites were significantly more likely to have lower methylation than the background methylation for Angus (Wilcoxon signed-rank test, p < 5.0 ×10^(-324)) and Brahman (Wilcoxon signed rank-test, p < 〖5.0×10〗^(-324)), respectively (Figure 3D-E)."

Lines 1345-1354:
"…D.) KDE plot illustrating the illustrating the difference in methylation distribution between the shared and Angus-specific CpG sites when aligning Angus samples to the Angus reference. Blue represents shared CpG sites. Orange represents Angus-specific CpG sites. P-values were determined with a Wilcoxon signed-rank test. The X-axis denotes the methylation percentage. The Y-axis represents the probability density. E.) KDE plot illustrating the illustrating the difference in methylation distribution between the shared and Brahman-specific CpG sites when aligning Brahman samples

to the Brahman reference. Blue represents shared CpG sites. Orange represents Brahman-specific CpG sites. P-values were determined with a Wilcoxon signed-rank test. X- and Y-axes are the same as D."

The author provided that high number of DEGs (>88%) overlapped with DMR, which eliminates the concern about artifacts. Since the canonical correlation we suggested is just a sanity check on data quality, we recommend putting it to supplementary and representing the strength of association by canonical correlation (not R2) with p-values provided, to be consistent with other correlations of the manuscript.

We have created a new supplementary table, 17, to show the CCA results and updated the main text accordingly at lines 796-797.

"Furthermore, we performed canonical correlation analysis (CCA) to estimate the canonical correlation between the gene expression and methylation data. The first five canonical correlations were >0.7 with p-values < 0.05 (S. Table 17)."

"A model without an intercept term would only be recommended in cases where there is a strong biological reason why a zero covariate should be associated with a zero-expression value, and such contexts are rare in gene expression modelling." Is there a special reason why the intercept is forced to be 0?

We forced the intercept to be zero as we are comparing the gene expression between groups as we implemented the groups-mean parametrisation method to calculate the differentially expressed genes. We have previously published with this method in Liu, Tearle [1]. We have specified this in the main text at lines 788-790.

"The group-mean parameterisation method was used to identify differentially expressed genes between groups with the following model design: '~0 + Genetics + Sex + Batch' [1]."

According to supplementary figure, the putative enhancer region is 25 times longer than the promoter region. It's not suitable to implicitly suggest a direct comparison between 73% with 0.3%, we suggest adding a short phrase describing the relative fold after normalizing by length.

We have now reported the relative difference between putative enhancer regions and promoter region overlapping DMRs with normalised lengths in each of their respective sections. We have also reported them in a supplementary table (S. table 14).

"Of the 123,602 DMRs observed, ~19% (23,575) overlapped with the surrounding region of the significant DEGs, with around twice as many DMRs falling within the putative-enhancer region compared to the promoter region after normalising for the length difference between the enhancer regions and promoter regions (S. table 14)."

These reciprocal overlap results could be organized into a table if that makes the result look clearer.

We have added S. table 14 to help improve the clarity of the reciprocal overlap results.

The explanation is acknowledged, but we are asking a different question. To illustrate, when deciding whether a region/site is significantly different, we need to compare its p-value to a threshold (e.g. 0.05). When there are many regions/sites to be decided, many p-values need to be compared, then the p-value adjustment (e.g. Bonferroni correction, BH procedure) is needed to account for the multiple comparison problem and control the False discovery rate (FDR).
Back to the question, we speculate the no-findings for DMC result is due to the stringent multiple-testing procedure. Some clarification on the p-value calculation and adjustment method is needed (what test is used? Is the p-value adjusted? if the answer is yes, what adjustment is being used when deciding DMR/DMC?) since there is no such information throughout the manuscript.

We thank the reviewers for their comments. We suspect there has perhaps been a

misunderstanding. We identified DMCs using the same method we identified DMRs, though without the sliding window approach. The DMCs were identified using the logistic regression and the p-values were adjusted using a sliding linear model (SLIM) to estimate the proportion of true null hypotheses among the DMC and DMRs. We've amended the methods at lines 753-755 to make this clearer.

"Q-values were obtained using the sliding linear model (SLIM) method [2]. We repeated this pipeline to identify DMCs, with the exclusion of the tiling step."

Line 28-30: "Genetic variation,including single nucleotide polymorphism (SNPs) and structural variants (SVs), can lead to differences in methylation sites (CpGs) between individuals of the same species". This sentence is a bit ambiguous as the differences can be resulted either by mQTL or by reference genome's artifact. It's better to be specific and precise.

We have changed this to: "Reference genome artefacts and genetic variation,…"

Line 39-40: "Furthermore, we found that SNPs and SVs were 14-fold (p-value < 5×10^{-324}) and 1.18-fold (p-value < 5×10^{-324}) higher in CpGs,". The sentence is a bit confusing as the proportion of CpGs is higher in SVs, not the other way around.

We have updated lines 39-41 to improve clarity.
"Furthermore, we found that SNPs impacted CpGs 13 times more than other autosomal sites (p-value < 5×10^(-324)) and SVs contained 1.18 times (p-value < 5×10^(-324)) more CpGs than non-SVs."

Line 41-43: "We found a poor association between differentially methylated regions (DMRs) and differentially expressed genes (DEGs) and suggest that DMRs may be impacting enhancers that target these DEGs". The "association" is suggested to be replaced by "overlap" to avoid confusion.

We have updated the line as suggested at line 41.

"We found a poor overlap between differentially methylated…"

Line 162-164: Only the average mapping rates for Brahman reference genome is reported, we suggest also write out the mapping rate of Angus to make it complete.

Done. "An average mapping rate of ~95% was achieved when reads were mapped from each sample to the Brahman reference genome and ~93% when mapping to the Angus reference genome (Table 1; S. table 1)."

Line 196-197: "indicating a potential Parent-of-origin effects" can be added to the end of the paragraph.
Done. "The separation of groups we observed from the methylation data was similar to that seen for the gene expression data (Fig 2B), indicating a potential parent-of-origin effect."

Line 219: "four per cent" -> 4%, to be consistent with the format in line 223/225.

We have changed to 4% as suggested.

"We found that around 4% of CpG alignments…"

Line 313: "CpGs that were between 35% and 65% were considered "hemimethylated"". "hemimethylated" specifically refers to the methylation pattern of CpG sites (one strand (forward/reverse) is methylated while the other strand (reverse/forward) is not). We recommend changing to another word that more precisely denotes the sites whose methylation level lies in between the binning threshold.

This has been addressed above.
Lines 311-319:
"Looking more closely at the breed-specific CpGs, we first determined the background

methylation for Angus samples as the mean methylation of all shared CpG sites when mapped to the Angus reference. We then extracted the methylation for all Angus samples from CpG sites that were identified as breed-specific. We then compared the background methylation distribution against the Angus-specific distribution. This was repeated for the Brahman using the Brahman reference. Breed -specific CpG sites were significantly more likely to have lower methylation than the background methylation for Angus (Wilcoxon signed-rank test, p < 5.0 ×10^(-324)) and Brahman (Wilcoxon signed rank-test, p < 〖5.0×10〗^(-324)), respectively (Figure 3D-E).”

Line 492: “Most DMRs identified in this study were not associated with DEGs”, we suggested to replace “associated” with “overlapped” to make it more precise.

Completed at line 483. “Most DMRs identified in this study did not overlap with DEGs.”

Some of url links (line 595/597/607) are underlined while (610/613/614) some are not, it’s better to keep it consistent throughout the paper.

Done. All URL links have been removed (i.e. they are plain text now).

Line 628: 102bp -> 1002bp

102bp is correct at this line. This was the length of the query sequence when we performed the local alignment within the ~1002bp block identified by minimap2.

Line 640: “To determine the accuracy of minimap2 in identifying SNPs”, minimap2 is a read aligner not a SNP-caller, this sentence is not precise. Throughout the manuscript, minimap2 is constantly referred as a SNP-caller, we suggest the authors revise thoroughly and make necessary corrections.

We have updated the manuscript as follows to improve the clarity of this section:
“To determine the accuracy of minimap2 alignments in identifying SNPs between the Brahman and Angus genomes, we first introduced artificial mutations into each genome using SNP Mutator [103].”

“Alignments from Mminimap2 [59] and were used as input to PAFtools which was were used to identify variants between the original and mutated sequences.”

Table1: “Mapped reads” and “CpGs covered with >= 10x” can be formatted as “counts (percentage)”, currently it only displayed the counts

Done.

Supplementary Figure2 legend: “with “other” SNPs making up ~19% of SNPs.” -> with other alternations making up ~19% of SNPs.

Done.

Figure 3D’s legend “D.) Boxplots illustrating the mean frequencies of methylation states observed in Angus and Brahman after 100 permutations.”. This is confusing since there is no permutation being done. The sub-sampling procedure is not equivalent to permutation.

The figure has been removed. We believe this has been resolved when we addressed prior comments.

1.Liu R, Tearle R, Low WY, Chen T, Thomsen D, Smith TPL, et al. Distinctive gene expression patterns and imprinting signatures revealed in reciprocal crosses between cattle sub-species. BMC Genomics. 2021;22 1 doi:10.1186/s12864-021-07667-2.
2.Wang H-Q, Tuominen LK and Tsai C-J. SLIM: a sliding linear model for estimating the proportion of true null hypotheses in datasets with dependence structures. Bioinformatics. 2010;27 2:225-31. doi:10.1093/bioinformatics/btq650.

Additional Information:

Powered by Editorial Manager® and ProduXion Manager® from Aries Systems Corporation

Question	Response
Are you submitting this manuscript to a special series or article collection?	No
**Experimental design and statistics**<br><br>Full details of the experimental design and statistical methods used should be given in the Methods section, as detailed in our Minimum Standards Reporting Checklist. Information essential to interpreting the data presented should be made available in the figure legends.<br><br>Have you included all the information requested in your manuscript?	Yes
**Resources**<br><br>A description of all resources used, including antibodies, cell lines, animals and software tools, with enough information to allow them to be uniquely identified, should be included in the Methods section. Authors are strongly encouraged to cite Research Resource Identifiers (RRIDs) for antibodies, model organisms and tools, where possible.<br><br>Have you included the information requested as detailed in our Minimum Standards Reporting Checklist?	Yes
**Availability of data and materials**<br><br>All datasets and code on which the conclusions of the paper rely must be either included in your submission or deposited in publicly available repositories (where available and ethically appropriate), referencing such data using a unique identifier in the references and in the “Availability of Data and Materials” section of your manuscript.	Yes

Powered by Editorial Manager® and ProduXion Manager® from Aries Systems Corporation

Have you have met the above requirement as detailed in our Minimum Standards Reporting Checklist?	

Manuscript

Click here to access/download;Manuscript;Methylation_paper_2nd_round_r

DNA methylation analysis to differentiate reference, breed and parent-of-origin effects in the bovine pangenome era

Authors:

Callum MacPhillamy[1], Tong Chen[1], Stefan Hiendleder[1,2], John L. Williams[1,3], Hamid Alinejad-Rokny[4], Wai Yee Low[1]

[1]The Davies Research Centre, School of Animal and Veterinary Sciences, University of Adelaide, Roseworthy, SA 5371, Australia

[2]Robinson Research Institute, The University of Adelaide, North Adelaide, SA 5006, Australia

[3]Department of Animal Science, Food and Nutrition, Università Cattolica del Sacro Cuore, 29122 Piacenza, Italy

[4]BioMedical Machine Learning Lab, The Graduate School of Biomedical Engineering, UNSW, Sydney, NSW 2052, Australia

Abstract

Background

Most DNA methylation studies have used a single reference genome with little attention paid to the bias introduced due to the reference chosen. Reference genome artefacts and genetic variation, including single nucleotide polymorphism (SNPs) and structural variants (SVs), can lead to differences in methylation sites (CpGs) between individuals of the same species. We analysed whole genome bisulfite sequencing (WGBS) data from the fetal liver of Angus (*Bos taurus taurus*), Brahman (*Bos taurus indicus*) and reciprocally crossed samples. Using reference genomes for each breed from the Bovine Pangenome Consortium, we investigated the influence of reference genome choice on the breed- and parent-of-origin effects in methylome analyses.

Results

Our findings revealed that ~75% of CpG sites were shared between Angus and Brahman, ~5% were breed-specific, and ~20% were unresolved. We demonstrated up to ~2% quantification bias in global methylation when an incorrect reference genome was used. Furthermore, we found that SNPs impacted CpGs 13 times more than other autosomal sites (p-value < 5×10^{-324}) and SVs contained 1.18 times (p-value < 5×10^{-324}) more CpGs than non-SVs. We found a poor overlap between differentially methylated regions (DMRs) and differentially expressed genes (DEGs) and suggest that DMRs may be impacting enhancers that target these DEGs. DMRs overlapped with imprinted genes, of which one, *DGAT1,* which is important for fat metabolism and weight gain, was found in the breed-specific and sire-of-origin comparisons.

Conclusions

This work demonstrates the need to consider reference genome effects to explore genetic and epigenetic differences accurately and identify DMRs involved in controlling certain genes.

Keywords

Bisulfite sequencing, methylation, CpG, structural variants, *Dgat1*, differentially methylated region, bovine pangenome

Background

DNA methylation is a key epigenetic modification that plays a vital role in regulating gene expression, repression of transposable elements, and parental chromosome specific regulation through genomic imprinting and X-chromosome inactivation [1, 2]. In mammals, DNA methylation primarily occurs at C-phosphate-G dinucleotides (CpGs) [3, 4]**.** DNA methylation influences gene expression either by recruiting proteins involved in gene repression or by blocking transcription factor binding sites (TFBSs) within promoter regions [5]. Hypomethylation of a promoter has been associated with the increased expression of the corresponding gene [6]. However, recent work has shown that promoter hypermethylation can also lead to gene expression [7]. The relationship between DNA methylation and gene expression is complicated by the role of enhancer methylation in regulating gene expression [8, 9]. In the presence of high DNA methylation, enhancers have been observed to be associated with high levels of the histone modification H3K27ac [10], which is often associated with active gene transcription [11-14].

Most DNA methylation studies have used a single reference genome with little or no knowledge of the impact of reference genome choice on the interpretation of methylome differences. The choice of reference genome has been shown to have an impact on DNA methylation analyses, with up to a nine per cent bias reported when the incorrect reference is used (Wulfridge, Langmead [15]. Using a single reference genome has been shown to bias read mapping in favour of reads with high similarity to the reference [16-20]. This bias occurs because reads containing non-reference alleles or regions that are divergent from the reference either align poorly, align to the wrong genomic region, or fail to align. This reference bias has been shown to affect analyses of cattle breeds [21, 22], humans [17, 23], and sheep [18].

The majority of mammalian methylation occurs in the CpG context. Consequently, a single nucleotide polymorphism (SNP) can remove a methylation site, thus introducing a reference bias if the individuals being studied do not possess the same SNPs as the individual used to generate the reference. In addition to SNPs, structural variations (SVs) among individuals may remove or introduce CpG sites. The disparity between CpG sites can confound analyses by identifying a methylated CpG in one individual when another individual has no CpG at that position. As a result, SNPs and SVs can both introduce bias, as reads may be unambiguously assigned in duplicated regions not found in the reference and mismatches in reads can result in the loss of some reads. Moreover, if individuals have insertion SVs that carry CpG sites, reads that originate from the insertion/deletion (indel) regions can only be mapped if the complete sequence data for the population is available. We consider SNPs and SVs that alter CpGs as genetic changes with potential effects on epigenetic regulation. We use the term ‘genetics of epigenetics’ to describe this phenomenon.

As more genomes for a given species become available, the research community is gradually shifting toward using pangenomes to account for genetic variation within a population more accurately. A pangenome is a collection of the genomes of multiple individuals, representing all genetic variation within that population and is thus a more accurate way to represent genetic diversity than a single reference genome [24]. Current pangenome projects include human [24, 25], cattle [26], and maize [27]. As genetic differences within a population can result in CpG differences, these pangenomes provide a valuable resource to study DNA methylation changes between diverse groups of individuals of the same species.

The two main lineages of modern cattle breeds are generally accepted to have been derived from two separate domestication events of the wild auroch (*Bos primigenius*) [28]. The first domestication event occurred in the Fertile Crescent around 10,000 years ago and gave rise to *Bos taurus taurus* from the wild auroch, *B. p. primigenius* [29-31]. A second domestication event occurred in the Indus Valley, ~1,500 years later, from *B. p. nomadicus*, which separated from *B. p. primigenius* around 250-330,000 years ago [32] and gave rise to *Bos taurus indicus*. The subspecies are referred to here as taurine and indicine cattle, respectively [28], where the Angus breed represents taurine cattle, and Brahman is representative of indicine cattle. Angus and Brahman have contrasting phenotypes, e.g., Angus have been bred for meat production traits [33], whereas Brahman have superior heat and disease tolerance traits [34, 35]. DNA methylation differences may partly be responsible for the phenotypic differences between these two breeds.

As expected from their domestication history, Angus and Brahman cattle represent genetically highly diverged subspecies [36, 37]. However, as they produce fertile offspring when mated [38], they are an appropriate model to investigate the impact of using a single reference genome on methylome analysis of two genetically diverse populations. We have previously produced high-quality haplotype-resolved reference genomes for Angus and Brahman [39], which are genomes included in the Bovine Pangenome Consortium project [26] and are used in the present study.

Breed-specific differences in CpGs may occur due to a SNP, such as those caused by spontaneous deamination [40, 41], or may result from SVs. A single SNP affecting a CpG site has been shown to drastically alter the methylation state of the *IGF2* gene in pigs, leading to changes in muscle development [42]. SVs have been associated with decreased methylation in cancers [43] and with changes in the methylation of the kappa opioid receptor (*KOR)* promoter associated with KOR dysfunction and schizophrenia [44].

Parent-of-origin effects (POEs) occur when only one allele is expressed, and the phenotype in the offspring may depend on which parent contributed the expressed allele [45]. Reciprocal crossing is necessary to elucidate how each parent contributes to a particular phenotype. POEs have been observed in hybrids of mice [46], cattle [47] and pigs [48], and there is increasing evidence that fetal development is influenced by POEs [49-53]. Given the similarity in gestation period between cattle and human and the single fetus with similar development trajectory, cattle are an attractive model species to study human reproductive and developmental biology [54-57].

To investigate the potential impact of reference genome choice on methylome analyses and to improve our understanding of the genetic and epigenetic factors driving the phenotypic differences between cattle subspecies, we used WGBS data from 24 fetal liver samples of purebred Brahman and Angus cattle and their reciprocal-crosses to perform a comprehensive assessment of the impact of reference genome choice on differential methylation and gene expression. This study serves as an example of how to investigate epigenetic differences between breeds, strains, and populations within species and informs about reference genome effects on the interpretation of methylome analyses.

Results

Mapping of WGBS data and calling CpG

Each of the 24 samples representing the four genetic groups (Fig 1A; S. table 1) was sequenced for WGBS analysis to at least 30X coverage and then mapped separately to the Brahman and Angus genomes (Fig 1B). An average mapping rate of ~95% was achieved when reads were mapped from each sample to the Brahman reference genome and ~93% when mapping to the Angus reference genome (Table 1; S. table 1). All samples had at least 10X coverage for 93% of the Brahman sequence. Using the Angus reference, all samples had 10X coverage for at least 90% of the sequence (Table 1; S. table 1).

We performed all analyses twice for each reference genome, first using all CpGs with ≥10X in each reference genome and again where we retained only CpG sites with ≥10X that we could confidently assign as being shared between both breeds. Between 85% and 88% of autosomal CpG sites had coverage ≥ 10X when considering all CpG sites on both the Brahman and Angus reference and shared CpG sites (Table 1; S. table 2). Median coverage of CpG sites across all samples ranged from 25-34X regardless of reference and CpG sites considered (i.e., shared or all) (Table 1; S. table 3).

Table 1. Mapping statistics of Angus and Brahman reference genomes.

	Angus	Brahman
Mapped reads*	1,455,481,398 (99%)	1,457,794,807 (99%)
Duplication rate (%)*	10	14
CpGs with ≥ 10X coverage in all samples	22,116,287 (86%)	21,962,589 (85%)
CpG coverage*	30	30

* Mean of all samples.

Clustering of genetic groups

Comparing the methylation patterns between the genetic groups, we found that samples within a genetic group were more similar to each other than with samples from other groups. For example, samples from the BTBT group had higher correlations with other BTBT samples than BIBI samples. BTBT had the highest within-group Pearson correlations (r between 0.81 and 0.88) (S. figure 1). The samples that were least correlated with one another were those belonging to BTBT and BIBI, with correlations between 0.75 and 0.78. Samples from the reciprocal cross groups (BIBT; BTBI) had similar correlations with other samples within their own group (r between 0.81 and 0.83) as well as with samples from the alternative reciprocal cross (r between 0.80 and 0.83). Overall, correlations were high within each genetic group ($r \geq 0.8$) (S. figure 1).

We performed a principal component analysis of the 24 samples using CpG sites covered by at least 10 reads in all samples (Fig 2A). BTBT and BIBI formed distinct clusters distant from one another, with the two hybrid genetic groups clustering much closer together and between the two parental genetic groups. Nevertheless, the hybrid groups were clearly separated on the PCA plot (Fig 2A). The separation of groups we observed from the methylation data was similar to that seen for the gene expression data (Fig 2B), indicating a potential parent-of-origin effect.

Overview of DNA methylation patterns

Samples had global mean CpG methylation of between 47-62%, with most samples ranging from 49-54% (S. table 4). Mean exon CpG methylation was 45-54% for all samples, with most samples ranging from 48-54% methylation (S. table 5). The 5’ UTRs and promoter regions had the lowest mean CpG methylation percentage across all samples, between 10-13% and 24-31%, respectively (S. table 5). The intergenic regions displayed mean methylation levels that ranged from 47 – 65% (S. table 5), with most samples ranging from 49-57%, similar to the global mean. The introns exhibited slightly higher methylation levels, with means ranging from 50-64% (S. table 5), and most samples in the 53-59% range. The 3’ UTRs revealed the highest overall CpG methylation levels, 57-69% (S. table 5). Lastly, the predicted enhancers, according to MacPhillamy, Alinejad-Rokny [58], exhibited CpG methylation levels ranging from 42-53%, with most samples within 43-48% methylated (S. table 5). Similar methylation patterns were observed using only shared CpGs in exons, 5’UTRs, intergenic, introns, promoters, predicted enhancers, and 3’UTRs, regardless of the reference genome used.

Shared and breed-specific CpGs

We were able to confidently identify 74-75% of CpGs in the Brahman and Angus genomes that were shared between the two breeds (Table 2; S. table 6). We found that around 4% of CpG alignments contained a SNP between reference genomes (S. table 6; S. figure 2), i.e., were breed-specific. About 22% of CpGs could not be confidently assigned as shared or breed-specific and so were not considered in the shared CpG analysis. By definition, breed-specific regions with CpG sites did not align when the other breed genome was used as the reference. We found ~1% of such CpG sites. In total, the SNP change and breed-specific categories of CpG sites constituted 4.7% and 4.9% of CpGs between the Angus and Brahman reference genomes, respectively, and were considered breed-specific. It should be noted that in this study, we consider breed-specific as CpGs that appear in one reference genome and not the other. The limitation of this being neither reference genome likely captures all variation present within the two breeds.

Table 2. Number of CpGs in the Angus and Brahman reference genomes.

	Angus	Brahman
Total CpGs[A]	25,712,300	25,799,151
CpGs aligned to other reference[B]	25,209,966	25,228,509
CpGs shared in other genome[C]	18,813,726	18,781,688
CpGs affected by SNP[D]	993,318	1,003,167
Unresolved CpGs[E]	5,402,922	5,443,654

[A] Total number of CpGs present within the genome.
[B] Number of CpGs that could be aligned from one genome to the other using Minimap2 [59].
[C] Number of CpGs in B that were CpGs in both species.
[D] Number of CpGs in B that were a CpG in one species but are no longer CpGs in the other.
[E] Number of CpGs in B that could not be confidently assigned as either shared or a SNP.

Enrichment of SNPs affecting CpG sites

Using the autosomal SNPs identified by Minimap2 and PAFtools.js [59], we observed that Brahman and Angus autosomal sequences differ by an average of ~0.4% (S. table 7). SNPs in CpG sites were enriched by ~13 times compared to the genomic background (Chi-square test of independence, adjusted p-value < 5×10^{-324}), which means SNPs between these two breeds affect CpG sites disproportionately more than other autosomal sites.

Looking more closely at the CpG SNP changes, we found that most (~81%) of the CpG SNP were either C to T or G to A changes (S. figure 2), which is very similar to the number of CpG SNP changes detected in humans (80.7%) [60]. The remaining SNP changes combined comprised ~19% of the total observed mutations at CpG sites (S. figure 2; S. table 6).

Increased number of CpGs within structural variants

Next, we tested whether CpGs were enriched in SVs compared to the rest of the genome. Using the Brahman genome as the reference, we observed 16,011 SVs between Brahman and Angus, making up ~15Mb of sequence. We observed 1.18-fold more CpGs within SVs than in non-SVs (Mann Whitney U-test, p-value = 8.91×10^{-5}). When considering CpGs affected or introduced by SNPs and SVs, the CpG mutation rate is approximately 6.7% between Brahman and Angus compared to the genome-wide mutation rate of around 1%.

Choice of reference genome influences methylome results

We examined the CpG methylation differences between each sample when mapped to Angus and Brahman reference genomes and observed a statistically significant difference in CpG methylation levels in all samples when using all CpGs with ≥10X coverage (two-sample Kolmogorov-Smirnov test, adjusted p-value < 0.05) (Table 3; S. table 8). When only considering the shared CpGs, we observed a significant quantification bias in all samples, though it was much smaller (two-sample Kolmogorov-Smirnov test, adjusted p-value < 0.05) (Table 3; S. table 8). When we pooled all CpG methylation values for a given group and compared the Brahman and Angus references, we observed a statistically significant quantification bias in all four groups (two-sample Kolmogorov-Smirnov test, adjusted p-value < 0.05) (Figure 3A; Table 3; S. figure 3A; S. table 8). When comparing only the shared CpGs, we observed a much weaker quantification bias between genomes, though it was still significant at the group level (two-sample Kolmogorov-Smirnov test, adjusted p-value < 0.05) (Figure 3B; Table 3; S. figure 3B; S. table 8). When comparing global CpG methylation differences between samples mapped to Brahman versus those mapped to Angus, the largest quantification bias was ~2% for BTBT. The other quantification biases were ~0.8%, ~0.7% and ~0.3% for BTBI, BIBT and BIBI samples, respectively (Figure 3A; Table 3; S. figure 3A; S. table 8). When using only shared CpGs, the quantification bias was reduced to ~0.6%, ~0.5%, ~0.4%, and ~0.2% in BTBT, BTBI, BIBT and BIBI, respectively (Figure 3B; Table 3; S. figure 3B; S. table 8).

Next, we examined whether the shared CpGs exhibited correlated methylation levels regardless of the reference genome used. For example, we wanted to determine whether a given CpG in the Brahman reference has the same methylation percentage as the corresponding CpG in the Angus reference for a given sample. We observed RMSE values of around 0.01 for groups (S. figure 4), with most CpGs exhibiting less than 10% difference in methylation between the reference genomes. Interestingly, we observed several CpGs that appeared sensitive to reference genome choice. For example, 264,023 CpGs had an absolute methylation difference of least 10%, with 429 CpGs having an absolute difference of at least 50% depending on the reference genome used (S. table 9).

To further investigate the influence of the reference genome on downstream analyses, we compared DMRs identified by the two reference genomes to evaluate if the direction of methylation changed, i.e., hypermethylated became hypomethylated and vice versa. DMRs from Angus were mapped to the Brahman reference and we found approximately 12% (28,922) of Angus DMRs overlapped with Brahman DMRs by at least 90% of their length. Of these DMRs that mapped to the Angus reference, 3,575 showed changes in methylation direction when mapped to the Brahman reference (S. table 10). That is, a DMR that was observed as hypomethylated in Angus samples relative to Brahman when mapped to the Angus reference was observed to be hypermethylated in Angus relative to Brahman when mapped to the Brahman reference. We observed similar numbers (3,581) when lifting DMRs from Brahman to Angus (S. table 10). There were no methylation direction changes when we considered differentially methylated cytosines (DMCs).

Lastly, we compared homologous regions between the Angus and Brahman reference genomes to determine whether any differentially methylated regions existed between these two genomes for each group (Methods). Using a window size of 100kb, we observed 41, 31, 30 and 36 windows being significantly differentially methylated when using BTBT, BTBI, BIBT and BIBI samples, respectively (paired Wilcoxon test, adjusted p-value < 0.05) (S. Table 11).

Breed-specific CpGs show distinct methylation patterns

Looking more closely at the breed-specific CpGs, we first determined the background methylation for Angus samples as the methylation of all shared CpG sites when mapped to the Angus reference. We then extracted the methylation for all Angus samples from CpG sites that were identified as breed-specific. We then compared the background methylation distribution against the Angus-specific distribution. This was repeated for the Brahman using the Brahman reference. Breed-specific CpG sites were significantly more likely to have lower methylation than the background methylation for Angus (Wilcoxon signed-rank test, $p < 5.0 \times 10^{-324}$) and Brahman (Wilcoxon signed rank-test, $p < 5.0 \times 10^{-324}$), respectively (Figure 3D-E).

DMRs between breeds show limited overlap with DEG promoters

As we observed a quantification bias when using all CpGs mapped against each reference genome, we restricted breed-specific and POE analyses to those CpGs identified as shared. Additionally, we examined the number of DMRs at the 25% and 50% difference thresholds, i.e., more stringent thresholds for calling DMRs which substantially reduced the numbers (S. table 12). Given that minor changes of less than 10-15% in methylation have been observed to influence gene expression and phenotype [61, 62], we used a difference threshold of 10% to interpret the results.

Using Brahman as the reference, we identified 123,602 DMRs and 1,549 DEGs (S. table 12; S. table 13). Of the 123,602 DMRs observed, ~19% (23,575) overlapped with the surrounding region of the significant DEGs, with around twice as many DMRs falling within the putative-enhancer region compared to the promoter region after normalising for the length difference between the enhancer regions and promoter regions (S. table 14). Only 68 DMRs overlapped with promoters of DEGs, despite 99% of significant DEGs being overlapped by a DMR. When the Angus reference was used, of the 125,544 DMRs identified, ~25% (31,252) of those overlapped with a DEG. Around 1.6 times as many DMRs fell into an enhancer region compared to DEG promoter regions (S. table 14), despite substantially more (1,872) DEGs observed (S. table 13) and 99% overlapping with a DMR.

We then examined the overlap of DMRs and imprinted genes, first using Brahman as the reference. Here, ~1% (1,182) of the DMRs identified between BIBI and BTBT overlapped 79 imprinted genes. Only one imprinted gene, Par-6 family cell polarity regulator gamma (*PARD6G*), did not overlap with any DMR. Most DMRs (~79% of the 1,182) that overlapped an imprinted gene fell into putative enhancer regions. Five imprinted genes were significantly differentially expressed when comparing BIBI and BTBT (Table 4). These genes were DS cell adhesion molecule (*DSCAM*), neuronatin (*NNAT*), Lin-28 homolog B (*LIN2B*) and protein phosphate 1 regulatory subunit 9A (*PPP1R9A*). *DSCAM* and *NNAT* had higher expression in BTBT, and the remaining three DEGs, *DGAT1*, *LIN2B* and *PPP1R9A*, had higher expression in BIBI. We again observed five imprinted DEGs using the Angus reference; however, one gene was a novel gene. The remaining genes (*NNAT*, *LIN28B*, *DGAT1* and *PP1R9A*) showed the same expression pattern as when mapped to Brahman (Table 5).

Dam-of-origin methylation shows less overlap with imprinted genes

To investigate the dam-of-origin effects (DOEs), we compared samples with Brahman dams (BIBI and BTBI) with those with Angus dams (BTBT and BIBT). Using the Brahman genome as the reference, 457 DEGs were identified in the DOE comparison. Around 6% (1,236) of the DMRs overlapped with DEGs, with ~78% of DEGs being overlapped by a DMR. Approximately 2.4 times as many DMRs fell into putative enhancer regions compared to DEG promoter regions (S. table 14). There were 52 imprinted genes that overlapped with ~1% (190) of the DMRs identified in the comparison. Of the 190 DMRs that overlapped with an imprinted gene, 128 DMRs overlapped with the putative enhancer region. Four imprinted genes were significantly differentially expressed (Table 4). Zinc finger CCCH-type containing 12C (*ZC3H12C*), *PPP1R9A* and *LIN28B* had higher expression in samples with Brahman mothers. Using Angus as the reference, we observed 1,254 (~5%) DMRs overlap with the 358 DEGs and ~88% of DEGs covered by a DMR. Around 2.30 fold more DMRs fell into putative enhancer regions compared to DEG promoter regions (S. table 14). Three imprinted DEGs (*ZC3H12C*, *PPP1R9A* and *LIN28B*) were identified using the Angus genome as the reference (Table 5).

Sire-of-origin methylation may be driving differential gene expression

To investigate the sire-of-origin effects (SOEs), we compared samples with Brahman sires (BIBI and BIBT) with those with Angus sires (BTBT and BTBI). Using the Brahman reference, we identified 62,056 DMRs and 1,190 DEGs in the sire group comparison using shared CpGs (S. table 12; S. table 13), with 2.4 times more DMRs overlapping a putative enhancer region than promoter region (S. table DMR_DEG). There were 63,516 DMRs identified using the Angus reference, but substantially more DEGs (1,568) were identified (S. table 13). Around 12% (7,840) of the DMRs overlap with a significant DEG; ~93% of DEGs were overlapped by a DMR. Around 19% (~12,251) of DMRs overlapped a DEG and ~96% of DEGs overlapped with a DMR; ~75% of the DMRs overlapped a putative enhancer region. This was approximately twice as many DMRs overlapped with a putative enhancer compared to a promoter when using the Angus reference.

We observed 73 imprinted genes that overlapped DMRs identified between the two different sire groups. Less than 1% (586) of DMRs overlapped the 73 imprinted genes, with most (~73% of 586) occurring in the putative enhancer region. Eight imprinted genes were significantly differentially expressed and overlapped with a DMR (Table 4). These genes were *DSCAM* 5-hydroxytryptamine receptor 2A (*HTR2A*), *NNAT*, *DGAT1*, necdin MAGE family member (*NDN*), and tissue factor pathway inhibitor 2 (*TFPI2*). *DSCAM*, *NNAT*, *NDN, MKRN3* and *TFPI2* had higher expression in samples with Angus sires, with the other genes (*HTR2A*, *DGAT1*, *SLC22A18*) being more highly expressed in samples with Brahman sires. *SLC22A18* showed high expression in the Brahman sire group, and *MKRN3* showed higher expression in the Angus sire group, but neither overlapped with any DMRs. Seven significantly differentially expressed imprinted genes were observed when using the Angus reference (Table 5). In this case, the seven significantly differentially expressed imprinted genes with DMR overlap were *DSCAM*, *HTR2A*, *NNAT*, *MKRN3*, *NDN*, and *SLC22A18*. *DSCAM*, *NNAT*, *MKRN3* and *NDN* had higher expression in samples with an Angus sire. The remaining three genes (*HTR2A*, *DGAT1* and *SLC22A18*) had higher expression in samples with a Brahman sire. *DGAT1* did not overlap any DMRs when using the Angus reference.

Discussion

In the present study, we observed genome-wide CpG methylation correlations among replicates that ranged from 75% to 82% between groups and from 81% to 87% within groups. These correlations were similar to a recent study in mice where genome-wide CpG methylation correlations among replicates ranged from 73% to greater than 80% [63]. Moreover, we observed levels of liver global CpG methylation between 47-62% in the present study, which is similar to previous studies of human [64], mouse [63] and cattle [65].

Mapping statistics can provide an insight into how the choice of reference genome will affect downstream analyses [66]. However, we observed negligible differences in raw mapping statistics regardless of whether the Angus or Brahman reference genomes were used. Additionally, the global methylation quantification bias observed was less than 2%, depending on the reference genome used. This quantification bias is lower than the 7-9% quantification bias found in the mouse genome, depending on the reference genome used (Wulfridge, Langmead [15]. The extent of this bias is influenced by the divergence between reference genomes and whether the breed-specific CpGs tend to be hypo- or hypermethylated. Brahman and Angus have a CpG divergence of ~4%, whereas the mouse genomes analysed by Wulfridge, Langmead [15] had a CpG divergence of 10.7%. The bias we observed was greatest in the BTBT samples when the Brahman genome was used as the reference, most likely because the Angus-specific CpG sites tended to be hypermethylated. Conversely, the quantification bias was lower in the other genetic groups, possibly due to the hypomethylation in Brahman-specific CpG sites.

Spontaneous deamination of methylated CpG to TpG is the most common dinucleotide mutation in the mammalian genome [40, 41]. We observed around 81% of SNPs between Brahman and Angus as being C-T or G-A mutations. A recent study observed 34,677 SNPs affecting CpG sites between indicine and taurine genomes [67]. The difference in the number of SNPs between the two studies is likely due to Capra, Lazzari [67] having used reduced representation bisulfite sequencing with substantially lower coverage than the present study and that they only considered SNPs that affected CpG sites. When differential methylation analysis is performed, a breed that has lost the C (mutated to T) will be reported as having 0% methylation at that site when, in fact, there is no CpG present. This incorrect identification of an unmethylated site can then severely impact the interpretation of results.

SVs have been associated with various traits in humans, including HIV-1 susceptibility [68], autism [69-71] and carcinogen metabolism [72]. In livestock, SVs have been implicated in diverse traits ranging from horn (polled) status [73, 74] to bulldog calf syndrome [75]. The SVs between Brahman and Angus have significantly more CpGs than the background genome, potentially introducing CpGs with important regulatory effects. However, due to their presence in only one subspecies, a single reference genome will fail to account for these breed-specific CpG sites. Therefore, the phenotypic differences between the two breeds may be influenced by CpGs that cannot be compared accurately with a single reference genome if they are in breed-specific regions.

We observed relatively few changes in methylation direction, and these were likely to be an artifact of how the genome was tiled and possible erroneous alignments in the coordinate conversion. A common step of some DMR callers is to perform window tiling of the genome to identify DMRs or to enable analysis when coverage is low [76-78]. SNPs and SVs can potentially complicate analyses when genome tiling is used to identify DMRs, as a single reference genome cannot account for these variants. Although we observed no directional changes when considering DMCs, it is possible that SNPs impacting CpGs in otherwise shared regions affected the quantification of DMRs. For example, MethylKit uses a genome tiling approach to identify DMRs and so any mutation that impacts a CpG site, particularly spontaneous deamination (C > T), is likely to erroneously identify a region as hypomethylated in the group that has the spontaneous deamination when in fact, no CpG exists in that group. As such, researchers should be careful when using genome tiling in methylation analyses that compare breeds, strains or populations. Moreover, we demonstrated several CpG sites that appear sensitive to reference genome choice, differing by more than 10% between the two genomes. Additionally, we demonstrated that multiple 100kb regions are differentially methylated between the genomes when using a single group of samples. It is likely that these CpG sites and variable regions would confound downstream analysis, especially if they overlap with regions of interest, such as cis-regulatory elements. Thus, this demonstrates that quantification bias can remain even when attempting to control for reference genome differences.

While DNA methylation analyses between breeds and strains are challenging, exciting solutions are on the horizon. Indeed, with long-read sequencing like Oxford Nanopore becoming more cost-effective and given it has the ability to capture DNA methylation with no additional sample preparation, it is much more likely that researchers will be able to follow the recommendations of Wulfridge, Langmead [15] and examine DNA methylation of individuals with personalised reference genomes. Long-read sequencing has the potential to simplify DNA methylation analyses of diverse populations substantially.

Most DMRs identified in this study did not overlap with DEGs. However, of the DEGs that overlapped with a DMR, there was a tendency for the overlap to occur more frequently in the putative enhancer region than in promoters or DEG bodies. This trend suggests that differential methylation of enhancers may impact gene expression differences in bovine fetal liver. Indeed, a growing body of evidence suggests that enhancer methylation is important in embryonic and fetal development [63, 79-81].

There were more significant DEGs when mapping to the Angus reference than the Brahman reference. Interestingly, genes that were DE using the Brahman reference were not always DE when using the Angus reference. The choice of reference genome has been shown to impact differential expression analysis in rice [82], bacteria [83] and human [84, 85] when using short-read RNA-seq. When the reference genome better represents the individuals being studied, more reads can be uniquely aligned to the correct position, providing a more accurate estimate of gene expression.

We identified several interesting DEGs associated with DMRs, particularly imprinted genes. Among these was *DGAT1,* which is involved in fat metabolism in milk production [86], feed conversion and adipogenesis [86, 87]. Several studies have investigated the role of *DGAT1* in weight gain [88, 89]; expression of *DGAT1* is necessary for weight gain, especially when the caloric density of food is high [89]. We found differential expression of *DGAT1*, with higher expression in Brahman than Angus (BIBI vs BTBT) and when Brahman was the sire (BIBI, BIBT vs BTBT, BTBI). Taken together, the comparison of the breed and sire of origin suggests that the breed of the sire may be an important determinant in the expression of this gene. Higher *DGAT1* expression may result from adaptation to poor feed quality; e.g. Elzo, Riley [90] observed better feed conversion efficiency in Brahman compared to Angus and Brahman x Angus cattle. Regulation of *DGAT1* expression may occur via DNA methylation, as there is a DMR ~42kb downstream of the transcription start site, which was identified in both the breed-specific and SOE comparisons.

Parent-of-origin DMRs may change how cis-regulatory elements interact with target genes and influence gene expression in the offspring [45, 91]. It has been observed that parent-specific methylation can alter the cis-regulatory landscape around certain genes, such as *IGF*2 [92, 93]. DMRs may influence the DEGs and, ultimately, help drive the differences in phenotype. However, to confidently assign gene expression and DMRs to a particular parent, long-read sequencing [94] is needed to identify variations that link the sequences to the parent of origin. Additionally, the use of reciprocal crosses will enable one to investigate if a combination of breed and sex of the parent impacts which transcript is expressed.

SNPs and SVs have been shown to complicate and bias analyses in several studies [15, 82-85]. In our analysis, we observed an enrichment of SNPs affecting CpGs between Brahman and Angus. Capra, Lazzari [67] also reported a higher frequency of breed-specific SNPs around DMCs in a study of indicine and taurine cattle. This finding suggests that genetic differences between the two breeds may contribute to epigenetic variations. Using individual animal genomes in the study to account for genetic variations, as Wulfridge, Langmead [15] suggested, would enhance the accuracy for each individual. However, despite decreasing sequencing costs, the cost will likely be prohibitive in most livestock contexts. A possible solution was explored in a recent study comparing methylation in taurine and indicine cattle [67]. Here, the authors used genotyping by sequencing to exclude SNPs affecting CpG sites from the analysis [67]. While this simplifies downstream analysis, it may also remove CpGs involved in the phenotypic differences between the two breeds, representing a limitation of the present study and that of Capra, Lazzari [67]. An alternative approach to using single reference genomes is the utilisation of pan-genomes, which encompass the majority of variations within the population [20, 95]**.** This feature is particularly important in the context of DNA methylation studies where, demonstrated in our study, SNPs at CpG sites can exert substantial effects on local methylation information.

Conclusions

This study generated a substantial WGBS dataset derived from two phenotypically diverse cattle breeds which are representative of the two cattle subspecies and highlighted the importance of reference genome choice in methylation analyses. Our findings suggest that the DMRs may primarily exert their influence on enhancer elements rather than promoters. We also identified 11 genes that might be under DMR control. The results underscore the advantages of using the appropriate reference genome for the data set and provide additional evidence supporting the incorporation of genome graphs to improve analyses of populations with high genetic divergence.

Methods

Study Animals and Sample Collection

All animal experiments and procedures described in this study complied with Australian guidelines, approved by the University of Adelaide Animal Ethics Committee and followed the ARRIVE Guidelines (https://arriveguidelines.org/) (Approval No. S-094-2005). Liver tissue samples from concepti were the same as those described in Liu, Tearle [96]. Briefly, the parents were purebred Angus (*B. t. taurus*) and purebred Brahman (*B. t. indicus*), herein denoted as BT and BI. Fetuses were sired by three BTBT bulls and 2 BIBI bulls. Primiparous females and their fetuses were ethically sacrificed at day 153±1 of gestation. Concepti were dissected, and tissue samples snap-frozen in liquid nitrogen and stored at -80°C until further use. Liver samples from three female and three male individuals from each of the four genetic combinations: BT x BT, BT x BI, BI x BT, and BI x BI were used.

DNA extraction and sequencing

DNA was extracted from frozen fetal liver tissues using Qiagen® Dneasy® Blood & Tissue Kit following the manufacturer's instruction and sent to BGI Hong Kong, China, for WGBS library preparation and sequencing. Bisulfite conversion was performed using the Zymo Research™ EZ DNA Methylation™ - Gold Kit (D5005). All samples were sequenced in a single batch, and each sample was sequenced to ~30X coverage using the BGI DNB-seq.

RNA was extracted from frozen fetal liver tissues using Illumina® RiboZero Gold kits following the manufacturer's instruction and prepared for Illumina RNA-seq short-read sequencing. The RNA-seq protocol and data availability (GEO accession number: GSE148909) have been described in our previous work [96].

The same tissue samples were used in both the RNA-seq and WGBS. Individual sample names and their corresponding genetic group are given in Supplementary Table 11.

WGBS mapping

WGBS reads were mapped using the MethylSeq Nextflow pipeline (v. 1.6.1) [97] with the '—zymo' trimming parameter. The reads were first checked for quality with FastQC (v. 0.11.9) (https://www.bioinformatics.babraham.ac.uk/projects/fastqc/), then adapters were trimmed using Trim Galore (v. 0.6.6) (https://github.com/FelixKrueger/TrimGalore), and the reads were reassessed for quality post-trimming. Trimmed reads passing qvalue ≥ 20 were mapped to the Brahman (GCA_003369695.2) and

Angus (GCA_003369685.2) genomes [39] using BWA-Meth (0.2.2) (arXiv:1401.1129). The non-pseudo autosomal region of the Angus Y chromosome was added to the Brahman reference. This step enabled us to include the Y chromosome sequence whilst avoiding duplication of the pseudoautosomal region. Both Brahman and Angus chromosome sequences were reorientated to match the orientation of ARS-UCD1.2 chromosomes [98]. After sorting the alignment files with SAMtools (v. 1.11) [99], duplicates were marked with Picard (v. 2.25.4) (https://broadinstitute.github.io/picard/). Bam file quality control was performed with the bamqc function from qualimap (v. 2.2.2d) [100] by setting the '-gd' parameter to HUMAN. Methylation calls were extracted using MethylDackel (v. 0.5.2) (https://github.com/dpryan79/MethylDackel) extract with the parameter '—minDepth 10' and output in MethylKit [76] format ('—methylKit') and a more generic cytosine report ('—cytosine_report'). In-house scripts were used to convert the MethylDackel output for use with DNMTools https://dnmtools.readthedocs.io/en/latest/ (see https://github.com/DaviesCentreInformatics/Brahman_Angus_WGBS). All samples had a bisulfite conversion efficiency of >99%. All downstream analyses only used CpGs from autosomes with ≥ 10X coverage.

Identification of shared and breed-specific CpG sites

For a given autosome, we extracted 1000bp around all CpGs that were not in the first 500bp or last 500bp of the chromosome; this yielded sequences that were 1002bp long. We then mapped the 1002bp CpG sequences from one subspecies to the reference of the other. We used minimap2 (v. 2.24) [59] with the ‘map-hifi’ preset to align CpGs from a given chromosome in one breed to the same chromosome in the other breed; alignments were sorted using SAMtools (v 1.11) [99]. Once the long sequences were aligned, we filtered the BAM file and considered all alignments where at least 900 bp were successfully aligned to the reference. We then used the Align package from BioPython (v 1.80) [101] to perform a local alignment between the 102bp sequences taken from the midpoint of the query and reference. We then recorded which CpG sites were shared between Brahman and Angus, which CpG sites differed, and which could not be aligned during the initial minimap2 alignment step (S. table 6). We performed subsequent analyses using all CpGs present on the autosomes for each reference and again using only the shared CpGs that passed the $\geq$ 10X coverage criteria. The CpG sites that could not be aligned in the initial alignment step with minimap2 (i.e. a genomic region that is present in one breed but missing in the other breed) or constituted a SNP were considered breed-specific CpG sites. All steps described in this section were performed for both Brahman and Angus reference genomes.

Identification of SNPs and SVs between genomes

To determine the accuracy of minimap2 alignments in identifying SNPs between the Brahman and Angus genomes, we first introduced artificial mutations into each genome using SNP Mutator [102]. The Angus and Brahman genomes have previously been reported to differ by ~1% [39]. Therefore, to determine how well minimap2 alignments can be used to detect SNPs between sequences that are divergent by ~1%, we first simulated mutations in each autosome for both species. For example, chromosome 1 in the Angus reference genome is 157,005,132 bp long, so we set the number of substitutions to 1,570,051 SNPs. In addition, the random seed was set to 12, and the number of times each autosome was mutated was set to 1. We repeated this for all autosomes in the Brahman and Angus genomes, adjusting the number of SNPs to maintain the 1% divergence in each autosome. We then mapped the mutated autosomal sequence to the original sequence for each autosome and breed, giving us 58 “replicates”. Alignments from minimap2 [59] were used as input to PAFtools which was used to identify variants between the original and mutated sequences. The minimap2 mapping parameters used were ‘-x asm10, -c, --cs’, followed by PAFtools ‘call --f’, where the file provided to the --f argument was the original, unmutated autosomal sequence. Minimap2 and PAFtools showed a mean accuracy of ~99% across the 58 autosomes, suggesting a good ability to identify SNPs between the two breeds (S. table 15). We then aligned each autosome from Brahman to each autosome from Angus, using minimap2 with the parameters ‘-cx asm10’ and ‘—cs’. The output from minimap2 was then used as input to paftools.js call with the parameter ‘-f <reference_autosome.fa>’, where reference_autosome.fa refers to the autosome that was supplied first to minimap2, i.e. the reference sequence, not the query. We then used the output VCF files to determine the SNP and SVs between the two genomes.

SNP and SV Enrichment

To determine whether CpGs were significantly impacted by SNPs, we identified all SNPs between Brahman and Angus that impacted a CpG site. As Brahman autosomes were used as the reference to minimap2, we used the coordinates of all CpGs within the Brahman genome to identify which SNPs in the VCF file had altered a CpG site. We constructed 2×2 contingency tables to perform a Chi-square test of independence per chromosome to determine whether CpGs had a higher likelihood of being affected by a SNP than non-CpGs. The four categories where 'CpG affected by SNP', 'non-CpG affected by SNP', 'unaffected CpG' and 'unaffected non-CpG'. We then adjusted the p-values to correct for multiple testing using the Benjamini-Hochberg correction procedure.

To assess whether CpGs were significantly enriched within SVs identified between the two genomes, we counted the number of CpG dinucleotides occurring within SV sequences and compared that against the number of CpGs that occurred in non-SV regions. To determine the probability of a CpG occurring outside an SV, we first identified the SV coordinates from the VCF file produced by PAFtools and constructed a bed file of SVs for each reference genome. Next, we used BEDTools [103] to generate coordinates of the complementary regions, i.e., the non-SV regions of each genome. We then extracted the fasta sequence of these regions for each genome. We then counted the number of CpGs that occurred in these regions on each chromosome and divided them by the combined length of all SVs on the given chromosome to determine the proportion of CpGs occurring in the SV regions; this value was added to a vector. We repeated this for CpGs occurring in non-SV regions, adding the proportions to a difference vector. With these two vectors we then performed a Mann-Whitney U test to determine whether SVs were more likely to introduce CpGs than non-SV regions.

Determining quantification bias between genomes

To determine whether there was a significant quantification bias between the Brahman and Angus reference genomes for a given sample, we compared the vector of all CpG sites with at least 10X coverage when mapped to Angus in sample *I* against the vector of all CpG sites with at least 10X coverage when mapped to Brahman. To ensure the vectors were equal, we randomly subset the larger vector to be the same length as the smaller, i.e., if the Brahman reference genome had more CpG sites with 10X coverage for that sample, the Brahman vector was randomly subset to match the number of CpG sites in the Angus vector for that sample. We repeated this for all samples using all CpGs and again with just the CpG sites marked as shared (Table 3; S. table 8). We then pooled all CpG sites for each sample within a group, e.g. all samples from BTBT, and determined whether the CpG methylation differed significantly for that group when mapped to Angus and Brahman. The p-value was determined using a Wilcoxon Rank Sum test and adjusted for multiple testing using the Benjamini-Hochberg procedure. We identified variable CpGs by matching the shared CpGs between reference genomes and then identifying those with an absolute methylation difference greater than 10%.

To further assess the presence of a quantification bias, we generated non-overlapping windows of both Angus and Brahman reference genomes with a length of 100 kb. We then aligned these 100 kb sequences to the opposite reference genome with `minimap2 –t 8 –c –x map-ont`. We then used the output PAF files to identify homolgous 100 kb windows from each genome. We extracted the methylation values from each window for a given group when mapped to each genome. For example, consider homologous-region “A” (HR-A), identified between the Angus and Brahman reference genomes. To identify whether this region exhibits differential methylation between reference genomes and thus exhibits quantification bias, we quantified all methylation values that fell within the Angus coordinates of HR-A using the methylation values for BTBT samples mapped to the Angus reference. We then quantified the methylation values that fell within the Brahman coordinates of HR-A using the methylation values for BTBT samples mapped to the Brahman reference. This gave two vectors of methylation values, which were then used as input to a Wilcoxon signed-rank test to determine whether the methylation values in this region were significantly different between genomes. The Benjamini-Hochberg false discovery procedure was used to adjust for multiple testing. This was repeated for each of the four groups.

Identification of differentially methylated regions

The methylKit package (v. 1.22.0) [76] was used to identify DMRs between breed and POE groups. We investigated breed effects by comparing BIBI samples with BTBT samples, maternal effects by comparing samples with BIBI dams (BIBI; BTBI) and those with BTBT dams (BTBT; BIBT) and paternal effects by comparing samples with BIBI sires (BIBI; BIBT) to those with BTBT sires (BTBT; BTBI) (S. table 16). The reference group was always the breed that matched the reference genome. For example, when BIBI and BTBT WGBS reads were aligned to the Brahman reference genome, BIBI samples were treated as the control group and BTBT as the treatment group.

We followed the pipeline described by the methylKit authors for DMR analysis [76]. Briefly, we only considered CpGs that were identified as shared. We then removed all CpG sites with less than 10X coverage and more than the 99.9^{th} percentile of coverage. Reads with too high coverage (e.g. from PCR duplication bias) can impair the accurate determination of the methylation percentage at that site and is a recommended pre-processing step for methylKit [76]. We then normalized the coverage using the default methylKit normalization strategy. We merged the CpG counts per group using the ‘unite’ function with ‘destrand = T’ and ‘min.per.group = 5L’ so that a given CpG site had to be covered by at least ten reads in five out of six samples per group. For the parent of origin DMR analyses, we set ‘min.per.group = 10L’.

We then identified differentially methylated cytosines between groups using the ‘calculateDiffMeth’ function, with sex as a covariate in the model. To determine differentially methylated regions, we used the ‘tileMethylCounts’ function with default parameters to divide the genome into regions for differential methylation analysis. This step allowed methylKit to divide the genome into non-overlapping regions based on the tiling windows. MethylKit then models the methylation at a given cytosine or region by fitting a logistic regression:

$$\log\left(\frac{P_i}{1-P_i}\right) = \beta_0 + \beta_1 * T_i + \mathrm{a}_{\mathrm{sex}} * \mathrm{Sex}_i$$

P_i denotes the methylation proportion for sample i in samples 1,…,*n*, where *n* is the number of samples across both groups in the comparison [76]. T_i represents the groups (0 for control, 1 for treatment). β_0 denotes the log odds of the control group (fraction of reads reporting C / 1 – the fraction of reads reporting C). β_1 denotes the log odds ratio between the control and treatment. α_{sex} denotes the parameter for the sex covariate and Sex_i denotes the sex (0 = male; 1 = female) for sample *i*. For further details, refer to Akalin, Kormaksson [76]. This design resulted in six different logistic models being fit: model 1A (breed comparison when aligned to the Angus reference), model 1B (breed comparison when aligned to the Brahman reference), model 2A (dam of origin comparison when aligned to the Angus reference) model 2B (dam of origin when aligned to the Brahman reference), model 3A (sire of origin when aligned to the Angus reference) and model 3B (sire of origin when aligned to the Brahman reference) (S. table 16). Any DMRs identified were either hypo- or hypermethylated with respect to the control group. We retained all DMRs with a difference in methylation of ≥ 10% and a q-value of ≤ 0.01 for further analysis. Q-values were obtained using the sliding linear model (SLIM) method [104]. We repeated this pipeline to identify DMCs, with the exclusion of the tiling step. An overview of the samples, reference genomes, types of CpGs and DMR analysis is given in Figure 1A-G.

DMR coordinate conversion

To determine if a given DMR changed methylation direction between genomes, we had to convert the coordinates of DMRs identified by alignment with the Angus genome to Brahman coordinates and vice versa. We considered a DMR as changing methylation direction if, for example, it is hypomethylated in BIBI samples compared to BTBT samples when using the Brahman reference but becomes hypermethylated in BIBI using the Angus reference genome. To investigate this, we first converted the DMR bed files to GTF files and then used Liftoff (v.1.6.2) [105] to transfer coordinates from one reference genome to the other. We then identified DMRs reciprocally overlapping one another by at least 90% between the two genomes, with these DMRs being considered successfully lifted over. DMRs that did not overlap by 90% were not considered for the methylation direction change analysis.

RNA-seq mapping and pre-processing

RNA-seq reads were mapped to the Brahman and Angus genomes as in the WGBS mapping step. Briefly, reads were checked for quality using FastQC (v. 0.11.4) (https://www.bioinformatics.babraham.ac.uk/projects/fastqc/) before being trimmed with Trim Galore (v. 0.4.2) (https://github.com/FelixKrueger/TrimGalore) with the parameters '--quality 10' and '--length 100'. Reads were mapped using HiSAT2 (v. 2.1.0) to both the Brahman and Angus reference genomes [39]; alignment files were sorted using SAMtools (v. 1.10) [99]. FeatureCount from the Rsubread package (v. 2.10.5) [106] was used to count how many reads mapped to genes.

Differential gene expression

Differential gene expression analysis was performed using an in-house R script with the DESeq2 (v. 1.40.2) [107] R package. The genome annotation was based on Ensembl v.104 for Brahman and Angus. The orientation of the genes was reversed where necessary to correspond with the orientation of the chromosomes of ARS-UCD1.2. In the breed comparison, where the number of samples in each group was six, we retained genes that had a count ≥ 10 in at least three samples. In the POE comparisons, we retained genes that had a count ≥ 10 in at least six samples. The group-mean parameterisation method was used to identify differentially expressed genes between groups with the following model design: '~0 + Genetics + Sex + Batch' [96]. DESeq2 then estimated size factors, dispersion and finally fits a negative binomial generalised linear model to identify DEGs. We then compared differential gene expression between purebred Angus and Brahman, Angus dams and Brahman dams, and Angus sires and Brahman sires. Genes with significant differences in gene expression at an adjusted p-value ≤ 0.05 were retained for further analysis. Furthermore, we performed canonical correlation analysis (CCA) to estimate the canonical correlation between the gene expression and methylation data. The first five canonical correlations were >0.7 with p-values < 0.05 (S. Table17).

Identifying imprinted genes

We downloaded a list of genes with evidence of imprinting in human, mouse and cattle from Morison, Ramsay [108] and https://www.geneimprint.org. We then used OrthoFinder to identify human orthologs of both Brahman and Angus genes [109], allowing us to assign Human Genome Organisation Gene Nomenclature Committee (HGNC) symbols to genes in each breed. To do this, we first identified which Brahman proteins had orthologs in human. We then identified the genes that encoded these proteins and used this information to assign human and Brahman genes as orthologs. We repeated the process for the Angus genes. We then identified all genes that could be assigned an HGNC symbol from the Brahman Ensembl annotation version 104 that were also present in the imprinted gene list (S. table 18). This filtering gave us 80 imprinted genes for Brahman autosomes. We repeated the process for Angus using the Angus Ensembl annotation version 104 and identified 79 imprinted genes. The discrepancy is due to one imprinted gene for Angus occurring on an unplaced scaffold.

Linking DMRs to DEGs

For each DEG, we considered five different regions in and around the gene where DMRs might have an influence. These regions included putative enhancer regions, 5kb outside the gene body, and the gene body itself (S. figure 3). The upstream putative enhancer region started 130kb upstream of the gene and then stopped 5kb upstream of the gene body for a total length of 125kb. We repeated this for the downstream putative enhancer region, starting 5kb downstream of the gene body and extending out 125kb. This number was based on the median distance between enhancers and their gene targets [110]. The 5kb region was from upstream of the start of the gene body to the start of the gene body. Again, this was repeated for the downstream 5kb region. The gene body was the region annotated as "gene" in the Ensembl annotation file. We then found all DMRs that overlapped these regions by at least 90% of their length using 'bedtools intersect' with the '-f' and '-F' arguments, both set at 0.9 and the '-e' argument set to True.

Declarations

Ethics approval

All animal experiments and procedures described in this study complied with Australian guidelines, approved by the University of Adelaide Animal Ethics Committee and followed the ARRIVE Guidelines (https://arriveguidelines.org/) (Approval No. S-094-2005).

Consent for publication

Not applicable

Availability of data and materials

The datasets generated and analysed during the current study are available in the NCBI SRA repository under BioProject: PRJNA626458. Code used to analyse the data is available at: https://github.com/DaviesCentreInformatics/Brahman_Angus_WGBS.

Competing interests

The authors declare that they have no competing interests.

Funding

The study was funded by the JS Davies bequest through the Davies Livestock Research Centre.

Authors' contributions

WYL, JLW and SH conceived and managed the project. SH designed and obtained *Bos taurus* and *Bos indicus* fetal resources. TC extracted WGBS samples and performed QC. CM performed all analyses; CM, SH, WYL and HAR interpreted data. CM and WYL drafted the manuscript, and all authors read, edited and approved the final manuscript.

Acknowledgements

We thank Yan Ren for uploading and performing the initial QC of WGBS data on the University of Adelaide's Phoenix HPC.

Supplementary information

Additional file 1. Supplementary figures referred to in the main text.

Additional file 2. WGBS mapping statistics.

Additional file 3. Table describing the number of CpGs in each reference and what percentage of those have 10X coverage.

Additional file 4. Mean global CpG coverage.

Additional file 5. Global CpG methylation.

Additional file 6. Methylation of CpGs in different genomic regions.

Additional file 7. Alignment statistics of cross-reference genome CpG mapping

Additional file 8. SNP and SV enrichment at CpG sites.

Additional file 9. Methylation quantification bias between genomes.

Additional file 10. Table containing the variable CpG sites between the genomes.

Additional file 11. Table of differentially methylated regions that exhibited direction changes.

Additional file 12. 100kb windows that display quantification bias when mapped between the two genomes.

Additional file 13. Number of DMRs in each comparison.

Additional file 14. Number of up and down-regulated genes in each comparison.

Additional file 15. DMR-DEG overlap after normalising for length.

Additional file 16. Minimap2 accuracy in detecting SNPs introduced to the reference genome.

Additional file 17. Sample information.

Additional file 18. Results from the canonical correlation analysis

Additional file 19. List of imprinted genes.

References

1. Jansz N. DNA methylation dynamics at transposable elements in mammals. Essays in Biochemistry. 2019;63 6:677-89. doi:10.1042/ebc20190039.

2. Li E and Zhang Y. DNA methylation in mammals. Cold Spring Harb Perspect Biol. 2014;6 5:a019133. doi:10.1101/cshperspect.a019133.

3. Ramsahoye BH, Biniszkiewicz D, Lyko F, Clark V, Bird AP and Jaenisch R. Non-CpG methylation is prevalent in embryonic stem cells and may be mediated by DNA methyltransferase 3a. Proceedings of the National Academy of Sciences. 2000;97 10:5237-42. doi:doi:10.1073/pnas.97.10.5237.

4. Ziller MJ, Müller F, Liao J, Zhang Y, Gu H, Bock C, et al. Genomic Distribution and Inter-Sample Variation of Non-CpG Methylation across Human Cell Types. PLOS Genetics. 2011;7 12:e1002389. doi:10.1371/journal.pgen.1002389.

5. Moore LD, Le T and Fan G. DNA Methylation and Its Basic Function. Neuropsychopharmacology. 2013;38 1:23-38. doi:10.1038/npp.2012.112.

6. Kass SU, Landsberger N and Wolffe AP. DNA methylation directs a time-dependent repression of transcription initiation. Curr Biol. 1997;7 3:157-65. doi:10.1016/s0960-9822(97)70086-1.

7. Smith J, Sen S, Weeks RJ, Eccles MR and Chatterjee A. Promoter DNA Hypermethylation and Paradoxical Gene Activation. Trends in Cancer. 2020;6 5:392-406. doi:https://doi.org/10.1016/j.trecan.2020.02.007.

8. Cho J-W, Shim HS, Lee CY, Park SY, Hong MH, Lee I, et al. The importance of enhancer methylation for epigenetic regulation of tumorigenesis in squamous lung cancer. Experimental & Molecular Medicine. 2022;54 1:12-22. doi:10.1038/s12276-021-00718-4.

9. Spainhour JCG, Lim HS, Yi SV and Qiu P. Correlation Patterns Between DNA Methylation and Gene Expression in The Cancer Genome Atlas. Cancer Informatics. 2019;18 doi:10.1177/1176935119828776.

10. Charlet J, Duymich Christopher E, Lay Fides D, Mundbjerg K, Dalsgaard Sørensen K, Liang G, et al. Bivalent Regions of Cytosine Methylation and H3K27 Acetylation Suggest an Active Role for DNA Methylation at Enhancers. Molecular Cell. 2016;62 3:422-31. doi:10.1016/j.molcel.2016.03.033.

11. Creyghton MP, Cheng AW, Welstead GG, Kooistra T, Carey BW, Steine EJ, et al. Histone H3K27ac separates active from poised enhancers and predicts developmental state. Proceedings of the National Academy of Sciences. 2010;107 50:21931-6. doi:10.1073/pnas.1016071107.

12. Kang Y, Kim YW, Kang J and Kim A. Histone H3K4me1 and H3K27ac play roles in nucleosome eviction and eRNA transcription, respectively, at enhancers. The FASEB Journal. 2021;35 8 doi:10.1096/fj.202100488r.

13. Wang M, Hancock TP, MacLeod IM, Pryce JE, Cocks BG and Hayes BJ. Putative enhancer sites in the bovine genome are enriched with variants affecting complex traits. Genet Sel Evol. 2017;49 1:56. doi:10.1186/s12711-017-0331-4.

14. Zhu Y, Zhou Z, Huang T, Zhang Z, Li W, Ling Z, et al. Mapping and analysis of a spatiotemporal H3K27ac and gene expression spectrum in pigs. Sci China Life Sci. 2022;65 8:1517-34. doi:10.1007/s11427-021-2034-5.

15. Wulfridge P, Langmead B, Feinberg AP and Hansen KD. Analyzing whole genome bisulfite sequencing data from highly divergent genotypes. Nucleic Acids Research. 2019;47 19:e117-e. doi:10.1093/nar/gkz674.

16. Brandt DYC, Aguiar VRC, Bitarello BD, Nunes K, Goudet J and Meyer D. Mapping Bias Overestimates Reference Allele Frequencies at the HLA Genes in the 1000 Genomes Project Phase I Data. G3 Genes|Genomes|Genetics. 2015;5 5:931-41. doi:10.1534/g3.114.015784.

17. Degner JF, Marioni JC, Pai AA, Pickrell JK, Nkadori E, Gilad Y, et al. Effect of read-mapping biases on detecting allele-specific expression from RNA-sequencing data. Bioinformatics. 2009;25 24:3207-12. doi:10.1093/bioinformatics/btp579.

18. Salavati M, Bush SJ, Palma-Vera S, McCulloch MEB, Hume DA and Clark EL. Elimination of Reference Mapping Bias Reveals Robust Immune Related Allele-Specific Expression in Crossbred Sheep. Frontiers in Genetics. 2019;10 doi:10.3389/fgene.2019.00863.

19. Chen N-C, Solomon B, Mun T, Iyer S and Langmead B. Reference flow: reducing reference bias using multiple population genomes. Genome Biology. 2021;22 1:8. doi:10.1186/s13059-020-02229-3.

20. Groza C, Kwan T, Soranzo N, Pastinen T and Bourque G. Personalized and graph genomes reveal missing signal in epigenomic data. Genome Biology. 2020;21 1:124. doi:10.1186/s13059-020-02038-8.

21. Crysnanto D and Pausch H. Bovine breed-specific augmented reference graphs facilitate accurate sequence read mapping and unbiased variant discovery. Genome Biology. 2020;21 1:184. doi:10.1186/s13059-020-02105-0.

22. Lloret-Villas A, Bhati M, Kadri NK, Fries R and Pausch H. Investigating the impact of reference assembly choice on genomic analyses in a cattle breed. BMC Genomics. 2021;22 1 doi:10.1186/s12864-021-07554-w.

23. Günther T and Nettelblad C. The presence and impact of reference bias on population genomic studies of prehistoric human populations. PLOS Genetics. 2019;15 7:e1008302. doi:10.1371/journal.pgen.1008302.

24. Wang T, Antonacci-Fulton L, Howe K, Lawson HA, Lucas JK, Phillippy AM, et al. The Human Pangenome Project: a global resource to map genomic diversity. Nature. 2022;604 7906:437-46. doi:10.1038/s41586-022-04601-8.

25. Liao W-W, Asri M, Ebler J, Doerr D, Haukness M, Hickey G, et al. A draft human pangenome reference. Nature. 2023;617 7960:312-24. doi:10.1038/s41586-023-05896-x.

26. Smith TPL, Bickhart DM, Boichard D, Chamberlain AJ, Djikeng A, Jiang Y, et al. The Bovine Pangenome Consortium: democratizing production and accessibility of genome assemblies for global cattle breeds and other bovine species. Genome Biology. 2023;24 1:139. doi:10.1186/s13059-023-02975-0.

27. Woodhouse MR, Cannon EK, Portwood JL, Harper LC, Gardiner JM, Schaeffer ML, et al. A pan-genomic approach to genome databases using maize as a model system. BMC Plant Biology. 2021;21 1:385. doi:10.1186/s12870-021-03173-5.

28. McTavish EJ, Decker JE, Schnabel RD, Taylor JF and Hillis DM. New World cattle show ancestry from multiple independent domestication events. Proceedings of the National Academy of Sciences. 2013;110 15:E1398-E406. doi:doi:10.1073/pnas.1303367110.

29. Bruford MW, Bradley DG and Luikart G. DNA markers reveal the complexity of livestock domestication. Nature Reviews Genetics. 2003;4 11:900-10. doi:10.1038/nrg1203.

30. Ajmone-Marsan P, Garcia JF and Lenstra JA. On the origin of cattle: How aurochs became cattle and colonized the world. Evolutionary Anthropology: Issues, News, and Reviews. 2010;19 4:148-57. doi:https://doi.org/10.1002/evan.20267.

31. MacHugh DE, Larson G and Orlando L. Taming the Past: Ancient DNA and the Study of Animal Domestication. Annual Review of Animal Biosciences. 2017;5 1:329-51. doi:10.1146/annurev-animal-022516-022747.

32. Loftus RT, MacHugh DE, Bradley DG, Sharp PM and Cunningham P. Evidence for two independent domestications of cattle. Proceedings of the National Academy of Sciences. 1994;91 7:2757-61. doi:doi:10.1073/pnas.91.7.2757.

33. Elzo MA, Johnson DD, Wasdin JG and Driver JD. Carcass and meat palatability breed differences and heterosis effects in an Angus–Brahman multibreed population. Meat Science. 2012;90 1:87-92. doi:https://doi.org/10.1016/j.meatsci.2011.06.010.

34. Dikmen S, Mateescu RG, Elzo MA and Hansen PJ. Determination of the optimum contribution of Brahman genetics in an Angus-Brahman multibreed herd for regulation of body temperature during hot weather. Journal of Animal Science. 2018;96 6:2175-83. doi:10.1093/jas/sky133.

35. Goszczynski DE, Corbi-Botto CM, Durand HM, Rogberg-Muñoz A, Munilla S, Peral-Garcia P, et al. Evidence of positive selection towards Zebuine haplotypes in the BoLA region of Brangus cattle. Animal. 2018;12 2:215-23. doi:https://doi.org/10.1017/S1751731117001380.

36. Koren S, Rhie A, Walenz BP, Dilthey AT, Bickhart DM, Kingan SB, et al. De novo assembly of haplotype-resolved genomes with trio binning. Nat Biotechnol. 2018;36 12:1174-82. doi:10.1038/nbt.4277.

37. Decker JE, Mckay SD, Rolf MM, Kim J, Molina Alcalá A, Sonstegard TS, et al. Worldwide Patterns of Ancestry, Divergence, and Admixture in Domesticated Cattle. PLoS Genetics. 2014;10 3:e1004254. doi:10.1371/journal.pgen.1004254.

38. Hiendleder S, Lewalski H and Janke A. Complete mitochondrial genomes of Bos taurus and Bos indicus provide new insights into intra-species variation, taxonomy and domestication. Cytogenetic and Genome Research. 2008;120 1-2:150-6. doi:10.1159/000118756.

39. Low WY, Tearle R, Liu R, Koren S, Rhie A, Bickhart DM, et al. Haplotype-resolved genomes provide insights into structural variation and gene content in Angus and Brahman cattle. Nature Communications. 2020;11 1:1-14.

40. Yang J, Horton JR, Akdemir KC, Li J, Huang Y, Kumar J, et al. Preferential CEBP binding to T:G mismatches and increased C-to-T human somatic mutations. Nucleic Acids Research. 2021;49 9:5084-94. doi:10.1093/nar/gkab276.

41. Żemojtel T, Kiełbasa SM, Arndt PF, Behrens S, Bourque G and Vingron M. CpG Deamination Creates Transcription Factor–Binding Sites with High Efficiency. Genome Biology and Evolution. 2011;3 0:1304-11. doi:10.1093/gbe/evr107.

42. Van Laere A-S, Nguyen M, Braunschweig M, Nezer C, Collette C, Moreau L, et al. A regulatory mutation in IGF2 causes a major QTL effect on muscle growth in the pig. Nature. 2003;425 6960:832-6. doi:10.1038/nature02064.

43. Zhang YQ, Yang LX, Kucherlapati M, Hadjipanayis A, Pantazi A, Bristow CA, et al. Global impact of somatic structural variation on the DNA methylome of human cancers. Genome Biology. 2019;20 1 doi:10.1186/s13059-019-1818-9.

44. Lutz PE, Almeid D, Belzeaux R, Yalcin I and Turecki G. Epigenetic regulation of the kappa opioid receptor gene by an insertion-deletion in the promoter region. European Neuropsychopharmacology. 2018;28 2:334-40. doi:10.1016/j.euroneuro.2017.12.013.

45. Lawson HA, Cheverud JM and Wolf JB. Genomic imprinting and parent-of-origin effects on complex traits. Nat Rev Genet. 2013;14 9:609-17. doi:10.1038/nrg3543.

46. Shi W, Krella A, Orth A, Yu Y and Fundele R. Widespread disruption of genomic imprinting in adult interspecies mouse (Mus) hybrids. Genesis. 2005;43 3:100-8. doi:10.1002/gene.20161.

47. Vaughn RN, Kochan KJ, Torres AK, Du M, Riley DG, Gill CA, et al. Skeletal Muscle Expression of Actinin-3 (ACTN3) in Relation to Feed Efficiency Phenotype of F-2 Bos indicus-Bos taurus Steers. Frontiers in Genetics. 2022;13 doi:10.3389/fgene.2022.796038.

48. Pan ZX, Zhang JL, Zhang JB, Zhou B, Chen J, Jiang ZH, et al. Expression Profiles of the Insulin-like Growth Factor System Components in Liver Tissue during Embryonic and Postnatal Growth of Erhualian and Yorkshire Reciprocal Cross F-1 Pigs. Asian-Australasian Journal of Animal Sciences. 2012;25 7:903-12. doi:10.5713/ajas.2011.11385.

49. Moore GE, Ishida M, Demetriou C, Al-Olabi L, Leon LJ, Thomas AC, et al. The role and interaction of imprinted genes in human fetal growth. Philos Trans R Soc Lond B Biol Sci. 2015;370 1663:20140074. doi:10.1098/rstb.2014.0074.

50. Eggermann T, Davies JH, Tauber M, van den Akker E, Hokken-Koelega A, Johansson G, et al. Growth Restriction and Genomic Imprinting-Overlapping Phenotypes Support the Concept of an Imprinting Network. Genes. 2021;12 4 doi:10.3390/genes12040585.

51. Yuen RKC, Jiang R, Penaherrera MS, McFadden DE and Robinson WP. Genome-wide mapping of imprinted differentially methylated regions by DNA methylation profiling of human placentas from triploidies. Epigenetics & Chromatin. 2011;4 doi:10.1186/1756-8935-4-10.

52. Doria S, Sousa M, Fernandes S, Ramalho C, Brandao O, Matias A, et al. Gene expression pattern of IGF2, PHLDA2, PEG10 and CDKN1C imprinted genes in spontaneous miscarriages or fetal deaths. Epigenetics. 2010;5 5:444-50. doi:10.4161/epi.5.5.12118.

53. Piedrahita JA. The Role of Imprinted Genes in Fetal Growth Abnormalities. Birth Defects Research Part a-Clinical and Molecular Teratology. 2011;91 8:682-92. doi:10.1002/bdra.20795.

54. Amat S, Dahlen CR, Swanson KC, Ward AK, Reynolds LP and Caton JS. Bovine Animal Model for Studying the Maternal Microbiome, in utero Microbial Colonization and Their Role in Offspring Development and Fetal Programming. Front Microbiol. 2022;13:854453. doi:10.3389/fmicb.2022.854453.

55. Peruffo A and Cozzi B. Bovine Brain: An in vitro Translational Model in Developmental Neuroscience and Neurodegenerative Research. Front Pediatr. 2014;2:74. doi:10.3389/fped.2014.00074.

56. Chen Z, Robbins KM, Wells KD and Rivera RM. Large offspring syndrome: a bovine model for the human loss-of-imprinting overgrowth syndrome Beckwith-Wiedemann. Epigenetics. 2013;8 6:591-601.

57. Malhi PS, Adams GP and Singh J. Bovine Model for the Study of Reproductive Aging in Women: Follicular, Luteal, and Endocrine Characteristics1. Biology of Reproduction. 2005;73 1:45-53. doi:10.1095/biolreprod.104.038745.

58. MacPhillamy C, Alinejad-Rokny H, Pitchford WS and Low WY. Cross-species enhancer prediction using machine learning. Genomics. 2022;114 5:110454. doi:10.1016/j.ygeno.2022.110454.

59. Li H. Minimap2: pairwise alignment for nucleotide sequences. Bioinformatics. 2018;34 18:3094-100.

60. Zhou D, Li Z, Yu D, Wan L, Zhu Y, Lai M, et al. Polymorphisms involving gain or loss of CpG sites are significantly enriched in trait-associated SNPs. Oncotarget. 2015;6 37.

61. Leenen FAD, Muller CP and Turner JD. DNA methylation: conducting the orchestra from exposure to phenotype? Clinical Epigenetics. 2016;8 1:92. doi:10.1186/s13148-016-0256-8.

62. Thomson K, Game J, Karouta C, Morgan IG and Ashby R. Correlation between small-scale methylation changes and gene expression during the development of myopia. The FASEB Journal. 2022;36 1:e22129. doi:https://doi.org/10.1096/fj.202101487R.

63. He Y, Hariharan M, Gorkin DU, Dickel DE, Luo C, Castanon RG, et al. Spatiotemporal DNA methylome dynamics of the developing mouse fetus. Nature. 2020;583 7818:752-9. doi:10.1038/s41586-020-2119-x.

64. Hama N, Totoki Y, Miura F, Tatsuno K, Saito-Adachi M, Nakamura H, et al. Epigenetic landscape influences the liver cancer genome architecture. Nature Communications. 2018;9 doi:10.1038/s41467-018-03999-y.

65. Zhou Y, Liu S, Hu Y, Fang L, Gao Y, Xia H, et al. Comparative whole genome DNA methylation profiling across cattle tissues reveals global and tissue-specific methylation patterns. BMC Biology. 2020;18 1:85. doi:10.1186/s12915-020-00793-5.

66. Valiente-Mullor C, Beamud B, Ansari I, Francés-Cuesta C, García-González N, Mejía L, et al. One is not enough: On the effects of reference genome for the mapping and subsequent analyses of short-reads. PLOS Computational Biology. 2021;17 1:e1008678. doi:10.1371/journal.pcbi.1008678.

67. Capra E, Lazzari B, Milanesi M, Nogueira GP, Garcia Jf, Utsunomiya YT, et al. Comparison between indicine and taurine cattle DNA methylation reveals epigenetic variation associated to differences in morphological adaptive traits. Epigenetics. 2023;18 1:2163363. doi:10.1080/15592294.2022.2163363.

68. Gonzalez E, Kulkarni H, Bolivar H, Mangano A, Sanchez R, Catano G, et al. The influence of CCL3L1 gene-containing segmental duplications on HIV-1/AIDS susceptibility. Science. 2005;307 5714:1434-40. doi:10.1126/science.1101160.

69. Marshall CR, Noor A, Vincent JB, Lionel AC, Feuk L, Skaug J, et al. Structural variation of chromosomes in autism spectrum disorder. Am J Hum Genet. 2008;82 2:477-88. doi:10.1016/j.ajhg.2007.12.009.

70. Kumar RA, KaraMohamed S, Sudi J, Conrad DF, Brune C, Badner JA, et al. Recurrent 16p11.2 microdeletions in autism. Hum Mol Genet. 2008;17 4:628-38. doi:10.1093/hmg/ddm376.

71. Weiss LA, Shen Y, Korn JM, Arking DE, Miller DT, Fossdal R, et al. Association between microdeletion and microduplication at 16p11.2 and autism. N Engl J Med. 2008;358 7:667-75. doi:10.1056/NEJMoa075974.

72. Bell DA, Taylor JA, Paulson DF, Robertson CN, Mohler JL and Lucier GW. Genetic risk and carcinogen exposure: a common inherited defect of the carcinogen-metabolism gene glutathione S-transferase M1 (GSTM1) that increases susceptibility to bladder cancer. J Natl Cancer Inst. 1993;85 14:1159-64. doi:10.1093/jnci/85.14.1159.

73. Lamb HJ, Ross EM, Nguyen LT, Lyons RE, Moore SS and Hayes BJ. Characterization of the poll allele in Brahman cattle using long-read Oxford Nanopore sequencing. Journal of Animal Science. 2020;98 5 doi:10.1093/jas/skaa127.

74. Rothammer S, Capitan A, Mullaart E, Seichter D, Russ I and Medugorac I. The 80-kb DNA duplication on BTA1 is the only remaining candidate mutation for the polled phenotype of Friesian origin. Genet Sel Evol. 2014;46 1:44. doi:10.1186/1297-9686-46-44.

75. Jacinto JGP, Häfliger IM, Letko A, Drögemüller C and Agerholm JS. A large deletion in the COL2A1 gene expands the spectrum of pathogenic variants causing bulldog calf syndrome in cattle. Acta Vet Scand. 2020;62 1:49. doi:10.1186/s13028-020-00548-w.

76. Akalin A, Kormaksson M, Li S, Garrett-Bakelman FE, Figueroa ME, Melnick A, et al. methylKit: a comprehensive R package for the analysis of genome-wide DNA methylation profiles. Genome Biology. 2012;13 10:R87. doi:10.1186/gb-2012-13-10-r87.

77. Kishore K, de Pretis S, Lister R, Morelli MJ, Bianchi V, Amati B, et al. methylPipe and compEpiTools: a suite of R packages for the integrative analysis of epigenomics data. BMC Bioinformatics. 2015;16 1:313. doi:10.1186/s12859-015-0742-6.

78. Park Y, Figueroa ME, Rozek LS and Sartor MA. MethylSig: a whole genome DNA methylation analysis pipeline. Bioinformatics. 2014;30 17:2414-22. doi:10.1093/bioinformatics/btu339.

79. Lee HJ, Lowdon RF, Maricque B, Zhang B, Stevens M, Li D, et al. Developmental enhancers revealed by extensive DNA methylome maps of zebrafish early embryos. Nature Communications. 2015;6 1:6315. doi:10.1038/ncomms7315.

80. Alajem A, Roth H, Ratgauzer S, Bavli D, Motzik A, Lahav S, et al. DNA methylation patterns expose variations in enhancer-chromatin modifications during embryonic stem cell differentiation. PLOS Genetics. 2021;17 4:e1009498. doi:10.1371/journal.pgen.1009498.

81. Slieker RC, Roost MS, van Iperen L, Suchiman HE, Tobi EW, Carlotti F, et al. DNA Methylation Landscapes of Human Fetal Development. PLoS Genet. 2015;11 10:e1005583. doi:10.1371/journal.pgen.1005583.

82. Slabaugh E, Desai JS, Sartor RC, Lawas LMF, Jagadish SVK and Doherty CJ. Analysis of differential gene expression and alternative splicing is significantly influenced by choice of reference genome. Rna. 2019;25 6:669-84. doi:10.1261/rna.070227.118.

83. Price A and Gibas C. The quantitative impact of read mapping to non-native reference genomes in comparative RNA-Seq studies. PLOS ONE. 2017;12 7:e0180904. doi:10.1371/journal.pone.0180904.

84. Kaminow B, Ballouz S, Gillis J and Dobin A. Pan-human consensus genome significantly improves the accuracy of RNA-seq analyses. Genome Research. 2022;32 4:738-49. doi:10.1101/gr.275613.121.

85. Wu P-Y, Phan JH and Wang MD. Assessing the impact of human genome annotation choice on RNA-seq expression estimates. BMC Bioinformatics. 2013;14 11:S8. doi:10.1186/1471-2105-14-S11-S8.

86. Khan MZ, Ma Y, Ma J, Xiao J, Liu Y, Liu S, et al. Association of DGAT1 With Cattle, Buffalo, Goat, and Sheep Milk and Meat Production Traits. Frontiers in Veterinary Science. 2021;8 doi:10.3389/fvets.2021.712470.

87. Abeel T, Van de Peer Y and Saeys Y. Toward a gold standard for promoter prediction evaluation. Bioinformatics. 2009;25 12:I313-I20. doi:10.1093/bioinformatics/btp191.

88. Tsuda N, Kumadaki S, Higashi C, Ozawa M, Shinozaki M, Kato Y, et al. Intestine-Targeted DGAT1 Inhibition Improves Obesity and Insulin Resistance without Skin Aberrations in Mice. PLOS ONE. 2014;9 11:e112027. doi:10.1371/journal.pone.0112027.

89. Zhang XD, Yan JW, Yan GR, Sun XY, Ji J, Li YM, et al. Pharmacological inhibition of diacylglycerol acyltransferase 1 reduces body weight gain, hyperlipidemia, and hepatic steatosis in db/db mice. Acta Pharmacol Sin. 2010;31 11:1470-7. doi:10.1038/aps.2010.104.

90. Elzo MA, Riley DG, Hansen GR, Johnson DD, Myer RO, Coleman SW, et al. Effect of breed composition on phenotypic residual feed intake and growth in Angus, Brahman, and Angus x Brahman crossbred cattle. J Anim Sci. 2009;87 12:3877-86. doi:10.2527/jas.2008-1553.

91. Giannoukakis N, Deal C, Paquette J, Goodyer CG and Polychronakos C. Parental genomic imprinting of the human IGF2 gene. Nature genetics. 1993;4 1:98-101.

92. Szabo PE, Tang SHE, Rentsendorj A, Pfeifer GP and Mann JR. Maternal-specific footprints at putative CTCF sites in the H19 imprinting control region give evidence for insulator function. Current Biology. 2000;10 10:607-10. doi:10.1016/s0960-9822(00)00489-9.

93. Yang YW, Hu JF, Ulaner GA, Li T, Yao XM, Vu TH, et al. Epigenetic regulation of Igf2/H19 imprinting at CTCF insulator binding sites. Journal of Cellular Biochemistry. 2003;90 5:1038-55. doi:10.1002/jcb.10684.

94. Ren Y, Tseng E, Smith TPL, Hiendleder S, Williams JL and Low WY. Long read isoform sequencing reveals hidden transcriptional complexity between cattle subspecies. BMC Genomics. 2023;24 1:108. doi:10.1186/s12864-023-09212-9.

95. Paten B, Novak AM, Eizenga JM and Garrison E. Genome graphs and the evolution of genome inference. Genome Res. 2017;27 5:665-76. doi:10.1101/gr.214155.116.

96. Liu R, Tearle R, Low WY, Chen T, Thomsen D, Smith TPL, et al. Distinctive gene expression patterns and imprinting signatures revealed in reciprocal crosses between cattle sub-species. BMC Genomics. 2021;22 1 doi:10.1186/s12864-021-07667-2.

97. Di Tommaso P, Chatzou M, Floden EW, Barja PP, Palumbo E and Notredame C. Nextflow enables reproducible computational workflows. Nature Biotechnology. 2017;35 4:316-9. doi:10.1038/nbt.3820.

98. Rosen BD, Bickhart DM, Schnabel RD, Koren S, Elsik CG, Tseng E, et al. De novo assembly of the cattle reference genome with single-molecule sequencing. Gigascience. 2020;9 3:giaa021-giaa. doi:10.1093/gigascience/giaa021.

99. Li H, Handsaker B, Wysoker A, Fennell T, Ruan J, Homer N, et al. The Sequence Alignment/Map format and SAMtools. Bioinformatics. 2009;25 16:2078-9. doi:10.1093/bioinformatics/btp352.

100. Okonechnikov K, Conesa A and García-Alcalde F. Qualimap 2: advanced multi-sample quality control for high-throughput sequencing data. Bioinformatics. 2016;32 2:292-4. doi:10.1093/bioinformatics/btv566.

101. Cock PJA, Antao T, Chang JT, Chapman BA, Cox CJ, Dalke A, et al. Biopython: freely available Python tools for computational molecular biology and bioinformatics. Bioinformatics. 2009;25 11:1422-3. doi:10.1093/bioinformatics/btp163.

102. Davis S, Pettengill JB, Luo Y, Payne J, Shpuntoff A, Rand H, et al. CFSAN SNP Pipeline: an automated method for constructing SNP matrices from next-generation sequence data. PeerJ Computer Science. 2015;1:e20.

103. Quinlan AR and Hall IM. BEDTools: a flexible suite of utilities for comparing genomic features. Bioinformatics. 2010;26 6:841-2. doi:10.1093/bioinformatics/btq033.

104. Wang H-Q, Tuominen LK and Tsai C-J. SLIM: a sliding linear model for estimating the proportion of true null hypotheses in datasets with dependence structures. Bioinformatics. 2010;27 2:225-31. doi:10.1093/bioinformatics/btq650.

105. Shumate A and Salzberg SL. Liftoff: accurate mapping of gene annotations. Bioinformatics. 2021;37 12:1639-43. doi:10.1093/bioinformatics/btaa1016.

106. Liao Y, Smyth GK and Shi W. The R package Rsubread is easier, faster, cheaper and better for alignment and quantification of RNA sequencing reads. Nucleic Acids Research. 2019;47 8:e47-e. doi:10.1093/nar/gkz114.

107. Love MI, Huber W and Anders S. Moderated estimation of fold change and dispersion for RNA-seq data with DESeq2. Genome Biology. 2014;15 12:550. doi:10.1186/s13059-014-0550-8.

108. Morison IM, Ramsay JP and Spencer HG. A census of mammalian imprinting. Trends in Genetics. 2005;21 8:457-65. doi:10.1016/j.tig.2005.06.008.

109. Emms DM and Kelly S. OrthoFinder: phylogenetic orthology inference for comparative genomics. Genome Biology. 2019;20 1:238. doi:10.1186/s13059-019-1832-y.

110. Jin F, Li Y, Dixon JR, Selvaraj S, Ye Z, Lee AY, et al. A high-resolution map of the three-dimensional chromatin interactome in human cells. Nature. 2013;503 7475:290-4. doi:10.1038/nature12644.

Tables

Table 3. Sample-wise and group-wise methylation quantification biases.

Sample-wise				Group-wise			
**ID**	**Difference[A]**	**p-value**	**Adj. p-value**	**ID**	**Difference[A]**	**p-value**	**Adj. p-value**
All CpGs[B]				All CpGs[C]			
F103	1.99	5.00E-324	5.00E-324	BTBT	1.98	5.00E-324	5.00E-324
F105	2.16	5.00E-324	5.00E-324				
F52	2.25	5.00E-324	5.00E-324				
F53	1.88	5.00E-324	5.00E-324				
F60	1.73	5.00E-324	5.00E-324				
F7	1.87	5.00E-324	5.00E-324				
F100	1.15	5.00E-324	5.00E-324	BTBI	0.79	5.00E-324	5.00E-324
F104	0.83	5.00E-324	5.00E-324				
F106	0.85	5.00E-324	5.00E-324				
F61	1.06	5.00E-324	5.00E-324				
F74	0.86	5.00E-324	5.00E-324				
F97	0.01	3.69E-02	3.69E-02				
F13	0.66	5.00E-324	5.00E-324	BIBT	0.69	5.00E-324	5.00E-324
F62	0.63	5.00E-324	5.00E-324				
F77	0.73	5.00E-324	5.00E-324				
F80	0.71	5.00E-324	5.00E-324				
F8	0.75	5.00E-324	5.00E-324				
F91	0.65	5.00E-324	5.00E-324				
F22	0.34	5.00E-324	5.00E-324	BIBI	0.34	5.00E-324	5.00E-324
F46	0.34	8.02E-301	8.37E-301				
F56	0.29	5.81E-301	6.34E-301				
F65	0.40	5.00E-324	5.00E-324				

F78	0.36	5.00E-324	5.00E-324				
F99	0.34	5.00E-324	5.00E-324				
Shared[D]				*Shared*[E]			
F103	0.06	1.24E-09	7.41E-09	BTBT	0.05	1.65E-40	6.61E-40
F105	0.06	3.78E-13	9.06E-12				
F52	0.06	2.12E-12	2.55E-11				
F53	0.05	1.36E-08	6.55E-08				
F60	0.04	4.05E-08	1.62E-07				
F7	0.05	6.76E-10	5.41E-09				
F100	0.04	5.22E-06	1.79E-05	BTBI	0.04	1.52E-18	3.04E-18
F104	0.03	2.15E-04	4.29E-04				
F106	0.04	3.88E-04	7.16E-04				
F61	0.04	6.40E-06	1.92E-05				
F74	0.04	2.93E-05	7.82E-05				
F97	0.03	3.89E-02	5.19E-02				
F13	0.03	1.83E-03	2.92E-03	BIBT	0.03	1.85E-16	2.47E-16
F62	0.03	6.50E-03	9.18E-03				
F77	0.03	1.07E-03	1.83E-03				
F80	0.04	3.82E-05	9.17E-05				
F8	0.04	1.34E-04	2.92E-04				
F91	0.03	3.82E-03	5.73E-03				
F22	0.02	1.09E-01	1.14E-01	BIBI	0.02	2.94E-16	2.94E-16
F46	0.03	5.05E-02	6.38E-02				
F56	0.02	7.49E-02	8.99E-02				
F65	0.02	1.65E-01	1.65E-01				

F78	0.02	8.18E-02	9.35E-02				
F99	0.02	1.03E-01	1.13E-01				

[A] Refers to the absolute percentage difference of the mean CpG methylation when mapped to Angus compared to Brahman for a given sample

[B] denotes sample-wise values calculated using all (21,432,071) CpG sites. Refers to Figure 3A.

[C] denotes group-wise values calculated using all CpG sites in all samples within a group (i.e. pooled) (21,432,071 $\times$ 6 = 128,592,426). Refers to Figure 3A.

[D] denotes sample-wise values using only shared (16,204,834) CpG sites. Refers to Figure 3B.

[E] denotes group-wise values calculated using only shared CpG sites in all samples within a group (i.e. pooled) (16,204,834 $\times$ 6 = 97,229,004) CpG sites. Refers to Figure 3B.

Table 4. Significant imprinted DEGs and their overlap with DMRs when using the Brahman reference genome.

Gene ID	Gene name	Protein name	Increased expression in Brahman*	Number of hypo-DMRs in Brahman	Number of hyper-DMRs in Brahman
Breed comparison					
ENSBIXG00005007073	*DSCAM*	DS cell adhesion molecule	No	61	18
ENSBIXG00005012203	*NNAT*	Neuronatin	No	37	1
ENSBIXG00005029958	*LIN28B*	Lin-28 homolog B	Yes	8	1
ENSBIXG00005009822	*DGAT1*	Diacylglycerol O-acyltransferase 1	Yes	3	2
ENSBIXG00005007141	*PPP1R9A*	Protein phosphatase 1 regulatory subunit 9A	Yes	11	5
Dam of origin comparison					
ENSBIXG00005019306	*ZC3H12C*	Zinc finger CCCH-type containing 12C	Yes	5	1
ENSBIXG00005007141	*PPP1R9A*	Protein phosphatase 1 regulatory subunit 9A	Yes	3	2
ENSBIXG00005029958	*LIN28B*	Lin-28 homolog B	Yes	1	0
ENSBIXG00005015804	*RTL1*	Retrotransposon Gag like 1	No	3	0
Sire of origin comparison					
ENSBIXG00005007073	*DSCAM*	DS cell adhesion molecule	No	38	5
ENSBIXG00005021735	*HTR2A*	5-hydroxytryptamine receptor 2A	Yes	18	2
ENSBIXG00005012203	*NNAT*	Neuronatin	No	8	0

ENSBIXG00005009822	*DGAT1*	Diacylglycerol O-acyltransferase 1	Yes	0	1
ENSBIXG00005025714	*MKRN3*	Makorin ring finger protein 3	No	0	0
ENSBIXG00005025694	*NDN*	Necdin MAGE family member	No	0	1
ENSBIXG00005024991	*SLC22A18*	Solute-carrier family 22 member 18	Yes	0	0
ENSBIXG00005013434	*TFPI2*	Tissue factor pathway inhibitor 2	No	25	0

* Increased expression in Brahman denotes genes that were significantly more highly expressed in Brahman than in Angus. “No” denotes that gene was significantly more highly expressed in Angus.

Table 5. Significant imprinted DEGs and their overlap with DMRs when using the Angus reference genome.

Gene ID	Gene name	Protein name	Increased expression in Angus*	Num of hypo-DMRs in Angus	Num of hyper-DMRs in Angus
Breed comparison					
ENSBIXG00000027129	Novel gene		Yes	20	66
ENSBIXG00000021864	*NNAT*	Neuronatin	Yes	2	32
ENSBIXG00000002586	*LIN28B*	Lin-28 homolog B	No	0	6
ENSBIXG00000012321	*DGAT1*	Diacylglycerol O-acyltransferase 1	No	3	3
ENSBIXG00000005197	*PPP1R9A*	Protein phosphatase 1 regulatory subunit 9A	No	9	15
Dam of origin comparison					
ENSBIXG00000011151	*ZC3H12C*	Zinc finger CCCH-type containing 12C	No	0	4
ENSBIXG00000005197	*PPP1R9A*	Protein phosphatase 1 regulatory subunit 9A	No	6	3
ENSBIXG00000002586	*LIN28B*	Lin-28 homolog B	No	0	1
Sire of origin comparison					
ENSBIXG00000027129	*DSCAM*	DS cell adhesion molecule	Yes	5	35
ENSBIXG00000008539	*HTR2A*	5-hydroxytryptamine receptor 2A	No	1	17
ENSBIXG00000021864	*NNAT*	Neuronatin	Yes	1	11
ENSBIXG00000012321	*DGAT1*	Diacylglycerol O-acyltransferase 1	No	0	0
ENSBIXG00000015087	*MKRN3*	Makorin ring finger protein 3	Yes	1	1
ENSBIXG00000015080	*NDN*	Necdin MAGE family member	Yes	1	0
ENSBIXG00000028529	*SLC22A18*	Solute-carrier family 22 member 18	No	0	2

* Increased expression in Angus denotes genes that were significantly more highly expressed in Angus than in Brahman. “No” denotes that gene was significantly more highly expressed in Brahman.

Figures

Figure 1. Overview of methods. A.) Representation of the four genetic groups used in this study. The blue cow represents pure Angus individuals (BTBT). The blue then orange cow represents individuals with an Angus sire and Brahman dam (BTBI). The orange then blue cow represents individuals with a Brahman sire and Angus dam (BIBT). The orange cow represents pure Brahman individuals (BIBI). **B.)** Process of mapping WGBS reads (light green-blue) and RNA-seq reads (green) to both the Brahman and Angus reference genomes. **C.)** Simple representation of shared and breed-specific CpG sites between Brahman and Angus reference genomes. **D.)** Breed-specific CpGs arise from a single nucleotide polymorphism between Brahman and Angus, such as spontaneous deamination of the C to a T. Structural variants, such as indels between the two genomes, can introduce or remove CpGs in one genome relative to the other. **E.)** Simple representation of how differential methylation can be influenced by breed-specific CpGs. The grey boxes demonstrate how a differentially methylated cytosine is identified when both breeds share that site. Essentially, one compares the number of Cs and Ts in group 1 against the number of Cs and Ts in group 2. If one group reports significantly more Cs than the other, it is considered differentially methylated. The yellow boxes represent a breed-specific CpG where only samples from one group have that CpG, so differential methylation cannot be determined. The red boxes represent a situation where the CpG is present in one subspecies, but spontaneous deamination has mutated the CpG site into a TpG site in the other subspecies. In this case, differential methylation can be calculated. However, it will be erroneous as only one group has a true CpG at that site. **F.)** Graphical representation of how breed differences were determined. We compared methylation and gene expression between BTBT and BIBI samples. **G.)** Graphical representation of how we determined parent-of-origin effects (POEs). Maternal POEs were determined by comparing BTBT and BIBT against BIBI and BTBI. Paternal POEs were determined by comparing BTBT and BTBI against BIBI and BIBT.

Figure 2. A.) PCA plot showing separation of genetic groups by methylation. Blue represents BTBT, orange represents BIBI, green represents BTBI and red represents BIBT. The X axis is principal component 1, and the Y axis is principal component 2. **B.)** PCA plot showing separation of genetic groups by gene expression data; colours are same as **A**. The X axis is the first dimension of the logCPM, and the Y axis is the second dimension of the logCPM.

Figure 3. A.) Kernel density estimate (KDE) plots showing global CpG methylation using all CpG sites for all samples in the four genetic groups. Each panel represents a genetic group in the order BTBT, BTBI, BIBT and BIBI. The X-axis represents the methylation percentage for a given CpG site. The Y-axis represents the density. Each reference genome is represented by a different colour; blue for Angus and orange for Brahman. **B.)** Same as A; however, only the shared CpGs were considered. Assoicated p-values for **A** and **B** can be found in Table 3. **C.)** KDE plot showing the methylation values of matched CpGs when mapped to the Angus reference (X-axis) and Brahman reference (Y-axis) for all chromosomes. Only CpGs that differed by > 10 are plotted (0.18% of all CpGs from all samples). **D.)** KDE plot illustrating the illustrating the difference in methylation distribution between the shared and Angus-specific CpG sites when aligning Angus samples to the Angus reference. Blue represents shared CpG sites. Orange represents Angus-specific CpG sites. P-values were determined with a Wilcoxon signed-rank test. The X-axis denotes the methylation percentage. The Y-axis represents the probability density. **E.)** KDE plot illustrating the illustrating the difference in methylation distribution between the shared and Brahman-specific CpG sites when aligning Brahman samples to the Brahman reference. Blue represents shared CpG sites. Orange represents Brahman-specific CpG sites. P-values were determined with a Wilcoxon signed-rank test. X- and Y-axes are the same as **D**.

Figure 1

Click here to access/download;Figure;Figure 1 - methylation-Overview_figure.png

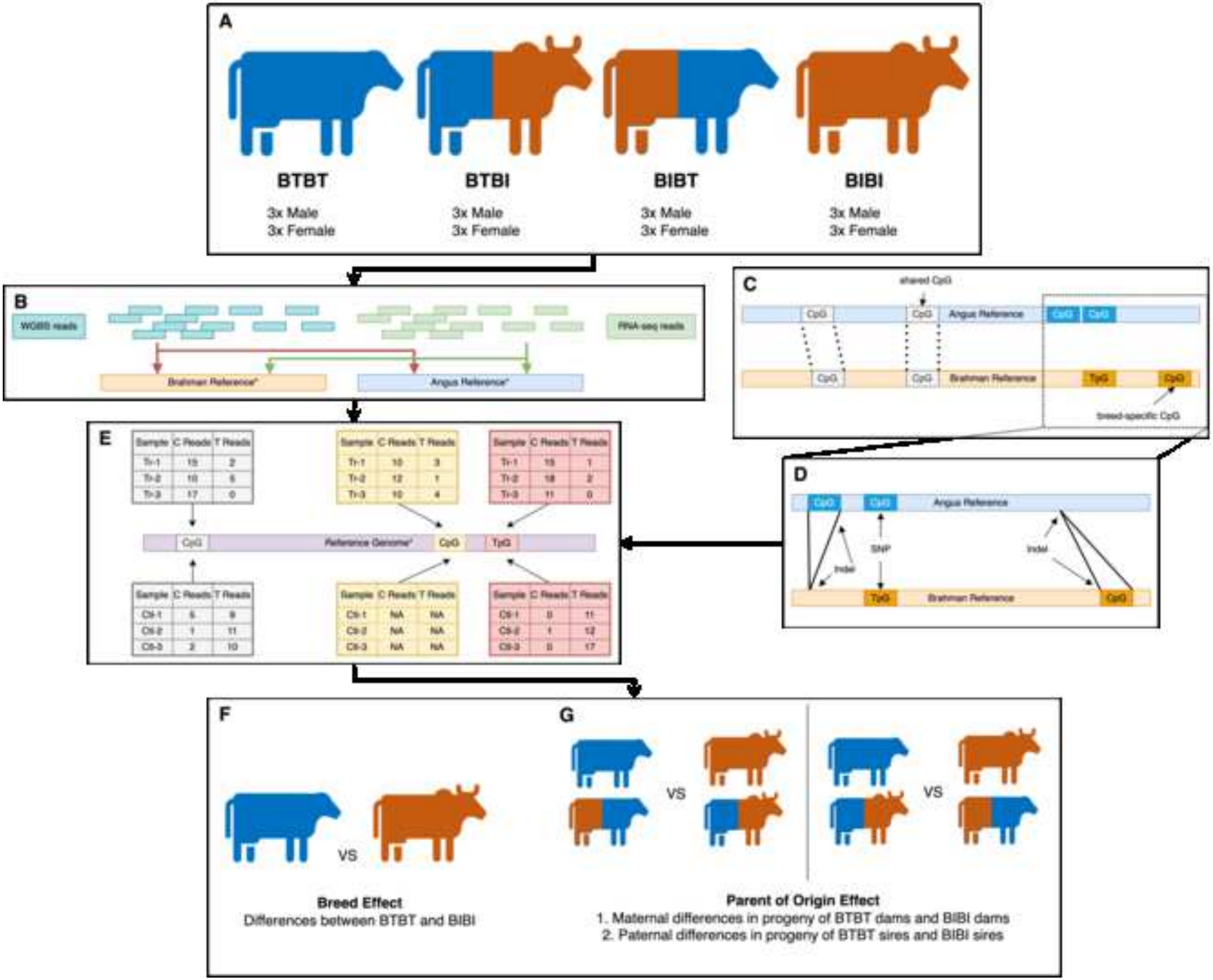

Figure 2

Click here to access/download;Figure;Figure 2 - PCA.Brahman.meth_and_rna.png

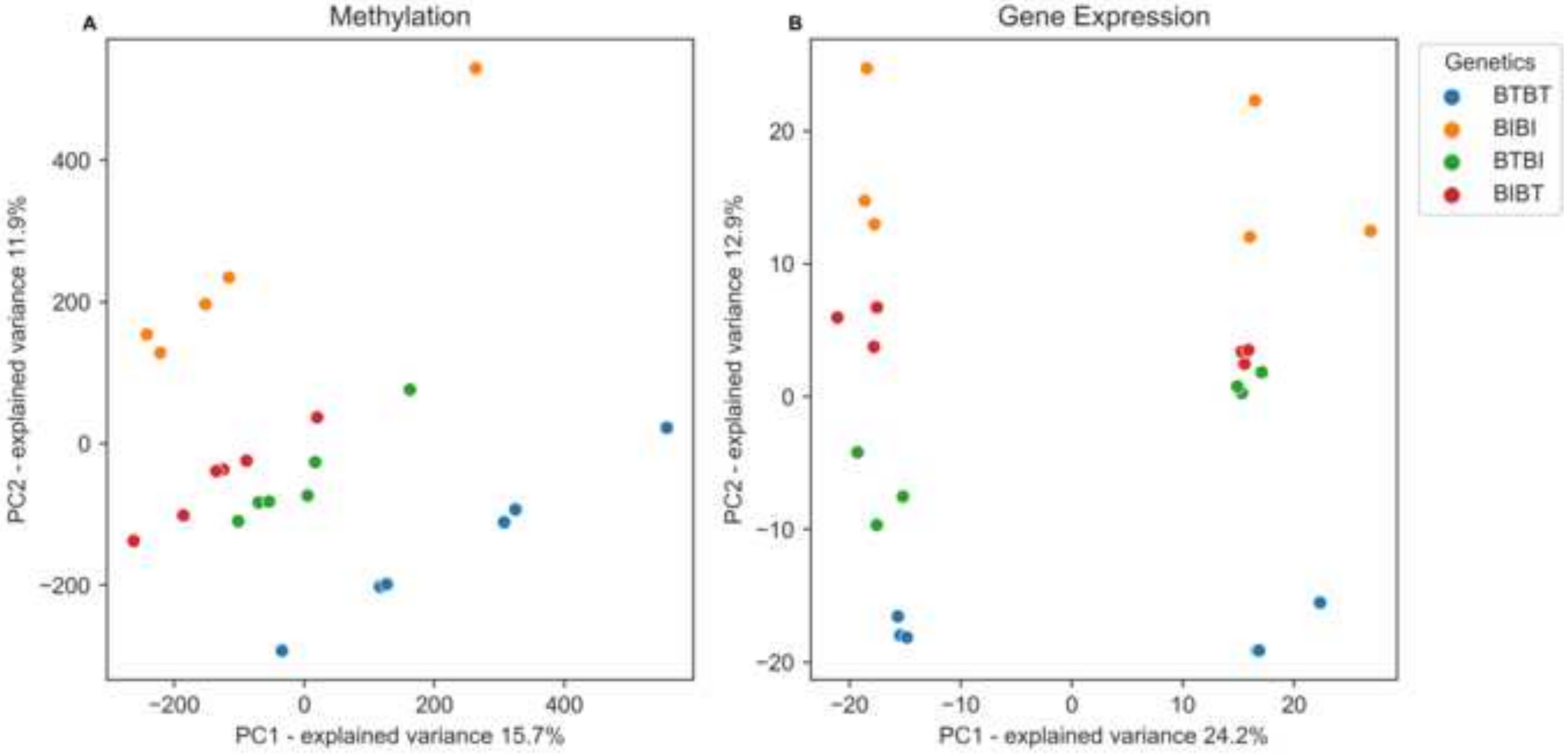

Figure 3

Click here to access/download;Figure;figure3.pdf

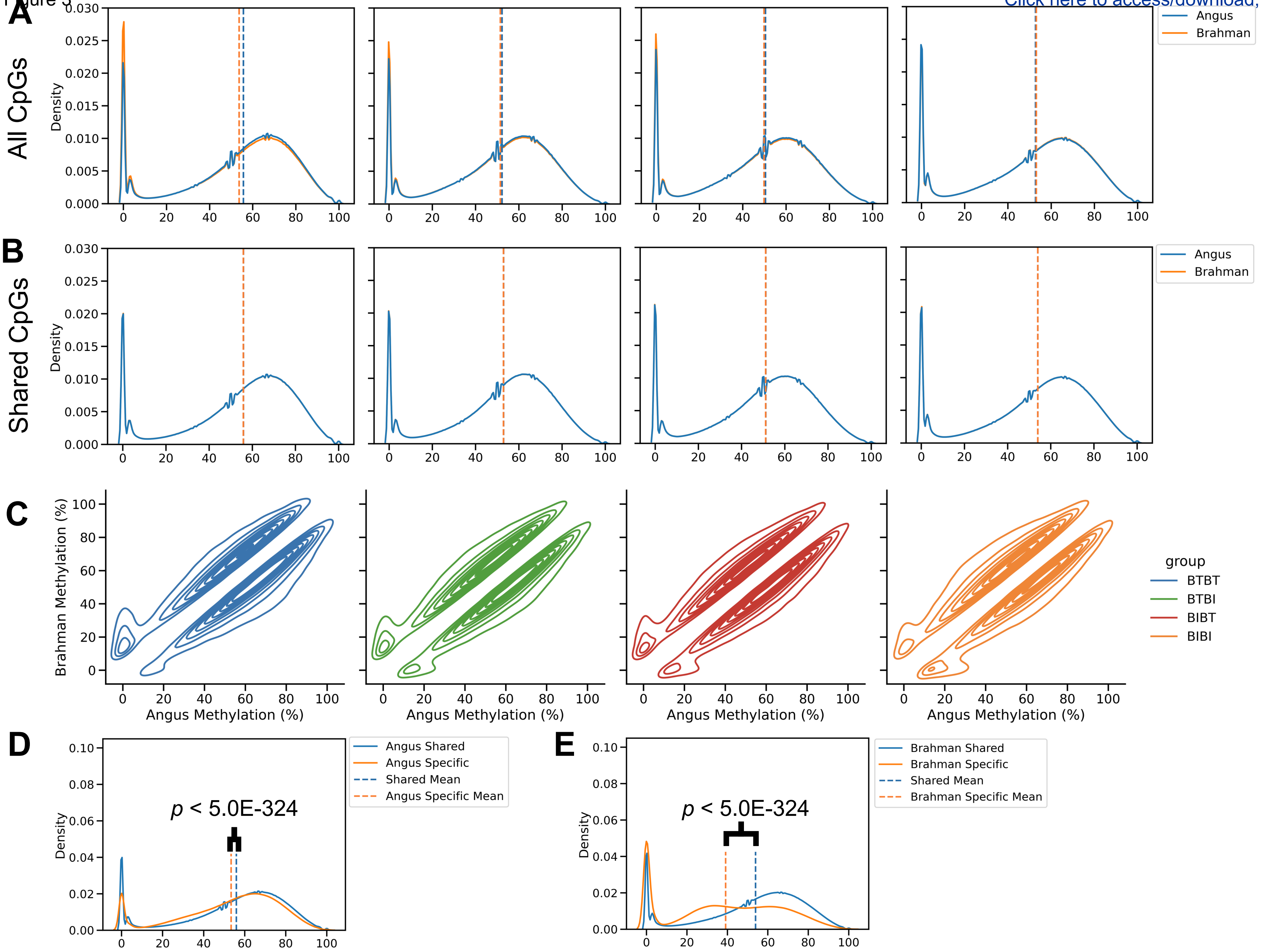

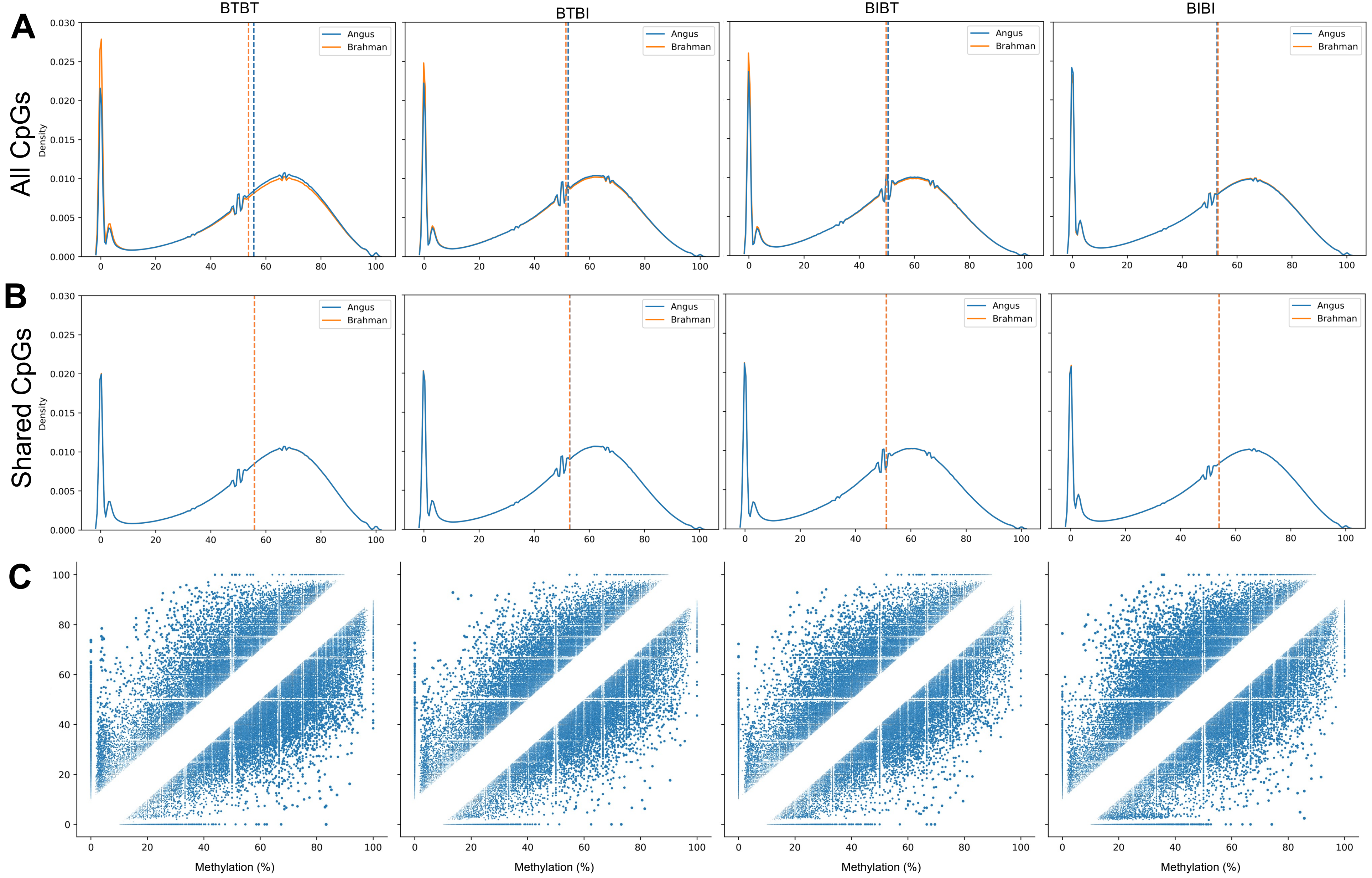
BTBT
BTBI
BIBT
BIBI
A
B
C
All CpGs
Shared CpGs
Density
Angus
Brahman
Methylation (%)
0.000
0.005
0.010
0.015
0.020
0.025
0.030
0
20
40
60
80
100

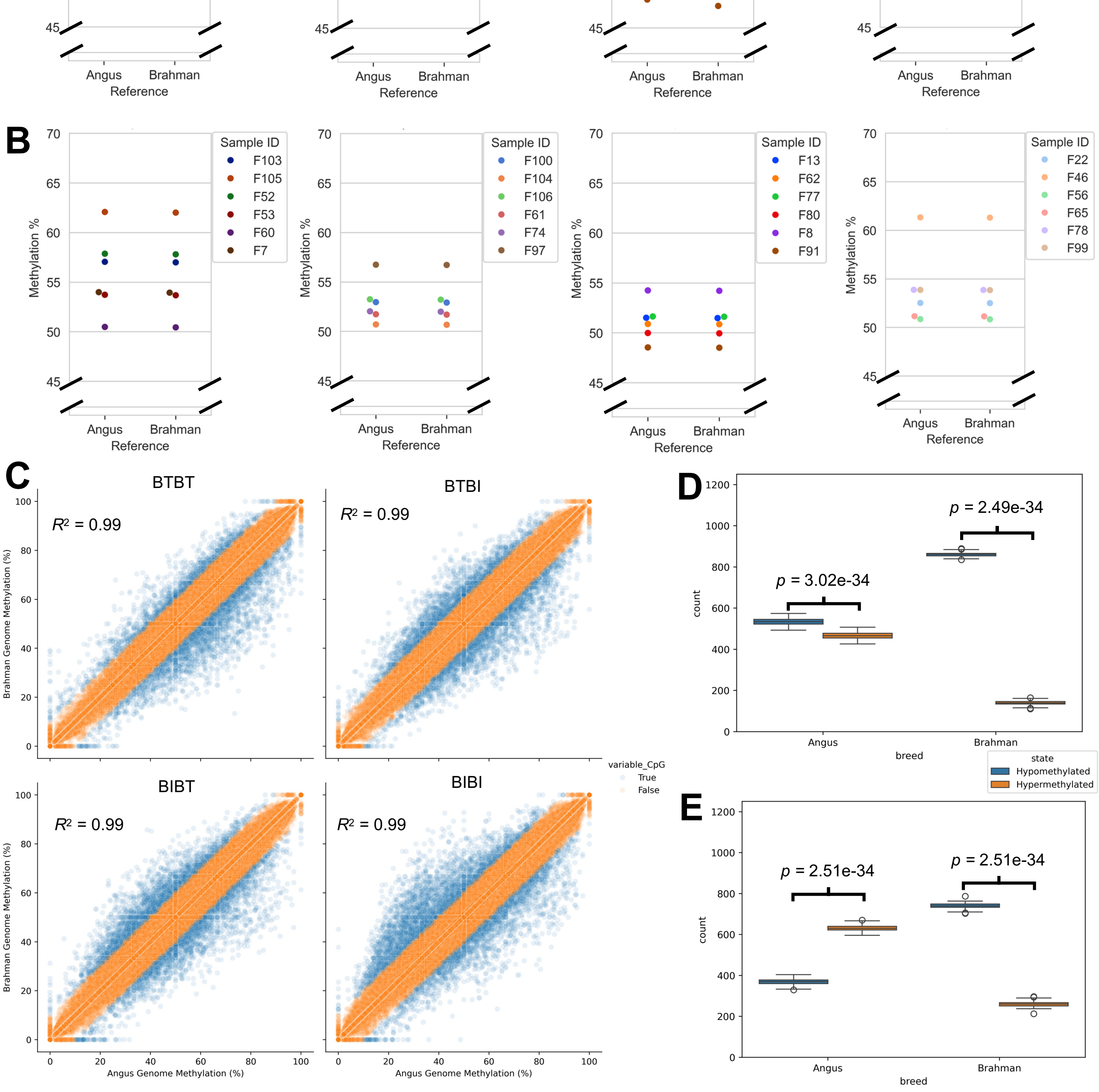
B
C
D
E
Reference
Methylation %
Sample ID
F103
F105
F52
F53
F60
F7
F100
F104
F106
F61
F74
F97
F13
F62
F77
F80
F8
F91
F22
F46
F56
F65
F78
F99
Angus
Brahman
BTBT
BTBI
BIBT
BIBI
R^2 = 0.99
Brahman Genome Methylation (%)
Angus Genome Methylation (%)
variable_CpG
True
False
p = 3.02e-34
p = 2.49e-34
p = 2.51e-34
p = 2.51e-34
count
breed
state
Hypomethylated
Hypermethylated

Supplementary Table 1

Click here to access/download
Supplementary Material
S. table 1 - WGBS mapping stats.xlsx

Supplementary Table 2

Click here to access/download
Supplementary Material
S. table 2 - global coverage stats.xlsx

Supplementary Table 3

Click here to access/download
Supplementary Material
S. table 3 - CpG coverage stats.xlsx

Supplementary Table 4

Click here to access/download
Supplementary Material
S. table 4 - global methylation.xlsx

Supplementary Table 5

Click here to access/download
Supplementary Material
S. table 5 - Descriptive stats regional meth.xlsx

Supplementary Table 6

Click here to access/download
Supplementary Material
S. table 6 - CpG Mapping between refs.xlsx

Supplementary Table 7

Click here to access/download
Supplementary Material
S. table 7 - SNPs and SV enrichment.xlsx

Supplementary Table 8

Click here to access/download
Supplementary Material
S. table 8 - CpG reference bias.xlsx

Supplementary Table 9

Click here to access/download
Supplementary Material
S. table 9 - variable_cpgs.xlsx

Supplementary Table 10

Click here to access/download
Supplementary Material
S. table 10 - meth change.xlsx

Supplementary Table 11

Click here to access/download

Supplementary Material

S. table 11 - REF TO REF QUANT BIAS.xlsx

Supplementary Table 12

Click here to access/download
Supplementary Material
S. table 12 - DMRs.xlsx

Supplementary Table 13

Click here to access/download
Supplementary Material
S. table 13 - DEGs_DESeq2.xlsx

Supplementary Table 14

Click here to access/download
Supplementary Material
S. table 14 - DMR_DEG_overlap_normalised.xlsx

Supplementary Table 15

Click here to access/download
Supplementary Material
S. table 15 - minimap2_accuarcy.xlsx

Supplementary Table 16

Click here to access/download
Supplementary Material
S. table 16 - sample info.xlsx

Supplementary Table 17

Click here to access/download
Supplementary Material
S. table 17 - CCA.xlsx

Supplementary Table 18

Click here to access/download
Supplementary Material
S. table 18 - imprinted_genes.xlsx

Supplementary Figures

Click here to access/download
Supplementary Material
Supplementary figures.docx

Response document

Click here to access/download
Supplementary Material
Response_to_second_round_comments_20240601.docx
