# Supplementary material for: DNA methylation analysis to differentiate reference, breed, and parent-of-origin effects in the bovine pangenome era
Source: Gigascience. 2024 Oct 17;13:giae061. doi: 10.1093/gigascience/giae061 (PMC11484048; doi:10.1093/gigascience/giae061)
Supplement: giae061_Supplemental_Files [file giae061_supplemental_files.zip › Supplementary figures.docx]

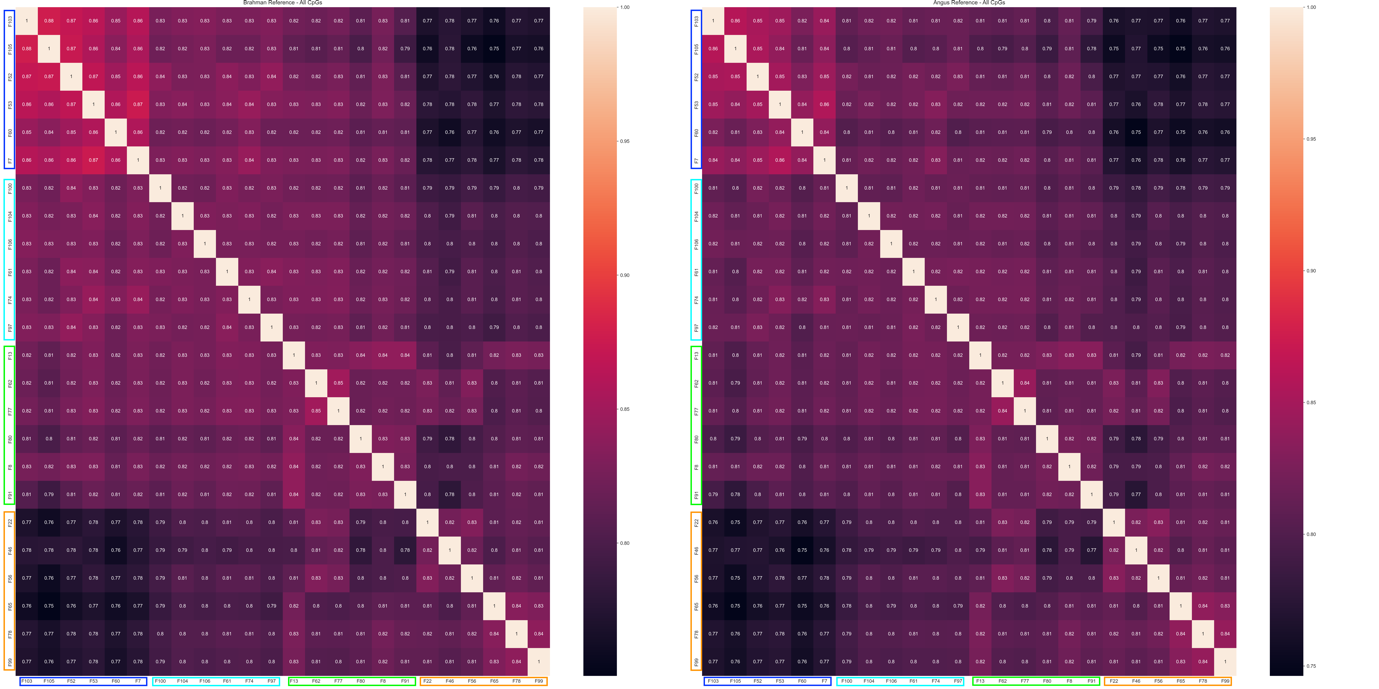


**Supplementary Figure 1. Correlation heat maps of all 24 samples mapped to Brahman and Angus reference genomes. Left)** Correlation heat map for all 24 samples when mapped to the Brahman reference and using all CpGs with ≥ 10X coverage in all samples. Darker colours denote lower correlation. **Right)** Correlation heat map for all 24 samples when mapped to the Angus reference using all CpGs with ≥ 10X coverage in all samples. Darker colours denote lower correlation. Dark blue denotes BTBT samples, cyan denotes BTBI samples, green denotes BIBT samples and orange denotes BIBI samples.


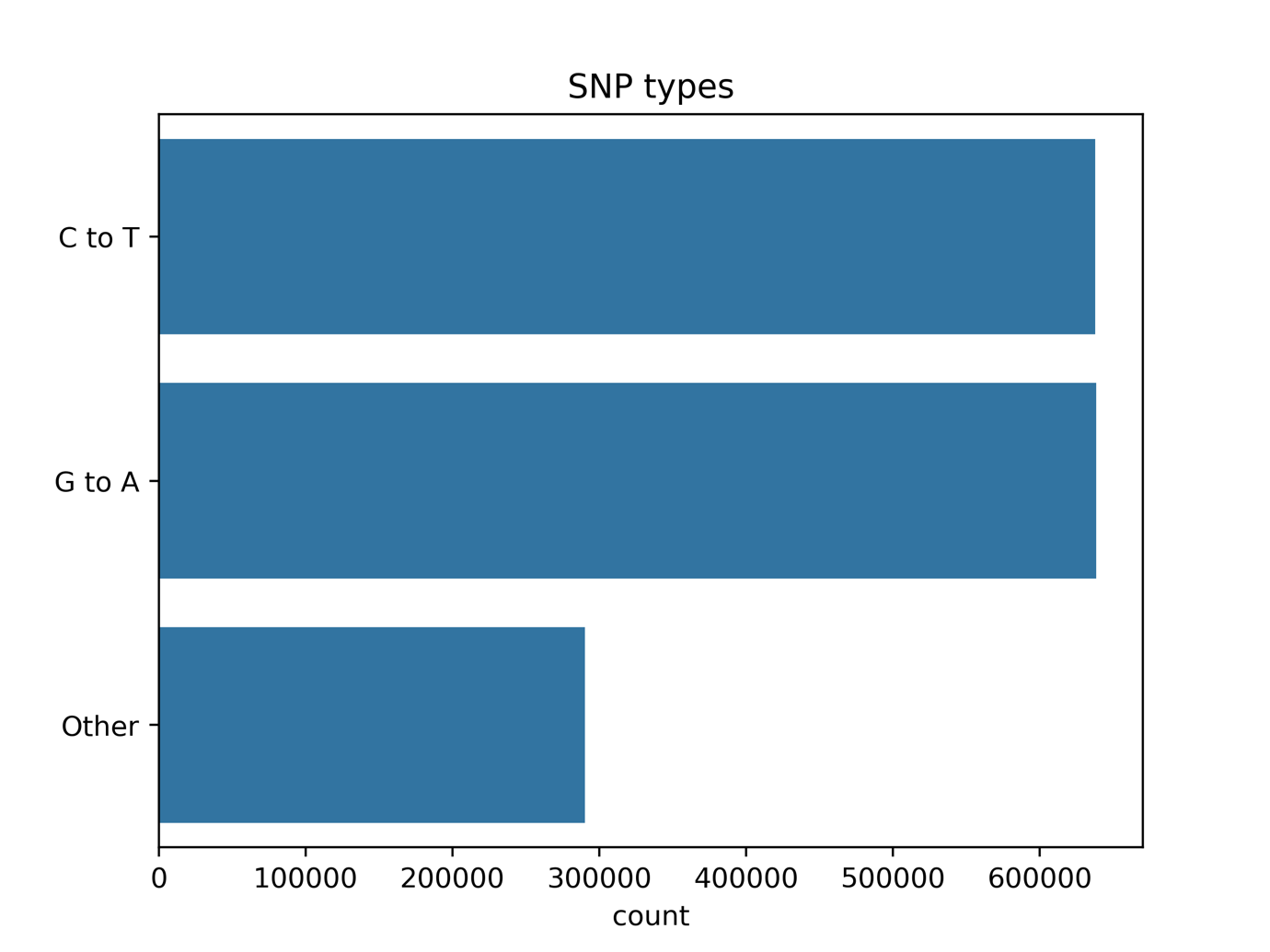


**Supplementary Figure 2**. **Number of C > T, G > A and other SNPs between Brahman and Angus reference genomes.** Horizontal bar graph showing the number of SNPs that affected a CpG site. Most (~81%) SNPs that occur in the CpG site are either C > T or G > A with other alterations making up ~19% of SNPs.


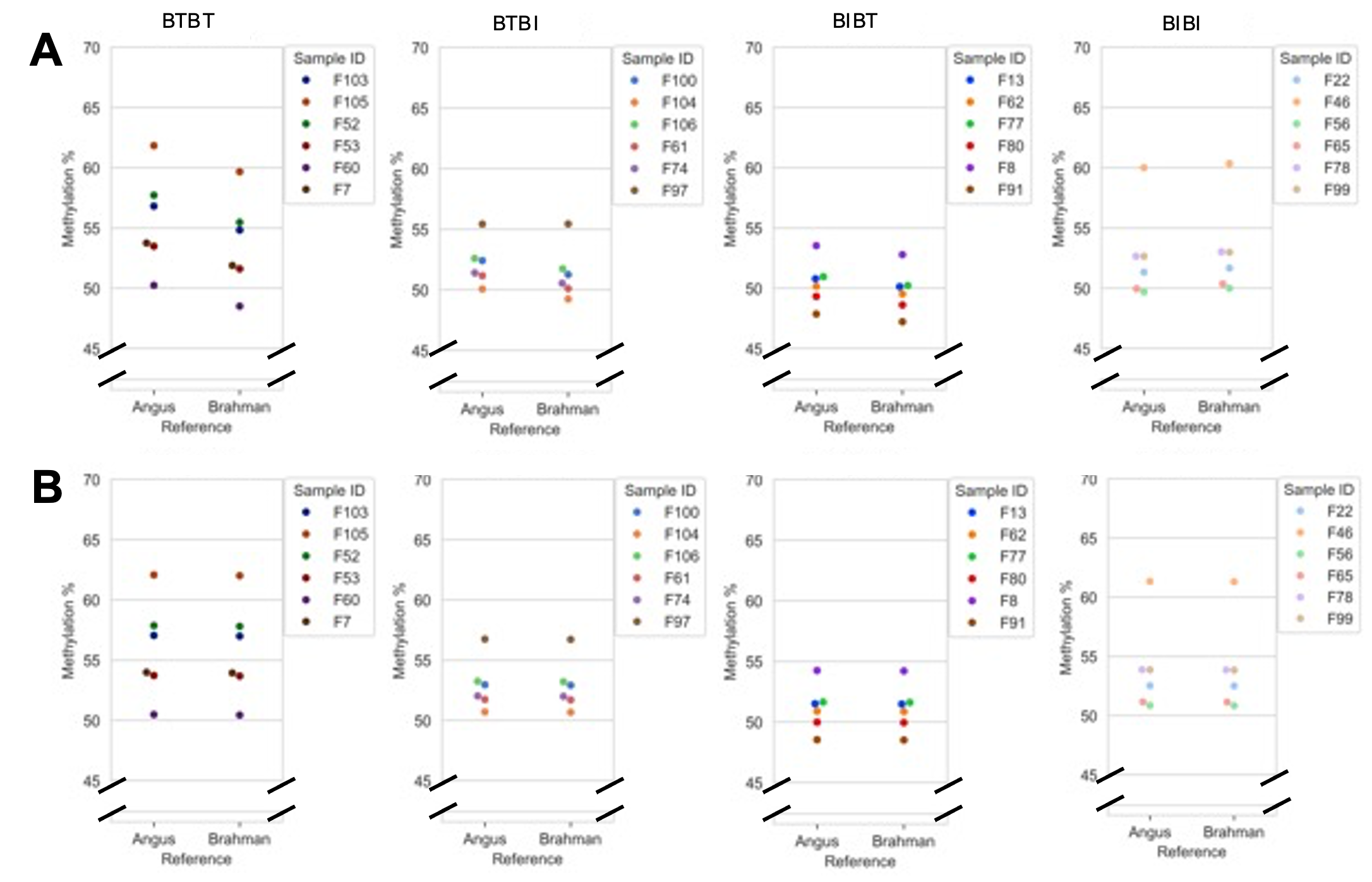


**Supplementary Figure 3. Comparison of global methylation per sample when mapped to Angus and Brahman reference genomes.**

**A)** Strip plot showing difference in global methylation between each sample when mapped to Angus vs Brahman reference genomes. Each sample is denoted by a different colour. Each panel represents one of the genetic groups in the order BTBT, BTBI, BIBT and BIBI. The X-axis denotes the reference genome and the Y-axis denotes the global methylation. **B)** same as **A** except only shared CpG sites were considered.


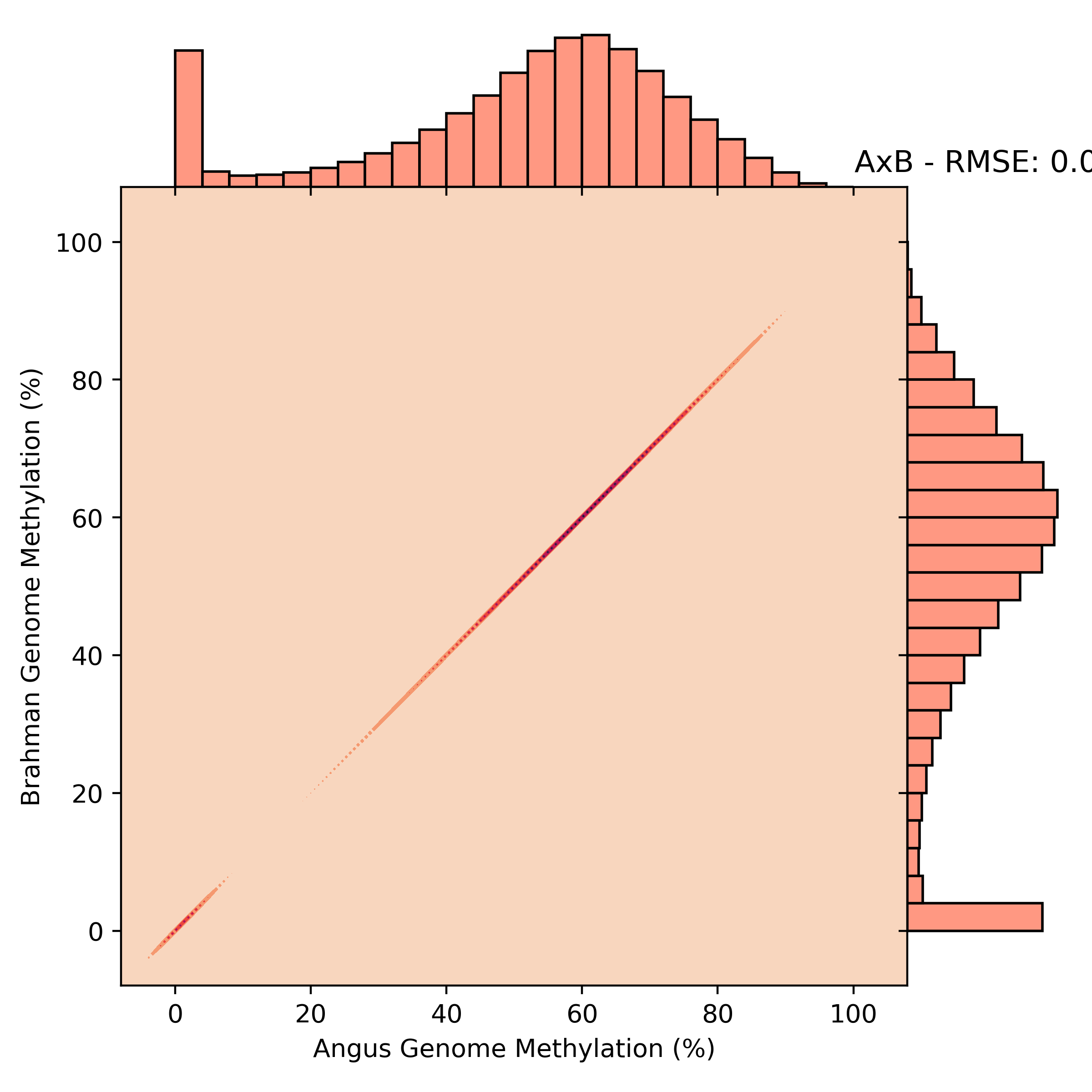

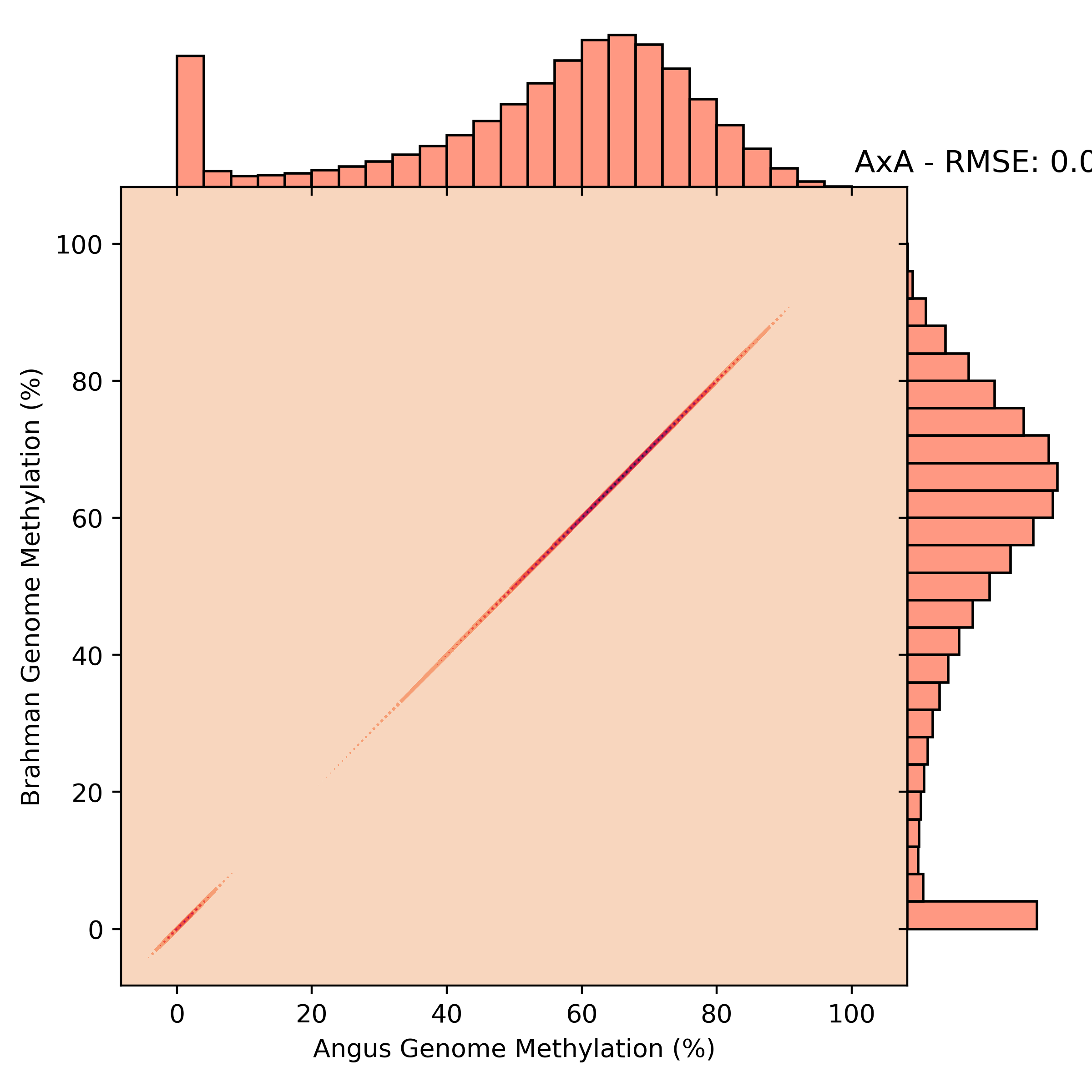

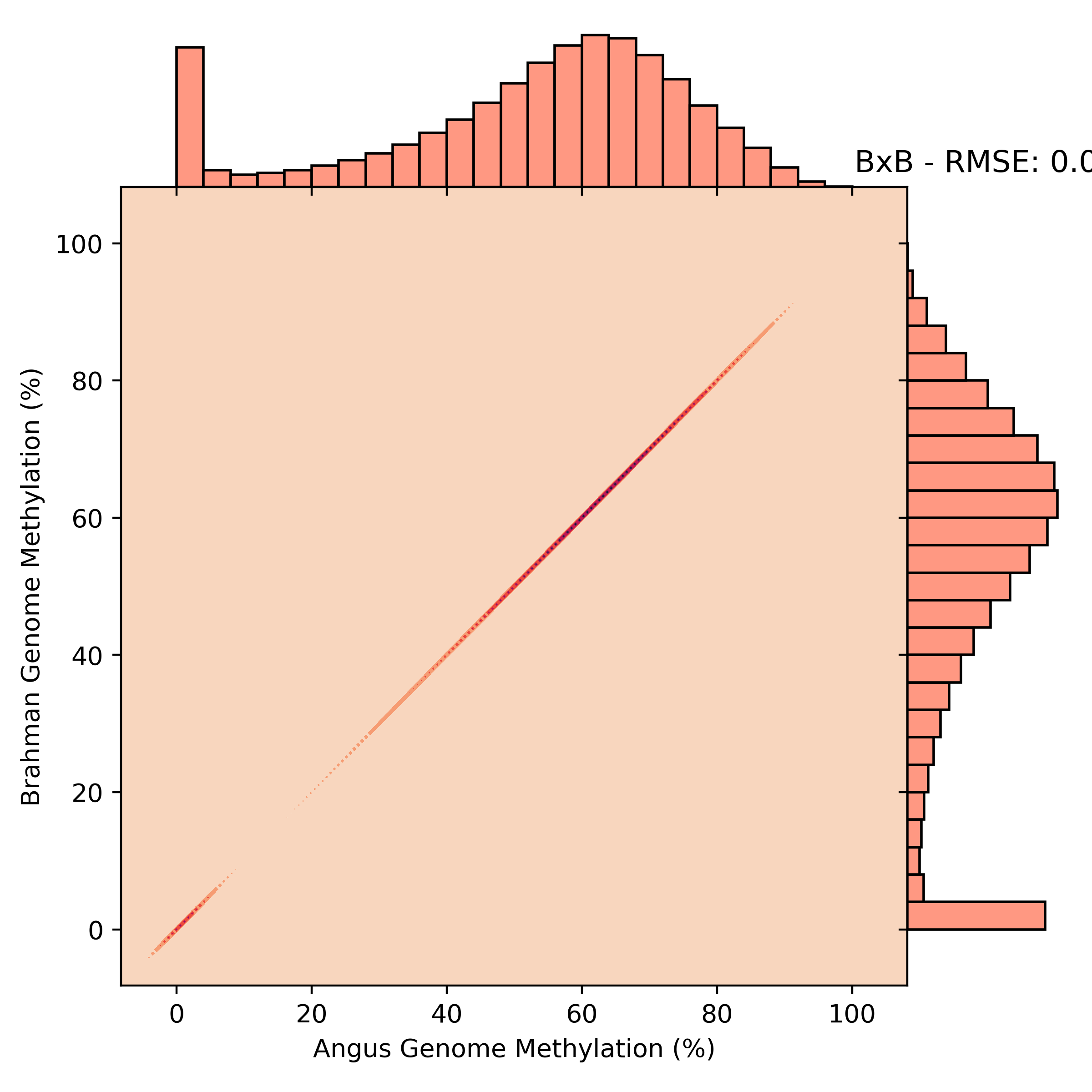

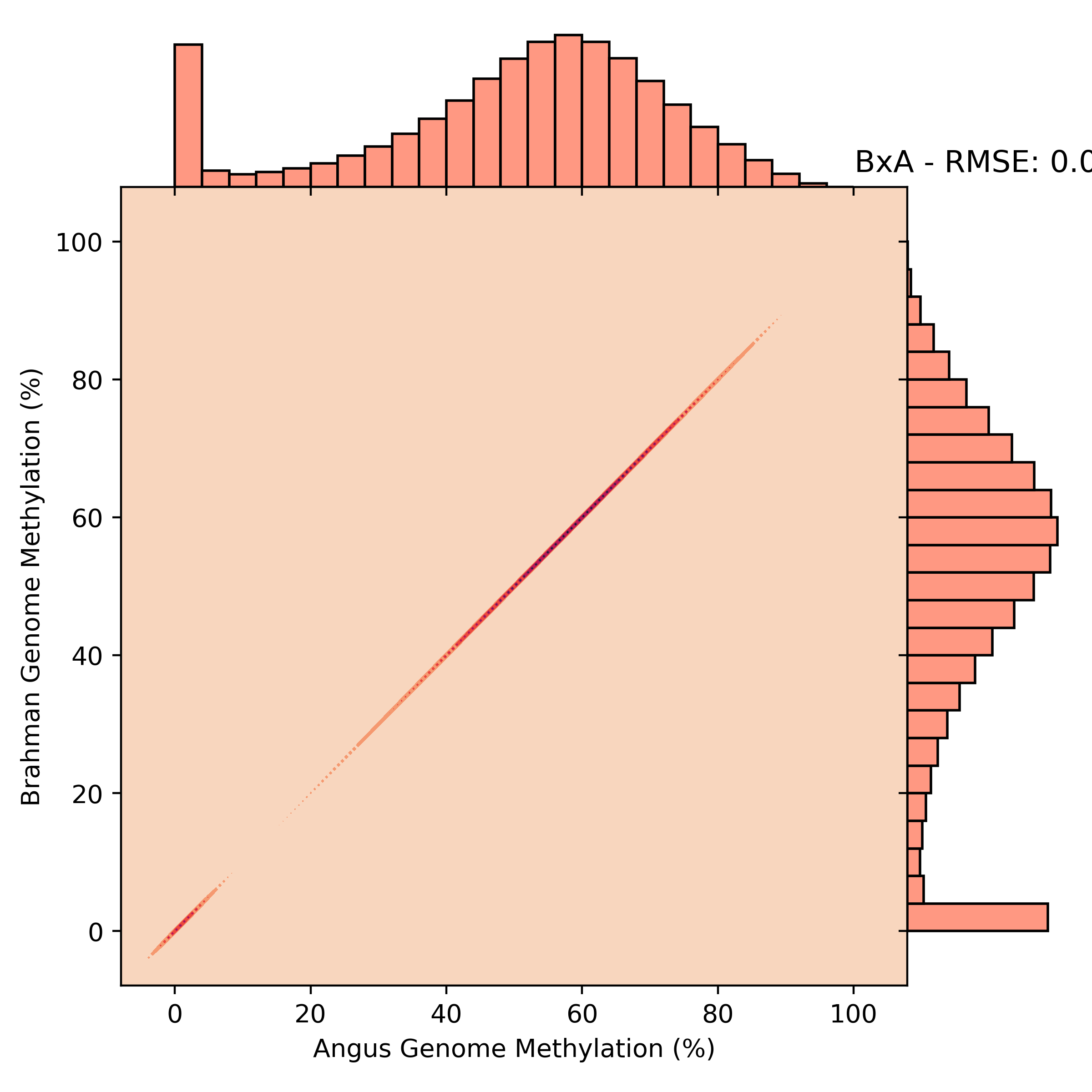


BIBI

BIBT

BTBI

BTBT

RMSE: 0.01

RMSE: 0.01

RMSE: 0.01

RMSE: 0.01

**Supplementary Figure 4. Two-dimensional KDE plots of shared CpG methylation variation.** KDE plots comparing methylation of a given CpG site when mapped to Angus (X-axis) compared to the methylation of that CpG site when mapped to Brahman (Y-axis). This is using all CpG sites that passed our filtering criteria.


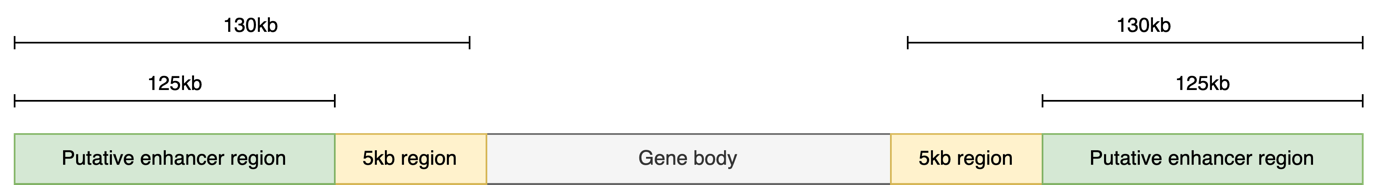


**Supplementary Figure 5. Graphical representation of search region.** For each feature labelled as “gene” in the GTF files for Brahman and Angus, we took 130kb either side of the gene body. The outermost region in green represents the putative enhancer regions; these were 125kb in length, regardless of the size of the gene body. Next were the 5kb regions, which extended 5kb up or downstream of the gene body. Lastly, the gene body was kept as the length that it was in the GTF file.
