# Supplementary material for: DNA methylation analysis to differentiate reference, breed, and parent-of-origin effects in the bovine pangenome era
Source: Gigascience. 2024 Oct 17;13:giae061. doi: 10.1093/gigascience/giae061 (PMC11484048; doi:10.1093/gigascience/giae061)
Supplement: giae061_Response_to_Reviewer_Comments_Original_Submission [file giae061_response_to_reviewer_comments_original_submission.pdf]

Author's Response To Reviewer Comments

GIGA-D-23-00314 Authors' responses to reviewers

Reviewer 1
In this study, the authors investigate the impact of reference genome choice on downstream epigenetic analysis. To explore this impact, the authors sequenced and analyzed the Whole Genome Bisulfite Sequencing (WGBS) data from two cattle breeds, Bos taurus taurus (BT) and Bos taurus indicus (BI), as well as their reciprocal cross offspring (BTBI, BIBT). Through the analysis, the authors found (1) up to 2% quantification bias in global methylation when using an incorrect reference genome. (2) SNPs and SVs were 8-fold and 1.13-fold higher in CpGs than in the background, respectively, with enrichment p-values both smaller than 5 * 10^-324. (3) There was little overlap between DMR and DEG surroundings regarding genomic coordinates. The first two findings emphasized the importance of choosing the correct reference genome. Overall, the research topic is important as it can reveal/emphasize the potential computational artifacts in epigenetic analyses, and the research design is clear and straightforward. However, we have several questions and comments to present regarding the manuscript.

Major points:
In the manuscript's title, the phrase "genetics of epigenetics" is employed to highlight the phenomenon that reference genome choice can impact methylation signals, potentially resulting in inaccurate findings. Nevertheless, this phrase is overly expansive and not entirely reflective of the content covered in this study. The term "genetics" typically implies a connection to inheritance, making the phrase more pertinent for research examining the genetic factors associated with/regulating DNA methylation, such as methylation Quantitative Trait Loci (meQTL) studies or genetics causality studies. On the other hand, though "genetics" denotes changes in DNA sequence, it usually refers to the variations in the individual genomes, not on the reference genomes. Therefore, to enhance clarity and specificity, it is recommended to revise the title to a more precise expression reflecting the paper's key message.
We appreciated the reviewer's comments regarding the clarity of the title and have altered it to "DNA methylation analysis to differentiate reference, breed and parent-of-origin effects in the bovine pangenome era".

In the section `Overview of DNA methylation patterns`, the authors summarize the methylation levels of different genomic regions using the global mean of methylation. This information is too limited. The authors should consider displaying the whole distribution of methylation levels using density plots or violin plots, which contain more information (mean, median, quantiles, and skewness). It is also suggested that the descriptive statistics of genomic regions be organized into a table.

Response: We appreciate the reviewers' comments regarding the methylation overview section. We agree that descriptive statistics such as mean, variance, skewness would be helpful and have added a supplementary table (S. Table 5). This table contains descriptive stats for each of the seven genomic regions (5'UTR, 3'UTR, enhancer, exon, intergenic, intron, promoter) among genetic groups (BTBT, BTBI, BIBT, BIBI) for Brahman and Angus reference genome.

However, we maintain the presentation of the mean methylation with slight changes in the main text figure (Figure 3A-B). Wulfridge et al. 2019 also presented global methylation differences between samples mapped to different genomes in similar way as we did in figure 3A-B. Additionally, to plot the distribution of all CpG sites per sample would result in a violin plot that spans from 0 – 100% methylation with the widest point around the global mean for that sample and would not be very informative in that way, which was why we adopted the approach of Wulfridge et al. 2019. We altered the figures to display each sample from each group and their median methylation level when mapped to both reference genomes. We performed a Wilcoxon rank sum test on each sample to compare the distribution of methylation between the two genomes and have reported the p-values in Table 3.

In the section `Enrichment of SNPs affecting CpG sites`, the p-value calculation is problematic. Based on the Jupyter Notebook code in the Github link of supplementary table 7, the enrichment significance is

computed by the following code: `stats.binomtest(k=(cpg_snps - cpg_snps_sv), n=total_snps, p=(total_snps / brahman_len), alternative='greater')`. Here, the authors assume the `p=total_snps / brahman_len` to be the background probability of having a mutation in the CpG sites. However, this does not seem right since the formula denotes that p is the probability of having a mutation in a single nucleotide, not for a CpG dinucleotide. Similarly, the SV's p-value calculation should be corrected as well. The authors should also explicitly write the enrichment analysis details in the method section.

Response: We thank the reviewers for taking the time to review the supplied code. When determining the probability that a CpG site was affected by a mutation, it is only necessary to consider single mutations, as any mutation of the 'C' alters the CpG site. Similarly, any mutation of the G also alters the CpG site. If both 'C' and 'G' in the CpG were mutated, we scored this as two SNPs occurring in this CpG site. Therefore, using the denominator SNP rates at single nucleotide level is appropriate as we effectively scored the CpG at single nucleotide level. For SV's p-value calculation, we used a different way to calculate enrichment of SVs. This essentially was a binomial test comparing CpG in SVs versus CpG not in SVs. The null hypothesis was CpG proportion was equal in SVs and non-SVs, which was rejected at $p < 0.001$. We have amended the methods to explicitly state these details of the enrichment analysis at lines 642-667.

"SNP and SV Enrichment
To determine whether CpGs were significantly impacted by SNPs, we identified all SNPs between Brahman and Angus that impacted a CpG site. As Brahman autosomes were used as the reference to minimap2, we used the coordinates of all CpGs within the Brahman genome to identify which SNPs in the VCF file were altering a CpG site. We determined the probability of a SNP occurring as the number of SNPs (n) identified between a given pair of autosomes divided by the length of the autosome (l), P("SNP_occurring") = n/l. As a CpG site only needs a single SNP to destroy it, one need only consider the probability of a single base changing, I.e., either the 'C' or the 'G'. To test whether CpGs were significantly more likely to be affected by a SNP, we performed a binomial test for each autosome where k was equal to the number of SNPs affecting a CpG site, n was the number of SNPs that occurred, and P was the probability of a SNP occurring.

To assess whether CpGs were significantly enriched within SVs identified between the two genomes, we counted the number of CpG dinucleotides occurring within SV sequences and compared that against the number of CpGs that occurred in non-SV regions. To determine the probability of a CpG occurring outside an SV, we first identified the SV coordinates from the VCF file produced by PAFtools and constructed a bed file of SVs for each reference genome. Next, we used BEDTools [104] to generate coordinates of the complementary regions, i.e., the non-SV regions of each genome. We then extracted the fasta sequence of these regions for each genome. We then counted the number of CpGs that occurred in these regions and divided them by the combined length of each region to determine the probability of a CpG occurring in the non-SV portion of the genome. We then performed a binomial test to determine if CpGs were more likely to occur within an SV than non-SV regions."

Additionally, as the SNPs between reference genomes are estimated using MUMmer, whose results are not guaranteed to be perfect, some validations of MUMer output accuracy are needed. Such validations are essential for this study because if MUMer outputs inaccurate SNP results, the background mutation probability will be distorted, leading to imprecise enrichment p-values. MUMer's accuracy can be obtained from the literature if there is any, or evaluated by the authors themselves (e.g., supplying two copies of the same input reference genome to MUMmer, any SNP found should be false positive; and supplying a reference genome and a variation of the reference genome with bases randomly mutated at a given rate, and evaluate if the software can accurately recovers the mutation frequency).

Response: We appreciate the reviewer's comments about the potential unreliability of MUMmer. We tested the evaluated how well MUMmer is able to identify SNPs and found it to be poor. We evaluated Minimap2 as an alternative to identify SNPs and found this to be far superior (Supplementary Table 13). The steps of this evaluation have been added to the methods at lines 616-640. We then reanalysed all related sections using SNPs identified by Minimap2 and PAFtools.js.

"Identification of SNPs and SVs between genomes
To determine the accuracy of minimap2 in identifying SNPs between the Brahman and Angus genomes, we

first introduced artificial mutations into each genome using SNP Mutator [103]. The Angus and Brahman genomes have previously been reported to differ by ~1% [39]. Therefore, to determine how well minimap2 can detect SNPs between sequences that are divergent by ~1%, we first simulated mutations in each autosome for both species. For example, chromosome 1 in the Angus reference genome is 157,005,132 bp long, so we set the number of substitutions to 1,570,051 SNPs. In addition, the random seed was set to 12, and the number of times each autosome was mutated was set to 1. We repeated this for all autosomes in the Brahman and Angus genomes, adjusting the number of SNPs to maintain the 1% divergence in each autosome. We then mapped the mutated autosomal sequence to the original sequence for each autosome and breed, giving us 58 "replicates". Minimap2 [59] and PAFtools were used to identify variants between the original and mutated sequences. The minimap2 mapping parameters used were '-x asm10, -c, --cs', followed by PAFtools 'call —f', where the file provided to the —f argument was the original, unmutated autosomal sequence. Minimap2 and PAFtools showed a mean accuracy of ~99% across the 58 autosomes, suggesting a good ability to identify SNPs between the two breeds (S. Table 13). We then aligned each autosome from Brahman to each autosome from Angus, using minimap2 with the parameters '-cx asm10' and '—cs'. The output from minimap2 was then used as input to paftools.js call with the parameter '-f ', where reference_autosome.fa refers to the autosome that was supplied first to minimap2, i.e. the reference sequence, not the query. We then used the output VCF files to determine the SNP and SVs between the two genomes."

The authors found that over 80% of CpG SNP were either C2T or G2A. Although a potential explanation from the spontaneous deamination perspective is provided, the ratio still looks high. The authors would need to provide additional support for this finding, e.g., theoretical support where probabilistic calculations show the high ratio falls into a certain confidence interval, or empirical support where a different method yields a similar high ratio in other mammals. Having either support would eliminate the concern that this high ratio reflects computational artifacts.

Response: We appreciate the reviewers concerns and have included a reference to a human study that found similarly high levels of spontaneous deamination in SNPs affecting CpG sites. This can be found at line 241-243, which is as follows: "Looking more closely at the CpG SNP changes, we found that most (~81%) of the CpG SNP were either C to T or G to A changes (S. figure 9), which is very similar to the number of CpG SNP changes detected in humans (80.7%) [60]" .

The section `Choice of reference genome influences methylome results` is the primary section supporting the manuscript's main conclusion; there is room for improvement, and more details should be provided to support the conclusion better. Specifically,
The authors used boxplots to present the overall CpG methylation difference caused by reference choices. The boxplot can be improved. Raw data points should be shown on the plot, with a line connecting the data point between groups, indicating they are the same biological sample but used different reference genomes. It is also advised to report the exact p-value in the figure, not just the significance symbol. Besides, it is better to compare the whole distribution, not just the global mean, as also denoted in previous comments.

Response: We believe we have addressed this in the revised Figure 3, which now includes mean global methylation for each sample per genetic group and partitioned by reference genomes used. As per our first response to comment 2 above, we prefer to show mean global methylation following Wulfridge et al. 2019. We have included the mean, variance and p-value of different genetic group comparisons in S Table 8. Additionally, we have added how the p-values presented in Table 3 were determined at lines 669-684.

"Determining quantification bias between genomes
To determine whether there was a significant quantification bias between the Brahman and Angus reference genomes for a given sample, we compared the vector of all CpG sites with at least 10X coverage when mapped to Angus in sample I against the vector of all CpG sites with at least 10X coverage when mapped to Brahman. To ensure the vectors were equal, we randomly subset the larger vector to be the same length as the smaller, i.e., if the Brahman reference genome had more CpG sites with 10X coverage for that sample, the Brahman vector was randomly subset to match the number of CpG sites in the Angus vector for that sample. We repeated this for all samples using all CpGs and again with just the CpG sites marked as shared (Table 3; S. table 8). We then pooled all CpG sites for each sample within a group, e.g.

all samples from BTBT, and determined whether the CpG methylation differed significantly for that group when mapped to Angus and Brahman. The p-value was determined using a Wilcoxon Rank Sum test and adjusted for multiple testing using the Benjamini-Hochberg procedure. We identified variable CpGs by matching the shared CpGs between reference genomes and then identifying those with an absolute methylation difference greater than 10%."

Only the global methylation of a sample is presented, which is too abstract to be practically useful. Since most CpG sites have methylation levels close to 0 or 1, the global methylation change may only represent different compositions of CpG sites in the two reference genomes. It may not be related to how methylation levels would change on the site level. The site level methylation is more worthwhile to examine than the global methylation as it is the basis of epigenetic analysis; if there is no change on the shared sites (which is the majority of sites) no matter which reference genome is used, then the significance of this manuscript's conclusion is greatly diminished. Therefore, It is highly suggested to focus on the site level methylation analysis, e.g., plot the scatter plot/2d-density plot where the x-axis is the methylation level using one reference genome, and the y-axis is the methylation level using another reference genome, each dot is the shared CpG sites in 2 references, and summarize the similarity using correlation/R square, Rooted Mean Squared Error (RMSE), etc.

To follow up on bullet point b, the authors can also examine what specific CpG sites have relatively large deviations in methylation level when incorrect reference is used and explore the potential reason and consequence for the difference from genomic and biological pathway perspectives. Another good side for this disentangling is that this would also yield a set of confidence sites (sites not sensitive to the reference choice) and variable sites (sites sensitive to the reference choice). If the variable sites account for a large proportion of all sites, this would be strong evidence for the manuscript's conclusion. The disentangling would also be helpful to the bovine research community (say whether a published study used the correct reference genome is unclear, how much of their conclusion and what proportion of their findings in the epigenetic analysis should be trusted? Is it necessary to reanalyze everything?)

Response: The authors thank the reviewers for their comments in 6b and 6c. As a summary of the overall differences among groups when using different reference genomes, we believe global methylation is useful similar to Wulfridge et al 2019's DNA methylation quantification bias in mice. We have included Figure 3C and provided a table to summarize the number of variable CpG sites (S. table 9). From Figure 3C, even among shared CpG sites, there are sites that deviate from the diagonal, which indicate reference genome effects. The shared CpG affected by reference genome effect is 264,023 sites or 1.87%. This is based on using an absolute methylation difference threshold of 10% to call the CpG sites as variable because a difference of 10% could have biological effect is based on Leenen et al., 2016 and Thomson et al., 2022. Although the proportion of shared CpG sites affected by reference genome effect is small, the breed specific CpG sites is ~1.1 million, which is substantial and hence, a pangenome approach is needed to capture the methylation level. We provided a Supplementary table 9 of variable CpG sites for the bovine research community. We have added to the discussion to address this at lines 459-464. "Moreover, we demonstrated a number of CpG sites that appear to be sensitive to reference genome choice, differing by more than 10% between the two genomes. It is likely that these CpG sites would confound downstream analysis, especially if they overlap with regions of interest such as cis-regulatory elements. Thus, this demonstrates that even by attempting to control for reference genome differences, quantification bias can remain."

It is hard to guess what the DMR analysis is about in the section `Choice of reference genome influences methylome results` (lines 260-262). A more precise explanation of the DMR analysis design is needed. Besides, more research should investigate the reference genome's influence on downstream DMR analysis. Some directions include (1) Intra-breed perspective: For the samples in the same breed, if their data are aligned to 2 reference genomes forming 2 groups, will these 2 groups have DMR identified on the shared CpG regions? (2) Inter-breed perspective: For analyzing the DMR when comparing one breed (BTBT) to another breed (BIBI), there are 4 possibilities in total (1 correct case: BTBT used BT reference, BIBI used BI reference; and 3 incorrect cases where the matching between samples and reference genomes is not exact). How will the DMR results differ between the correct case and incorrect cases? The Venn diagram is suggested to show the concordance of DMR results in different comparison scenarios.

Response: We agree that the section 'Identification of differentially methylated regions' is not clear at describing how the DMR analysis was carried out and what models were fitted. We have now included the design matrices of all fitted models as part of Supplementary table 14. Additionally, we have rearranged the paragraph in this section to improve clarity lines 686-728 (also pasted below). There were 6 models: 2 for breed effect, 2 for maternal effect and 2 for paternal effect. These were all logistic regression models and sex was always fitted as a covariate. The convention in differential methylation analysis and likewise, gene expression analysis with RNA-seq, is to choose a single reference genome to start the analysis. As such, to our knowledge, no tool can compare the impact of same samples called on different reference genomes. For example, the reviewers mentioned 1 correct and 4 incorrect cases: A) BTBT on Angus ref vs BIBI on Angus ref, B) BTBT on Angus ref vs BIBI on Brahman ref, C) BTBT on Brahman ref vs BIBI on Angus ref, and D) BTBT on Brahman ref vs BIBI on Brahman ref. The correct case is B but it is impossible to do with existing tools. We could do case A and D.

"Identification of differentially methylated regions
The methylKit package (v. 1.22.0) [77] was used to identify DMRs between breed and POE groups. We investigated breed effects by comparing BIBI samples with BTBT samples, maternal effects by comparing samples with BIBI dams (BIBI; BTBI) and those with BTBT dams (BTBT; BIBT) and paternal effects by comparing samples with BIBI sires (BIBI; BIBT) to those with BTBT sires (BTBT; BTBI) (S. table 14). The reference group was always the breed that matched the reference genome. For example, when BIBI and BTBT WGBS reads were aligned to the Brahman reference genome, BIBI samples were treated as the control group and BTBT as the treatment group.
We followed the pipeline described by the methylKit authors for DMR analysis [77]. Briefly, we only considered CpGs that were identified as shared. We then removed all CpG sites with less than 10X coverage and more than the 99.9th percentile of coverage. Reads with too high coverage (e.g. from PCR duplication bias) can impair the accurate determination of the methylation percentage at that site and is a recommended pre-processing step for methylKit [77]. We then normalized the coverage using the default methylKit normalization strategy. We merged the CpG counts per group using the 'unite' function with 'destrand = T' and 'min.per.group = 5L' so that a given CpG site had to be covered by at least ten reads in five out of six samples per group. For the parent of origin DMR analyses, we set 'min.per.group = 10L'.
We then identified differentially methylated cytosines between groups using the 'calculateDiffMeth' function, with sex as a covariate in the model. To determine differentially methylated regions, we used the 'tileMethylCounts' function with default parameters to divide the genome into regions for differential methylation analysis. This step allowed methylKit to divide the genome into non-overlapping regions based on the tiling windows. MethylKit then models the methylation at a given cytosine or region by fitting a logistic regression:

$$\log(P_i/(1-P_i))=\beta_0+\beta_1*T_i+a_{\text{"sex"}} * \text{"Sex"}_i$$

Pi denotes the methylation proportion for sample i in samples 1,…,n, where n is the number of samples across both groups in the comparison [77]. Ti represents the groups (0 for control, 1 for treatment). β_0 denotes the log odds of the control group (fraction of reads reporting C / 1 – the fraction of reads reporting C). β_1 denotes the log odds ratio between the control and treatment. $\alpha_{\text{"sex"}}$ denotes the parameter for the sex covariate and "Sex"_i denotes the sex (0 = male; 1 = female) for sample i. For further details, refer to Akalin, Kormaksson [77]. This design resulted in six different logistic models being fit: model 1A (breed comparison when aligned to the Angus reference), model 1B (breed comparison when aligned to the Brahman reference), model 2A (dam of origin comparison when aligned to the Angus reference) model 2B (dam of origin when aligned to the Brahman reference), model 3A (sire of origin when aligned to the Angus reference) and model 3B (sire of origin when aligned to the Brahman reference) (S. table 14). Any DMRs identified were either hypo- or hypermethylated with respect to the control group. We retained all DMRs with a difference in methylation of ≥ 10% and a qvalue of ≤ 0.01 for further analysis. An overview of the samples, reference genomes, types of CpGs and DMR analysis is given in Figure 1A-G."
In the section `Breed-specific CpGs show distinct methylation patterns`, the threshold choice of defining methylated (>0.65) and unmethylated (<0.35) should be justified. Is the threshold based on the data distribution or based on subjective choice? What is the rationale behind it? Do the shared CpGs also show a distinct methylation pattern similar to the breed-specific CpGs when different reference genomes are used?

Response: We have performed this analysis with the 35 and 65% thresholds as well as at 25 and 75%. We observed much the same pattern in both scenarios and have updated the manuscript at lines 304-306

(“We repeated this with “unmethylated” thresholds of ≤ 25% and “methylated” thresholds of ≥ 75%, with the CpG methylation values in between being considered hemimethylated [61].”) and at lines 309-315 (“At both the 25 and 75%, and 35 and 65% thresholds, we observed significantly more Brahman-specific CpGs as hypomethylated than hypermethylated (Mann-Whitney U-test, p = 2.5 × 10-34) (Mann-Whitney U-test, p = 2.5 × 10-34), respectively Interestingly, we observed the inverse when considering Angus-specific CpGs; significantly more Angus-specific CpGs were hypermethylated than hypomethylated (Mann-Whitney U-test, p = 3.0 × 10-34) (Mann-Whitney U-test, p = 2.5 × 10-34), respectively (Figure 3D-E).”)

We demonstrated at lines 266-269 that the shared CpGs tend to follow the same methylation pattern regardless of the genome used. However, there are some that still differ substantially (lines 282-285).

In the section `Breed-specific DMRs show poor association with DEGs`, the authors found that a small portion (~20%) of DMRs overlapped with DEG surroundings when comparing breeds. The authors should also report the portion of DEG whose surroundings overlapped with DMR. If both numbers are small, there will be concerns about whether such discordance is due to intrinsic biological or other technical reasons. For example, we noticed that in the DMR analysis, the authors controlled the sex effect when comparing the breed difference. While in the DEG analysis, the authors did not mention if they correct sex effects or not. Nevertheless, given that the RNA-seq and WGBS data are sequenced at different time points with different sequencing platforms, some sanity checks should be performed to ensure the group differences presented in both data are mainly the breed differences. Otherwise, the overlap analysis is pointless. To solve this problem, the authors can consider quantifying how much of the variance in transcriptome can be explained by the variance in the methylome (compute the canonical correlation between these 2 data modalities); alternatively, the authors can perform pathway enrichment analysis for both the DMR and DEG respectively and confirm if the differential analysis results are concordant in the pathway levels despite their difference in the genomic coordinates.

Response: We have added the number of DEGs that overlapped with a DMR at lines 331-332, 334-336, 356-357, 364-366, 376-378 and 378-380 (see below for responses). The number of DEGs that overlapped with a DMR was high, >88%. Given this result, we have re-written the title of this Method’s section as “DMRs between breeds show limited overlap with DEG promoters” and modify our results to emphasize that DMRs are near or in gene bodies of DEG but showed poor overlap with promoters. We have fitted sex as a covariate in both methylation and gene expression (RNA-seq) analyses. We appreciate the reviewers’ comments and concerns around how much variance in methylation explains the variation gene expression. To address this, we performed canonical cross correlation analysis, as suggested, to determine the correlation between the RNA-seq and methylation data. We used DESeq2 to transform the RNA-seq counts data into a more appropriate format for CCA. We found that the R2 between the two datasets is 0.18, which is a moderate correlation; we have added this at lines 769-771. Given that methylome is only one of the factors influencing gene expression, this result is not surprising.

“Furthermore, we performed canonical correlation analysis (CCA) to estimate the coefficient of determination between gene expression and DNA methylation, R2 = 0.18, suggesting a moderate relationship between gene expression and DNA methylation.”

Furthermore, to avoid confusion with the DEG results presented, we reanalysed the DEGs using DESeq2. The model formula given to DESeq2 was `~0 + Genetics + Sex + Batch`. We have updated the methods at lines 762-763.

Lines 331-332: Only 0.3% of the DMRs overlapped with promoters of DEGs, despite 99% of significant DEGs being overlapped by a DMR.

Lines 334-336: Most (~73%) of these DMRs fell into the putative enhancer region, while only 0.3% of the DMRs overlapped with a DEG promoter, despite substantially more (1,872) DEGs observed (S. table 10) and 99% overlapping with a DMR.

Lines 356-357: Around 6% (1,236) of the DMRs overlapped with DEGs with ~78% of DEGs being overlapped by a DMR.

Lines 364-366: Using Angus as the reference, we observed 1,254 (~5%) DMRs overlap with the 358 DEGs and ~88% of DEGs covered by a DMR.

Lines 376-378: Around 6% (1,236) of the DMRs overlapped with DEGs with ~78% of DEGs being overlapped by a DMR.

Lines 378-380: Using the Angus reference, ~19% (~12,251) of DMRs overlapped a DEG and ~96% of DEGs overlapped with a DMR; ~75% of the DMRs overlapped a putative enhancer region.

In the discussion (lines 431-433), the authors explained the potential reason why there is no directional change found when considering DMC, but there are direction changes when considering DMRs. The authors reasoned that the cause might be that some DMRs are specific to the reference genome. However, this explanation is not as convincing. We think it might be more plausible that the number of tests in the DMC analysis is way larger than the number of tests in the DMR analysis. As a result, when correcting p-values for multiple hypothesis testing, the threshold will be more stringent in DMC analysis than that of DMR analysis, rendering no Cytoscine pass that threshold. The authors should explicitly write what p-value adjustment method is used or justify if the p-value correction method is suitable for the problem setting.

Response: We appreciate the reviewers' comments regarding the DMR inversion results. We reported no p-values for this result. The point of this section was to illustrate that when using a tiling method as part of DMR identification, the influence of breed-specific CpGs will likely play a role in determining methylation status of that region. For example, methylKit examines the number of Cs reported, the number of Ts reported and the overall coverage at that region. Using a tiling approach (which MethylKit does), it is likely that the CpGs affected by SNPs will be included in these regions. Therefore, some positions will be erroneously reported as unmethylated (high portion of Ts) in the non-reference genome group. We have better explained this reasoning in the discussion at lines 456-459.

Minor points:
The current analysis is purely based on real data, where the underlying true genome responsible for generating the WGBS data remains unidentified. For a comprehensive understanding, validating the conclusion through in-silico simulations is suggested. This involves creating synthetic bisulfite sequencing reads, simulating from BT and BI reference genomes to represent BTBT and BIBI samples, respectively (reciprocal cross-offspring samples can be fabricated via the in-silico mixture of synthetic BTBT and BIBI samples). These synthetic reads should then be aligned to the BT and BI reference genomes, respectively. In such simulations, the reference genome serves as the known true genome. Consequently, when comparing downstream analysis results of a particular sample where different reference genomes are used, any observed discrepancies can be attributed to the differences in the reference genomes. This controlled simulation provides a clean environment to establish a theoretical limit on the accuracy attainable when employing the correct or incorrect reference genome, thereby revealing the true significance of selecting the appropriate reference genome. On the other hand, since the genomic origins of the synthetic reads are known, it offers an opportunity to investigate what specific factors (SNPs, SVs, repetitive elements or telomere regions, etc.) in the reference genome and how they contribute to the observed discrepancy.
The authors appreciate this suggestion but argue this would be an entirely new study in itself and is beyond the scope of this work. Moreover, the authors suggest a more appropriate alternative would be to employ long-read sequencing technologies like Oxford Nanopore, where we are able to generate and assemble the true genome of each individual and identify DNA methylation without the risk of DNA damage that can occur using bisulfite based methods.

For the parent-of-origin effects analysis (lines 589-593), the maternal effects are investigated by comparing samples with BIBI dams (BIBI; BTBI) and those with BTBT dams (BTBT, BIBT); the parental effects are investigated in a similar way. The comparison design has an underlying linear assumption that the parental and maternal chromosomes of different breeds have no synergy effects. Under that assumption, when comparing samples with BIBI dams (BIBI; BTBI) and those with BTBT dams (BTBT, BIBT), the parental effects are averaged out, thus all the differences can be attributed to the maternal effects. This assumption should be acceptable in general, but we think a better idea to bypass the assumption is to construct in-silico mixture samples where half of the sequencing reads are sampled from

BTBT and half from BIBI; in this scenario, the methylation profile is equally contributed by BT and BI breed (the null case when the parental and maternal effects do not exist). Then, the maternal effects can be investigated by comparing the BTBI to the in silico samples, and the parental effects can be investigated by comparing the BIBT samples to in silico samples. This suggestion is not required to be implemented since it is not the main focus of the manuscript, but it would be interesting to explore.
The authors thank the reviewers for this suggestion and agree it would be interesting to explore. However, as with the previous suggestion, we consider it to be beyond the scope of this study and suggest it would be more appropriate to investigate with long-read sequencing technologies where the paternal and maternal genomes can be more easily disentangled.

For CpG sites filtering (line 594), the authors mentioned the sites were excluded if their coverage are greater than the 99.9th percentile and reasoned that the extremely high coverage is due to the PCR duplicates. However, since the authors already marked the PCR duplicates in the WGBS data processing, the high coverage should no longer be due to PCR duplicates. We speculate it might be due to the repetitive nature of some regions in the genome (or alignment artifacts). Perhaps the authors can check if the genomic coordinates of these extremely high coverage sites are consistent across all the samples in the same breed (if it is due to PCR duplicates, these coordinates might be more stochastic; if it is the nature of the reference genome, then the coordinates might be more enriched/restricted to certain regions)
While the filter for coverage greater than 99.9th percentile may not be due to PCR duplicates, this step is recommended by the authors of MethylKit. We have clarified this in the methods at lines 832-833 ("... site and is a recommend pre-processing step for methylKit [77].")

In supplementary table 1, the WGBS mapping stats should contain more information about the read mapping summary for each sample, e.g., the total number of reads, mapped reads (%), and mapped reads after marking duplicates (%), etc.
We have updated supplementary table 1 to include the total number of reads and reads mapped after deduplicating.

Word suggestions:
In the subtitle `Breed-specific DMRs show poor association with DEGs`, the term `Breed-specific` is not an accurate expression for what it tries to describe. The term breed-specific means something exists in one breed but not in another, e.g. (the authors used `Breed-specific CpGs` to denote CpG sites in one breed, but not in another in the previous section). It looks like the `Breed-specific` is abused here, where it actually means the regions or genes exist in both breeds, but they are different in breeds. In that sense, the authors could consider changing it to something like `breed-informative` or `breed-distinguishing` or the equivalent.
We have rewritten this header as "DMRs between breeds show limited overlap with DEG promoters".

We also suggest replacing all `poor association` with `poor overlap` since the association between DMR and DEG is never examined; only the overlap is examined.
We have addressed this in our previous response above.

In the Figure 2B caption, the sentence `The X axis is the first dimension of the logFC, and the Y axis is the second dimension of the logFC` might be inaccurate; should the logFC be logCPM? If not, the authors should explain the definition of logFC.
The authors appreciate the reviewers highlighting this. We have corrected it to logCPM.
Reviewer 2
This manuscript is a well-written and readable account of a study of methylation in two subspecies of cattle - Bos taurus and Bos Indicus. There is a specific focus on the effect of which reference genome assembly is used as the framework for the analyses. The authors demonstrate that the reference genome used can introduce a bias, albeit a relatively modest bias in global terms. Whilst the 2% bias can be considered modest at a whole genome scale, it can be critical when considering individual genes or regulatory sequences.
The average mapping rate of CpG with at least 10% coverage to the Brahman and Angus reference assemblies is reported in S Table 1 (94.95% and 92.49%, respectively), but only the average mapping rate to the Brahman assembly is reported in the main text.

Interestingly, a higher proportion of the Angus (Bos taurus) CpGs with at least 10x coverage map to the Brahman assembly than to the Angus assembly (94.83% and 92.75% respectively on average). In contrast, as one might predict a smaller proportion of the Brahman CpGs with at least 10x coverage map to the Angus assembly than to the Brahman assembly. Some comment on the apparent anomaly in the mapping rate for the Angus reads might be appropriate.
The Angus lacks X chromosome due to the use of trio binning genome assembly method. As a result, reads that belonged to the X chromosome were not mapped. Our analysis focused on the autosomes, hence the sex chromosomes impact was not included.

The authors highlight the bias that may be introduced by using an inappropriate assembly as the framework for epigenetic and other analyses. The coming pangenomes are presented as a potential solution without demonstrating that solution in this particular study. Perhaps a comment on other solutions such as the generation of experimental population specific genome assemblies as a framework for analyses. What will be the impact of the use of long read sequencing data that includes scoring of modified bases in epigenetic analyses?
We have added this to the discussion at lines 468-475. "While DNA methylation analyses between breeds and strains are challenging, exciting solutions are on the horizon. Indeed, with long-read sequencing like Oxford Nanopore becoming more cost-effective and given it has the ability to capture DNA methylation with no additional sample preparation, it is much more likely that researchers will be able to follow the recommendations of Wulfridge, Langmead [15] and examine DNA methylation of individuals with personalised reference genomes. Long read sequencing has the potential to simplify DNA methylation analyses of diverse populations substantially."

The manuscript is suitable for publication after minor changes.

Given the small number of individuals used in this study the term "breed-specific" should be used with caution. Perhaps the term and its usage in the paper should be explained, complete with caveats, in the introductory text.
We have added this disclaimer at lines 221-224. "551 noted that in this study, we consider breed-specific as CpGs that appear in one reference genome and not the other. The limitation of this being neither reference genome likely captures all variation present within the two breeds."

Historically, a sample size of 20-25 UNRELATED individuals was considered an appropriate sample of a population in breed genetic diversity studies.
The number of individuals sampled per breed or crossbreeds in this study is a fraction of this recommended sample size. Moreover, no information is provided on the degree of relatedness between the individuals sampled.
We appreciate the reviewer's comments regarding sample size. Taurine and indicine cattle are known to have high phenotypic and genotypic divergence even when sample sizes are small. Moreover, we based our sample size of 3 males and 3 females per group on Liu et al., 2021. We have added information regarding the relatedness of the individuals at line 551-552 (". Fetuses were sired by three BTBT bulls and 2 BIBI bulls.").

The WGBS and transcriptomics data used for the analyses are available in the public domain at PRJNA626458.

The gene symbol nomenclature for cattle follows the nomenclature style used for human genes. Thus, all gene symbols should be fully capitalised as well as italicised.
We have amended the manuscript accordingly.
