# Supplementary material for: DNA methylation analysis to differentiate reference, breed, and parent-of-origin effects in the bovine pangenome era
Source: Gigascience. 2024 Oct 17;13:giae061. doi: 10.1093/gigascience/giae061 (PMC11484048; doi:10.1093/gigascience/giae061)
Supplement: giae061_Response_to_Reviewer_Comments_Revision_1 [file giae061_response_to_reviewer_comments_revision_1.pdf]

Author's Response To Reviewer Comments

PLEASE NOTE: We hav also attached our responses as a word document.

Response to reviewers – second round.
Authors responses in BLUE.

We acknowledged the authors provide additional summaries and test results, which partially addressed the question. As for the visualization (Figure 3A/3B), we agreed the global mean is useful. But we still humbly suggest displaying the distributions (e.g. density or ridge plot) if possible, which provides a more comprehensive view of the data. Our suggestion stems from the following consideration: Global mean may be a good representation when the data follows a Gaussian distribution. But when the underlying distribution is bimodal (e.g. in human's DNA methylation data, most of the sites' methylation level are either close to 0 or 1), global mean is no longer sufficient to represent the whole data. By knowing the global mean, we still cannot say much about the data distribution. As for implementation, If the key point is to display the reference genome's effects on quantification, the authors can consider keeping the current panel layout, where each subplot still represents a group (BTBT, BTBI, etc). Within each subplot, samples in the same group with the same reference genome are summarized by average, leading to the averaged methylation level per site, thus a methylation distribution of all sites can be plotted using a density curve for each reference genome. Such treatment enables a statistical comparison where the significance of 2 density curves' difference can be computed using Kolmogorov-Smirnov test, echoing the quantification bias introduced by reference genome, if there is any. Notably, the global mean (or median) can also be annotated onto the density curve using vertical lines. In that sense, the suggested figure still contains global mean information. Although the per-sample differences (shown in current Figure 3A/3B) are not present in the suggested figure, they are less informative with respect to the key message and can be considered as supplementary figure.

We appreciate the reviewer's detailed comments and suggestions regarding the presentation of figure 3. We have replotted figure 3 as a series of kernel density estimate plots as suggested. Moreover, we also calculated a 2 sample Kolmogorov-Smirnov test for each group to test whether the methylation distribution was different within a given group when using a different reference genome. We have included this at lines 252-277.

"We examined the CpG methylation differences between each sample when mapped to Angus and Brahman reference genomes and observed a statistically significant difference in CpG methylation levels in all samples when using all CpGs with ≥10X coverage (two-sample Kolmogorov-Smirnov test, adjusted p-value < 0.05) (Table 3; S. table 8). When only considering the shared CpGs, we observed a significant quantification bias in all samples, though it was much smaller (two-sample Kolmogorov-Smirnov test, adjusted p-value < 0.05) (Table 3; S. table 8). When we pooled all CpG methylation values for a given group and compared the Brahman and Angus references, we observed a statistically significant quantification bias in all four groups (two-sample Kolmogorov-Smirnov test, adjusted p-value < 0.05) (Figure 3A; Table 3; S. figure 3A; S. table 8). When comparing only the shared CpGs, we observed a much weaker quantification bias between genomes, though it was still significant at the group level (two-sample Kolmogorov-Smirnov test, adjusted p-value < 0.05) (Figure 3B; Table 3; S. figure 3B; S. table 8)."

Inspecting the per-sample differences in Figure 3A/3B, we noticed there is always one sample that has bigger global mean than the rest in each group no matter what reference used (such as sample F97, F8, F46, which behave like outliers as the rest are closer to each other). Is there a potential explanation for this phenomenon? Is it due to biological (origin/age) or technical (different coverage/sequencing depth) reasons?

It is possible those samples had somewhat different sampling from the others. There could be differences in timing, type and number of cells collected from each liver. These spatiotemporal factors may be influencing the difference in methylation of these samples. However, we are not sure why these three samples tend to have a higher global methylation mean than the other samples.

Per author’s comment “a violin plot that spans from 0 – 100% methylation with the widest point around the global mean”, this makes us wonder what the distribution/density curve looks like and why it’s different from other mammals. In other mammals (mouse, human, dog), the majority sites are around 1 or 0, few of them are in the middle, displaying a bimodal distribution. Is it due to the species / tissue / age difference?

We do see a bimodal distribution, though the peaks are around 0 and 0.6 rather than 0 and 1. It could be a combination of tissue and age causing this particular bimodal distribution of methylation. He et al. (2020) investigated methylation levels in the developing mouse embryo across a variety of tissues. For most tissues and timepoints from embryo day 10.5 to adult, the authors observed global methylation levels between 70-80%. Liver was the exception, with global methylation levels dropping to ~60-65% methylation from embryonic day 12.5-16.5. This developmental timepoint in mice is similar to the developmental timepoint of the foetuses in the present study, suggesting the observed methylation pattern may be a hallmark of fetal liver development. At day 153, the cattle fetus begins log phase growth and so cell division is high. Given that hemimethylation increases during DNA synthesis, it is possible that this higher degree of hemimethylation is drawing the global mean methylation more toward 0.5 than 1. We cannot find any comparable studies in cattle with which we can compare our findings to.

We hold a different opinion with author’s argument on mutation rate. Specifically, if we assume the single nucleotide’s mutation rate is uniform across nucleotides (denoted as p) and the mutation events are independent. Then for a dinucleotide, the probability of **not** carrying a mutation is (1-p)^2. In other words, the probability of containing a mutation in a dinucleotide is **1-(1-p)^2**, which is different from the used background mutation rate (p). Such calculation, although oversimplified by making assumptions, shows the current procedure might not be precise.

Nevertheless, since the authors obtained a set of SNPs and want to test if they are enriched in CpG sites. We think an appropriate test is to construct a 2 by 2 contingency table, where the rows record whether a base is in CpG context, and columns denote whether that base is a SNP site or not. The enrichment p-value can then be calculated using chi-squared test.

SNP Not SNP Total
In CpG a b a+b
Not In CpG c d c+d
Total a+c b+d a+b+c+d

For SV’s enrichment, if the goal is to test whether the proportion of CpGs in SV region is higher than that of non-SV region, a natural idea is to calculate the proportions of CpGs for each SV region and each non-SV region respectively, with these 2 groups of proportions, the authors can perform a t-test or Wilcoxon test.

We think these test approaches are better than the binomial test currently employed by the authors since the binomial test heavily reply on the assumptions that the true background probability is known and the CpG occurrence events are independent (that’s why a 1.18-fold will yield p-values as small as lower than 5*10^-324). Such assumptions are too strong and might easily fail in reality, especially for the first one which used the estimated background probability as the true probability and ignores the risk that the background probability could be far from the unobserved truth. The tests we suggested above for SNP and SV respectively have weaker assumptions and should be more suitable for calculating the enrichment significance.

Besides, the enrichment fold is 14 and 1.18 for SNP and SV in the manuscript but is 8.3 and 1.13 in the Jupyter Notebook, we suggested to check and update accordingly.

The Jupyter Notebook was not updated at the time you read it, now it is. The fold enrichment is 14 and 1.18 for SNP and SV, respectively. The authors appreciate the suggestion and effort taken by the reviewers

to provide suggestions for this analysis. As suggested, we reanalysed the SNP enrichment, using a 2x2 contingency table, followed by a Chi-square test. We have amended the manuscript at lines 238-241 and lines 654-660 to reflect these changes.

Lines 238-241:
"SNPs in CpG sites were enriched by ~13 times compared to the genomic background (Chi-square test of independence, adjusted p-value < 5×10^(-324)), which means SNPs between these two breeds affect CpG sites disproportionately more than other autosomal sites."

Lines 654-660:
"We constructed 2×2 contingency tables to perform a Chi-square test of independence per chromosome to determine whether CpGs had a higher likelihood of being affected by a SNP than non-CpGs. The four categories where 'CpG affected by SNP', 'non-CpG affected by SNP', 'unaffected CpG' and 'unaffected non-CpG'. We then adjusted the p-values to correct for multiple testing using the Benjamini-Hochberg correction procedure."

We have also reanalysed the SV CpG enrichment results as suggested and have amended the manuscript at lines 251 to 252 and lines 669-675.

Lines 251-252:
"We observed 1.18-fold more CpGs within SVs than in non-SVs (Mann Whitney U-test, p-value = 8.91×10^(-5))."

Lines 669-675:
"We then counted the number of CpGs that occurred in these regions on each chromosome and divided them by the combined length of all SVs on the given chromosome to determine the proportion of CpGs occurring in the SV regions; this value was added to a vector. We repeated this for CpGs occurring in non-SV regions, adding the proportions to a difference vector. With these two vectors we then performed a Mann-Whitney U test to determine whether SVs were more likely to introduce CpGs than non-SV regions."

The authors provided additional data (Figure 3C and tables) which can effectively address our question in 6b. We only have small plot suggestions. The current scatter plot cannot show where the data points mainly accumulate around, although authors tried to adjust the dot's transparency, but with too many overlapped points, it failed to deliver such information. A replacement with 2d density plot with a diagonal dashed line is suggested. Such density plot is useful to reflect what would happen to most sites if a different reference genome were used (e.g. if the density peak lies above the diagonal line, it shows that using the alternative reference genome would generally inflate the methylation levels). With density plot, all CpG sites can be plotted instead of only that of chromosome 29 as the figure 3C currently shows.

Besides the R2, we recommend reporting the RMSE on the figure, this is because if the key message is that reference genome brings methylation quantification difference, then the overall difference resulted by reference genome is more cared about than the concordance (represented by R2).

We appreciate the suggestion to change Figure 3C to a 2D density plot. We have replotted figure 3C as a KDE plot. While we did use all chromosomes, we present only CpGs that were different by > 10 in Figure 3C. The figure using all CpGs from all chromosomes can be found in supplementary figure 4. We have updated the manuscript at lines 282-284.

"We observed RMSE values of around 0.01 for groups (S. figure 4), with most CpGs exhibiting less than 10% difference in methylation between the reference genomes."

The authors provided additional description and increased clarity, thus addressed our first question/concern in 6d.

For the second and third part, our point is that if we can map the features from one reference genome to another (For shared CpG sites, it's the coordinate mapping derived by the authors; For shared genes, the gene id or gene name), then the methylation level /gene expression matrix that are quantified using one reference genome can be lifted to another reference genome. As a result, the same sample called on different reference genomes can be compared and analyzed on the shared sites/genes. (by treating the quantification results of same biological sample from 2 different reference genomes as paired samples, the usual DMR or DEG analysis strategy can be applied)

Based on the manuscript that less than 2% of shared CpG sites exhibits >=10% methylation when different reference genome used, we speculate there might be few DMRs when comparing the same samples between 2 different reference genomes. Does this suggest that Brahman/Angus reference genome choices are mostly consistent on downstream differential analysis results for the shared sites or genes? And the observed difference is mainly caused by the breed specific CpGs/genes? If that's the case, the authors should explicitly report/discuss it.

We thank the reviewers for their detailed responses. To address this comment, we made non-overlapping 100kb windows of each genome and aligned them to one another. We then filtered the alignments to retain only those that aligned to the same chromosome (e.g. a window from chromosome 1 in Angus aligned to a region of chromosome 1 in Brahman) and those with mapq = 60. With this completed for both references, we could then match 100kb windows between Brahman and Angus. It should be noted here that ~2000 of these windows could not be aligned, thus immediately demonstrating a reference genome effect. For the remaining windows that could be compared, we performed differential methylation analysis. We have updated the manuscript at lines 303-308 and lines 694-710.

Lines 303-308:
"Lastly, we compared homologous regions between the Angus and Brahman reference genomes to determine whether any differentially methylated regions existed between these two genomes for each group (Methods). Using a window size of 100kb, we observed 41, 31, 30 and 36 windows being significantly differentially methylated when using BTBT, BTBI, BIBT and BIBI samples, respectively (paired Wilcoxon test, adjusted p-value < 0.05) (S. Table 11)."

Lines 694-710:
"To further assess the presence of a quantification bias, we generated non-overlapping windows of both Angus and Brahman reference genomes with a length of 100 kb. We then aligned these 100 kb sequences to the opposite reference genome with `minimap2 –t 8 –c –x map-ont`. We then used the output PAF files to identify homolgous 100 kb windows from each genome. We extracted the methylation values from each window for a given group when mapped to each genome. For example, consider homologous-region "A" (HR-A), identified between the Angus and Brahman reference genomes. To identify whether this region exhibits differential methylation between reference genomes and thus exhibits quantification bias, we quantified all methylation values that fell within the Angus coordinates of HR-A using the methylation values for BTBT samples mapped to the Angus reference. We then quantified the methylation values that fell within the Brahman coordinates of HR-A using the methylation values for BTBT samples mapped to the Brahman reference. This gave two vectors of methylation values, which were then used as input to a Wilcoxon signed-rank test to determine whether the methylation values in this region were significantly different between genomes. The Benjamini-Hochberg false discovery procedure was used to adjust for multiple testing. This was repeated for each of the four groups."

We reckoned there might be some miscommunications on this question. Let us rephrase the question. This section titled "Breed-specific CpGs show distinct methylation patterns". By "distinct", we assume the authors imply one or both of the following: 1. the methylation pattern in breed-specific CpGs is different from that of the breed-shared CpGs, 2. the methylation pattern in Brahman-specific CpGs is different from that of the Angus-specific CpGs.

To support the first claim, the authors would need to directly compare the breed specific CpGs methylation

levels to the baseline background (shared CpGs) and see if there is a difference and what's the direction of the difference. To support the second claim, the authors should compare the Brahman-specific CpGs to that of Angus-specific CpGs. Either claim should involve comparing the original values (without bining) of the entire set of CpGs (without subsampling).

Nevertheless, the current procedure, first binned the methylation level into hyper/hypo-methylation by thresholding and then repeated sampling to count the occurrence of hyper/hypo-methylated sites, is essentially showing if the count (or proportion) of hypomethylation sites is higher/lower than that of hypermethylation sites for a given set. Take the figure 3D as an example, the figure shows for breed-specific CpGs, #hypomethylation sites is more than the #hypermethylation sites in both breeds. Our original review questions are: 1. The findings seem to be sensitive to the choice of hyper/hypo-methylation threshold, how was the threshold decided, will the findings still hold if a different threshold used? 2. Using the same threshold, will we get the same findings (#hypomethylation sites is more than the #hypermethylation sites) on the breed-shared sites? If the answer is yes, the authors probably shouldn't claim it to be a distinct pattern for breed-specific sites, since the background (shared sites) also have the same pattern.

In the first response, the authors adjusted the threshold from to 0.25/0.75 and found one reference genome displayed a opposite pattern. This confirmed our first concern that the findings are not robust to threshold choices, leading to concerns that the conclusion claim is not solid or sound. As a result, the current analysis and response **did not** fully resolve the previous questions/concerns.

Additionally, as we denoted above, the current testing procedure (repeated-sampling + Mann-Whitney U-test) is essentially trying to validate if the proportion of hypermethylation sites is the same as that of hypomethylation sites. But this procedure has an issue that the Mann-Whitney U-test's iid assumption will no longer hold under the repeated-sampling procedure. With sufficient repetitions, this procedure will give very small p-values even if the proportion difference is minute. We suggested to switch to testing for the entire set of sites using chi-square test (if the binning procedure is used) or the Wilcoxon test (if the raw methylation values are used).

We have replotted this figure using the raw methylation values via a KDE plot and performed a Wilcoxon test as suggested. We have amended the manuscript at lines 311-319 and lines 1345-1354.

Lines 311-319:
"Looking more closely at the breed-specific CpGs, we first determined the background methylation for Angus samples as the methylation of all shared CpG sites when mapped to the Angus reference. We then extracted the methylation for all Angus samples from CpG sites that were identified as breed-specific. We then compared the background methylation distribution against the Angus-specific distribution. This was repeated for the Brahman using the Brahman reference. Breed -specific CpG sites were significantly more likely to have lower methylation than the background methylation for Angus (Wilcoxon signed-rank test, p < 5.0 ×10^(-324)) and Brahman (Wilcoxon signed rank-test, p < 〖5.0×10〗^(-324)), respectively (Figure 3D-E)."

Lines 1345-1354:
"…D.) KDE plot illustrating the illustrating the difference in methylation distribution between the shared and Angus-specific CpG sites when aligning Angus samples to the Angus reference. Blue represents shared CpG sites. Orange represents Angus-specific CpG sites. P-values were determined with a Wilcoxon signed-rank test. The X-axis denotes the methylation percentage. The Y-axis represents the probability density. E.) KDE plot illustrating the illustrating the difference in methylation distribution between the shared and Brahman-specific CpG sites when aligning Brahman samples to the Brahman reference. Blue represents shared CpG sites. Orange represents Brahman-specific CpG sites. P-values were determined with a Wilcoxon signed-rank test. X- and Y-axes are the same as D."

The author provided that high number of DEGs (>88%) overlapped with DMR, which eliminates the concern about artifacts. Since the canonical correlation we suggested is just a sanity check on data quality, we recommend putting it to supplementary and representing the strength of association by canonical correlation (not R2) with p-values provided, to be consistent with other correlations of the manuscript.

We have created a new supplementary table, 17, to show the CCA results and updated the main text accordingly at lines 796-797.

“Furthermore, we performed canonical correlation analysis (CCA) to estimate the canonical correlation between the gene expression and methylation data. The first five canonical correlations were >0.7 with p-values < 0.05 (S. Table 17).”

“A model without an intercept term would only be recommended in cases where there is a strong biological reason why a zero covariate should be associated with a zero-expression value, and such contexts are rare in gene expression modelling.” Is there a special reason why the intercept is forced to be 0?

We forced the intercept to be zero as we are comparing the gene expression between groups as we implemented the groups-mean parametrisation method to calculate the differentially expressed genes. We have previously published with this method in Liu, Tearle [1]. We have specified this in the main text at lines 788-790.

“The group-mean parameterisation method was used to identify differentially expressed genes between groups with the following model design: ‘~0 + Genetics + Sex + Batch’ [1].”

According to supplementary figure, the putative enhancer region is 25 times longer than the promoter region. It’s not suitable to implicitly suggest a direct comparison between 73% with 0.3%, we suggest adding a short phrase describing the relative fold after normalizing by length.

We have now reported the relative difference between putative enhancer regions and promoter region overlapping DMRs with normalised lengths in each of their respective sections. We have also reported them in a supplementary table (S. table 14).

“Of the 123,602 DMRs observed, ~19% (23,575) overlapped with the surrounding region of the significant DEGs, with around twice as many DMRs falling within the putative-enhancer region compared to the promoter region after normalising for the length difference between the enhancer regions and promoter regions (S. table 14).”

These reciprocal overlap results could be organized into a table if that makes the result look clearer.

We have added S. table 14 to help improve the clarity of the reciprocal overlap results.

The explanation is acknowledged, but we are asking a different question. To illustrate, when deciding whether a region/site is significantly different, we need to compare its p-value to a threshold (e.g. 0.05). When there are many regions/sites to be decided, many p-values need to be compared, then the p-value adjustment (e.g. Bonferroni correction, BH procedure) is needed to account for the multiple comparison problem and control the False discovery rate (FDR).
Back to the question, we speculate the no-findings for DMC result is due to the stringent multiple-testing procedure. Some clarification on the p-value calculation and adjustment method is needed (what test is used? Is the p-value adjusted? if the answer is yes, what adjustment is being used when deciding DMR/DMC?) since there is no such information throughout the manuscript.

We thank the reviewers for their comments. We suspect there has perhaps been a misunderstanding. We identified DMCs using the same method we identified DMRs, though without the sliding window approach. The DMCs were identified using the logistic regression and the p-values were adjusted using a sliding linear model (SLIM) to estimate the proportion of true null hypotheses among the DMC and DMRs. We’ve amended the methods at lines 753-755 to make this clearer.

“Q-values were obtained using the sliding linear model (SLIM) method [2]. We repeated this pipeline to identify DMCs, with the exclusion of the tiling step.”

Line 28-30: “Genetic variation,including single nucleotide polymorphism (SNPs) and structural variants (SVs), can lead to differences in methylation sites (CpGs) between individuals of the same species”. This sentence is a bit ambiguous as the differences can be resulted either by mQTL or by reference genome’s artifact. It’s better to be specific and precise.

We have changed this to: “Reference genome artefacts and genetic variation,…”

Line 39-40: “Furthermore, we found that SNPs and SVs were 14-fold (p-value < 5 × 10−324) and 1.18-fold (p-value < 5 × 10−324) higher in CpGs,”. The sentence is a bit confusing as the proportion of CpGs is higher in SVs, not the other way around.

We have updated lines 39-41 to improve clarity.
“Furthermore, we found that SNPs impacted CpGs 13 times more than other autosomal sites (p-value < 5×10^(-324)) and SVs contained 1.18 times (p-value < 5×10^(-324)) more CpGs than non-SVs.”

Line 41-43: “We found a poor association between differentially methylated regions (DMRs) and differentially expressed genes (DEGs) and suggest that DMRs may be impacting enhancers that target these DEGs”. The “association” is suggested to be replaced by “overlap” to avoid confusion.

We have updated the line as suggested at line 41.

“We found a poor overlap between differentially methylated…”

Line 162-164: Only the average mapping rates for Brahman reference genome is reported, we suggest also write out the mapping rate of Angus to make it complete.

Done. “An average mapping rate of ~95% was achieved when reads were mapped from each sample to the Brahman reference genome and ~93% when mapping to the Angus reference genome (Table 1; S. table 1).”

Line 196-197: “indicating a potential Parent-of-origin effects” can be added to the end of the paragraph.
Done. “The separation of groups we observed from the methylation data was similar to that seen for the gene expression data (Fig 2B), indicating a potential parent-of-origin effect.”

Line 219: “four per cent” -> 4%, to be consistent with the format in line 223/225.

We have changed to 4% as suggested.

“We found that around 4% of CpG alignments…”

Line 313: “CpGs that were between 35% and 65% were considered “hemimethylated””. “hemimethylated” specifically refers to the methylation pattern of CpG sites (one strand (forward/reverse) is methylated while the other strand (reverse/forward) is not). We recommend changing to another word that more precisely denotes the sites whose methylation level lies in between the binning threshold.

This has been addressed above.
Lines 311-319:
“Looking more closely at the breed-specific CpGs, we first determined the background methylation for Angus samples as the mean methylation of all shared CpG sites when mapped to the Angus reference. We then extracted the methylation for all Angus samples from CpG sites that were identified as breed-specific. We then compared the background methylation distribution against the Angus-specific distribution. This was repeated for the Brahman using the Brahman reference. Breed -specific CpG sites were significantly more likely to have lower methylation than the background methylation for Angus (Wilcoxon signed-rank test, p < 5.0 ×10^(-324)) and Brahman (Wilcoxon signed rank-test, p < 〖5.0×10〗^(-324)), respectively (Figure 3D-E).”

Line 492: “Most DMRs identified in this study were not associated with DEGs”, we suggested to replace “associated” with “overlapped” to make it more precise.

Completed at line 483. “Most DMRs identified in this study did not overlap with DEGs.”

Some of url links (line 595/597/607) are underlined while (610/613/614) some are not, it’s better to keep it consistent throughout the paper.

Done. All URL links have been removed (i.e. they are plain text now).

Line 628: 102bp -> 1002bp

102bp is correct at this line. This was the length of the query sequence when we performed the local alignment within the ~1002bp block identified by minimap2.

Line 640: “To determine the accuracy of minimap2 in identifying SNPs”, minimap2 is a read aligner not a SNP-caller, this sentence is not precise. Throughout the manuscript, minimap2 is constantly referred as a SNP-caller, we suggest the authors revise thoroughly and make necessary corrections.

We have updated the manuscript as follows to improve the clarity of this section:
“To determine the accuracy of minimap2 alignments in identifying SNPs between the Brahman and Angus genomes, we first introduced artificial mutations into each genome using SNP Mutator [103].”

“Alignments from Mminimap2 [59] and were used as input to PAFtools which was were used to identify variants between the original and mutated sequences.”

Table1: “Mapped reads” and “CpGs covered with >= 10x” can be formatted as “counts (percentage)”, currently it only displayed the counts

Done.

Supplementary Figure2 legend: “with “other” SNPs making up ~19% of SNPs.” -> with other alternations making up ~19% of SNPs.

Done.

Figure 3D’s legend “D.) Boxplots illustrating the mean frequencies of methylation states observed in Angus and Brahman after 100 permutations.”. This is confusing since there is no permutation being done. The sub-sampling procedure is not equivalent to permutation.

The figure has been removed. We believe this has been resolved when we addressed prior comments.

1. Liu R, Tearle R, Low WY, Chen T, Thomsen D, Smith TPL, et al. Distinctive gene expression patterns and imprinting signatures revealed in reciprocal crosses between cattle sub-species. BMC Genomics. 2021;22 1 doi:10.1186/s12864-021-07667-2.
2. Wang H-Q, Tuominen LK and Tsai C-J. SLIM: a sliding linear model for estimating the proportion of true null hypotheses in datasets with dependence structures. Bioinformatics. 2010;27 2:225-31. doi:10.1093/bioinformatics/btq650.
