# Supplementary material for: DNA methylation analysis to differentiate reference, breed, and parent-of-origin effects in the bovine pangenome era
Source: Gigascience. 2024 Oct 17;13:giae061. doi: 10.1093/gigascience/giae061 (PMC11484048; doi:10.1093/gigascience/giae061)
Supplement: giae061_Response_to_Reviewer_Comments_Revision_2 [file giae061_response_to_reviewer_comments_revision_2.pdf]

Author's Response To Reviewer Comments

We have replaced URLs with reference numbers at lines:

555 - and followed the ARRIVE Guidelines [96] (Approval No. S-094-2005).
581 to 583 - The reads were first checked for quality with FastQC (v. 0.11.9) (RRID:SCR_014583) [99], then adapters were trimmed using Trim Galore (v. 0.6.6) (RRID:SCR_011847) [100]
592 - duplicates were marked with Picard (v. 2.25.4) (RRID:SCR_006525) [103].
595 to 596 - MethylDackel (v. 0.5.2) [105] extract with the parameter
772 to 773 - Briefly, reads were checked for quality using FastQC (v. 0.11.4) [99] before being trimmed with Trim Galore (v. 0.4.2) [100]

We have added the Availablity of Source code and requrements section at lines 829 to 839:

Availability of Source Code and Requirements
1. Project name: Brahman_Angus_WGBS
2. Project home page: https://github.com/DaviesCentreInformatics/Brahman_Angus_WGBS
3. Operating system(s): Linux and MacOS
4. Programming language: Python, R
5. Other requirements: NA
6. License: MIT
7. Any restrictions to use by non-academics: No restrictions
8. RRID: NA
9. bio.tools ID: NA

880 - and followed the ARRIVE Guidelines [96] (Approval No. S-094-2005).

We have added abbreviations at lines 847 to 876
Abbreviations
3’UTR - Three prime untranslated region
5’UTR - Five prime untranslated region
BIBI - Brahman sire and Brahman dam
BIBT - Brahman sire and Angus dam
BTBI - Angus sire and Brahman dam
BTBT - Angus sire and Angus dam
CCA - Canonical correlation analysis
CpG - Cytosine phosphodiester-linkage Guanine
DEGs - Differentially expressed genes
DGAT1 - Diacylglycerol O-acyltransferase 1
DSCAM - DS cell adhesion molecule
HGNC - Human Genome Organisation Gene Nomenclature Committee
HTR2A - 5-hydroxytryptamine receptor 2A
IGF2 - Insulin-like growth factor 2
Indel - Insertion/deletion
LIN2B - Lin-28 homologous B
MKRN3 - Makorin ring finger protein 3
NDN - necdin MAGE family member
NNAT - Neuronatin
PARD6G - Par-6 family cell polarity regulator gamma
POE - Parent-of-origin effect
PPP1R9A - protein phosphate 1 regulatory subunit 9A
SLC22A1 - Solute-carrier family 22 member 18
SNP - Single nulceotide polymorphism

SV - Structural variant
TFPI2 - tissue factor pathway inhibitor 2
TpG - Thymine phosphodiester-linkage Guanine
WGBS - Whole genome bisulfite sequencing
ZC3H12C - Zinc finger CCCH-type containing 12C
