# Supplementary material for: DNA methylation analysis to differentiate reference, breed, and parent-of-origin effects in the bovine pangenome era
Source: Gigascience. 2024 Oct 17;13:giae061. doi: 10.1093/gigascience/giae061 (PMC11484048; doi:10.1093/gigascience/giae061)
Supplement: giae061_Reviewer_2_Report_Original_Submission — Alan L Archibald -- 12/19/2023 Reviewed [file giae061_reviewer_2_report_original_submission.pdf]

Reviewer Report

Title: DNA methylation analysis to differentiate reference, breed and parent-of-origin effects in the bovine pangenome era

Version: Original Submission **Date:** 12/19/2023

Reviewer name: Alan L Archibald

Reviewer Comments to Author:

This manuscript is a well-written and readable account of a study of methylation in two subspecies of cattle - Bos taurus and Bos Indicus. There is a specific focus on the effect of which reference genome assembly is used as the framework for the analyses. The authors demonstrate that the reference genome used can introduce a bias, albeit a relatively modest bias in global terms. Whilst the 2% bias can be considered modest at a whole genome scale, it can be critical when considering individual genes or regulatory sequences.

The average mapping rate of CpG with at least 10% coverage to the Brahman and Angus reference assemblies is reported in S Table 1 (94.95% and 92.49%, respectively), but only the average mapping rate to the Brahman assembly is reported in the main text. Interestingly, a higher proportion of the Angus (Bos taurus) CpGs with at least 10% coverage map to the Brahman assembly than to the Angus assembly (94.83% and 92.75% respectively on average). In contrast, as one might predict a smaller proportion of the Brahman CpGs with at least 10% coverage map to the Angus assembly than to the Brahman assembly. Some comment on the apparent anomaly in the mapping rate for the Angus reads might be appropriate.

The authors highlight the bias that may be introduced by using an inappropriate assembly as the framework for epigenetic and other analyses. The coming pangenomes are presented as a potential solution without demonstrating that solution in this particular study. Perhaps a comment on other solutions such as the generation of experimental population specific genome assemblies as a framework for analyses. What will be the impact of the use of long read sequencing data that includes scoring of modified bases in epigenetic analyses?

The manuscript is suitable for publication after minor changes.

Given the small number of individuals used in this study the term "breed-specific" should be used with caution. Perhaps the term and its usage in the paper should be explained, complete with caveats, in the introductory text.

Historically, a sample size of 20-25 UNRELATED individuals was considered an appropriate sample of a population in breed genetic diversity studies.

The number of individuals sampled per breed or crossbreeds in this study is a fraction of this recommended sample size. Moreover, no information is provided on the degree of relatedness between the individuals sampled.

The WGBS and transcriptomics data used for the analyses are available in the public domain at PRJNA626458.

The gene symbol nomenclature for cattle follows the nomenclature style used for human genes. Thus,

all gene symbols should be fully capitalised as well as italicised.

Methods

Are the methods appropriate to the aims of the study, are they well described, and are necessary controls included? Choose an item.

Conclusions

Are the conclusions adequately supported by the data shown? Choose an item.

Reporting Standards

Does the manuscript adhere to the journal's guidelines on minimum standards of reporting? Choose an item.

Choose an item.

Statistics

Are you able to assess all statistics in the manuscript, including the appropriateness of statistical tests used? Choose an item.

Quality of Written English

Please indicate the quality of language in the manuscript: Choose an item.

Declaration of Competing Interests

Please complete a declaration of competing interests, considering the following questions:

- Have you in the past five years received reimbursements, fees, funding, or salary from an organisation that may in any way gain or lose financially from the publication of this manuscript, either now or in the future?
- Do you hold any stocks or shares in an organisation that may in any way gain or lose financially from the publication of this manuscript, either now or in the future?
- Do you hold or are you currently applying for any patents relating to the content of the manuscript?
- Have you received reimbursements, fees, funding, or salary from an organization that holds or has applied for patents relating to the content of the manuscript?
- Do you have any other financial competing interests?
- Do you have any non-financial competing interests in relation to this paper?

If you can answer no to all of the above, write 'I declare that I have no competing interests' below. If your reply is yes to any, please give details below.

'I declare that I have no competing interests'

I agree to the open peer review policy of the journal. I understand that my name will be included on my report to the authors and, if the manuscript is accepted for publication, my named report including any attachments I upload will be posted on the website along with the authors' responses. I agree for my report to be made available under an Open Access Creative Commons CC-BY license (http://creativecommons.org/licenses/by/4.0/). I understand that any comments which I do not wish to be included in my named report can be included as confidential comments to the editors, which will not be published.

Choose an item.

To further support our reviewers, we have joined with Publons, where you can gain additional credit to further highlight your hard work (see: https://publons.com/journal/530/gigascience). On publication of this paper, your review will be automatically added to Publons, you can then choose whether or not to claim your Publons credit. I understand this statement.

Yes Choose an item.
